# Unveiling an Alternative Mechanism for Lewis Basic Selenium as a C─H Hydrogen Bond Catalyst and Its Application in the Halogenation of Arenes

**DOI:** 10.1002/anie.202511770

**Published:** 2025-08-10

**Authors:** Jingxian Huang, Qingyu Zhang, Haihui Wang, Junjie Yang, Ying‐Lung Steve Tse, Xiaojian Jiang, Ying‐Yeung Yeung

**Affiliations:** ^1^ Department of Chemistry and State Key Laboratory of Synthetic Chemistry The Chinese University of Hong Kong Shatin, NT Hong Kong China; ^2^ State Key Laboratory of Bioactive Molecules and Druggability Assessment Guangdong Basic Research Center of Excellence for Natural Bioactive Molecules and Discovery of Innovative Drugs College of Pharmacy Jinan University Guangzhou 510632 China

**Keywords:** Halogenation, Homogeneous catalysis, Hydrogen bonds, Lewis bases, Selenium

## Abstract

Electrophilic halogenation is a fundamentally useful transformation, and Lewis basic selenium catalysts together with *N*‐haloimides are known to promote the reaction under mild conditions. Selenourea is a frequently used platform for the Lewis base catalyst design. In literature, it is generally believed that the Lewis basic selenium can form a Lewis adduct with the halogen as the active halogenating species. Herein, we report our recent finding that unveils an alternative mechanism of this type of reaction. It was found that the Lewis basic selenourea is first oxidized by the halogen source to give a benchtop stable and non‐hygroscopic imidazolium hypervalent selenium dibromide compound that acts as the actual catalyst. The negative charge in the hypervalent selenium compound is stabilized by the electronegative bromide, leading to the site‐isolated and unoccupied imidazolium cationic moiety. Mechanistic studies suggest that the electropositive C–Hs of the imidazolium cation can act as a strong hydrogen bond donor to activate the *N*‐haloimide reagent for efficient halogenation reaction, including late‐stage halogenation of drug molecules, with low catalyst loading. Further study revealed that hypervalent imidazolium selenium dibromide can generally be applied to other mechanistically unrelated hydrogen bond‐catalyzed reactions.

## Graphical abstract

This work demonstrates an unconventional mechanism in Lewis basic selenium‐catalyzed electrophilic halogenation, where in situ oxidation of selenourea by the halogen source generates hypervalent Se‐imidazolium species. These intermediates function as hydrogen‐bond catalysts to activate *N*‐haloamide reagents, enabling efficient and mild halogenation of arenes, particularly valuable for late‐stage bromination of pharmaceuticals.

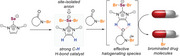

## Introduction

The electrophilic halogenation of unsaturated compounds is a crucial process to synthesize halogenated molecules.^[^ ^1^, ^2^, ^3^, ^4^, ^5^, ^6^, ^7^, ^8^ ^]^ The resultant halogenated building blocks have extensive applications across multiple fields, including agrochemicals, pharmaceuticals, optoelectronic materials, and the post‐translational modification of oligopeptides.^[^ ^9^, ^10^, ^11^, ^12^, ^13^, ^14^, ^15^, ^16^ ^]^ A notable instance of this is the halogenation of aromatic compounds, which leads to the formation of haloarenes.^[^ ^17^, ^18^, ^19^, ^20^, ^21^, ^22^, ^23^, ^24^, ^25^, ^26^, ^27^, ^28^, ^29^, ^30^, ^31^, ^32^ ^]^ Haloarenes play a vital role in contemporary drug development, proving indispensable in pharmaceutical research due to their ability to improve drug efficacy and stability. A significant number of newly developed drugs continue to integrate halogen atoms.^[^ ^33^, ^34^, ^35^ ^]^ These compounds are particularly common in FDA‐approved medications, clinical trial candidates, and top‐selling pharmaceuticals.^[^ ^36^, ^37^ ^]^

Molecular halogens (e.g., Br_2_) serve as cost‐effective halogenating agents. However, their volatility and high corrosiveness present significant challenges, including poor compatibility with various functional groups, limited chemo‐ and regioselectivity, and considerable safety concerns.^[^ ^38^ ^]^ *N*‐haloamides (e.g., *N*‐bromosuccinimide, NBS) are benchtop stable solid compounds that are commonly employed as alternatives to molecular halogens. Despite their utility, these compounds exhibit relatively low reactivity, necessitating the use of external activators in numerous reactions.^[^ ^18^, ^39^ ^]^ Recent research has highlighted the application of soft selenium Lewis bases as activators,^[^ ^40^, ^41^, ^42^, ^43^, ^44^ ^]^ attributed to their ability to facilitate reactions under mild conditions, their compatibility with a wide range of functional groups, and the capacity to achieve good selectivity through fine‐tuning the catalyst structure.

**Scheme 1:**
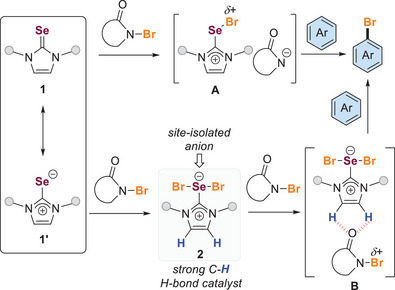
Typical mechanism of Lewis basis selenium‐catalyzed halogenation and our study to unveil an alternative mechanism.

In the conventional design of Lewis base chalcogen catalysts, it is common to integrate electron‐donating groups in conjunction with chalcogen atoms. One notable motif employed in catalyst design is chalcogen‐urea.^[^ ^45^, ^46^ ^]^ For example, selenourea **1** serves as a prominent catalyst motif and the Lewis basicity of the selenium is enhanced through electron delocalization from the nitrogen atoms to give the resonance structure **1′**. In the widely accepted mechanism, it is believed that the n→σ* interaction^[^ ^40^, ^47^ ^]^ between the soft Lewis basic chalcogen (e.g., Se in **1**) and the halogen of *N*‐haloamides (e.g., NBS) facilitates the dissociation of the amide anion (Scheme 1). This process results in a redistribution of electron density, leading to the formation of a cationic chalcogen–halogen adduct **A**, which might act as the active species. Subsequently, unsaturated substrates such as arenes could interact with the σ‐hole of the activated halogen species **A**, resulting in the production of the corresponding halogenated compounds.

As an ongoing research interest in Lewis base‐catalyzed halogenation,^[^ ^48^, ^49^, ^50^, ^51^, ^52^, ^53^, ^54^ ^]^ we have further examined the reaction mechanisms associated with this kind of process. Here, we report our recent findings, which reveal that the Lewis basic chalcogen‐catalyzed halogenation reaction can proceed through an alternative mechanism, which has not been previously addressed in the literature. Our research suggests that the Lewis basic selenium catalyst might undergo oxidation to form hypervalent selenium **2**, which could act as a C─H hydrogen bond catalyst (Scheme 1). Consequently, the activation of *N*‐haloamide reagents appears to occur through C─H···O non‐classical hydrogen bond (NCHB) interaction^[^ ^55^, ^56^, ^57^ ^]^ rather than Lewis base catalysis. The newly developed catalytic protocol is applicable to the late‐stage halogenation of drug molecules. These findings establish a robust foundation for subsequent investigations in the field of electrophilic halogenation reactions involving Lewis basic chalcogens.

## Results and Discussion

**Scheme 2:**
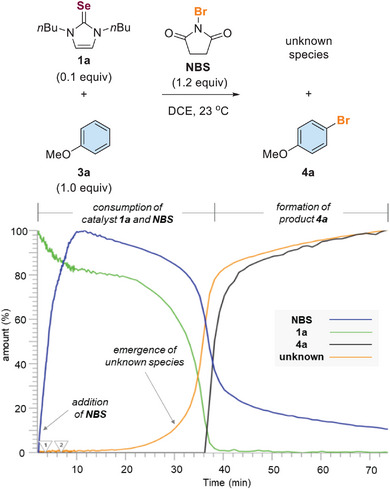
In situ IR of Lewis base catalyzed anisole bromination.

Our study commenced with aromatic halogenation, which is a highly useful process for giving haloarene building blocks for various applications.^[^ ^17^, ^18^, ^19^, ^20^, ^21^, ^22^, ^23^, ^24^, ^25^, ^26^, ^27^, ^28^, ^29^, ^30^, ^31^, ^32^ ^]^ The butylated selenourea catalyst **1a** was used because of its high solubility. Anisole (**3a**) and NBS were used as the model substrate and halogen source, respectively. In situ IR was used to monitor the reaction in real‐time and collect useful mechanistic data. Catalyst **1a** was consumed gradually upon the addition of NBS (Scheme 2). However, the desired product 4‐bromoanisole (**4a**) was not detected and substrate **3a** remained unconsumed. Instead, an unknown species emerged upon the consumption of catalyst **1a**. NBS was consumed dramatically and the bromination of **3a** proceeded after the unknown species was generated, suggesting that the unknown species was crucial in activating NBS and promoting halogenation.

**Scheme 3:**
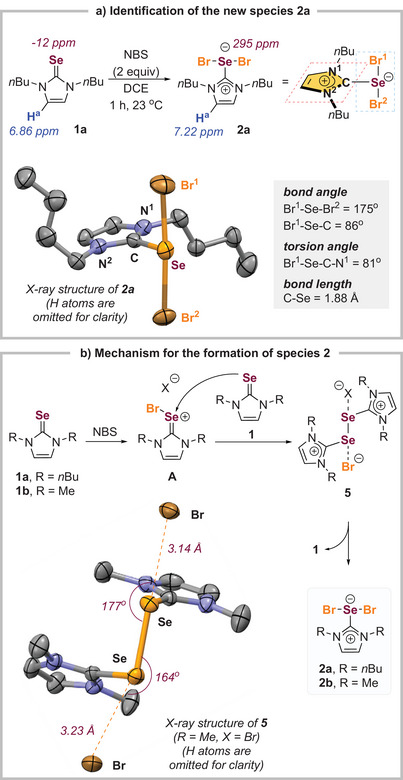
Identification of the actual catalytic species. a) Identification of the new species **2a**. b) Mechanism for the formation of species **2**.

After extensive experimentations, the unknown species was identified to be imidazolium selenodibromide **2a** and it was a benchtop stable solid (Scheme 3a). **2a** could be prepared by reacting catalyst **1a** and NBS alone, suggesting that the new species **2a** was formed independently from the substrate. Upon the addition of 2 equivalents of NBS, the H^a^ signal of the imidazolium hydrogen exhibited a downfield shift from 6.86 to 7.22 ppm, while the ^77^Se signal shifted from −12 to 295 ppm. These data resemble the formation of the imidazolium cationic moiety and hypervalent selenium. The structure of **2a** was further confirmed by an X‐ray crystallographic study on its single crystal.^[^ ^58^ ^]^ The Br─Se─Br (bond angle = 175°) system is almost linear and is orthogonal to the imidazolium planar (torsion angle = 81°). The C─Se bond length (1.88 Å) in **2a** is much longer than the typical C─Se double bond (1.71 Å),^[^ ^59^ ^]^ suggesting that the C─Se in **2a** has a significant single bond character. The structure of **2a** could be stabilized due to: 1) the electron sharing by the non‐bonding electron of Br to the empty p‐orbital of the sp^2^‐hybridized carbon in the imidazolium; 2) the 3c‐4e Br─Se─Br system in the hypervalent selenium (Figure S8 for the orbital analysis).^[^ ^60^, ^61^, ^62^, ^63^ ^]^

Next, we investigated the mechanism underlying the formation of compound **2** during the reaction. The reaction between selenourea **1a** and NBS was rapid, yielding limited insights from spectroscopic studies. In contrast, the methylated selenourea **1b** exhibited reduced solubility and a slower rate of active species formation, which was advantageous for our mechanistic investigations (Scheme 3b). Upon the gradual addition of NBS to selenourea **1b**, the latter dissolved and was consumed over time, resulting in the formation of a precipitate identified as bis‐imidazolium **5** through X‐ray crystallographic analysis.^[^ ^58^ ^]^ However, with the introduction of additional NBS, the *bis*‐imidazolium **5** precipitate re‐dissolved, leading to the generation of hypervalent selenium dibromide **2b**, confirmed via X‐ray crystallography.^[^ ^58^ ^]^ These findings suggest that the formation of compound **2** occurs through intermediate **5**.

According to some literature reports, the diselenide compounds analogous to **5** can be synthesized through the reaction of imidazolidine‐2‐selone with molecular halogen, which can subsequently be transformed into the hypervalent selenium species.^[^ ^64^, ^65^ ^]^ Thus, we believe that the Lewis basic selenourea **1** might first engage in a reaction with NBS to yield the Se–Br complex **A** (Scheme 3b). Rather than reacting with the arene substrates, an additional molecule of imidazole‐2‐selenone **1** could react with complex **A**, resulting in the formation of the *bis*‐imidazolium diselenide **5**. Given that NBS was present in excess relative to the selenourea catalyst **1** (10 mol%) in the reaction environment, it was anticipated that there would be ample NBS available to consume selenourea **1** fully. It is possible that some NBS may gradually decompose via hydrolysis or disproportionation to give molecular bromine, while the succinimide anion in **5** could exchange with bromide (Figure S6). The disproportionation of **5** might lead to the generation of **2**, which would act as the active catalytic species.

**Scheme 4:**
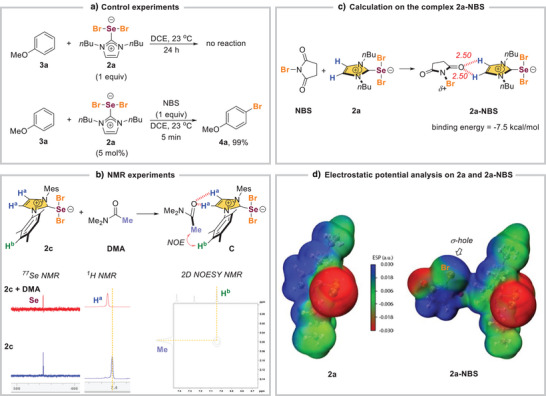
Mechanistic studies. a) Control experiments. b) NMR experiments. c) Calculation on the complex **2a‐NBS**. d) Electrostatic potential analysis on **2a** and **2a‐NBS**.

We subjected **2a** to the bromination of anisole (**3a**) in the absence of NBS and no reaction was observed. This suggests that the Br in **2a** was unlikely to serve as the electrophilic brominating source (Scheme 4a). The uncatalyzed reaction of **3a** with NBS was also negligible. However, when 5 mol% of **2a** was added to the mixture of **3a** and NBS, the reaction completed within 5 min and **4a** was obtained quantitatively, indicating that **2a** could be the actual catalyst in the reaction. Further studies were performed to probe the activation mode of the reaction. *N*,*N*‐Dimethylacetamide (DMA) that serves as a stronger electron donor to mimic succinimide (Figure S3) and probe the interaction with **2** (Scheme 4b). ^77^Se NMR experiment was performed on a mixture of **2c** and DMA, and no significant change in the Se signal was detected. Instead, the C(sp^2^)–H^a^ (imidazolium hydrogen) of **2c** has a considerable chemical shift in the ^1^H NMR (Figure S4). These results suggested that the imidazolium's hydrogens in **2** might be the interaction site. Further analysis was performed using 2D NOESY NMR experiment **2c** and DMA (mixing time = 200 ms) (Figure S5). A significant NOE between the acetamide's methyl group of DMA and the aromatic C─H^b^ in the mesityl group of **2c** was observed. The results from these NMR experiments could be attributed to the C─H···O NCHB^[^ ^55^, ^56^, ^57^ ^]^ interaction between the imidazolium C─H and the Lewis basic oxygen of DMA, which could bring the acetamide and the mesityl group into close proximity (i.e., species **C**).

We performed DFT calculations to better understand the molecular interactions. The single‐point energy calculations // optimization were carried out at the ωB97M‐V/def2‐QZVPP // B3LYP‐D3(BJ)/def2‐SVP levels (see Supporting Information for details).^[^ ^66^, ^67^, ^68^, ^69^, ^70^ ^]^ A complex **2a‐NBS** was optimized by our DFT calculations to give a binding energy of −7.5 kcal mol^−1^ (Scheme 4c). The C─H···O bond length (2.50 Å) was found to be shorter than the sum of their van der Waals radii. Electrostatic potential analysis on **2a** indicates that the negative charge is localized at the SeBr_2_ moiety (Scheme 4d, see Supporting Information for details).^[^ ^71^, ^72^ ^]^ The relatively electronegative bromine atoms could stabilize the anionic charge on the selenium, leading to a site‐isolated and unoccupied imidazolium cation that could serve as strong C─H NCHB donors (blue region in Scheme 4d) to interact with NBS to give the complex **2a‐NBS**. Based on the calculation, the electrophilicity of Br in NBS is increased significantly after the formation of **2a‐NBS** (Figure S9). Although other activation modes (e.g., chalcogen bonding)^[^ ^73^, ^74^ ^]^ cannot be completely ruled out, the data presented above support a NCHB‐mediated catalytic mechanism.

**Scheme 5:**
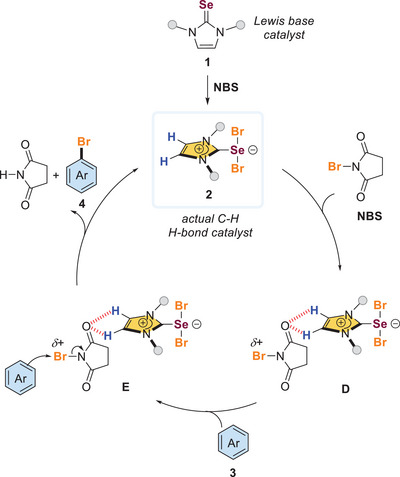
A postulated reaction mechanism.

According to the abovementioned control experiments and mechanistic studies, we believe that Lewis basic selenourea **1** did not promote the electrophilic halogenation directly (Scheme 5). Instead, the selenourea **1** could first react with NBS to give the hypervalent selenodibromide **2**. The site‐isolated imidazolium cation in **2** could serve as the actual catalyst core. Thus, catalyst **2** might activate NBS via C─H···O interaction to give species **D**. Subsequently, the arene substrates **3** could react with the electrophilic Br via species **E** to give the halogenated arenes **4** together with the regeneration of the catalyst **2**.

**Scheme 6:**
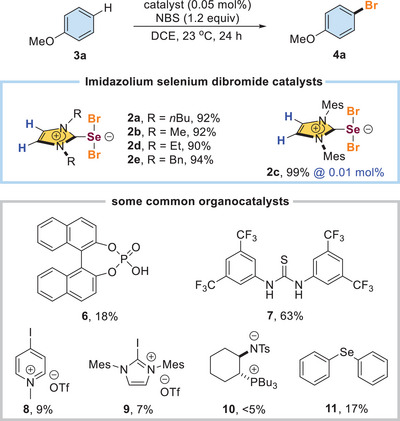
Evaluation of the catalytic performance.

After verifying that **2** was the actual catalyst in the halogenation, we studied various catalysts (0.05 mol%) to compare their performance (Scheme 6). It was found that the performance of **2a–2e** generally worked well. In particular, catalyst **2c** bearing mesityl as the substituents gave the best performance and its catalyst loading could be further reduced to 0.01 mol%. We also examined a number of common organocatalysts, including phosphoric acid **6**, thiourea **7**, halogen bond catalysts **8** and **9**, zwitterion **10**, and Lewis basic selenide **11**, and their performances were far inferior to that of **2**.

**Scheme 7:**
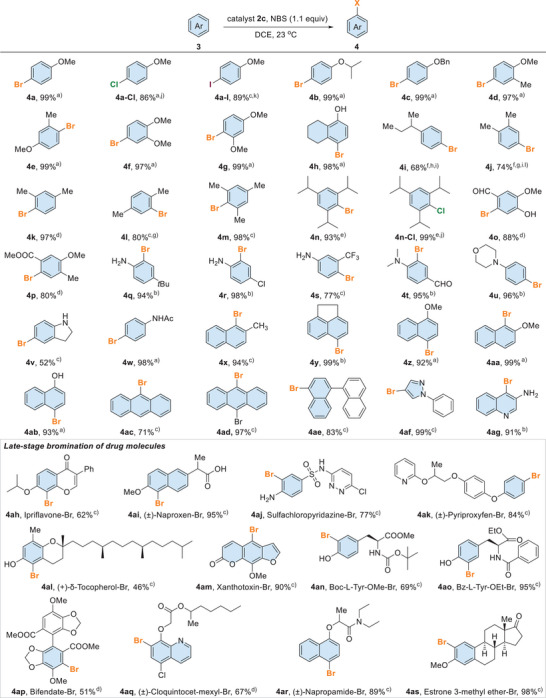
Substrate scope study. Reactions were carried out with substrate **3** (0.10 mmol), catalyst **2c**, and NBS (0.11 mmol) in DCE (0.2 mL) at 23 °C. ^a)^ 0.01 mol% of **2c** for 1 h. ^b)^ 0.05 mol% of **2c** for 1 h. ^c)^ 0.1 mol% of **2c** for 6 h. ^d)^ 5 mol% of **2c** for 12 h. ^e)^ 2 mol% of **2c** for 8 h in MeCN (2.0 M). ^f)^ 10 mol% of **2c** for 12 h. ^g)^ Reaction time was 40 h. ^h)^ Reaction time was 72 h. ^i)^ MeCN instead of DCE. ^j)^ NCS instead of NBS. ^k)^ NIS instead of NBS. ^l)^ The *para*/*ortho* selectivity = 7:1.

Next, catalyst **2c** (0.01 mol%) was used to examine the substrate scope of electrophilic aromatic halogenation (Scheme 7). In general, the catalytic protocol could be applied to various arene substrates **3** and excellent yields and regioselectivity were obtained. For instance, alkoxy (methoxy, isopropoxy, benzyloxy) arenes **3a**–**3e** were brominated to give the corresponding brominated arenes **4a**–**4e** smoothly. Dialkyl ether substrates **3f**−**3g** and phenol **3h** also worked well under the optimized catalytic protocol. The less reactive substrates bearing alkyl (**3i**–**3n**) or electron‐withdrawing (**3o**−**3p**) groups were examined. To our delight, these substrates could be halogenated using a relatively higher loading of catalyst **2c**. Less hindered alkyl substrates typically suffer from difficulty in controlling the regioselectivity.^[^ ^75^, ^76^ ^]^ However, good regioselectivity was obtained using **2c** as the catalyst and NBS as the halogen source. Alkyl substrates, including *s*‐butylbenzene (**3i**), xylenes **3j**–**3l**, and mestylene (**3m**) were examined, and their corresponding mono‐brominated products **4i**–**4m** were obtained with excellent regioselectivity (Figure S7). The triisopropylbenzene (**3n**) was very bulky and hard to functionalize. Nonetheless, under the optimized conditions, it could also be brominated to give **4n**. Other than ether, nitrogen‐substituted substrates, including aniline **3q**–**3u**, indoline (**3v**), and phenyl acetamide (**3w**) were also compatible to give **4q**–**4w** in excellent yield. Aldehyde was susceptible to oxidation with halogen sources.^[^ ^77^ ^]^ Nonetheless, the aldehyde in **4o** and **4t** remained intact. Moreover, alkyl naphthalenes **3x**–**3y**, naphthyl ethers **3z**–**3aa**, and naphthanol **3ab** could be brominated to give **4x**–**4ab** with excellent yields and regioselectivity. Polyaromatic compounds, including anthracenes **3ac**–**3ad** and binaphthalene **3ae** were also studied. It is typically non‐trivial to control the bromination because there are multiple bromination sites.^[^ ^17^, ^18^, ^19^, ^20^, ^21^, ^22^, ^23^, ^24^, ^25^, ^26^, ^27^, ^28^, ^29^, ^30^, ^31^, ^32^ ^]^ Nonetheless, mono‐brominated products **4ac**–**4ae** were obtained in satisfying conversions. Heteroaromatic compounds such as **3af** and **3ag** were also compatible, giving brominated pyrazole **4af** and quinoline **4ag** in excellent yields. Other than bromination, we also examined the catalytic protocol in selected substrates using other halogen sources, including *N*‐chlorosuccinimide (NCS) and *N*‐iodosuccinimide (NIS), and the corresponding chlorinated and iodinated products **4a‐Cl**, **4a**‐**I**, and **4n‐Cl** were obtained smoothly.

Brominated compounds have emerged as promising tools in medicine,^[^ ^36^, ^37^ ^]^ leveraging bromine's distinct advantages for diagnostic imaging and radiotherapy. Compared to fluorine and iodine, bromine isotopes offer a good balance; they have longer half‐lives than fluorine and greater in vivo stability than iodine, making them particularly suitable for PET imaging.^[^ ^78^, ^79^, ^80^ ^]^ In addition, bromine incorporation can strengthen a drug's target binding affinity via halogen bonding while also extending its duration of action, ultimately improving therapeutic performance.^[^ ^81^, ^82^ ^]^ Thus, we have utilized the newly developed catalytic protocol to perform late‐stage bromination of several valuable drug molecules and bioactive compounds, including Ipriflavone,^[^ ^83^ ^]^ Naproxen,^[^ ^84^ ^]^ Sulfachloropyridazine,^[^ ^85^ ^]^ Pyriproxyfen,^[^ ^86^ ^]^ Tocopherol,^[^ ^87^ ^]^ Xanthotoxin,^[^ ^88^ ^]^ Bifendate,^[^ ^89^ ^]^ Cloquintocet,^[^ ^89^ ^]^ Napropamide,^[^ ^90^ ^]^ Estrone,^[^ ^91^ ^]^ and tyrosine derivatives.^[^ ^92^ ^]^ These drug molecules contain multiple functional groups that are sensitive to base, acid, and oxidant. Nonetheless, all these compounds were successfully mono‐brominated to give **4ah**–**4as** with good regioselectivity.

**Scheme 8:**
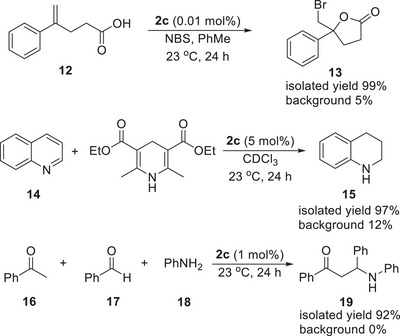
Study on other hydrogen bond‐catalyzed reactions.

After demonstrating that the hypervalent selenium dibromide **2** could be a potent hydrogen bond catalyst to activate NBS, we envisioned that **2** could catalyze other halogenations and mechanistically unrelated reactions. Thus, **2c** (0.01 mol%) was used to promote the electrophilic bromolactonization of carboxylic acid **12** to give the corresponding brominated lactone **13** quantitatively (Scheme 8). Catalyst **2c** was also applied to activate quinoline (**14**) in the Hantzsch ester reduction to give dihydroquinoline **15** in 97% yield. The catalyst was also applied to the Mannich reaction using **16**–**18**, and amine **19** was obtained smoothly. These results further support the fact that compound **2** acted as a hydrogen bond catalyst instead of a Lewis base.

## Conclusion

In summary, we have unrevealed that Lewis basic selenourea can be oxidized by NBS to form hypervalent Se–imidazolium species, which act as effective catalysts for halogenation via NCHB interactions. This discovery reveals a previously unexplored mechanism for Lewis basic chalcogen‐catalyzed halogenation, distinct from known pathways. The newly developed catalyst enables efficient electrophilic halogenation with low catalyst loadings. Furthermore, this catalytic method proves applicable to the late‐stage bromination of several drug molecules. These findings provide a strong basis for future research in electrophilic halogenation reactions mediated by Lewis basic chalcogens.

## Conflict of Interests

The authors declare no conflict of interest.

## Supporting information



Supporting Information

Supporting Information

## Data Availability

The data that support the findings of this study are available in the Supporting Information of this article.
