# Supplementary material for: Unveiling an Alternative Mechanism for Lewis Basic Selenium as a C─H Hydrogen Bond Catalyst and Its Application in the Halogenation of Arenes
Source: Angew Chem Int Ed Engl. 2025 Aug 10;64(39):e202511770. doi: 10.1002/anie.202511770 (PMC12455386; doi:10.1002/anie.202511770)

Supporting Information

Unveiling an Alternative Mechanism for Lewis Basic Selenium as a C-H Hydrogen Bond Catalyst and Its Application in the Halogenation of Arenes

Jingxian Huang,[a] Qingyu Zhang,[a] Haihui Wang,[a] Junjie Yang,[a] Ying-Lung Steve Tse,[a]* Xiaojian Jiang,[b]* and Ying-Yeung Yeung[a]*

[a] Department of Chemistry and State Key Laboratory of Synthetic Chemistry, The Chinese University of Hong Kong, Shatin, NT, Hong Kong

[b] State Key Laboratory of Bioactive Molecules and Druggability Assessment, Guangdong Basic Research Center of Excellence for Natural Bioactive Molecules and Discovery of Innovative Drugs, College of Pharmacy, Jinan University
Guangzhou, 510632, China

Table of Contents

**S2**	**General information**
**S3-S8**	**Figure S1-S7**
**S9-S10**	**General procedures**
**S11-S30**	**Physical data**
**S31-S36**	**Computational studies**
**S37-S38**	**References**
**S39-S72**	**X-ray crystallographic data**
**S73-S137**	**NMR spectra**

1. General information

Commercially available reagents were used as received. The solvents were dried over a solvent purification system from Innovative Technology. ^{1}H NMR, ^{13}C NMR, and ^{19}F NMR spectra were recorded on a Bruker AMX500 (500 MHz) spectrometer or a Bruker AMX400 (400 MHz) spectrometer. Proton and carbon chemical shifts are reported in parts per million (ppm) values downfield from TMS (δ 0.00) and referenced to residual protons in NMR solvents ($CDCl_3$ at 7.26) or carbon signals in NMR solvent ($CDCl_3$ at 77.0). High-resolution Mass spectra were conducted on a Thermo Finnigan MAT 95 XL spectrometer and Bruker solariX 9.4 Tesla FTICR spectrometer. Analytical thin layer chromatography (TLC) was performed with Merck pre-coated TLC plates, silica gel 60F-254, layer thickness 0.25 mm. Flash chromatography separations were performed on Merck 60 (0.040-0.063 mm) mesh silica gel. Infrared spectra were recorded on a Nicolet 420 FT-IR spectrophotometer and reported in wave numbers (cm-1). XRD patterns were recorded on a RU-300, Rigaku X-ray diffractometer with Cu Kα radiation.

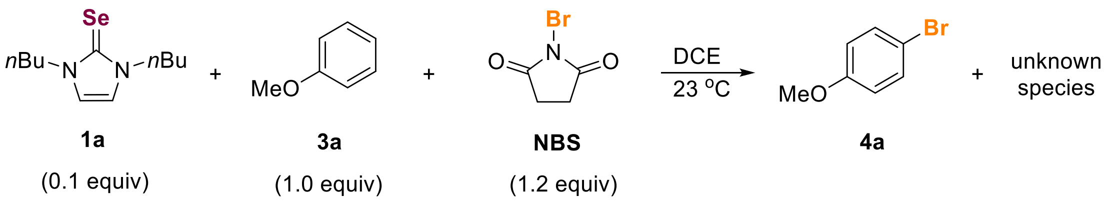

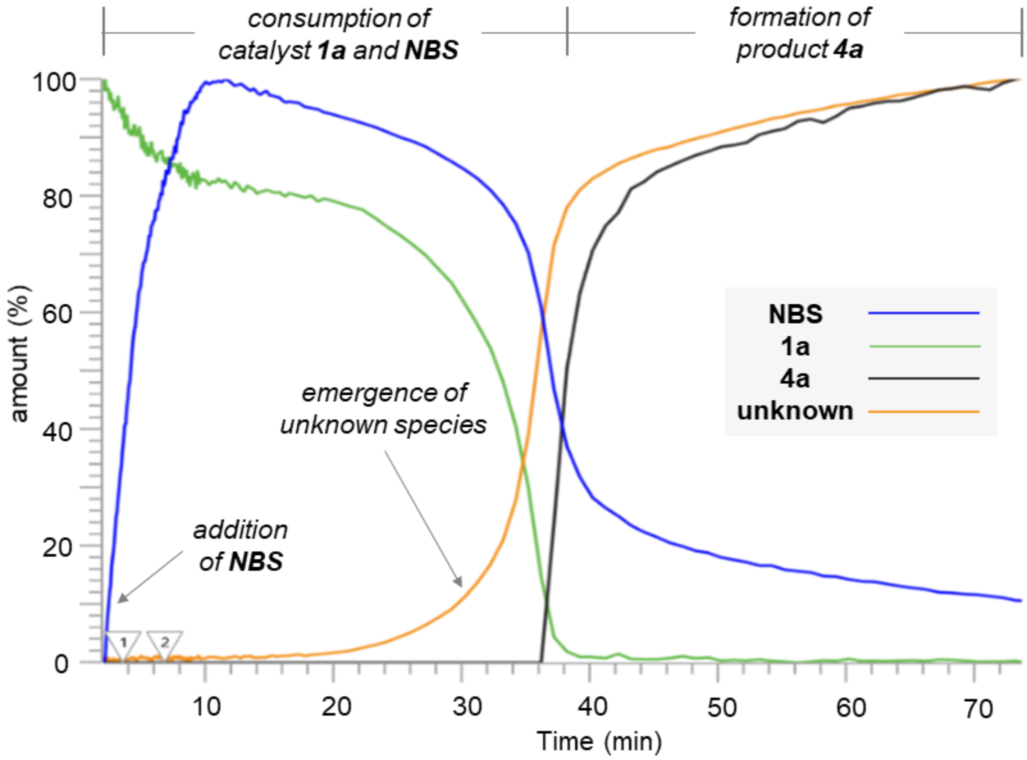

Figure S1. Probing the reaction mixture of selenourea **1a**, NBS and anisole **3a** using in situ IR

Note: to a solution of selenourea ***1a*** *(0.01 mmol) and anisole (****3a****) (0.1 mmol) in DCE (0.5 mL) was added NBS (0.1 mmol) and the reaction was monitored using in situ IR. The selenourea* ***1a*** *was consumed gradually but no brominated product* ***4a*** *was detected. Instead, a new unknown species (later identified to be compound* ***2a****) emerged. Since compound* ***5*** *(the precursor of* ***2a****) and* ***2a*** *have similar structural features and so they should have similar stretching frequency. As a result, compound* ***5*** *should be the major component in the unknown species at the initial stage. NBS was then consumed dramatically and the brominated anisole product* ***4a*** *was formed quickly.*

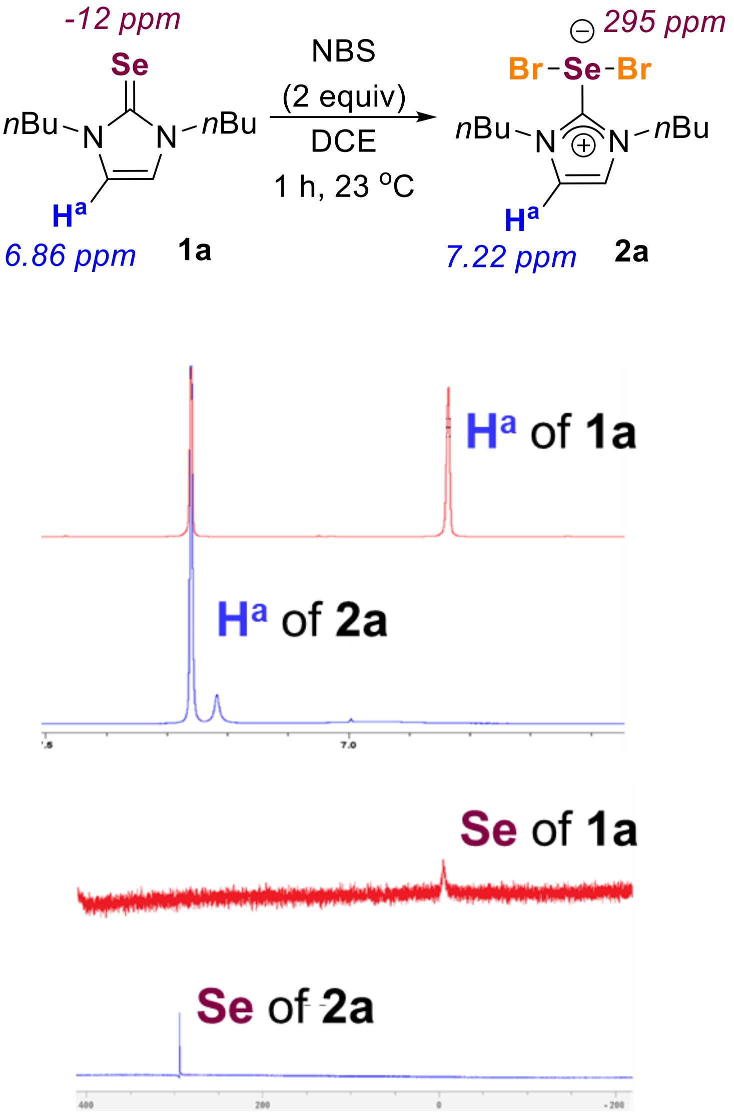

Figure S2. NMR experiments on the reaction with **1a** and NBS

*Note: NBS (0.2 mmol) was added to a solution of **1a** (0.1 mmol) in DCE (0.5 mL). ^{1}H and ^{77}Se NMR experiments were carried out on the sample after the reaction. In the sample of **2a**, the H^a signal of the imidazolium hydrogen exhibited a downfield shift from 6.86 ppm to 7.22 ppm, while the ^{77}Se signal shifted from -12 ppm to 295 ppm.*

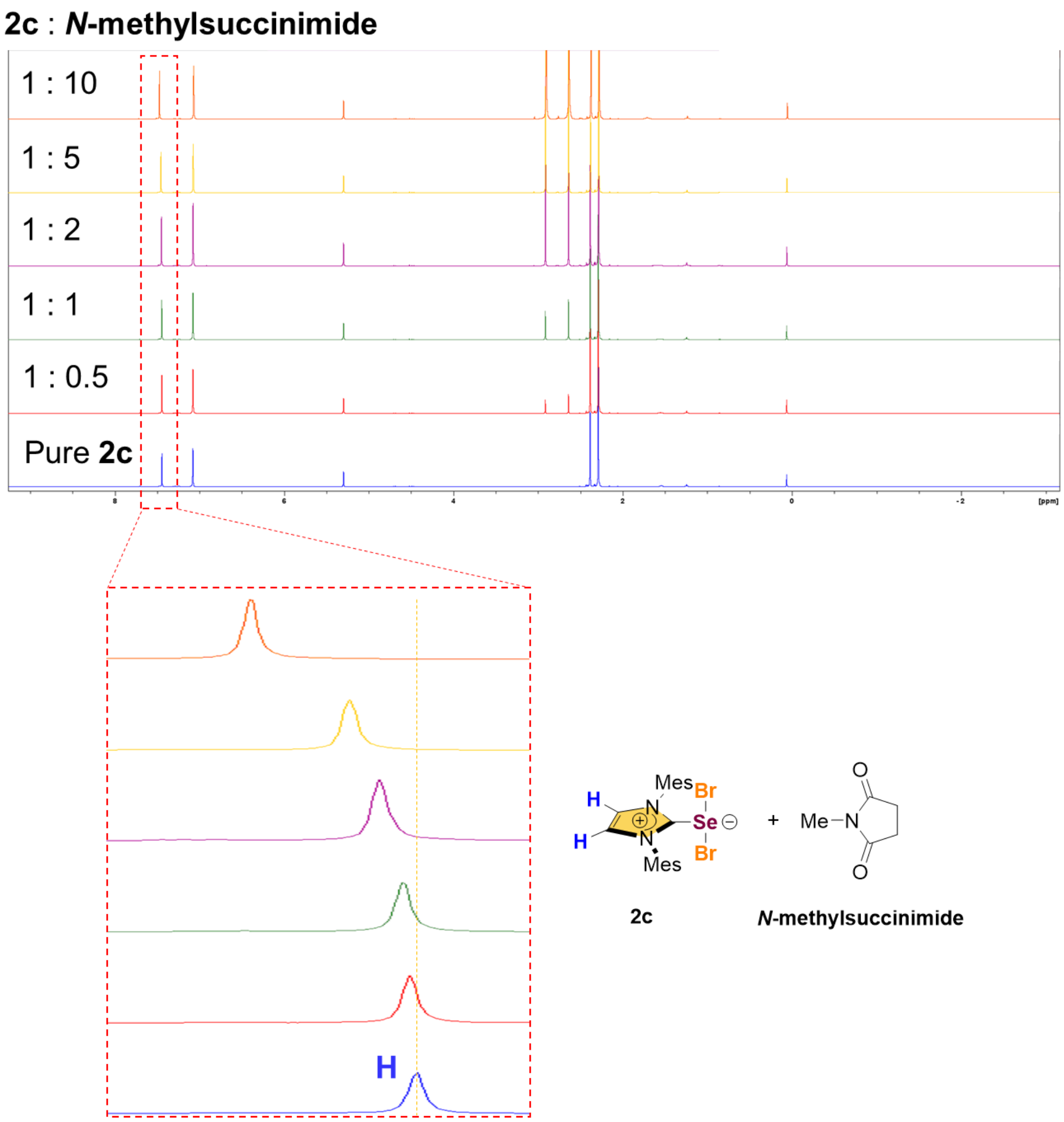

Figure S3. ^{1}H NMR titration experiment on the mixture of **2c** and *N*-methylsuccinimide *Note: different quantities of N-methylsuccinimide (as indicated by the ratios in the NMR spectra) were added to a solution of* ***2c*** *(0.1 mmol) in* CD_2Cl_2 *(0.5 mL).* ^{1}*H NMR analysis revealed that the chemical shift of the imidazolium proton became progressively more pronounced with increasing amounts of N-methylsuccinimide.*

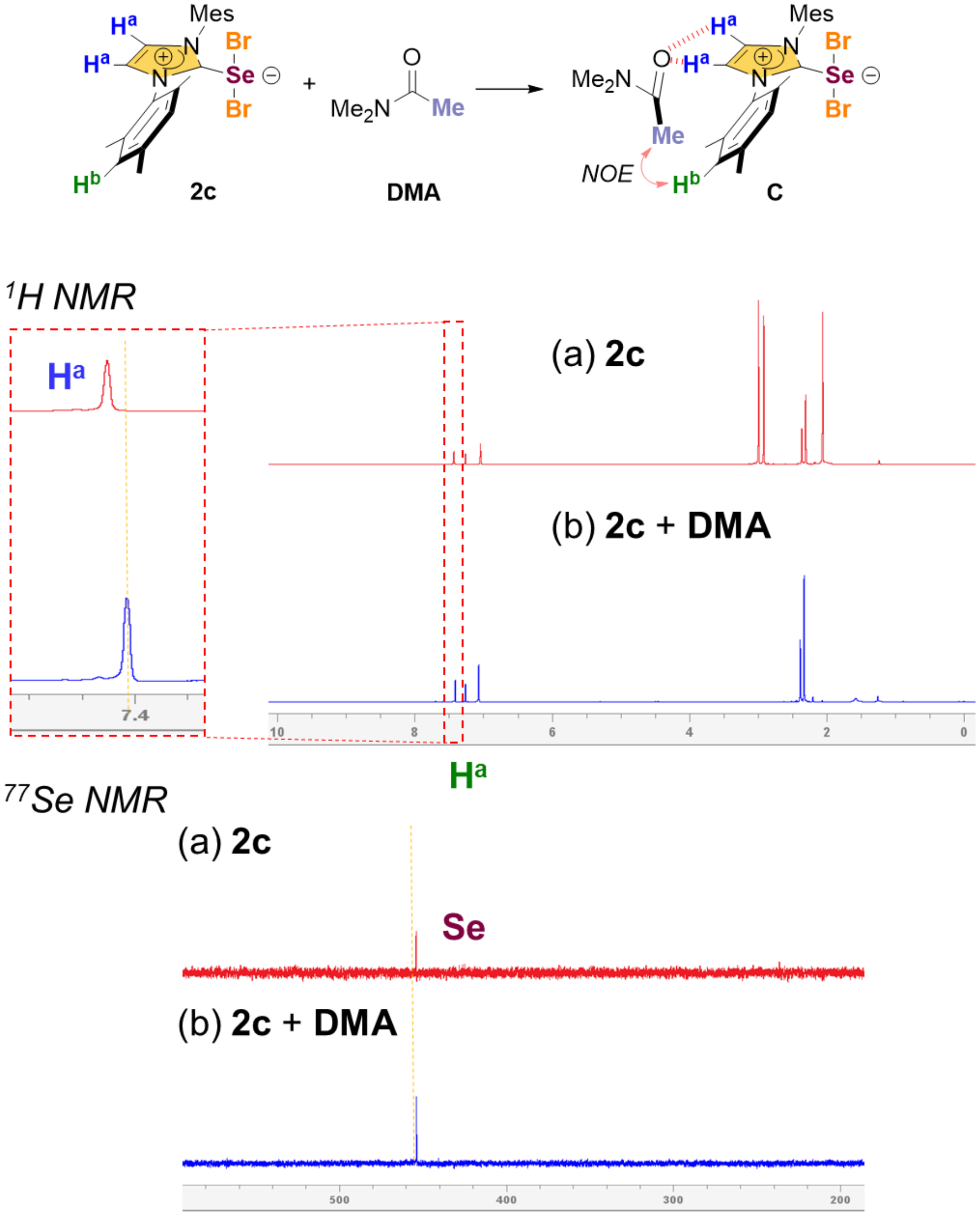

Figure S4. NMR experiments on the mixture of **2c** and DMA

Note: a mixture of ***2c*** *(0.05 mmol) and DMA (0.20 mmol) in $CDCl_3$ was studied using NMR. No significant change of the ^{77}Se signal upon the addition of DMA to* ***2c****. In contrast, considerable chemical shift of the H^a (the imidazolium proton in* ***2c****) was observed for the mixture of* ***2c*** *and DMA, attributed to the formation of species* ***C****.*

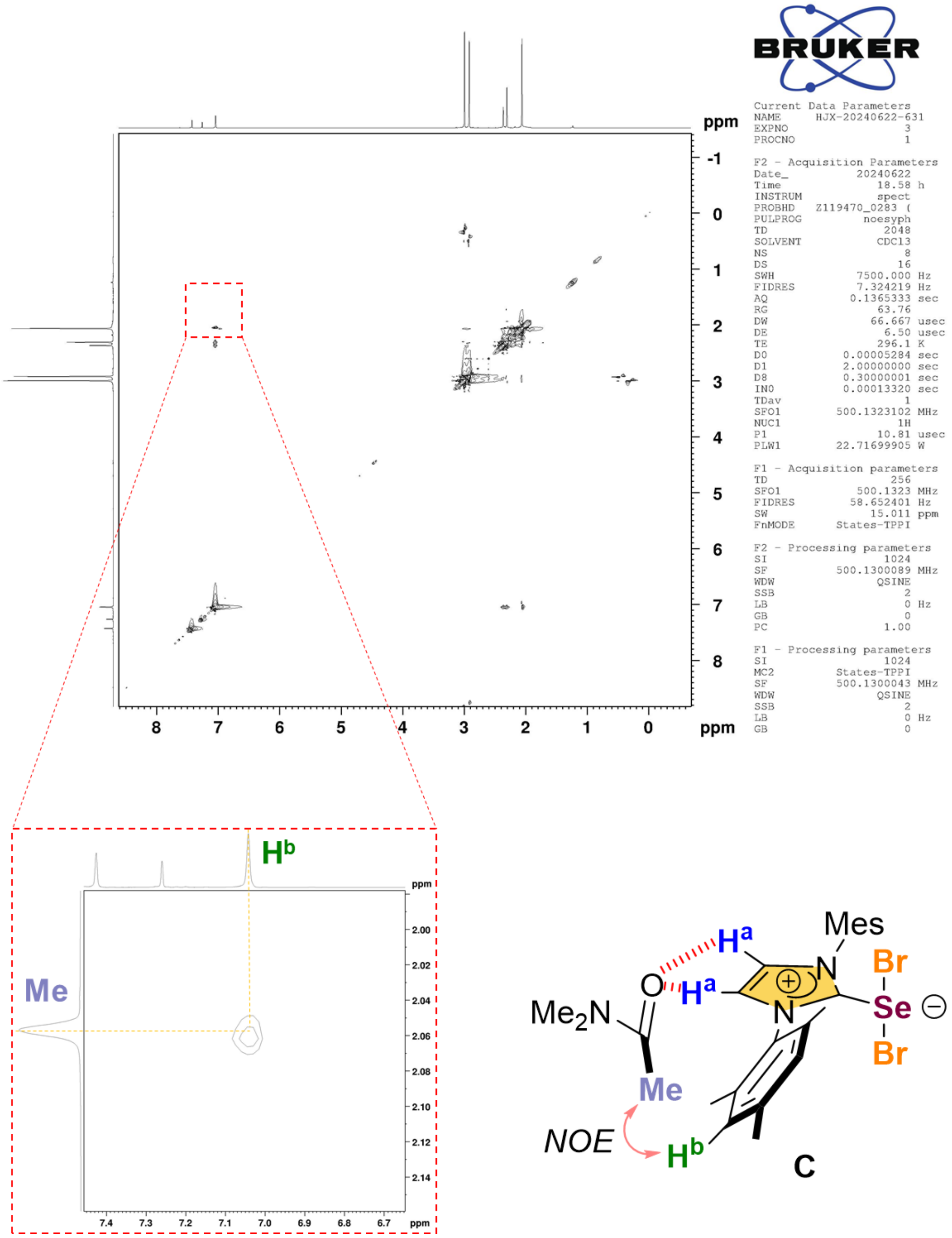

Figure S5. 2D NOESY NMR experiments on the mixture of **2c** and DMA

Note: the sample used in the study shown in Figure S4 was further investigated using 2D NOESY NMR (mixing time = 200 ms). A significant NOE was observed between the Me for DMA and the H^b *of the mesityl in* ***2c****, attributed to the formation of species* ***C****.*

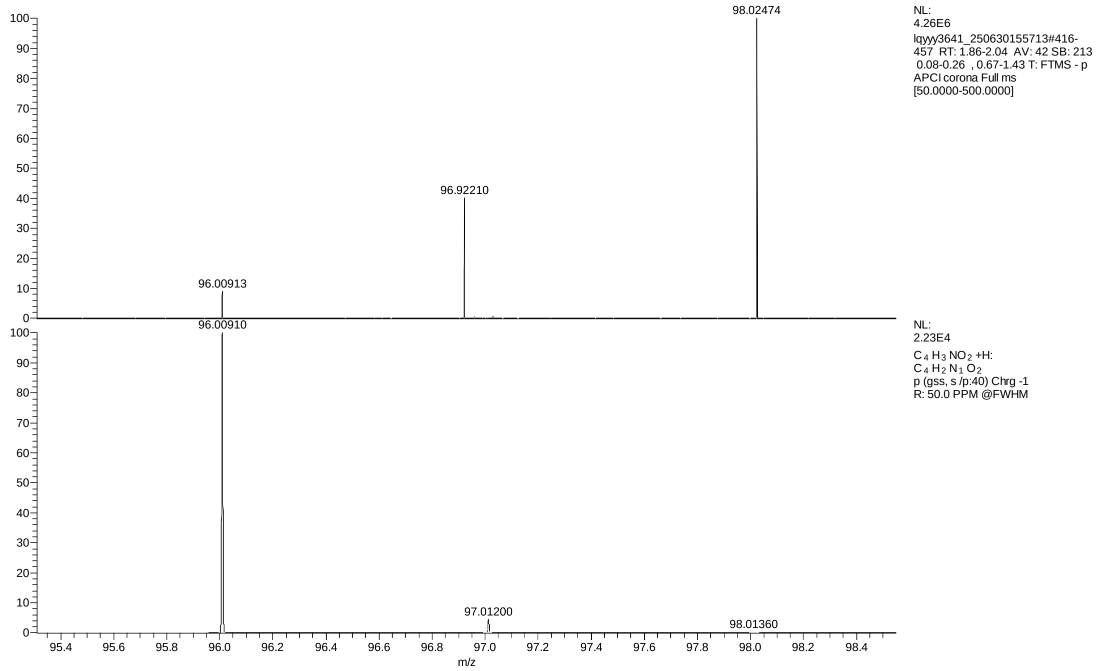

Figure S6. HRMS analysis on a mixture of **1b** and NBS

Note: we analyzed a sample containing a mixture of ***1b*** *and NBS using HRMS, and maleimide was detected ($C_4H_3NO_2$ m/z [M-H]$^-$: 96.0091, found: 96.0092). We believe that NBS might undergo disproportionation, leading to the formation of molecular bromine and maleimide, and the bromine might contribute to the bromide anion in the structure of* ***5****. However, this may represent just one of NBS's potential decomposition pathways. Alternative routes, such as moisture-induced hydrolysis of NBS, could also produce molecular bromine.*

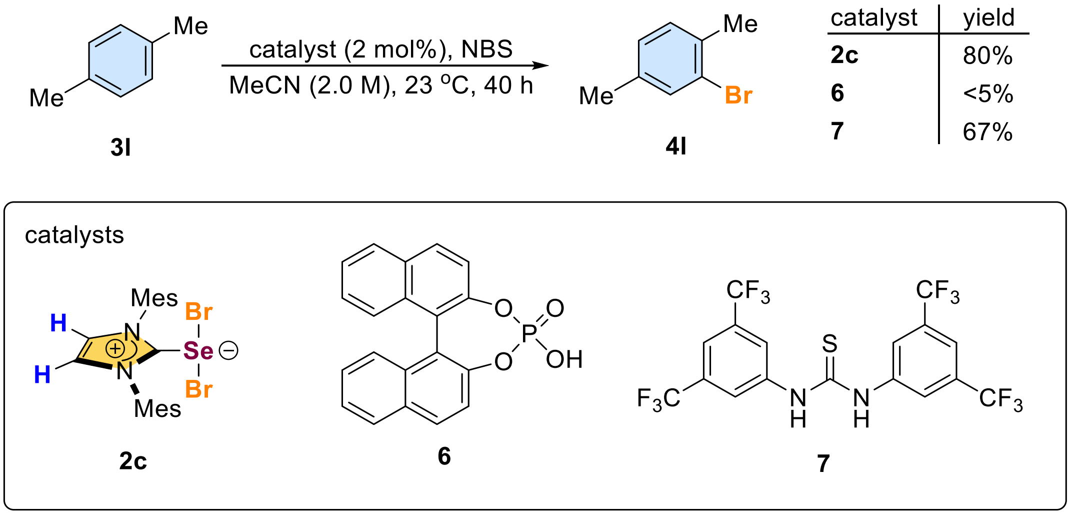

Figure S7. Comparison of catalytic performance with substrate **3l**

2. General procedures

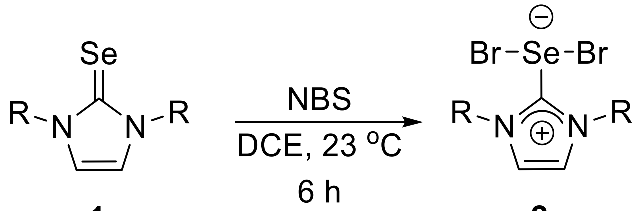

(i) General procedure for the synthesis of catalysts 2.

Selenoureas **1** were prepared according to the literature procedures.[1-2] To a solution of **1** in DCE was added NBS at 23 °C in the absence of light for 6 h. The solvent was removed under reduced pressure and the residue was purified by column chromatography ($CHCl_3$: MeOH = 100: 1) to afford the **2**.

(ii) General procedure for the imidazolium selenium dibromide catalyzed halogenation of arenes

To a solution of catalyst **2c** in 1,2-dichloroethane (0.2 mL) was added arene **3** (0.10 mmol) and NBS (0.11 mmol) at 23 °C in the absence of light. The resultant mixture was stirred at the indicated temperature and the reaction progress was monitored by TLC. Upon completion, the reaction was quenched with saturated aqueous Na_2SO_3 (1 mL). The organic layer was separated, and the aqueous layer was extracted with CH_2Cl_2 (3 × 10 mL). The combined organic layer was dried over anhydrous Na_2SO_4, filtered, and concentrated under reduced pressure. The residue was purified to yield the corresponding brominated product **4**.

(iii) Procedure for the imidazolium selenium dibromide catalyzed halocyclization.

To a solution of 0.01mol% catalyst **2c** in toluene (1.0 mL) was added olefinic substrate **12** (0.1 mmol) and NBS (0.11 mmol) at 23 °C in the absence of light. The resultant mixture was stirred at the indicated temperature and the reaction progress was monitored by TLC. Upon completion, the reaction was quenched with saturated aqueous Na_2SO_3 (3 mL). The organic layer was separated, and the aqueous layer was extracted with dichloromethane (3 × 10 mL). The combined organic layer was dried over anhydrous Na_2SO_4, filtered, and concentrated under reduced pressure. The residue was purified by silica gel column chromatography to afford **13**.

(iv) Procedure for the imidazolium selenium dibromide catalyzed Hantzsch ester reduction.

To a solution of 5 mol% catalyst **2c** in $CDCl_3$ (1.0 mL) was added quinoline substrate

14 (0.1 mmol) and Hantzsch Esters (0.15 mmol) at 23 °C. The resultant mixture was stirred at the indicated temperature and the reaction progress was monitored by TLC. Upon completion, the organic layer was directly purified by silica gel column chromatography with 10% Et_2O in cyclohexane to yield **15**.

(v) Procedure for the imidazolium selenium dibromide catalyzed Mannich reaction.

To a mixture of acetophenone (**16**) (1.0 mmol, 1.0 equiv.), benzaldehyde (**17**) (1.0 mmol, 1.0 equiv.) and aniline (**18**) (1.0 mmol, 1.0 equiv.) was added to catalyst **2c** (1 mol %) at 23 °C. The resultant mixture was stirred at 23 °C for 24 h. The reaction mixture was concentrated under reduced pressure. The residue was purified by flash column chromatography on silica gel (10% Et_2O/hexanes) to provide **19**.

3. Physical data

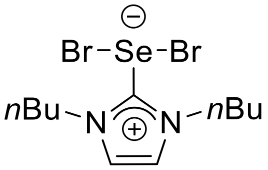

2a, **1,3-dibutyl-2-(dibromo-λ4-selanylidene)imidazole**

Orange solid, 60% yield, ^{1}H NMR (400 MHz, Chloroform-*d*) δ 7.23 (s, 2H), 4.43 – 4.35 (m, 4H), 2.09 – 1.97 (m, 4H), 1.46 (dt, *J* = 14.8, 7.4 Hz, 4H), 1.02 (t, *J* = 7.4 Hz, 6H); ^{13}C NMR (101 MHz, Chloroform-d) δ 141.83, 122.04, 51.61, 31.13, 19.87, 13.61.; ^{77}Se NMR (76 MHz, Chloroform-d) δ 294.41. HRMS (ESI) calcd for $C_{11}H_{20}Br_2N_2Se$ m/z [M+CH_3OH-H]$^-$: 450.93277, found: 450.93345.

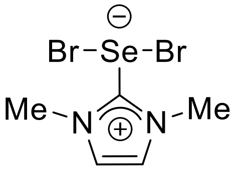

2b, **2-(dibromo-λ4-selanylidene)-1,3-dimethylimidazole**

Orange solid, 82% yield, ^{1}H NMR (500 MHz, Chloroform-*d*) δ 7.22 (s, 2H), 4.07 (s, 6H); ^{13}C NMR (126 MHz, Chloroform-*d*) δ 122.99, 38.53; ^{77}Se NMR (95 MHz, Chloroform-*d*) δ 282.70. HRMS (ESI) calcd for $C_5H_8Br_2N_2Se$ m/z [M+CH_3OH-H]$^-$: 366.83881, found: 366.83939.

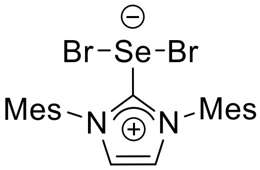

2c, **2-(dibromo-λ4-selanylidene)-1-(1,3,5-trimethylbenzen-2-yl)-3-(2,4,6-trimethylphenyl)imidazole**

Orange solid, 73% yield, ^{1}H NMR (400 MHz, Chloroform-d) δ 7.41 (s, 2H), 7.07 (s, 4H), 2.38 (s, 6H), 2.33 (s, 12 H); ^{13}C NMR (101 MHz, Chloroform-d) δ 141.07, 135.38, 132.17, 129.87, 125.44, 21.24, 20.05; ^{77}Se NMR (95 MHz, Chloroform-*d*) δ 453.51. APCI calcd for $C_{21}H_{24}N_2SeBr_2$ m/z [M-Br]$^+$: 463.02807, found: 463.02826.

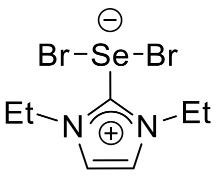

2d, **2-(dibromo-λ4-selanylidene)-1,3-diethylimidazole**

Orange solid, 64% yield, ^{1}H NMR (400 MHz, Chloroform-d) δ 7.28 (s, 2H), 4.49 (q, J = 7.4 Hz, 4H), 1.66 (t, J = 7.4 Hz, 6H); ^{13}C NMR (101 MHz, Chloroform-d) δ 141.69, 121.65, 46.96, 14.77; ^{77}Se NMR (76 MHz, Chloroform-d) δ 287.79. HRMS (ESI) calcd for $C_7H_{12}Br_2N_2Se$ m/z [M-Br]$^{+}$: 282.93401, found: 282.93375.

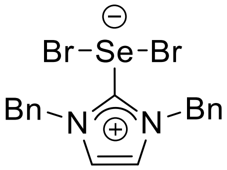

2e, **1,3-dibenzyl-2-(dibromo-λ4-selanylidene)imidazole**

Orange solid, 72% yield, ^{1}H NMR (400 MHz, Chloroform-d) δ 7.54 – 7.49 (m, 4H), 7.46 – 7.40 (m, 6H), 6.84 (s, 2H), 5.61 (s, 4H); ^{13}C NMR (101 MHz, Chloroform-d) δ 132.30, 129.80, 129.62, 129.53, 121.70, 55.23; ^{77}Se NMR (76 MHz, Chloroform-d) δ 280.09. APCI calcd for $C_{17}H_{16}N_2SeBr_2$ m/z [M-Br]$^{+}$: 406.96539, found: 406.96510.

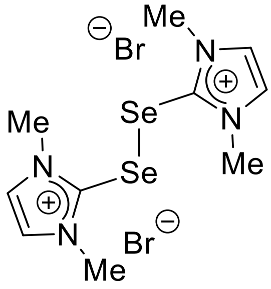

5, **2-[(1,3-dimethylimidazol-1-ium-2-yl)diselanyl]-1,3-dimethylimidazol-1-ium dibromide**

Yellow solid, 42% yield, ^{1}H NMR (500 MHz, Methanol-d4) δ 7.76 (s, 4H), 3.75 (s, 12H). ^{13}C NMR (126 MHz, DMSO-d6) δ 124.71, 37.50. HRMS (ESI) calcd for $C_{10}H_{16}Br_2N_4Se_2$ m/z [M-Br]$^{+}$: 430.88802, found: 430.88773

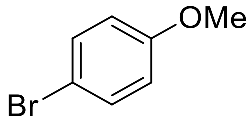

4a, 4-bromo-1-methoxybenzene

The title compound was purified by column chromatology using 5% Et_2O in cyclohexane as a colorless oil in 99% yield (**3a**: 0.1 mmol, **2c**: 0.005 mg, yield: 184.2 mg/mmol). ^{1}H NMR (500 MHz, Chloroform-d) δ 7.38 (d, J = 9.0 Hz, 2H), 6.78 (d, J = 8.9 Hz, 2H), 3.78 (s, 3H).; ^{13}C NMR (126 MHz, Chloroform-*d*) δ 158.69, 132.25, 115.73, 112.82, 55.45. QEFMS (APCI) calcd for C_7H_7BrO m/z $[M]^+$: 185.96748, found: 185.96721. The spectroscopic data is in accordance with the literature report. [3]

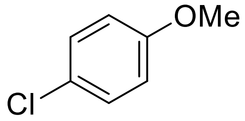

4a-Cl, 4-chloro-1-methoxybenzene

The title compound was purified by column chromatology using 5% Et_2O in cyclohexane as a colorless oil in 86% yield (**3a**: 0.1 mmol, **2c**: 0.005 mg, yield: 123.0 mg/mmol). ^{1}H NMR (400 MHz, Chloroform-d) δ 7.25 – 7.21 (m, 2H), 6.83 (d, J = 9.0 Hz, 2H), 3.79 (s, 3H); ^{13}C NMR (101 MHz, Chloroform-d) δ 158.21, 129.31, 125.53, 115.19, 55.48. QEFMS (APCI) calcd for C_7H_7ClO m/z $[M+H]^+$: 143.02582, found: 143.02565. The spectroscopic data is in accordance with the literature report.[4]

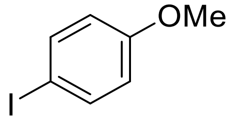

4a-I, 4-iodo-1-methoxybenzene

The title compound was purified by column chromatology using 5% Et_2O in cyclohexane as a colorless oil in 89% yield (**3a**: 0.1 mmol, **2c**: 0.005 mg, yield: 208.3 mg/mmol). ^{1}H NMR (500 MHz, Chloroform-d) δ 7.56 (d, J = 7.9 Hz, 2H), 6.68 (d, J = 7.9 Hz, 2H), 3.78 (s, 3H); ^{13}C NMR (126 MHz, Chloroform-d) δ 159.57, 138.31, 116.47, 82.81, 55.44. HRMS (EI) calcd for C_7H_7IO m/z $[M]^+$: 233.9536, found: 233.9541. The spectroscopic data is in accordance with the literature report. [5]

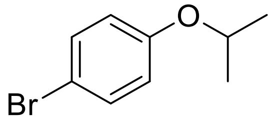

4b, 4-bromo-1-(prop-2-yloxy)benzene

The title compound was purified by column chromatology using 5% Et_2O in cyclohexane as a colourless oil in 99% yield (**3b**: 0.1 mmol, **2c**: 0.005 mg, yield: 211.9 mg/mmol). ^{1}H NMR (500 MHz, Chloroform-d) δ 7.35 (d, J = 8.9 Hz, 2H), 6.76 (d, J =

8.9 Hz, 2H), 4.49 (p, J = 6.1 Hz, 1H), 1.33 (s, 6H); ^{13}C NMR (126 MHz, Chloroform-d) δ 156.99, 132.27, 117.69, 112.54, 70.25, 21.94.; HRMS (EI) calcd for $C_9H_{11}BrO$ m/z $[M]^+$: 213.9988, found: 213.9988. The spectroscopic data is in accordance with the literature.[6]

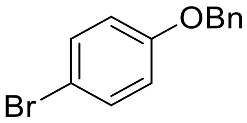

4c, 4-(benzyloxy)-1-bromobenzene

The title compound was purified by column chromatology using 5% Et_2O in cyclohexane as a white solid in 99% yield (**3c**: 0.1 mmol, **2c**: 0.005 mg, yield: 260.4 mg/mmol). ^{1}H NMR (500 MHz, Chloroform-d) δ 7.46 – 7.30 (m, 7H), 6.88 – 6.84 (m, 2H), 5.04 (s, 2H). ^{13}C NMR (126 MHz, Chloroform-d) δ 157.87, 136.56, 132.32, 128.68, 128.15, 127.48, 116.71, 113.14, 70.23.; QEFMS (APCI) calcd for $C_{13}H_{11}BrO$ m/z $[M-H]^-$: 260.99205, found: 260.99207. The spectroscopic data is in accordance with the literature.[6]

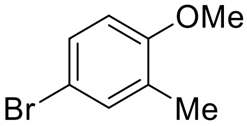

4d, 4-bromo-1-methoxy-2-methylbenzene

The title compound was purified by column chromatology using 5% Et_2O in cyclohexane as a colorless oil in 97% yield (**3d**: 0.1 mmol, **2c**: 0.005 mg, yield: 194.2 mg/mmol). ^{1}H NMR (500 MHz, Chloroform-d) δ 7.26 (d, J = 10.1 Hz, 2H), 6.68 (d, J = 8.3 Hz, 1H), 3.80 (s, 3H), 2.19 (s, 3H). ^{13}C NMR (126 MHz, Chloroform-d) δ 156.87, 133.17, 129.35, 128.99, 112.34, 111.48, 55.49, 16.07.; APCI calcd for C_8H_9BrO m/z $[M]^+$: 199.98313, found: 199.98311. The spectroscopic data is in accordance with the literature.[6]

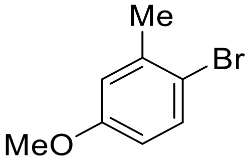

4e, 1-bromo-4-methoxy-2-methylbenzene

The title compound was purified by column chromatology using 5% Et_2O in cyclohexane as a colorless oil in 99% yield (**3e**: 0.1 mmol, **2c**: 0.005 mg, yield: 199.0 mg/mmol). ^{1}H NMR (500 MHz, Chloroform-d) δ 7.40 (d, J = 8.7 Hz, 1H), 6.79 (d, J = 3.1 Hz, 1H), 6.62 (dd, J = 8.7, 3.1 Hz, 1H), 3.77 (s, 3H), 2.37 (s, 3H). ^{13}C NMR (126 MHz, Chloroform-d) δ 158.80, 138.82, 132.81, 116.50, 115.42, 112.93, 55.41, 23.18.

QEFMS (APCI) calcd for C_8H_9BrO m/z $[M]^+$: 199.98313, found: 199.98304. The spectroscopic data is in accordance with the literature.[6]

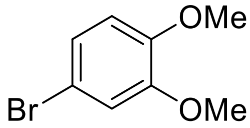

4f, 4-bromo-1,2-dimethoxybenzene

The title compound was purified by column chromatology using 10% Et_2O in cyclohexane as a colorless oil in 97% yield (**3f**: 0.1 mmol, **2c**: 0.005 mg, yield: 210.5 mg/mmol). ^1H NMR (500 MHz, Chloroform-d) δ 7.02 (dd, J = 8.5, 2.3 Hz, 1H), 6.97 (d, J = 2.3 Hz, 1H), 6.73 (d, J = 8.6 Hz, 1H), 3.85 (d, J = 5.5 Hz, 6H). ^{13}C NMR (126 MHz, Chloroform-d) δ 149.72, 148.31, 123.37, 114.74, 112.71, 112.44, 56.09, 56.03. HRMS (ESI) calcd for $C_8H_9BrO_2$ m/z $[M+Na]^+$: 238.96781, found: 238.96782. The spectroscopic data is in accordance with the literature.[6]

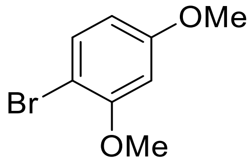

4g, 1-bromo-2,4-dimethoxybenzene

The title compound was purified by column chromatology using 10% Et_2O in cyclohexane as a colorless oil in 99% yield (**3g**: 0.1 mmol, **2c**: 0.005 mg, yield: 214.8 mg/mmol). ^1H NMR (500 MHz, Chloroform-d) δ 7.40 (d, J = 8.7 Hz, 1H), 6.48 (d, J = 2.7 Hz, 1H), 6.40 (dd, J = 8.7, 2.7 Hz, 1H), 3.87 (s, 3H), 3.79 (s, 3H); ^{13}C NMR (126 MHz, Chloroform-d) δ 160.24, 156.54, 133.16, 105.88, 102.43, 99.97, 56.15, 55.59. QEFMS (APCI) calcd for $C_8H_9BrO_2$ m/z $[M+H]^+$: 216.98587, found: 216.98580. The spectroscopic data is from the literature.[6]

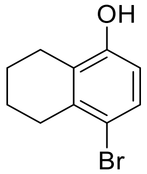

4h, 4-bromo-5,6,7,8-tetrahydronaphthalen-1-ol

The title compound was purified by column chromatology using 10% Et_2O in cyclohexane as a white solid in 98% yield (**3h**: 0.1 mmol, **2c**: 0.005 mg, yield: 222.5 mg/mmol). 1H NMR (500 MHz, Chloroform-d) δ 7.25 (d, J = 8.5 Hz, 1H), 6.53 (d, J = 8.5 Hz, 1H), 4.84 (s, 1H), 2.83 – 2.67 (m, 2H), 2.67 – 2.53 (m, 2H), 1.79 (p, J = 3.3 Hz, 4H); ^{13}C NMR (126 MHz, Chloroform-d) δ 152.62, 137.73, 129.72, 126.05, 116.36, 113.40, 30.60, 23.48, 22.77, 22.08. QEFMS (ESI) calcd for $C_{10}H_{11}BrO$ m/z $[M-H]^-$:

224.99205, found: 224.99226. The spectroscopic data is in accordance with the literature.[7]

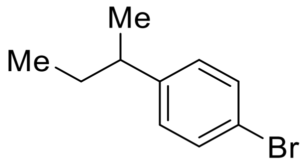

4i, 4-bromo-1-(but-2-yl)benzene

The residue was purified by thin-layer chromatography as a colorless oil with a 68% yield (**3i**: 0.1 mmol, **2c**: 5.4 mg, yield: 144.8 mg/mmol). ^{1}H NMR (400 MHz, Chloroform-d) δ 7.40 (d, J = 8.4 Hz, 2H), 7.05 (d, J = 8.4 Hz, 22H), 2.56 (h, J = 7.0 Hz, 1H), 1.56 (dtd, J = 14.8, 7.4, 2.4 Hz, 2H), 1.21 (d, J = 6.9 Hz, 3H), 0.80 (t, J = 7.4 Hz, 3H); ^{13}C NMR (101 MHz, Chloroform-d) δ 146.62, 131.29, 128.86, 119.33, 41.22, 31.05, 21.77, 12.15. HRMS (EI) calcd for $C_{10}H_{13}Br$ m/z $[M]^+$: 212.0195, found: 211.9918. The spectroscopic data is in accordance with the literature.[11]

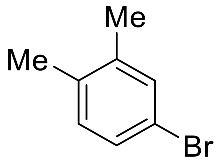

4j, 4-bromo-1,2-dimethylbenzene

The residue was purified by thin layer chromatography as a colorless oil in 74% yield (**3j**: 0.1 mmol, **2c**: 5.4 mg, yield: 136.9 mg/mmol), and its ratio was then determined by ^{1}H NMR as *para*:*ortho* = 7:1. ^{1}H NMR (400 MHz, Chloroform-d) δ 7.39 (d, J = 8.0 Hz, 2.5H), 7.27 (d, J = 1.8 Hz, 18H), 7.21 (dd, J = 8.0, 2.1 Hz, 18H), 7.08 (d, J = 7.5 Hz, 2.5H), 6.99 (d, J = 8.0 Hz, 18H), 6.93 (t, J = 7.7 Hz, 2.5H), 5.30 (s, 1H), 2.36 (s, 7.5H), 2.33 (s, 7.5H), 2.23 (s, 54H), 2.20 (s, 54H); ^{13}C NMR (101 MHz, Chloroform-d) δ 138.80, 135.45, 132.28, 131.15, 128.68, 119.16, 19.63, 19.28. HRMS (EI) calcd for C_8H_9Br m/z $[M]^+$: 183.9882, found: 183.9878. The spectroscopic data is in accordance with the literature.[5]

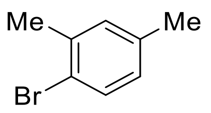

4k, 1-bromo-2,4-dimethylbenzene

The residue was purified by thin layer chromatography as a colorless oil in 97% yield (**3k**: 0.1 mmol, **2c**: 2.7 mg, yield: 179.4 mg/mmol); ^{1}H NMR (500 MHz, Chloroform-d) δ 7.39 (d, J = 8.1 Hz, 1H), 7.05 (s, 1H), 6.85 (d, J = 8.1 Hz, 1H), 2.35 (s, 3H), 2.27 (s, 3H); ^{13}C NMR (126 MHz, Chloroform-d) δ 137.46, 137.06, 132.02, 131.68, 128.11,

121.50, 22.79, 20.83. HRMS (EI) calcd for C_8H_9Br m/z $[M]^+$: 183.9882, found: 183.9878. The spectroscopic data is in accordance with the literature.[5]

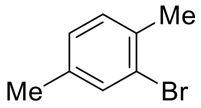

4l, 2-bromo-1,4-dimethylbenzene

The residue was purified by thin-layer chromatography as a colorless oil with 80% yield (**3l**: 0.1 mmol, **2c**: 1.1 mg, yield: 147.2 mg/mmol). ^{1}H NMR (400 MHz, Chloroform-d) δ 7.36 (s, 1H), 7.10 (d, J = 7.7 Hz, 1H), 7.00 (d, J = 8.4 Hz, 1H), 2.35 (s, 3H), 2.29 (s, 3H); ^{13}C NMR (101 MHz, Chloroform-d) δ 137.22, 134.59, 132.76, 130.51, 128.02, 124.64, 22.37, 20.56. HRMS (EI) calcd for C_8H_9Br m/z $[M]^+$: 183.9882, found: 183.9881. The spectroscopic data is in accordance with the literature.[5]

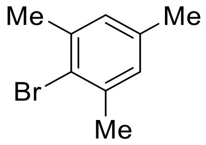

4m, 2-bromo-1,3,5-trimethylbenzene

The residue was purified by thin-layer chromatography as a colorless oil with 98% yield (**3m**: 0.1 mmol, **2c**: 0.05 mg, yield: 195.0 mg/mmol). ^{1}H NMR (500 MHz, Chloroform-d) δ 6.89 (s, 2H), 2.38 (s, 6H), 2.24 (s, 3H). ^{13}C NMR (126 MHz, Chloroform-d) δ 137.91, 136.30, 129.04, 124.20, 23.73, 20.68. QEFMS (APCI) calcd for $C_9H_{11}Br$ m/z $[M+H]^+$: 199.01169, found: 199.01175. The spectroscopic data is from the literature.[5]

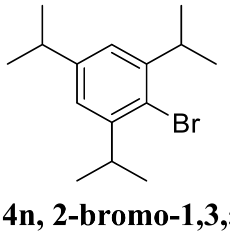

4n, 2-bromo-1,3,5-tri(prop-2-yl)benzene

The residue was purified by column chromatology using 2% Et_2O in cyclohexane as a colorless oil with a 93% yield (**3n**: 0.1 mmol, **2c**: 1.1 mg, yield: 263.2 mg/mmol). ^{1}H NMR (500 MHz, Chloroform-d) δ 7.00 (s, 2H), 3.50 (p, J = 6.8 Hz, 2H), 2.88 (p, J = 6.9 Hz, 1H), 1.26 (m, 18H); ^{13}C NMR (126 MHz, Chloroform-d) δ 147.83, 147.39, 123.61, 122.31, 34.07, 33.57, 24.06, 23.13. HRMS (EI) calcd for $C_{15}H_{23}Br$ m/z $[M]^+$: 282.0968, found: 282.0963. The spectroscopic data is in accordance with the literature.[10]

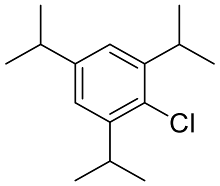

4n-Cl, 2-chloro-1,3,5-tri(prop-2-yl)benzene

The residue was purified by column chromatology using 2% Et_2O in cyclohexane as a colorless oil with a 99% yield (**3n**: 0.1 mmol, **2c**: 1.1 mg, yield: 236.1 mg/mmol). ^1H NMR (500 MHz, Chloroform-d) δ 7.01 (s, 2H), 3.49 (hept, J = 6.9 Hz, 2H), 2.89 (p, J = 6.9 Hz, 1H), 1.26 (dd, J = 6.9, 1.7 Hz, 18H); ^{13}C NMR (126 MHz, Chloroform-d) δ 147.09, 145.58, 130.06, 121.97, 34.12, 30.65, 24.11, 22.91. QEFMS (APCI) calcd for $C_{15}H_{23}Cl$ m/z $[M-Cl]^+$: 203.17943, found: 203.17910.

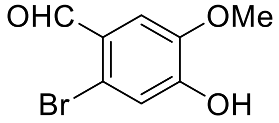

4o, 2-bromo-4-hydroxy-5-methoxybenzene-1-carbaldehyde

The residue was purified by thin-layer chromatography as a white solid with 88% yield (**3o**: 0.1 mmol, **2c**: 2.7 mg, yield: 204.2 mg/mmol). ^1H NMR (500 MHz, Chloroform-d) δ 9.79 (s, 1H), 7.64 (s, 1H), 7.37 (s, 1H), 6.49 (s, 1H), 3.99 (s, 3H). ^{13}C NMR (126 MHz, Chloroform-d) δ 189.71, 148.86, 147.66, 130.14, 108.16, 108.01, 107.92, 56.64. QEFMS (APCI) calcd for $C_8H_7BrO_3$ m/z $[M+H]^+$: 230.96513, found: 230.96506. The spectroscopic data is from the literature.[7]

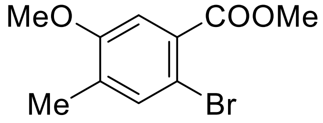

4p, methyl 2-bromo-5-methoxy-4-methylbenzoate

The residue was purified by column chromatology using 10% Et_2O in cyclohexane as a white solid with 80% yield (**3p**: 0.1 mmol, **2c**: 2.7 mg, yield: 207.2 mg/mmol). ^1H NMR (500 MHz, Chloroform-d) δ 7.40 (s, 1H), 7.27 (s, 1H), 3.92 (s, 3H), 3.84 (s, 3H), 2.21 (s, 3H); ^{13}C NMR (126 MHz, Chloroform-d) δ 166.49, 156.65, 135.96, 132.84, 129.44, 112.59, 112.24, 55.63, 52.36, 16.02. QEFMS (ESI) calcd for $C_{10}H_{11}BrO_3$ m/z $[M+Na]^+$: 280.97838, found: 280.97830.

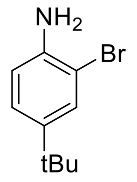

4q, 2-bromo-4-(2-methylprop-2-yl)aniline

The residue was purified by column chromatology using 10% Et_2O in cyclohexane as a white solid with 94% yield (**3q**: 0.1 mmol, **2c**: 0.03 mg, yield: 214.3 mg/mmol). ^1H NMR (500 MHz, Chloroform-d) δ 7.42 (d, J = 2.2 Hz, 1H), 7.14 (dd, J = 8.3, 2.2 Hz, 1H), 6.77 (d, J = 8.3 Hz, 1H), 3.95 (s, 2H), 1.27 (s, 9H); ^{13}C NMR (126 MHz, Chloroform-d) δ 143.46, 140.77, 129.42, 125.45, 116.05, 109.74, 34.07, 31.40. QEFMS (ESI) calcd for $C_{10}H_{14}BrN$ m/z $[M+H]^+$: 228.03824, found: 228.03841.

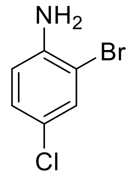

4r, 2-bromo-4-chloroaniline

The residue was purified by column chromatology using 10% Et_2O in cyclohexane as a white solid with 98% yield (**3r**: 0.1 mmol, **2c**: 0.03 mg, yield: 202.4 mg/mmol). ^1H NMR (500 MHz, Chloroform-d) δ 7.40 (d, J = 2.4 Hz, 1H), 7.07 (dd, J = 8.5, 2.4 Hz, 1H), 6.68 (d, J = 8.6 Hz, 1H), 4.03 (s, 2H); ^{13}C NMR (126 MHz, Chloroform-d) δ 142.85, 131.84, 128.36, 123.01, 116.22, 109.16. QEFMS (ESI) calcd for C_6H_5BrClN m/z $[M+H]^+$: 205.91986, found: 205.91979. The spectroscopic data is from the literature.[9]

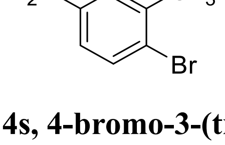

4s, 4-bromo-3-(trifluoromethyl)aniline

The residue was purified by column chromatology using 10% Et_2O in cyclohexane as a white solid with 77% yield (**3s**: 0.1 mmol, **2c**: 0.05 mg, yield: 184.8 mg/mmol). ^1H NMR (500 MHz, Chloroform-d) δ 7.41 (d, J = 8.5 Hz, 1H), 6.98 (d, J = 2.9 Hz, 1H), 6.66 (dd, J = 8.6, 2.9 Hz, 1H), 3.85 (s, 2H); ^{13}C NMR (126 MHz, Chloroform-d) δ 145.66, 135.47, 130.53 (q, J = 30.9 Hz), 126.15, 123.97, 121.80, 119.62, 118.88, 114.11 (d, J = 5.5 Hz), 106.71 (d, J = 2.0 Hz); ^{19}F NMR (471 MHz, Chloroform-d) δ -62.87. QEFMS (ESI) calcd for $C_7H_5BrF_3N$ m/z $[M-H]^-$: 237.94847, found: 237.94854. The spectroscopic data is from the literature.[8]

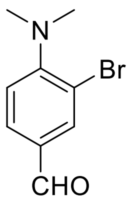

4t, 3-bromo-4-(dimethylamino)benzene-1-carbaldehyde

The residue was purified by column chromatology using 10% Et_2O in cyclohexane as a white solid with 95% yield (**3t**: 0.1 mmol, **2c**: 0.03 mg, yield: 217.6 mg/mmol). ^1H NMR (500 MHz, Chloroform-d) δ 9.80 (s, 1H), 8.02 (d, J = 1.9 Hz, 1H), 7.72 (dd, J = 8.4, 2.0 Hz, 1H), 7.07 (d, J = 8.3 Hz, 1H), 2.94 (s, 6H); ^{13}C NMR (126 MHz, Chloroform-d) δ 189.70, 156.81, 136.00, 131.05, 129.93, 119.48, 116.59, 43.50. QEFMS (ESI) calcd for $C_9H_{10}BrNO$ m/z $[M+H]^+$: 228.00185, found: 228.00195. The spectroscopic data is from the literature.[7]

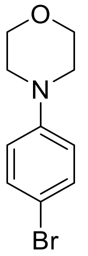

4u, 4-(4-bromophenyl)-1,4-oxazinane

The residue was purified by column chromatology using 10% Et_2O in cyclohexane as a white solid with 96% yield (**3u**: 0.1 mmol, **2c**: 0.03 mg, yield: 232.3 mg/mmol). ^1H NMR (500 MHz, Chloroform-d) δ 7.35 (d, J = 8.6 Hz, 2H), 6.77 (d, J = 8.6 Hz, 2H), 3.85 (t, J = 4.8 Hz, 3H), 3.11 (t, J = 4.8 Hz, 3H); ^{13}C NMR (126 MHz, Chloroform-d) δ 150.28, 131.96, 117.33, 112.22, 66.76, 49.17. HRMS (ESI) calcd for $C_{10}H_{12}BrNO$ m/z $[M+H]^+$: 242.01750, found: 242.01755. The spectroscopic data is in accordance with the literature.[6]

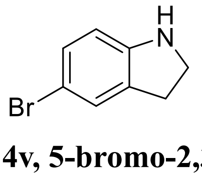

4v, 5-bromo-2,3-dihydro-1H-indole

The residue was purified by column chromatology using 10% Et_2O in cyclohexane as yellow oil with 52% yield (**3v**: 0.1 mmol, **2c**: 0.05 mg, yield: 103.0 mg/mmol). ^1H NMR (500 MHz, Chloroform-d) δ 7.19 (s, 1H), 7.09 (d, J = 8.2 Hz, 1H), 6.50 (d, J = 8.2 Hz, 1H), 3.56 (t, J = 8.4 Hz, 2H), 3.02 (t, J = 8.4 Hz, 2H); ^{13}C NMR (126 MHz, Chloroform-d) δ 150.57, 131.81, 129.85, 127.61, 110.62, 110.22, 47.57, 29.71. QEFMS (ESI) calcd for C_8H_8BrN m/z $[M+H]^+$: 197.99129, found: 197.99130. The spectroscopic data is in accordance with the literature.[12]

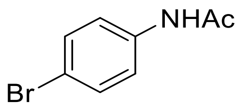

4w, N-(4-bromophenyl)acetamide

The title compound was purified by column chromatology using 30% Et_2O in cyclohexane as a white solid in 98% yield (**3w**: 0.1 mmol, **2c**: 0.005 mg, yield: 209.7 mg/mmol). ^{1}H NMR (500 MHz, Chloroform-d) δ 7.40 (m, 4H), 2.16 (s, 3H); ^{13}C NMR (126 MHz, Chloroform-d) δ 168.44, 136.96, 131.96, 121.42, 116.88, 24.60. QEFMS (APCI) calcd for C_8H_8BrNO m/z $[M+H]^+$: 213.98620, found: 213.98614. The spectroscopic data is in accordance with the literature.[6]

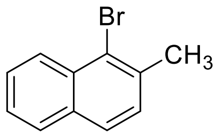

4x, 1-bromo-2-methylnaphthalene

The residue was purified by column chromatology using 1% Et_2O in cyclohexane as a colourless liquid with 94% yield (**3x**: 0.1 mmol, **2c**: 0.05 mg, yield: 207.7 mg/mmol). ^{1}H NMR (500 MHz, Chloroform-d) δ 8.32 (dd, J = 8.6, 1.1 Hz, 1H), 7.81 (dd, J = 8.2, 1.3 Hz, 1H), 7.72 (d, J = 8.3 Hz, 1H), 7.59 (ddd, J = 8.4, 6.9, 1.3 Hz, 1H), 7.48 (ddd, J = 8.1, 6.8, 1.2 Hz, 1H), 7.36 (d, J = 8.3 Hz, 1H), 2.65 (s, 3H); ^{13}C NMR (126 MHz, Chloroform-d) δ 136.02, 133.04, 132.55, 128.73, 128.05, 127.31, 127.30, 126.95, 125.68, 124.06, 24.21. QEFMS (APCI) calcd for $C_{11}H_9Br$ m/z $[M]^+$: 219.98821, found: 219.98831. The spectroscopic data is in accordance with the literature.[14]

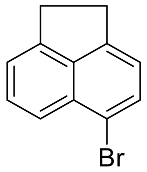

4y, 4-bromoacenaphthene

The residue was purified by column chromatology using 5% Et_2O in cyclohexane as a white solid in 99% yield (**3y**: 0.1 mmol, **2c**: 0.03 mg, yield: 230.7 mg/mmol). ^{1}H NMR (500 MHz, Chloroform-d) δ 7.78 (d, J = 8.4 Hz, 1H), 7.66 (d, J = 7.3 Hz, 1H), 7.56 (d, J = 7.0 Hz, 1H), 7.33 (d, J = 6.9 Hz, 1H), 7.13 (d, J = 7.3 Hz, 1H), 3.37 (dq, J = 39.5, 4.9 Hz, 4H); ^{13}C NMR (126 MHz, Chloroform-d) δ 146.26, 145.96, 140.27, 130.89, 129.07, 121.76, 120.19, 120.06, 116.78, 30.65, 29.93. QEFMS (APCI) calcd for $C_{12}H_9Br$ m/z $[M+H]^+$: 232.99604, found: 232.99573.

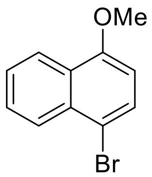

4z, 4-bromo-1-methoxynaphthalene

The title compound was purified by column chromatology using 5% Et_2O in cyclohexane as a colorless oil in 99% yield (**3z**: 0.1 mmol, **2c**: 0.005 mg, yield: 234.6 mg/mmol). ^1H NMR (500 MHz, Chloroform-d) δ 8.28 (d, J = 8.4 Hz, 1H), 8.17 (d, J = 8.5 Hz, 1H), 7.66 (d, J = 8.2 Hz, 1H), 7.61 (t, J = 7.7 Hz, 1H), 7.53 (t, J = 7.6 Hz, 1H), 6.68 (d, J = 8.2 Hz, 1H), 3.99 (s, 3H); ^{13}C NMR (126 MHz, Chloroform-d) δ 155.26, 132.42, 129.47, 127.78, 126.88, 126.81, 125.98, 122.44, 113.26, 104.52, 55.72.; QEFMS (APCI) calcd for $C_{11}H_9BrO$ m/z $[M+H]^+$: 236.99095, found: 236.99101. The spectroscopic data is in accordance with the literature.[6]

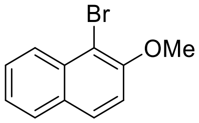

4aa, 1-bromo-2-methoxynaphthalene

The title compound was purified by column chromatology using 5% Et_2O in cyclohexane as a white solid in 97% yield (**3aa**: 0.1 mmol, **2c**: 0.005 mg, yield: 229.9 mg/mmol). ^1H NMR (500 MHz, Chloroform-d) δ 8.23 (d, J = 8.6 Hz, 1H), 7.82 (d, J = 9.0 Hz, 1H), 7.79 (d, J = 8.2 Hz, 1H), 7.57 (t, J = 8.2 Hz, 1H), 7.40 (t, J = 7.9 Hz, 1H), 7.27 (d, J = 9.1 Hz, 1H), 4.03 (s, 3H); ^{13}C NMR (126 MHz, Chloroform-d) δ 153.76, 133.14, 129.83, 129.01, 128.08, 127.78, 126.14, 124.35, 113.60, 108.64, 57.07.; QEFMS (APCI) calcd for $C_{11}H_9BrO$ m/z $[M+H]^+$: 236.99095, found: 236.99100. The spectroscopic data is from the literature.[6]

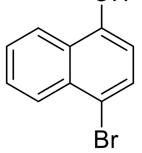

4ab, 4-bromo-1-hydroxynaphthalene

The title compound was purified by column chromatology using 10% Et_2O in cyclohexane as a colorless solid in 93% yield (**3ab**: 0.1 mmol, **2c**: 0.005 mg, yield: 207.4 mg/mmol). ^1H NMR (500 MHz, Chloroform-d) δ 8.33 – 8.00 (m, 2H), 7.62 (ddd, J = 8.4, 6.8, 1.3 Hz, 1H), 7.59 (d, J = 8.0 Hz, 1H), 7.55 (ddd, J = 8.2, 6.8, 1.2 Hz, 1H), 6.70 (d, J = 8.0 Hz, 1H), 5.44 (s, 1H); ^{13}C NMR (126 MHz, Chloroform-d) δ 151.29,

132.73, 129.40, 127.90, 127.08, 126.05, 125.64, 122.20, 113.43, 109.19. QEFMS (ESI) calcd for $C_{10}H_7BrO$ m/z $[M-H]^-$: 220.96075, found: 220.96077.

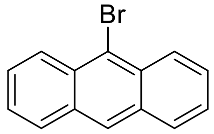

4ac, 9-bromoanthracene

The residue was purified by thin layer chromatography as a yellow oil in 71% yield (**3ac**: 0.1 mmol, **2c**: 0.05 mg, yield: 182.5 mg/mmol). ^{1}H NMR (500 MHz, Chloroform-d) δ 8.52 (d, J = 8.8 Hz, 2H), 8.42 (s, 1H), 7.98 (d, J = 8.5 Hz, 2H), 7.60 (ddd, J = 8.7, 6.6, 1.3 Hz, 2H), 7.50 (ddd, J = 8.0, 6.5, 1.1 Hz, 2H); ^{13}C NMR (126 MHz, Chloroform-d) δ 132.17, 130.60, 128.62, 127.64, 127.22, 127.13, 125.66, 122.38. QEFMS (APCI) calcd for $C_{14}H_9Br$ m/z $[M+H]^+$: 256.99604, found: 256.99606. The spectroscopic data is in accordance with the literature.[6]

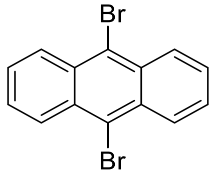

4ad, 9,10-dibromoanthracene

The residue was purified by thin layer chromatography as a yellow solid in 97% yield (**3ad**: 0.1 mmol, **2c**: 0.05 mg, yield: 325.9 mg/mmol). ^{1}H NMR (500 MHz, Chloroform-d) δ 8.58 (dq, J = 6.0, 2.8 Hz, 1H), 7.63 (dq, J = 5.9, 2.7 Hz, 1H); ^{13}C NMR (126 MHz, Chloroform-d) δ 131.06, 128.30, 127.48, 123.55. QEFMS (ESI) calcd for $C_{14}H_8Br_2$ m/z $[M]^+$: 335.89673, found: 335.89664. The spectroscopic data is in accordance with the literature.[6]

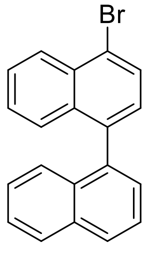

4ae, 4-bromo-1-(1-naphthyl)naphthalene

The residue was purified by column chromatology using 5% Et_2O in cyclohexane as a white solid in 83% yield (**3ae**: 0.1 mmol, **2c**: 0.05 mg, yield: 276.4 mg/mmol). ^{1}H NMR (500 MHz, Chloroform-d) δ 8.39 (d, J = 8.5 Hz, 1H), 7.98 (t, J = 8.5 Hz, 2H), 7.92 (d, J = 7.6 Hz, 1H), 7.61 (ddd, J = 8.7, 6.8, 1.9 Hz, 2H), 7.54 – 7.46 (m, 2H), 7.42 (d, J = 8.4 Hz, 1H), 7.40 – 7.28 (m, 4H); ^{13}C NMR (126 MHz, Chloroform-d) δ 138.65, 137.55, 134.13, 133.54, 132.71, 131.96, 129.54, 128.27, 128.19, 127.89, 127.42, 127.32,

127.21, 126.84, 126.38, 126.23, 125.99, 125.40, 122.67. QEFMS (APCI) calcd for $C_{20}H_{13}Br$ m/z $[M+H]^+$: 333.02734, found: 333.02713. The spectroscopic data is in accordance with the literature. [15]

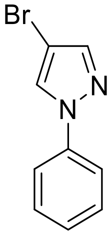

4af, 4-bromo-1-phenylpyrazole

The residue was purified by column chromatology using 10% Et_2O in cyclohexane as a white solid with 99% yield (**3af**: 0.1 mmol, **2c**: 0.05 mg, yield: 220.8 mg/mmol). ^1H NMR (500 MHz, Chloroform-d) δ 7.93 (s, 1H), 7.67 (s, 1H), 7.64 (d, J = 8.1 Hz, 2H), 7.46 (t, J = 7.7 Hz, 2H), 7.31 (t, J = 7.4 Hz, 1H); ^{13}C NMR (126 MHz, Chloroform-d) δ 141.54, 139.65, 129.57, 127.07, 127.04, 119.04, 95.65. HRMS (ESI) calcd for $C_9H_7BrN_2$ m/z $[M+H]^+$: 222.98654, found: 222.98661. The spectroscopic data is in accordance with the literature.[13]

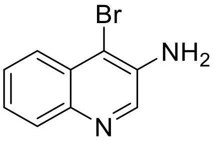

4ag, 4-bromoquinolin-3-amine

The residue was purified by column chromatology using 10% Et_2O in cyclohexane as a white solid with 91% yield (**3ag**: 0.1 mmol, **2c**: 0.03 mg, yield: 202.9 mg/mmol). ^1H NMR (500 MHz, Chloroform-d) δ 8.47 (s, 1H), 7.96 (d, J = 8.5 Hz, 3H), 7.54 (ddd, J = 8.3, 7.0, 1.3 Hz, 1H), 7.48 (ddd, J = 8.2, 6.8, 1.4 Hz, 1H), 4.44 (s, 2H); ^{13}C NMR (126 MHz, Chloroform-d) δ 142.86, 141.73, 138.39, 129.48, 128.21, 128.19, 126.00, 124.70, 112.65. HRMS (ESI) calcd for $C_9H_7BrN_2$ m/z $[M+H]^+$: 222.98654, found: 222.98677.

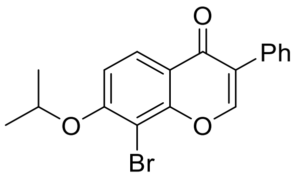

4ah, 8-bromo7-isopropoxyisoflavone

The residue was purified by column chromatology using 5% Et_2O in cyclohexane as a white solid in 62% yield (**3ah**: 0.1 mmol, **2c**: 2.7 mg, yield: 222.6 mg/mmol). ^1H NMR (500 MHz, Chloroform-d) δ 8.23 (d, J = 9.0 Hz, 1H), 8.06 (s, 1H), 7.56 (d, J = 7.2 Hz, 2H), 7.42 (dt, J = 26.6, 7.3 Hz, 3H), 7.04 (d, J = 9.0 Hz, 1H), 4.78 (p, J = 6.1 Hz, 1H), 1.46 (d, J = 6.1 Hz, 6H); ^{13}C NMR (126 MHz, Chloroform-d) δ 175.56, 159.45, 154.60,

153.02, 131.64, 129.08, 128.66, 128.44, 126.57, 125.26, 119.34, 112.08, 100.90, 72.92, 22.19. QEFMS (ESI) calcd for $C_{18}H_{15}BrO_3$ m/z $[M+Na]^+$: 381.00968, found: 381.00979.

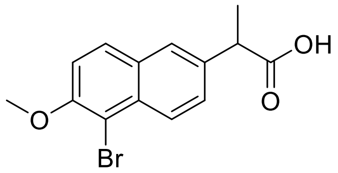

4ai, 5-bromo-naproxen

The residue was purified by column chromatology using 10% Et_2O in cyclohexane as a white solid in 95% yield (**3ai**: 0.1 mmol, **2c**: 0.05 mg, yield: 293.6 mg/mmol). ^1H NMR (500 MHz, Chloroform-d) δ 8.17 (d, J = 8.8 Hz, 1H), 7.76 (d, J = 9.0 Hz, 1H), 7.69 (s, 1H), 7.52 (d, J = 8.8 Hz, 1H), 7.24 (d, J = 2.9 Hz, 1H), 4.01 (s, 3H), 3.89 (q, J = 7.1 Hz, 1H), 1.59 (d, J = 7.2 Hz, 3H); ^{13}C NMR (126 MHz, Chloroform-d) δ 180.03, 153.83, 135.69, 132.46, 129.74, 128.88, 127.65, 126.78, 126.44, 113.94, 108.51, 57.07, 45.01, 18.07. QEFMS (ESI) calcd for $C_{14}H_{13}BrO_3$ m/z $[M+Na]^+$: 330.99403, found: 330.99411.

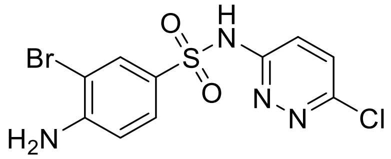

4aj, Sulfachloropyridazine-Br

The residue was purified by column chromatology using 5% Et_2O in cyclohexane as a white solid in 77% yield (**3aj**: 0.1 mmol, **2c**: 0.05 mg, yield: 280.0 mg/mmol). ^1H NMR (500 MHz, Chloroform-d) δ 10.65 (s, 1H), 7.98 (d, J = 2.2 Hz, 1H), 7.64 (dd, J = 8.6, 2.2 Hz, 1H), 7.58 – 7.47 (m, 1H), 7.38 (d, J = 9.5 Hz, 1H), 6.73 (d, J = 8.6 Hz, 1H), 4.66 (s, 2H); ^{13}C NMR (126 MHz, Chloroform-d) δ 152.84, 148.60, 132.05, 131.89, 128.93, 127.76, 114.52, 107.86. QEFMS (ESI) calcd for $C_{10}H_8BrClN_4O_2S$ m/z $[M+Na]^+$: 386.91094, found: 386.91088.

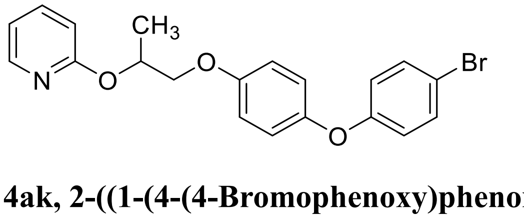

4ak, 2-((1-(4-(4-Bromophenoxy)phenoxy)propan-2-yl)oxy)pyridine

The residue was purified by column chromatology using 10% Et_2O in cyclohexane as a white solid in 84% yield (**3ak**: 0.1 mmol, **2c**: 0.05 mg, yield: 336.0 mg/mmol). ^1H NMR (500 MHz, Chloroform-d) δ 8.16 (dd, J = 5.1, 1.9 Hz, 1H), 7.58 (td, J = 8.4, 7.9, 2.0 Hz, 1H), 7.38 (d, J = 8.7 Hz, 2H), 6.99 – 6.90 (m, 4H), 6.87 (dd, J = 7.0, 5.2 Hz,

1H), 6.81 (d, J = 8.7 Hz, 2H), 6.76 (d, J = 8.4 Hz, 1H), 5.60 (h, J = 5.9 Hz, 1H), 4.19 (dd, J = 9.9, 5.4 Hz, 1H), 4.08 (dd, J = 9.9, 4.8 Hz, 1H), 1.49 (d, J = 6.4 Hz, 3H); ^{13}C NMR (126 MHz, Chloroform-d) δ 163.04, 157.79, 155.51, 149.77, 146.56, 138.96, 132.51, 129.63, 120.88, 119.21, 116.85, 115.90, 111.79, 71.04, 69.51, 17.02. QEFMS (ESI) calcd for $C_{20}H_{18}BrNO_3$ m/z $[M+Na]^+$: 422.03623, found: 422.03625. The spectroscopic data is in accordance with the literature. [16]

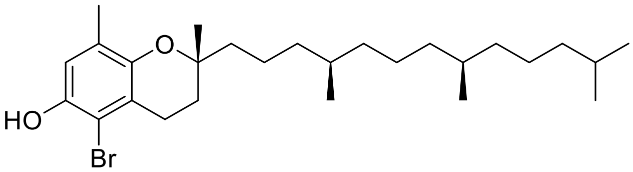

4al, Br-(+)-δ-tocopherol

The residue was purified by column chromatology using 10% Et_2O in cyclohexane as a white solid in 46% yield (**3al**: 0.1 mmol, **2c**: 0.05 mg, yield: 221.3 mg/mmol). ^{1}H NMR (500 MHz, Chloroform-d) δ 6.73 (s, 1H), 5.07 (s, 1H), 2.68 (t, J = 6.9 Hz, 2H), 2.11 (s, 3H), 1.80 (dh, J = 20.3, 6.9 Hz, 2H), 1.52 (dtd, J = 17.5, 12.8, 6.4 Hz, 3H), 1.40 (m, 21H), 0.86 (m, 16H); ^{13}C NMR (126 MHz, Chloroform-d) δ 146.37, 144.84, 126.96, 120.14, 115.17, 109.04, 75.44, 39.48, 37.46, 37.42, 37.30, 32.82, 32.70, 31.36, 28.01, 24.83, 24.47, 24.10, 23.72, 22.75, 22.66, 20.97, 19.78, 19.67, 16.05. QEFMS (ESI) calcd for $C_{27}H_{45}BrO_2$ m/z $[M-H]^+$: 479.25302, found: 479.25311. The spectroscopic data is in accordance with the literature.[7]

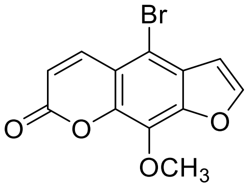

4am, 4-Bromo-9-methoxy-7H-furo[3,2-g]chromen-7-one

The residue was purified by column chromatology using 5% Et_2O in cyclohexane as a white solid in 90% yield (**3am**: 0.1 mmol, **2c**: 0.05 mg, yield: 265.5 mg/mmol). ^{1}H NMR (500 MHz, Chloroform-d) δ 8.08 (d, J = 9.9 Hz, 1H), 7.71 (s, 1H), 6.84 (s, 1H), 6.42 (d, J = 9.9 Hz, 1H), 4.26 (s, 3H); ^{13}C NMR (126 MHz, Chloroform-d) δ 159.76, 146.94, 146.56, 143.64, 142.60, 132.38, 127.97, 115.81, 115.62, 107.43, 105.48, 61.41. QEFMS (ESI) calcd for $C_{12}H_7BrO_4$ m/z $[M+Na]^+$: 316.94199, found: 316.94209. The spectroscopic data is in accordance with the literature. [16]

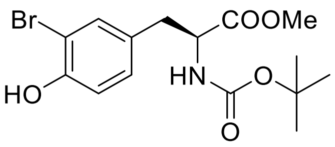

4an, Boc-L-Tyr-OMe-Br

The residue was purified by column chromatology using 20% Et_2O in cyclohexane as

a white solid in 69% yield (**3an**: 0.1 mmol, **2c**: 0.05 mg, yield: 258.1 mg/mmol). ^{1}H NMR (500 MHz, Chloroform-d) δ 7.21 (d, J = 2.0 Hz, 1H), 6.94 (dd, J = 8.4, 2.2 Hz, 1H), 6.88 (d, J = 8.3 Hz, 1H), 6.02 (s, 1H), 5.09 – 5.00 (m, 1H), 4.51 (q, J = 6.6 Hz, 1H), 3.71 (s, 3H), 3.03 (dd, J = 14.1, 5.9 Hz, 1H), 2.93 (dd, J = 14.0, 6.2 Hz, 1H), 1.41 (d, J = 5.4 Hz, 9H); ^{13}C NMR (126 MHz, Chloroform-d) δ 172.23, 155.17, 151.61, 132.84, 130.36, 129.90, 129.48, 116.18, 110.06, 80.25, 54.48, 52.38, 37.19, 28.31. QEFMS (ESI) calcd for $C_{15}H_{20}BrNO_5$ m/z $[M+Na]^+$: 396.04171, found: 396.04200. The spectroscopic data is in accordance with the literature. [18]

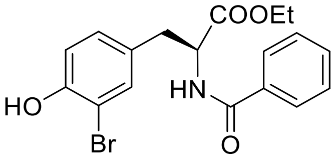

4ao, Bz-L-Tyr-OEt-Br

The residue was purified by column chromatology using 20% Et_2O in cyclohexane as a white solid in 95% yield (**3ao**: 0.1 mmol, **2c**: 0.05 mg, yield: 372.4 mg/mmol). ^{1}H NMR (500 MHz, Chloroform-d) δ 7.75 (dd, J = 7.9, 1.5 Hz, 2H), 7.55 – 7.47 (m, 1H), 7.44 (t, J = 7.6 Hz, 2H), 6.98 (dd, J = 8.4, 2.1 Hz, 1H), 6.91 (d, J = 8.3 Hz, 1H), 6.69 (d, J = 7.4 Hz, 1H), 5.01 (dt, J = 7.3, 5.5 Hz, 1H), 4.23 (qd, J = 7.2, 2.1 Hz, 2H), 3.20 (dd, J = 14.0, 6.0 Hz, 1H), 3.14 (dd, J = 14.0, 5.1 Hz, 1H), 1.30 (t, J = 7.1 Hz, 3H); ^{13}C NMR (126 MHz, Chloroform-d) δ 171.51, 167.01, 151.63, 133.79, 132.93, 131.94, 130.12, 129.43, 128.72, 127.04, 116.17, 110.09, 61.92, 53.68, 36.80, 14.23. QEFMS (ESI) calcd for $C_{18}H_{18}BrNO_4$ m/z $[M+Na]^+$: 414.03114, found: 414.03098.

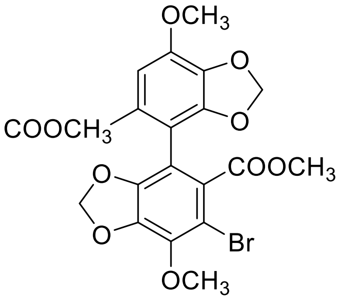

4ap, Dimethyl 6-bromo-7,7'-dimethoxy-[4,4'-bibenzo[d][1,3]dioxole]-5,5'-dicarbo-xylate

The residue was purified by column chromatology using 10% Et_2O in cyclohexane as a white solid in 51% yield (**3ap**: 0.1 mmol, **2c**: 2.7 mg, yield: 253.5 mg/mmol). ^{1}H NMR (500 MHz, Chloroform-d) δ 7.31 (s, 1H), 6.05 (d, J = 1.4 Hz, 1H), 6.02 – 5.97 (m, 4H), 4.07 (s, 3H), 3.95 (s, 3H), 3.70 (s, 3H), 3.60 (s, 3H); ^{13}C NMR (126 MHz, Chloroform-d) δ 166.95, 166.12, 148.02, 146.94, 143.12, 140.12, 138.27, 137.75, 129.93, 124.45, 111.37, 110.77, 109.30, 105.66, 102.74, 102.29, 60.39, 56.74, 52.47,

52.28. QEFMS (ESI) calcd for $C_{20}H_{17}BrO_{10}$ m/z $[M+Na]^+$: 518.98973, found: 518.99008. The spectroscopic data is in accordance with the literature. [16]

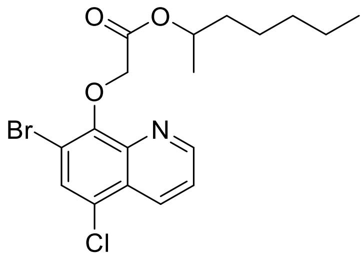

4aq, (±)-Cloquintocet-mexyl-Br

The residue was purified by column chromatology using 5% Et_2O in cyclohexane as a white solid in 67% yield (**3aq**: 0.1 mmol, **2c**: 2.7 mg, yield: 277.8 mg/mmol). ^{1}H NMR (500 MHz, Chloroform-d) δ 8.92 (dd, J = 4.2, 1.6 Hz, 1H), 8.54 (dd, J = 8.5, 1.6 Hz, 1H), 7.81 (s, 1H), 7.56 (dd, J = 8.6, 4.2 Hz, 1H), 5.21 (d, J = 1.7 Hz, 2H), 5.02 (h, J = 6.3 Hz, 1H), 1.58 (dt, J = 12.5, 7.3 Hz, 1H), 1.46 (ddd, J = 14.4, 9.1, 5.5 Hz, 1H), 1.24 (t, J = 6.0 Hz, 9H), 0.92 – 0.82 (m, 3H); ^{13}C NMR (126 MHz, Chloroform-d) δ 168.74, 150.53, 149.74, 142.03, 134.09, 130.59, 126.66, 126.01, 122.11, 115.07, 72.26, 70.96, 35.82, 31.61, 25.01, 22.56, 19.95, 14.01. QEFMS (ESI) calcd for $C_{18}H_{21}BrClNO_3$ m/z $[M+Na]^+$: 438.02644, found: 438.02571. The spectroscopic data is in accordance with the literature.[17]

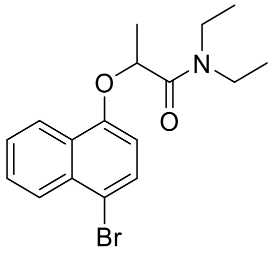

4ar, 2-((4-Bromonaphthalen-1-yl)oxy)-N,N-diethylpropanamide

The residue was purified by column chromatology using 5% Et_2O in cyclohexane as a white solid in 89% yield (**3ar**: 0.1 mmol, **2c**: 0.05 mg, yield: 311.5 mg/mmol). ^{1}H NMR (500 MHz, Chloroform-d) δ 8.32 (d, J = 8.4 Hz, 1H), 8.16 (d, J = 8.4 Hz, 1H), 7.65 – 7.56 (m, 2H), 7.53 (ddd, J = 8.3, 6.8, 1.2 Hz, 1H), 6.68 (d, J = 8.2 Hz, 1H), 5.09 (q, J = 6.7 Hz, 1H), 3.55 (dq, J = 14.3, 7.1 Hz, 1H), 3.46 – 3.38 (m, 1H), 3.41 – 3.30 (m, 2H), 1.73 (d, J = 6.7 Hz, 3H), 1.10 (q, J = 6.8 Hz, 3H), 1.01 (t, J = 7.1 Hz, 3H); ^{13}C NMR (126 MHz, Chloroform-d) δ 169.87, 153.06, 132.64, 129.37, 127.89, 126.95, 126.18, 122.51, 114.09, 106.45, 74.62, 41.12, 40.43, 17.98, 14.17, 12.64. QEFMS (ESI) calcd for $C_{17}H_{20}BrNO_2$ m/z $[M+Na]^+$: 372.05696, found: 372.05701. The spectroscopic data is in accordance with the literature.[16]

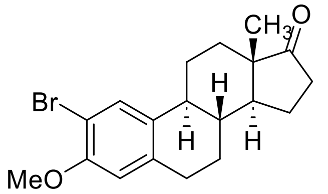

4as, (8R,9S,13S,14S)-2-Bromo-3-methoxy-13-methyl-6,7,8,9,11,12,13,14,15,16-decahydro-17Hcyclopenta[a]phenanthren-17-one

The residue was purified by column chromatology using 10% Et_2O in cyclohexane as a white solid in 98% yield (**3as**: 0.1 mmol, **2c**: 0.05 mg, yield: 355.7 mg/mmol). ^1H NMR (500 MHz, Chloroform-d) δ 7.42 (d, J = 1.1 Hz, 1H), 6.62 (s, 1H), 3.85 (s, 3H), 2.86 (dd, J = 8.9, 4.0 Hz, 2H), 2.55 – 2.46 (m, 1H), 2.38 – 2.28 (m, 1H), 2.23 (td, J = 10.6, 4.4 Hz, 1H), 2.14 (dt, J = 19.0, 8.9 Hz, 1H), 2.09 – 1.98 (m, 2H), 1.98 – 1.92 (m, 1H), 1.68 – 1.60 (m, 1H), 1.63 – 1.53 (m, 1H), 1.56 – 1.47 (m, 2H), 1.50 – 1.36 (m, 2H), 0.90 (s, 3H); ^{13}C NMR (126 MHz, Chloroform-d) δ 153.71, 136.99, 133.59, 130.25, 112.32, 108.74, 56.23, 50.30, 47.96, 43.74, 38.10, 35.86, 31.48, 29.48, 26.40, 25.92, 21.58, 13.85. QEFMS (ESI) calcd for $C_{19}H_{23}BrO_2$ m/z $[M+Na]^+$: 385.07736, found: 385.07694. The spectroscopic data is in accordance with the literature.[16]

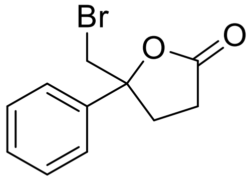

13, colorless oil with a 99% yield (**12**: 0.1 mmol, **2c**: 0.005 mg, yield: 252.4 mg/mmol). ^1H NMR (500 MHz, Chloroform-d) δ 7.47 – 7.37 (m, 4H), 7.40 – 7.32 (m, 1H), 3.74 (d, J = 11.3 Hz, 1H), 3.69 (d, J = 11.4 Hz, 1H), 2.88 – 2.75 (m, 2H), 2.62 – 2.48 (m, 2H). ^{13}C NMR (126 MHz, Chloroform-d) δ 175.59, 140.75, 128.89, 128.71, 124.93, 86.47, 41.09, 32.42, 29.11. QEFMS (APCI) calcd for $C_{11}H_{11}BrO_2$ m/z $[M+H]^+$: 255.00152, found: 255.00078. The spectroscopic data is in accordance with the literature.[12]

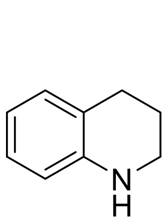

15, 97% yellow oil (**14**: 0.1 mmol, **2c**: 2.7 mg, yield: 129.0 mg/mmol). ^1H NMR (500 MHz, Chloroform-d) δ 7.03 – 6.93 (m, 2H), 6.62 (td, J = 7.3, 1.2 Hz, 1H), 6.49 (dd, J = 7.9, 1.2 Hz, 1H), 3.34 – 3.26 (m, 2H), 2.77 (t, J = 6.4 Hz, 2H), 2.01 – 1.88 (m, 2H);

^{13}C NMR (126 MHz, Chloroform-d) δ 144.63, 129.55, 126.75, 121.57, 117.09, 114.31, 42.00, 26.96, 22.16. QEFMS (APCI) calcd for $C_9H_{11}N$ m/z $[M+H]^+$: 134.09643, found: 134.09614. The spectroscopic data is in accordance with the literature.[37]

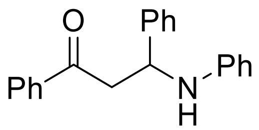

19, white solid in 92% yields (**16**, **17**, **18**: 1.0 mmol, **2c**: 5.4 mg, yield: 276.9 mg/mmol). ^1H NMR (500 MHz, Chloroform-d) δ 7.91 (d, J = 7.6 Hz, 2H), 7.57 (t, J = 7.4 Hz, 1H), 7.45 (t, J = 6.8 Hz, 4H), 7.33 (t, J = 7.6 Hz, 2H), 7.24 (t, J = 7.4 Hz, 1H), 7.10 (t, J = 7.8 Hz, 2H), 6.67 (t, J = 7.3 Hz, 1H), 6.58 (d, J = 7.9 Hz, 2H), 5.11 – 4.95 (m, 1H), 4.66 (s, 11H), 3.65 – 3.28 (m, 2H); ^{13}C NMR (126 MHz, Chloroform-d) δ 198.29, 146.87, 142.89, 136.67, 133.48, 129.14, 128.86, 128.73, 128.24, 127.41, 126.42, 117.91, 113.93, 54.90, 46.29. QEFMS (APCI) calcd for $C_{21}H_{19}NO$ m/z $[M+H]^+$: 302.15394, found: 302.15349. The spectroscopic data is in accordance with the literature.[25]

4. Computational studies

The geometry optimization and single-point energy calculations were carried out in Gaussian 16 (version C.02)[23] and ORCA (version 6.1.0)[24] with a CPCM solvent model (1,2-dichloroethane).[22] The optimization was carried out at the level of B3LYP/def2-SVP with Grimme's D3(BJ) dispersion correction.[19-21] The single-point energies were calculated with ωB97M-V functional[26] with def2-QZVPP basis set[27]. The auxiliary basis set def2/J[28] was utilized for Resolution of Identity (RI) approximation to accelerate the ORCA calculations. To investigate the interactions within binary complexes, constrained molecular simulations were conducted using xTB[29] with the GFN2-xTB model.[30] Several optimized configurations were obtained, and the one with the lowest potential energy was selected for further study. Natural Bond Orbital NBO analysis,[31] including second-order perturbation E(2) analyses. were performed at the level of B3LYP/def2-TZVP[25] using NBO 7.0.[32] Electrostatic potential (ESP) analysis[33] and pre-orthogonal natural atomic orbital(PNAO)[34] visualization were carried out using Multiwfn 3.8[35-36] and VMD 1.9.3.[37]

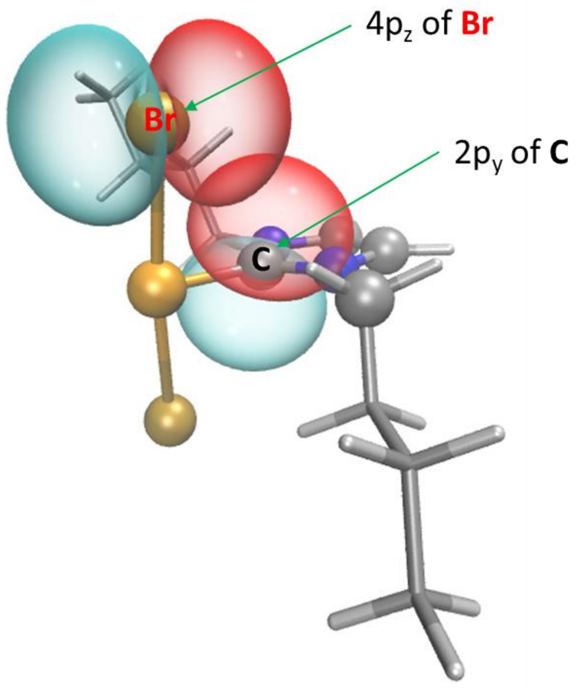

Figure S8. Computational study on the bonding nature in **2a**

Note: atomic orbital analysis of ***2a*** *indicates that the lone-pair electrons of Br (primarily the 4*p_z *orbital) overlap effectively with the 2*p_y *orbital of the imidazolium carbon.*

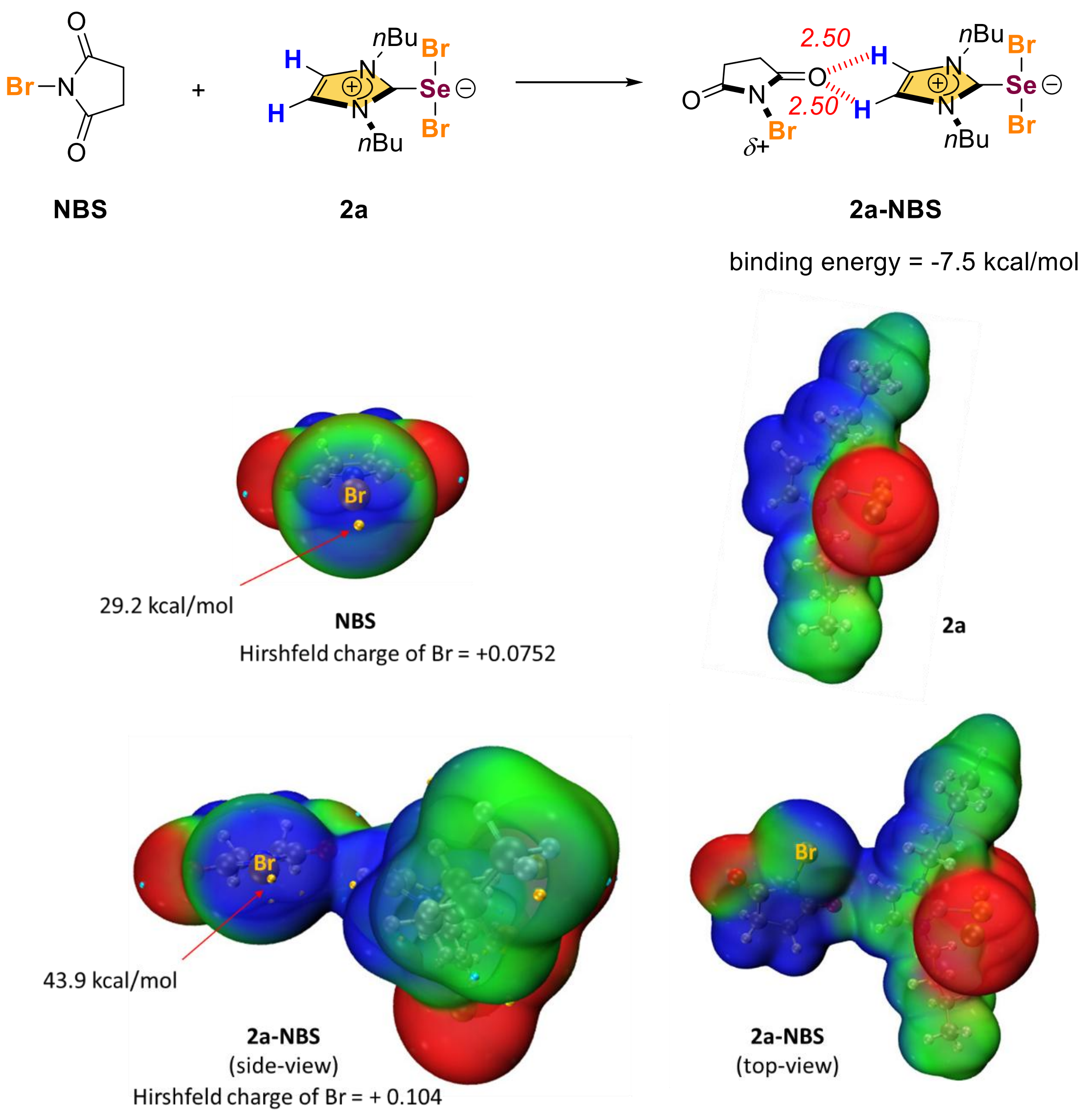

Figure S9. Computational study on the complex **2a-NBS**

*Note: electrostatic potential analysis on **2a** indicates that the negative charge is localized at the SeBr$_2$ moiety (the red region in **2a**). The relatively electronegative bromine atoms could stabilize the anionic charge on the selenium, leading to a site-isolated and unoccupied imidazolium cation that could serve as strong C-H non-classical hydrogen bond donors (the blue region in **2a**) to activate NBS. The complex **2a-NBS** was optimized by our DFT calculations to give a binding energy of -7.5 kcal/mol. The C-H...O bond length (2.50 Å) was found to be shorter than the sum of their van der Waals radii. For NBS, the maximum electrostatic potential (MEP) of the Br on the electron density isosurface (at 0.001 a.u.) was found to be 29.2 kcal/mol. For complex **2a-NBS**, the MEP increases significantly to 43.9 kcal/mol. The Hirshfeld charge of Br also increases after the formation of **2a-NBS**. These results suggest that binding of NBS with **2a** significantly increases the electrophilicity of the Br.*

Cartesian Coordinates

Complex NBS

E (ωB97M-V def2-QZVPP def2/J)= -2934.148826861567

Br	-1.69004200	-0.00000100	-0.00001000
N	0.16425700	-0.00000100	0.00000000
C	0.91330200	1.18490900	-0.00006300
O	0.44621800	2.29609100	-0.00002400
C	2.37321900	0.76739700	0.00007400
C	2.37322100	-0.76739100	0.00008000
C	0.91330500	-1.18490900	-0.00004500
O	0.44622500	-2.29609300	-0.00002000
H	2.85589300	1.20558200	0.88680500
H	2.85602000	1.20557200	-0.88659400
H	2.85602300	-1.20557200	-0.88658300
H	2.85589800	-1.20556500	0.88681500

Complex 2a

E (ωB97M-V def2-QZVPP def2/J)= -8090.345174827162

C	-0.58072800	0.36110000	2.90337600
C	0.57939900	-0.36308300	2.90338300
N	0.92269100	-0.57780800	1.58862900
C	-0.00017300	-0.00022200	0.79391700
N	-0.92340800	0.57677400	1.58862100
C	-2.12834300	1.27917700	1.13329000
C	2.12783500	-1.27987400	1.13332300
H	-1.18602600	0.73906500	3.72274800
H	1.18434600	-0.74160500	3.72275900
Se	0.00023500	0.00036700	-1.09932200
Br	0.92807300	2.47885300	-0.97127700
Br	-0.92730900	-2.47841900	-0.97240300
H	1.85100700	-1.85609700	0.23996900
H	-2.39361900	1.99347600	1.92513400
C	-3.27938700	0.31957000	0.84292300
H	-3.53083800	-0.23184600	1.76572100
H	-2.94384600	-0.42991900	0.10723800
C	-4.51256800	1.05276500	0.31462600
H	-4.23509100	1.61117900	-0.59734700

H	-4.83346600	1.81052300	1.05196600
C	-5.67181700	0.10612800	0.00604200
H	-5.98822400	-0.44315600	0.90871800
H	-6.54680900	0.65403400	-0.37840300
H	-5.38155000	-0.64054800	-0.75197300
H	-1.85124700	1.85558800	0.24015000
H	2.39307700	-1.99433900	1.92502500
C	3.27876900	-0.31996200	0.84344800
H	2.94314500	0.42974600	0.10802300
H	3.53003300	0.23113400	1.76648200
C	4.51210700	-1.05272500	0.31492200
H	4.23472400	-1.61099100	-0.59717200
H	4.83325500	-1.81058000	1.05205700
C	5.67110400	-0.10574000	0.00645900
H	6.54625400	-0.65337500	-0.37801900
H	5.38064200	0.64090900	-0.75151000
H	5.98735200	0.44356200	0.90918300

Complex 2a-NBS

E (ωB97M-V def2-QZVPP def2/J)= -11024.505943750048

C	-0.40457700	-0.94452100	-1.03195300
C	-0.73728900	0.24223400	-0.44571300
N	0.41744600	0.76710500	0.07900100
C	1.43482000	-0.06819300	-0.17517300
N	0.94572400	-1.11647000	-0.85322400
C	1.71868800	-2.28386700	-1.28337900
C	0.53344600	2.05592500	0.76535600
H	-1.02276400	-1.66519300	-1.55735500
H	-1.70053900	0.73626900	-0.36217400
Se	3.23851900	0.18634200	0.33523100
Br	3.64418000	0.86016000	-2.15042900
Br	2.45879300	-0.54622800	2.71574900
H	1.25035700	1.91878600	1.58685200
H	1.17728900	-2.72314800	-2.13188600
C	1.89675700	-3.29108000	-0.15483200
H	0.90504200	-3.63579400	0.18306000
H	2.36711100	-2.78377600	0.70343300

C	2.74731600	-4.48108400	-0.58625500
H	3.72512500	-4.11349400	-0.93816900
H	2.27362000	-4.97767000	-1.44955600
C	2.94761400	-5.48387500	0.54425800
H	1.98192000	-5.87388900	0.89972200
H	3.55957900	-6.33763400	0.22196900
H	3.45014600	-5.00911900	1.39988200
H	2.68535900	-1.91369700	-1.65269300
H	-0.45058600	2.26710100	1.20502100
C	0.97772700	3.16286700	-0.18187300
H	1.89756400	2.84047100	-0.69733700
H	0.20943700	3.30650800	-0.96015900
C	1.23047000	4.47218700	0.55792100
H	1.99904300	4.30210400	1.32983400
H	0.31577700	4.77641700	1.09405500
C	1.67881400	5.58637700	-0.38078200
H	1.87077100	6.51985100	0.16554400
H	2.60280000	5.30395200	-0.90685200
H	0.91116500	5.79000800	-1.14253800
Br	-4.63130000	1.39551800	0.44106000
N	-5.25150300	-0.22274800	-0.14892000
C	-4.49154300	-1.07283100	-0.94942000
O	-3.37354900	-0.84598300	-1.32260000
C	-5.34994000	-2.28916800	-1.23413000
C	-6.66557800	-2.05665400	-0.48749200
C	-6.53496100	-0.69698700	0.17176100
O	-7.32912100	-0.10591700	0.84020900
H	-4.80223300	-3.17995000	-0.89878800
H	-5.47135700	-2.36760000	-2.32308400
H	-7.54844000	-2.03782800	-1.14010000
H	-6.85530100	-2.79705400	0.30152600

4. References

[1] H. Zhao, F. W. Foss, R. Breslow, *J. Am. Chem. Soc.* **2008**, *130*, 12590-12591.

[2] W. P. Teh, D. C. Obenschain, B. M. Black, F. E. Michael, *J. Am. Chem. Soc.* **2020**, *142*, 16716-16722.

[3] X. He, X. Wang, Y.-L. S. Tse, Z. Ke, Y.-Y. Yeung, *ACS Catalysis* **2021**, *11*, 12632-12642.

[4] T. Maibunkaew, C. Thongsornkleeb, J. Tummatorn, A. Bunrit, S. Ruchirawat, *Synlett* **2014**, *25*, 1769-1775.

[5] R.-J. Tang, T. Milcent, B. Crousse, *J. Org. Chem.* **2018**, *83*, 930-938.

[6] X. Xiong, F. Tan, Y.-Y. Yeung, *Org. Lett.* **2017**, *19*, 4243-4246.

[7] S. Song, X. Sun, X. Li, Y. Yuan, N. Jiao, *Org. Lett.* **2015**, *17*, 2886-2889.

[8] M. Bal, W. Van Hoey, R. Cleirbaut, F. Lemière, S. Van Doorslaer, P. Cool, B. U. W. Maes, *ACS Catal.* **2025**, *15*, 4726-4738.

[9] S. P. Borikar, T. Daniel, V. Paul, *Tetrahedron Lett.* **2009**, *50*, 1007-1009.

[10] A. Granados, A. Shafir, A. Arrieta, F. P. Cossío, A. Vallribera, *J. Org. Chem.* **2020**, *85*, 2142-2150.

[11] P. Lu, X. Ren, H. Xu, D. Lu, Y. Sun, Z. Lu, *J. Am. Chem. Soc.* **2021**, *143*, 12433-12438.

[12] J. C. Borghs, V. Zubar, L. M. Azofra, J. Sklyaruk, M. Rueping, *Org. Lett.* **2020**, *22*, 4222-4227.

[13] H. Moradi, B. Karimi, T. Karimpour, P. Mastrorilli, S. Todisco, *Dalton Trans.* **2025**, *54*, 2512-2528.

[14] B. Han, C. Ren, L. Wu, *Organometallics* **2023**, *42*, 1248-1253.

[15] S. Hainke, I. Singh, J. Hemmings, O. Seitz, *J. Org. Chem.* **2007**, *72*, 8811-8819.

[16] H. Shi, J. Zhang, X. Li, J. He, Y. Sun, J. Wu, Y. Du, *Chem. Sci.* **2024**, *15*, 13058-13067.

[17] Y. Wang, C. Bi, Y. Kawamata, L. N. Grant, L. Samp, P. F. Richardson, S. Zhang, K. C. Harper, M. D. Palkowitz, A. Vasilopoulos, M. R. Collins, M. S. Oderinde, C. C. Tyrol, D. Chen, E. A. LaChapelle, J. B. Bailey, J. X. Qiao, P. S. Baran, *Nature Chem.* **2024**, *16*, 1539-1545.

[18] A. Walter, G. Storch, *Angew. Chem. Int. Ed.* **2020**, *59*, 22505-22509.

[19] P. J. Stephens, F. J. Devlin, C. F. Chabalowski, M. J. Frisch, *J. Phys. Chem.* **1994**, *98*, 11623–11627.

[20] S. Grimme, J. Antony, S. Ehrlich, H. Krieg, *J. Chem. Phys.* **2010**, *132*, 154104.

[21] S. Grimme, S. Ehrlich, L. Goerigk, *J. Comput. Chem.* **2011**, *32*, 1456–1465.

[22] V. Barone, M. Cossi, *J. Phys. Chem. A* **1998**, *102*, 1995–2001.

[23] M. J. Frisch, G. W. Trucks, H. B. Schlegel, G. E. Scuseria, M. A. Robb, J. R. Cheeseman, G. Scalmani, V. Barone, G. A. Petersson, H. Nakatsuji, X. Li, M. Caricato,

A. V. Marenich, J. Bloino, B. G. Janesko, R. Gomperts, B. Mennucci, H. P. Hratchian, J. V. Ortiz, A. F. Izmaylov, J. L. W. Sonnenberg, F. Ding, F. Lipparini, F. Egidi, J. Goings, B. Peng, A. Petrone, T. Henderson, D. Ranasinghe, V. G. Zakrzewski, J. Gao, N. Rega, G. Zheng, W. Liang, M. Hada, M. Ehara, K. Toyota, R. Fukuda, J. Hasegawa, M. Ishida, T. Nakajima, Y. Honda, O. Kitao, H. Nakai, T. Vreven, K. Throssell, J. A. Montgomery Jr., J. E. Peralta, F. Ogliaro, M. J. Bearpark, J. J. Heyd, E. N. Brothers, K. N. Kudin, V. N. Staroverov, T. A. Keith, R. Kobayashi, J. Normand, K. Raghavachari, A. P. Rendell, J. C. Burant, S. S. Iyengar, J. Tomasi, M. Cossi, J. M. Millam, M. Klene, C. Adamo, R. Cammi, J. W. Ochterski, R. L. Martin, K. Morokuma, O. Farkas, J. B. Foresman, D. J. Fox, *Gaussian 16*, C.02.; Gaussian, Inc.: Wallingford, CT, 2016.

[24] F. Neese, *Rev.: Comput. Mol. Sci.*, **2012**, *2*, 1, 73–78.

[25] F. Weigend, R. Ahlrichs, *Phys. Chem. Chem. Phys.* **2005**, *7*, 3297–3305.

[26] N. Mardirossian, M. Head-Gordon, *J. Chem. Phys.* **2016**, *144*, 214110.

[27] F. Weigend, F. Furche, R. Ahlrichs, *J. Chem. Phys.* **2003**, *119*, 12753–12762.

[28] F. Weigend, *Phys. Chem. Chem. Phys.* **2006**, *8*, 1057–1065.

[29] C. Bannwarth, E. Caldeweyher, S. Ehlert, A. Hansen, P. Pracht, J. Seibert, S. Spicher, S. Grimme, *WIREs Comput. Mol. Sci.* **2021**, *11*, e1493.

[30] C. Bannwarth, S. Ehlert, S. Grimme, *J. Chem. Theory Comput.* **2019**, *15*, 1652–1671.

[31] E. D. Glendening, C. R. Landis, F. Weinhold, *WIREs Comput. Mol. Sci.* **2012**, *2*, 1–42.

[32] E. D. Glendening, C. R. Landis, *J. Comput. Chem.* **2019**, *40*, 2234–2241.

[33] P. K. Weiner, R. Langridge, J. M. Blaney, R. Schaefer, P. A. Kollman, *Proc. Natl. Acad. Sci.* **1982**, *79*, 3754–3758.

[34] A. E. Reed, L. A. Curtiss, F. Weinhold, *Chem. Rev.* **1988**, *88*, 899–926.

[35] L. Tian, C. Feiwu, *J. Comput. Chem.* **2012**, *33*, 580–592.

[36] L. Tian, *J. Chem. Phys.* **2024**, *161*, 082503.

[37] W. Humphrey, A. Dalke, K. Schulten, *J. Mol. Graph.* **1996**, *14*, 33–38.

5. X-ray crystallographic data

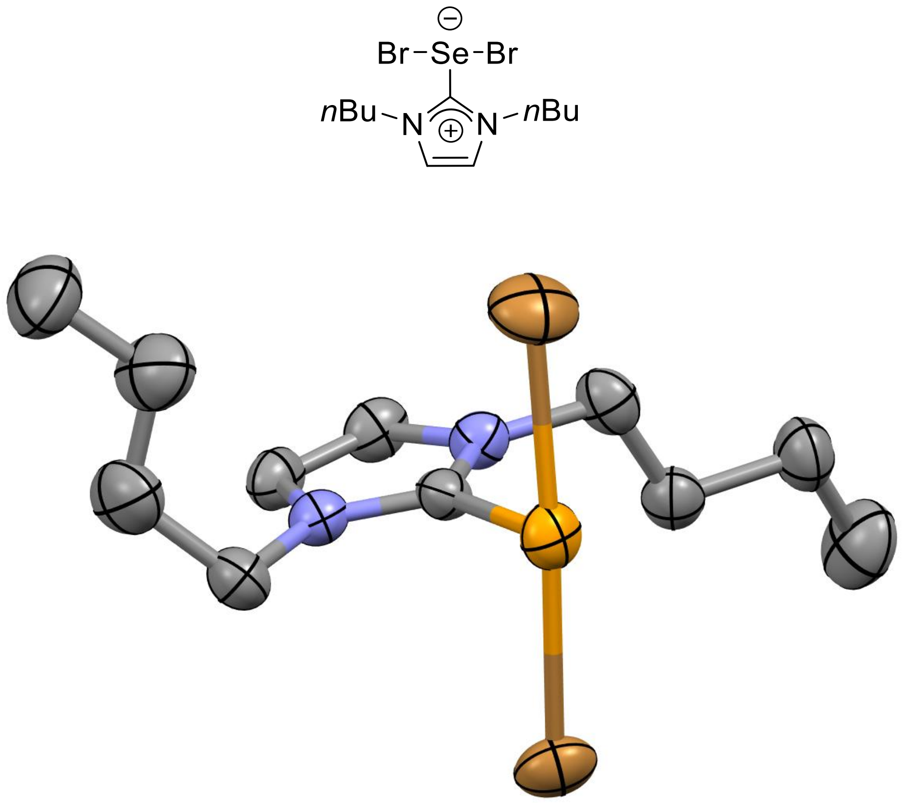

Figure S10. X-ray crystallographic structure of **2a** (CCDC 2358229).

Table S1. Crystal data and structure refinement for **2a**.

Identification code	5senbutylzbr	
Empirical formula	C11 H20 Br2 N2 Se	
Formula weight	419.07	
Temperature	296(2) K	
Wavelength	0.71073 Å	
Crystal system	Monoclinic	
Space group	$P2_1/n$	
Unit cell dimensions	a = 7.4789(5) Å	α= 90°.
	b = 15.9447(11) Å	β= 93.489(3)°.
	c = 13.0545(9) Å	γ = 90°.
Volume	1553.85(18) $Å^3$	
Z	4	
Density (calculated)	1.791 Mg/m^3	
Absorption coefficient	7.540 mm^{-1}	
F(000)	816	
Crystal size	0.500 x 0.300 x 0.300 mm^3	
Theta range for data collection	2.018 to 25.246°.	
Index ranges	-8<=h<=8, -19<=k<=19, -13<=l<=15	
Reflections collected	19277	
Independent reflections	2801 [R(int) = 0.0556]	
Completeness to theta = 25.242°	99.9 %	
Absorption correction	multi-scan	
Max. and min. transmission	0.7456 and 0.1417	
Refinement method	Full-matrix least-squares on F^2	
Data / restraints / parameters	2801 / 0 / 146	
Goodness-of-fit on F^2	1.059	
Final R indices [I>2sigma(I)]	R1 = 0.0436, wR2 = 0.1216	
R indices (all data)	R1 = 0.0603, wR2 = 0.1392	
Extinction coefficient	0.0189(14)	
Largest diff. peak and hole	0.893 and -0.745 e.$Å^{-3}$	

Table S2. Atomic coordinates (x 10^4) and equivalent isotropic displacement parameters ($Å^2$ x 10^3) for **2a**. U(eq) is defined as one third of the trace of the orthogonalized U^{ij} tensor.

	x	y	z	U(eq)
Se(1)	1832(1)	4062(1)	2397(1)	44(1)
Br(1)	800(1)	5395(1)	3390(1)	57(1)
Br(2)	3037(1)	2770(1)	1555(1)	72(1)
N(1)	5429(5)	4348(3)	3231(4)	39(1)
N(2)	3817(5)	3725(2)	4315(3)	38(1)
C(1)	3807(6)	4037(3)	3359(4)	36(1)
C(2)	6480(7)	4239(3)	4112(5)	43(1)
C(3)	5484(7)	3858(3)	4794(4)	44(1)
C(4)	6008(7)	4769(3)	2313(5)	48(1)
C(5)	5979(7)	5712(3)	2414(5)	47(1)
C(6)	6698(8)	6152(4)	1488(5)	58(2)
C(7)	6900(10)	7089(4)	1675(7)	77(2)
C(8)	2297(7)	3326(3)	4801(5)	47(1)
C(9)	2744(10)	2460(4)	5192(6)	69(2)
C(10)	3271(10)	1846(4)	4448(7)	74(2)
C(11)	3523(11)	959(4)	4819(7)	81(2)

Table S3. Bond lengths [Å] and angles [°] for **2a**.

Se(1)-C(1)	1.880(5)
Se(1)-Br(2)	2.5256(8)
Se(1)-Br(1)	2.6305(8)
N(1)-C(1)	1.330(6)
N(1)-C(2)	1.364(7)
N(1)-C(4)	1.462(7)
N(2)-C(1)	1.343(7)
N(2)-C(3)	1.376(6)
N(2)-C(8)	1.480(7)
C(2)-C(3)	1.340(8)
C(2)-H(2A)	0.9300
C(3)-H(3A)	0.9300
C(4)-C(5)	1.510(8)
C(4)-H(4A)	0.9700
C(4)-H(4B)	0.9700
C(5)-C(6)	1.524(8)
C(5)-H(5A)	0.9700
C(5)-H(5B)	0.9700
C(6)-C(7)	1.519(10)
C(6)-H(6A)	0.9700
C(6)-H(6B)	0.9700
C(7)-H(7A)	0.9600
C(7)-H(7B)	0.9600
C(7)-H(7C)	0.9600
C(8)-C(9)	1.502(9)
C(8)-H(8A)	0.9700
C(8)-H(8B)	0.9700
C(9)-C(10)	1.451(11)
C(9)-H(9A)	0.9700
C(9)-H(9B)	0.9700
C(10)-C(11)	1.504(9)
C(10)-H(10A)	0.9700
C(10)-H(10B)	0.9700
C(11)-H(11A)	0.9600
C(11)-H(11B)	0.9600
C(11)-H(11C)	0.9600

C(1)-Se(1)-Br(2)	89.22(14)
C(1)-Se(1)-Br(1)	86.00(14)
Br(2)-Se(1)-Br(1)	175.20(3)
C(1)-N(1)-C(2)	109.3(5)
C(1)-N(1)-C(4)	126.3(4)
C(2)-N(1)-C(4)	124.4(4)
C(1)-N(2)-C(3)	108.6(4)
C(1)-N(2)-C(8)	126.6(4)
C(3)-N(2)-C(8)	124.7(5)
N(1)-C(1)-N(2)	107.5(4)
N(1)-C(1)-Se(1)	126.4(4)
N(2)-C(1)-Se(1)	126.1(3)
C(3)-C(2)-N(1)	107.6(5)
C(3)-C(2)-H(2A)	126.2
N(1)-C(2)-H(2A)	126.2
C(2)-C(3)-N(2)	107.0(5)
C(2)-C(3)-H(3A)	126.5
N(2)-C(3)-H(3A)	126.5
N(1)-C(4)-C(5)	112.3(5)
N(1)-C(4)-H(4A)	109.1
C(5)-C(4)-H(4A)	109.1
N(1)-C(4)-H(4B)	109.1
C(5)-C(4)-H(4B)	109.1
H(4A)-C(4)-H(4B)	107.9
C(4)-C(5)-C(6)	112.4(5)
C(4)-C(5)-H(5A)	109.1
C(6)-C(5)-H(5A)	109.1
C(4)-C(5)-H(5B)	109.1
C(6)-C(5)-H(5B)	109.1
H(5A)-C(5)-H(5B)	107.8
C(7)-C(6)-C(5)	111.2(6)
C(7)-C(6)-H(6A)	109.4
C(5)-C(6)-H(6A)	109.4
C(7)-C(6)-H(6B)	109.4
C(5)-C(6)-H(6B)	109.4
H(6A)-C(6)-H(6B)	108.0
C(6)-C(7)-H(7A)	109.5

C(6)-C(7)-H(7B)	109.5
H(7A)-C(7)-H(7B)	109.5
C(6)-C(7)-H(7C)	109.5
H(7A)-C(7)-H(7C)	109.5
H(7B)-C(7)-H(7C)	109.5
N(2)-C(8)-C(9)	112.3(5)
N(2)-C(8)-H(8A)	109.1
C(9)-C(8)-H(8A)	109.1
N(2)-C(8)-H(8B)	109.1
C(9)-C(8)-H(8B)	109.1
H(8A)-C(8)-H(8B)	107.9
C(10)-C(9)-C(8)	117.2(7)
C(10)-C(9)-H(9A)	108.0
C(8)-C(9)-H(9A)	108.0
C(10)-C(9)-H(9B)	108.0
C(8)-C(9)-H(9B)	108.0
H(9A)-C(9)-H(9B)	107.3
C(9)-C(10)-C(11)	116.9(7)
C(9)-C(10)-H(10A)	108.1
C(11)-C(10)-H(10A)	108.1
C(9)-C(10)-H(10B)	108.1
C(11)-C(10)-H(10B)	108.1
H(10A)-C(10)-H(10B)	107.3
C(10)-C(11)-H(11A)	109.5
C(10)-C(11)-H(11B)	109.5
H(11A)-C(11)-H(11B)	109.5
C(10)-C(11)-H(11C)	109.5
H(11A)-C(11)-H(11C)	109.5
H(11B)-C(11)-H(11C)	109.5

Symmetry transformations used to generate equivalent atoms:

Table S4. Anisotropic displacement parameters ($Å^2 \times 10^3$) for **2a**. The anisotropic displacement factor exponent takes the form: $-2\pi^2[h^2 a^{*2}U^{11} + ... + 2 h k a^* b^* U^{12}]$

	U^{11}	U^{22}	U^{33}	U^{23}	U^{13}	U^{12}
Se(1)	37(1)	53(1)	41(1)	-1(1)	-9(1)	-4(1)
Br(1)	38(1)	56(1)	77(1)	-10(1)	-4(1)	8(1)
Br(2)	73(1)	74(1)	67(1)	-32(1)	-6(1)	2(1)
N(1)	33(2)	39(2)	43(3)	1(2)	0(2)	-2(2)
N(2)	36(2)	35(2)	42(3)	-2(2)	0(2)	1(2)
C(1)	34(3)	39(3)	34(3)	-3(2)	5(2)	2(2)
C(2)	35(3)	45(3)	48(4)	-4(3)	-10(2)	-2(2)
C(3)	46(3)	46(3)	37(3)	-2(2)	-10(2)	6(2)
C(4)	38(3)	59(3)	46(4)	1(3)	6(2)	-8(2)
C(5)	38(3)	53(3)	48(4)	4(3)	4(2)	1(2)
C(6)	57(4)	65(4)	51(4)	15(3)	10(3)	-2(3)
C(7)	73(5)	62(4)	99(6)	24(4)	12(4)	-12(3)
C(8)	46(3)	52(3)	45(4)	-1(3)	8(2)	-1(2)
C(9)	66(4)	68(4)	77(5)	11(4)	26(4)	-3(3)
C(10)	75(4)	57(4)	92(6)	8(4)	20(4)	-3(3)
C(11)	103(6)	59(4)	81(6)	10(4)	0(4)	8(4)

Table S5. Hydrogen coordinates (x 10^4) and isotropic displacement parameters (Å^2 x 10^3) for **2a**.

	x	y	z	U(eq)
H(2A)	7672	4400	4221	52
H(3A)	5850	3710	5463	52
H(4A)	5227	4604	1727	57
H(4B)	7213	4589	2187	57
H(5A)	6696	5874	3027	56
H(5B)	4759	5895	2495	56
H(6A)	7852	5916	1347	69
H(6B)	5885	6059	891	69
H(7A)	7354	7350	1081	116
H(7B)	5755	7326	1803	116
H(7C)	7720	7183	2258	116
H(8A)	1280	3294	4305	57
H(8B)	1954	3673	5368	57
H(9A)	3708	2509	5720	83
H(9B)	1708	2243	5517	83
H(10A)	4386	2032	4180	89
H(10B)	2371	1844	3880	89
H(11A)	3868	611	4264	122
H(11B)	4442	943	5366	122
H(11C)	2420	755	5065	122

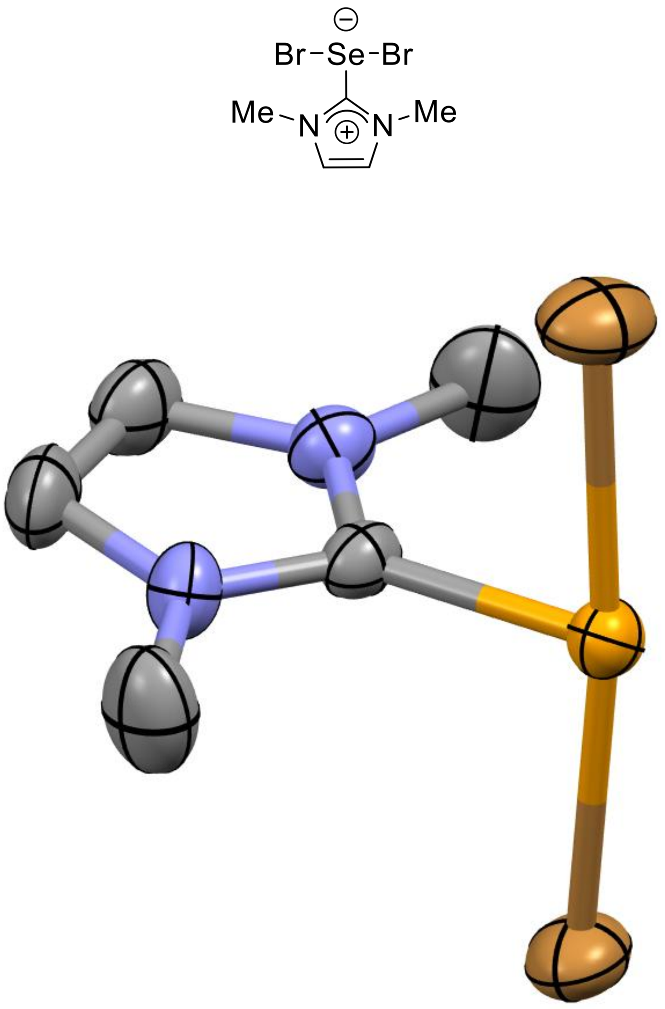

Figure S11. X-ray crystallographic structure of **2b** (CCDC 2357877).

Table S6. Crystal data and structure refinement for **2b**.

Identification code	hjx5seNBS	
Empirical formula	C5 H8 Br2 N2 Se	
Formula weight	334.91	
Temperature	296(2) K	
Wavelength	0.71073 Å	
Crystal system	Monoclinic	
Space group	$P2_1/c$	
Unit cell dimensions	a = 7.9911(9) Å	α= 90°.
	b = 14.6180(17) Å	β= 95.451(4)°.
	c = 16.697(2) Å	γ = 90°.
Volume	1941.6(4) $Å^3$	
Z	8	
Density (calculated)	2.292 Mg/m^3	
Absorption coefficient	12.036 mm^{-1}	
F(000)	1248	
Crystal size	0.400 x 0.300 x 0.200 mm^3	
Theta range for data collection	1.855 to 25.241°.	
Index ranges	-9<=h<=9, -17<=k<=17, -20<=l<=20	
Reflections collected	52681	
Independent reflections	3515 [R(int) = 0.1059]	
Completeness to theta = 25.241°	99.9 %	
Absorption correction	multi-scan	
Max. and min. transmission	0.7456 and 0.5578	
Refinement method	Full-matrix least-squares on F^2	
Data / restraints / parameters	3515 / 0 / 181	
Goodness-of-fit on F^2	1.021	
Final R indices [I>2sigma(I)]	R1 = 0.0359, wR2 = 0.0719	
R indices (all data)	R1 = 0.0580, wR2 = 0.0794	
Extinction coefficient	n/a	
Largest diff. peak and hole	1.007 and -0.666 e.$Å^{-3}$	

Table S7. Atomic coordinates (x 10^4) and equivalent isotropic displacement parameters ($Å^2$ x 10^3) for **2b**. U(eq) is defined as one-third of the trace of the orthogonalized U^{ij} tensor.

	x	y	z	U(eq)
Se(1)	6809(1)	7660(1)	6264(1)	42(1)
Se(2)	8169(1)	5566(1)	7098(1)	33(1)
Br(1)	8545(1)	8202(1)	5175(1)	69(1)
Br(2)	4979(1)	7138(1)	7417(1)	57(1)
Br(3)	10277(1)	6002(1)	8316(1)	52(1)
Br(4)	6298(1)	5002(1)	5883(1)	53(1)
N(1)	4901(5)	9329(3)	6298(3)	38(1)
N(2)	3987(6)	8473(3)	5310(3)	47(1)
N(3)	10963(5)	4956(3)	6254(2)	36(1)
N(4)	9957(5)	3874(3)	6941(3)	35(1)
C(1)	5147(6)	8534(3)	5937(3)	34(1)
C(2)	3590(7)	9768(4)	5892(4)	50(2)
C(3)	3039(8)	9251(5)	5280(4)	57(2)
C(4)	5846(8)	9649(4)	7033(4)	58(2)
C(5)	3813(10)	7706(5)	4737(4)	83(2)
C(6)	9821(6)	4761(3)	6767(3)	32(1)
C(7)	11810(7)	4174(4)	6108(3)	45(2)
C(8)	11188(7)	3499(4)	6534(3)	45(1)
C(9)	11287(8)	5863(4)	5930(4)	59(2)
C(10)	8931(8)	3387(4)	7490(4)	61(2)

Table S8. Bond lengths [Å] and angles [°] for **2b**.

Se(1)-C(1)	1.886(5)
Se(1)-Br(1)	2.5174(9)
Se(1)-Br(2)	2.6387(9)
Se(2)-C(6)	1.890(5)
Se(2)-Br(4)	2.5414(8)
Se(2)-Br(3)	2.5928(8)
N(1)-C(1)	1.331(6)
N(1)-C(2)	1.355(7)
N(1)-C(4)	1.456(7)
N(2)-C(1)	1.335(6)
N(2)-C(3)	1.365(7)
N(2)-C(5)	1.472(8)
N(3)-C(6)	1.341(6)
N(3)-C(7)	1.363(7)
N(3)-C(9)	1.463(7)
N(4)-C(6)	1.330(6)
N(4)-C(8)	1.364(6)
N(4)-C(10)	1.470(7)
C(2)-C(3)	1.313(8)
C(2)-H(2A)	0.9300
C(3)-H(3A)	0.9300
C(4)-H(4A)	0.9600
C(4)-H(4B)	0.9600
C(4)-H(4C)	0.9600
C(5)-H(5A)	0.9600
C(5)-H(5B)	0.9600
C(5)-H(5C)	0.9600
C(7)-C(8)	1.338(8)
C(7)-H(7A)	0.9300
C(8)-H(8A)	0.9300
C(9)-H(9A)	0.9600
C(9)-H(9B)	0.9600
C(9)-H(9C)	0.9600
C(10)-H(10A)	0.9600
C(10)-H(10B)	0.9600
C(10)-H(10C)	0.9600

C(1)-Se(1)-Br(1)	89.97(15)
C(1)-Se(1)-Br(2)	89.01(15)
Br(1)-Se(1)-Br(2)	178.47(3)
C(6)-Se(2)-Br(4)	86.50(15)
C(6)-Se(2)-Br(3)	87.83(15)
Br(4)-Se(2)-Br(3)	174.16(3)
C(1)-N(1)-C(2)	109.1(5)
C(1)-N(1)-C(4)	125.1(5)
C(2)-N(1)-C(4)	125.7(5)
C(1)-N(2)-C(3)	108.3(5)
C(1)-N(2)-C(5)	125.2(5)
C(3)-N(2)-C(5)	126.5(5)
C(6)-N(3)-C(7)	108.4(4)
C(6)-N(3)-C(9)	125.8(4)
C(7)-N(3)-C(9)	125.8(5)
C(6)-N(4)-C(8)	109.3(4)
C(6)-N(4)-C(10)	124.7(4)
C(8)-N(4)-C(10)	126.0(5)
N(1)-C(1)-N(2)	107.0(4)
N(1)-C(1)-Se(1)	126.2(4)
N(2)-C(1)-Se(1)	126.8(4)
C(3)-C(2)-N(1)	107.6(5)
C(3)-C(2)-H(2A)	126.2
N(1)-C(2)-H(2A)	126.2
C(2)-C(3)-N(2)	107.9(5)
C(2)-C(3)-H(3A)	126.0
N(2)-C(3)-H(3A)	126.0
N(1)-C(4)-H(4A)	109.5
N(1)-C(4)-H(4B)	109.5
H(4A)-C(4)-H(4B)	109.5
N(1)-C(4)-H(4C)	109.5
H(4A)-C(4)-H(4C)	109.5
H(4B)-C(4)-H(4C)	109.5
N(2)-C(5)-H(5A)	109.5
N(2)-C(5)-H(5B)	109.5
H(5A)-C(5)-H(5B)	109.5
N(2)-C(5)-H(5C)	109.5

H(5A)-C(5)-H(5C)	109.5
H(5B)-C(5)-H(5C)	109.5
N(4)-C(6)-N(3)	107.5(4)
N(4)-C(6)-Se(2)	126.0(4)
N(3)-C(6)-Se(2)	126.2(4)
C(8)-C(7)-N(3)	107.9(5)
C(8)-C(7)-H(7A)	126.0
N(3)-C(7)-H(7A)	126.0
C(7)-C(8)-N(4)	106.9(5)
C(7)-C(8)-H(8A)	126.6
N(4)-C(8)-H(8A)	126.6
N(3)-C(9)-H(9A)	109.5
N(3)-C(9)-H(9B)	109.5
H(9A)-C(9)-H(9B)	109.5
N(3)-C(9)-H(9C)	109.5
H(9A)-C(9)-H(9C)	109.5
H(9B)-C(9)-H(9C)	109.5
N(4)-C(10)-H(10A)	109.5
N(4)-C(10)-H(10B)	109.5
H(10A)-C(10)-H(10B)	109.5
N(4)-C(10)-H(10C)	109.5
H(10A)-C(10)-H(10C)	109.5
H(10B)-C(10)-H(10C)	109.5

Symmetry transformations used to generate equivalent atoms:

Table S9. Anisotropic displacement parameters (Å^2 x 10^3) for **2b**. The anisotropic displacement factor exponent takes the form: $-2\pi^2[h^2 a^{*2}U^{11} + ... + 2 h k a^* b^* U^{12}]$

	U^{11}	U^{22}	U^{33}	U^{23}	U^{13}	U^{12}
Se(1)	48(1)	36(1)	44(1)	6(1)	9(1)	13(1)
Se(2)	32(1)	35(1)	31(1)	-1(1)	4(1)	5(1)
Br(1)	74(1)	72(1)	67(1)	19(1)	38(1)	18(1)
Br(2)	59(1)	48(1)	67(1)	16(1)	28(1)	10(1)
Br(3)	53(1)	57(1)	43(1)	-15(1)	-7(1)	2(1)
Br(4)	40(1)	78(1)	41(1)	-11(1)	-6(1)	0(1)
N(1)	36(2)	36(3)	42(3)	2(2)	6(2)	6(2)
N(2)	51(3)	55(3)	33(3)	2(2)	-5(2)	-1(3)
N(3)	35(2)	39(3)	34(3)	-1(2)	8(2)	0(2)
N(4)	34(2)	27(2)	45(3)	-1(2)	7(2)	2(2)
C(1)	34(3)	35(3)	33(3)	6(2)	6(2)	2(2)
C(2)	47(4)	51(4)	51(4)	17(3)	5(3)	21(3)
C(3)	45(4)	81(5)	44(4)	20(4)	-6(3)	19(4)
C(4)	58(4)	45(4)	70(5)	-13(3)	6(3)	1(3)
C(5)	98(6)	91(6)	54(5)	-20(4)	-11(4)	-5(5)
C(6)	27(3)	37(3)	31(3)	-6(2)	4(2)	2(2)
C(7)	37(3)	54(4)	46(4)	-8(3)	10(3)	10(3)
C(8)	42(3)	38(3)	54(4)	-7(3)	5(3)	14(3)
C(9)	68(4)	53(4)	61(4)	13(3)	32(4)	-2(3)
C(10)	65(4)	45(4)	76(5)	19(3)	23(4)	5(3)

Table S10. Hydrogen coordinates (x 10^4) and isotropic displacement parameters ($Å^2$ x 10^3) for **2b**.

	x	y	z	U(eq)
H(2A)	3158	10334	6024	60
H(3A)	2159	9391	4894	69
H(4A)	5436	10240	7173	86
H(4B)	5710	9226	7461	86
H(4C)	7015	9694	6949	86
H(5A)	2889	7821	4340	124
H(5B)	4829	7643	4478	124
H(5C)	3609	7151	5021	124
H(7A)	12668	4118	5772	54
H(8A)	11531	2891	6549	54
H(9A)	12164	5820	5577	88
H(9B)	10282	6090	5637	88
H(9C)	11628	6273	6364	88
H(10A)	9257	2755	7520	91
H(10B)	9100	3656	8016	91
H(10C)	7765	3432	7293	91

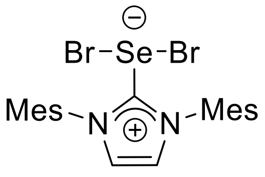

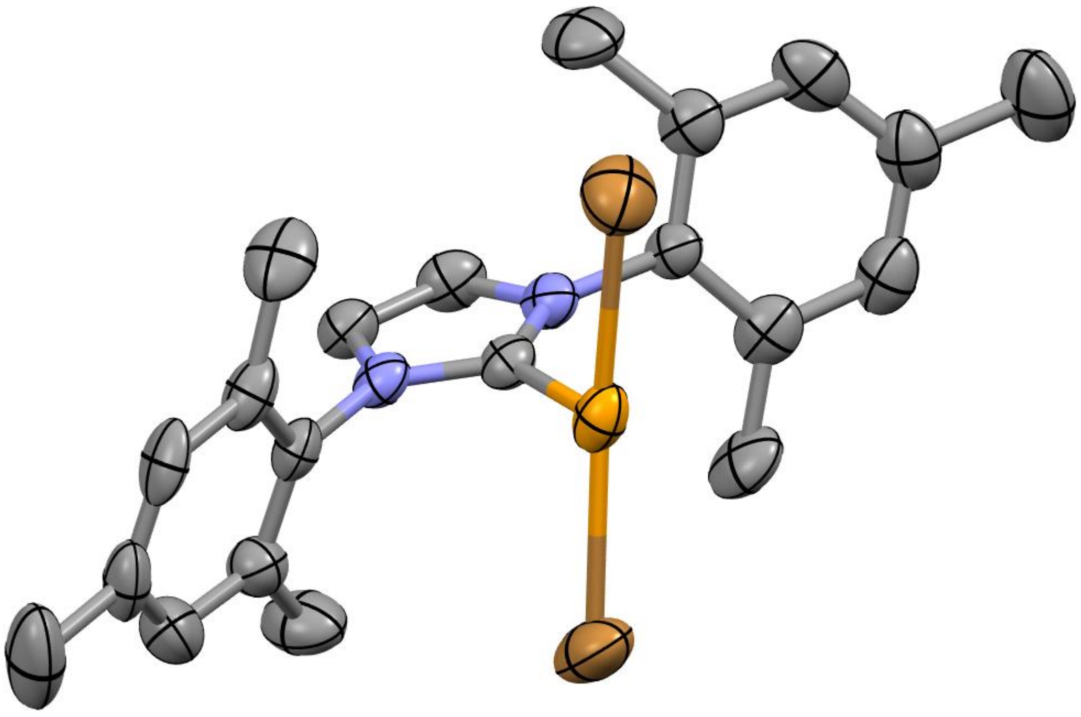

Figure S12. X-ray crystallographic structure of **2c** (CCDC 2358227).

Table S11. Crystal data and structure refinement for **2c**.

Identification code	semesbr	
Empirical formula	C21 H24 Br2 N2 Se	
Formula weight	543.20	
Temperature	296(2) K	
Wavelength	0.71073 Å	
Crystal system	Orthorhombic	
Space group	$P2_12_12_1$	
Unit cell dimensions	a = 9.3631(7) Å	α= 90°.
	b = 15.1437(12) Å	β= 90°.
	c = 15.3827(12) Å	γ = 90°.
Volume	2181.1(3) $Å^3$	
Z	4	
Density (calculated)	1.654 Mg/m^3	
Absorption coefficient	5.393 mm^{-1}	
F(000)	1072	
Crystal size	0.400 x 0.300 x 0.300 mm^3	
Theta range for data collection	1.887 to 25.239°.	
Index ranges	-11<=h<=11, -18<=k<=18, -18<=l<=18	
Reflections collected	24172	
Independent reflections	3950 [R(int) = 0.0565]	
Completeness to theta = 25.239°	99.9 %	
Absorption correction	multi-scan	
Max. and min. transmission	0.7456 and 0.2611	
Refinement method	Full-matrix least-squares on F^2	
Data / restraints / parameters	3950 / 0 / 235	
Goodness-of-fit on F^2	1.078	
Final R indices [I>2sigma(I)]	R1 = 0.0419, wR2 = 0.1071	
R indices (all data)	R1 = 0.0628, wR2 = 0.1270	
Absolute structure parameter	0.029(8)	
Extinction coefficient	n/a	
Largest diff. peak and hole	0.585 and -0.652 e.$Å^{-3}$	

Table S12. Atomic coordinates (x 10^4) and equivalent isotropic displacement parameters (Å^2 x 10^3) for **2c**. U(eq) is defined as one third of the trace of the orthogonalized U^{ij} tensor.

	x	y	z	U(eq)
Se(1)	4775(1)	6381(1)	2751(1)	47(1)
Br(1)	6229(2)	5844(1)	1417(1)	76(1)
Br(2)	3085(1)	6961(1)	3940(1)	75(1)
N(1)	7492(8)	6853(4)	3529(4)	37(2)
N(2)	6610(8)	5659(4)	4127(4)	39(2)
C(1)	6360(9)	6282(5)	3516(5)	36(2)
C(2)	8428(10)	6566(5)	4160(5)	43(2)
C(3)	7883(10)	5835(6)	4521(5)	47(2)
C(4)	7776(9)	7584(5)	2955(5)	39(2)
C(5)	8836(10)	7467(6)	2330(6)	48(2)
C(6)	9142(11)	8186(7)	1804(6)	58(3)
C(7)	8439(12)	8978(6)	1892(7)	55(3)
C(8)	7433(11)	9072(5)	2543(6)	52(3)
C(9)	7076(10)	8387(5)	3089(6)	47(2)
C(10)	9631(13)	6608(6)	2227(7)	71(3)
C(11)	8812(15)	9726(8)	1287(8)	93(4)
C(12)	6077(11)	8531(6)	3845(7)	64(3)
C(13)	5626(10)	4985(5)	4403(5)	38(2)
C(14)	5561(11)	4193(6)	3928(6)	49(2)
C(15)	4609(12)	3550(6)	4240(6)	56(3)
C(16)	3796(11)	3680(6)	4971(6)	59(3)
C(17)	3934(12)	4464(7)	5427(6)	60(3)
C(18)	4872(11)	5119(5)	5166(5)	50(2)
C(19)	6464(13)	4028(6)	3165(6)	63(3)
C(20)	2715(15)	3010(8)	5259(8)	88(4)
C(21)	5090(13)	5944(7)	5708(6)	69(3)

Table S13. Bond lengths [Å] and angles [°] for **2c**.

Se(1)-C(1)	1.899(8)
Se(1)-Br(2)	2.5731(16)
Se(1)-Br(1)	2.5936(15)
N(1)-C(1)	1.368(10)
N(1)-C(2)	1.379(10)
N(1)-C(4)	1.440(10)
N(2)-C(1)	1.352(10)
N(2)-C(3)	1.363(12)
N(2)-C(13)	1.440(11)
C(2)-C(3)	1.340(12)
C(2)-H(2A)	0.9300
C(3)-H(3A)	0.9300
C(4)-C(5)	1.394(12)
C(4)-C(9)	1.396(11)
C(5)-C(6)	1.387(12)
C(5)-C(10)	1.507(13)
C(6)-C(7)	1.374(14)
C(6)-H(6A)	0.9300
C(7)-C(8)	1.383(14)
C(7)-C(11)	1.507(13)
C(8)-C(9)	1.375(13)
C(8)-H(8A)	0.9300
C(9)-C(12)	1.509(14)
C(10)-H(10A)	0.9600
C(10)-H(10B)	0.9600
C(10)-H(10C)	0.9600
C(11)-H(11A)	0.9600
C(11)-H(11B)	0.9600
C(11)-H(11C)	0.9600
C(12)-H(12A)	0.9600
C(12)-H(12B)	0.9600
C(12)-H(12C)	0.9600
C(13)-C(18)	1.384(12)
C(13)-C(14)	1.405(11)
C(14)-C(15)	1.405(14)
C(14)-C(19)	1.469(14)

C(15)-C(16)	1.371(15)
C(15)-H(15A)	0.9300
C(16)-C(17)	1.384(14)
C(16)-C(20)	1.501(14)
C(17)-C(18)	1.385(14)
C(17)-H(17A)	0.9300
C(18)-C(21)	1.517(12)
C(19)-H(19A)	0.9600
C(19)-H(19B)	0.9600
C(19)-H(19C)	0.9600
C(20)-H(20A)	0.9600
C(20)-H(20B)	0.9600
C(20)-H(20C)	0.9600
C(21)-H(21A)	0.9600
C(21)-H(21B)	0.9600
C(21)-H(21C)	0.9600
C(1)-Se(1)-Br(2)	93.9(2)
C(1)-Se(1)-Br(1)	93.2(2)
Br(2)-Se(1)-Br(1)	172.88(6)
C(1)-N(1)-C(2)	107.7(6)
C(1)-N(1)-C(4)	128.3(7)
C(2)-N(1)-C(4)	123.8(7)
C(1)-N(2)-C(3)	108.9(7)
C(1)-N(2)-C(13)	126.1(7)
C(3)-N(2)-C(13)	124.5(7)
N(2)-C(1)-N(1)	107.3(7)
N(2)-C(1)-Se(1)	128.4(6)
N(1)-C(1)-Se(1)	124.4(6)
C(3)-C(2)-N(1)	108.1(8)
C(3)-C(2)-H(2A)	126.0
N(1)-C(2)-H(2A)	126.0
C(2)-C(3)-N(2)	108.1(7)
C(2)-C(3)-H(3A)	126.0
N(2)-C(3)-H(3A)	126.0
C(5)-C(4)-C(9)	123.1(8)
C(5)-C(4)-N(1)	117.1(7)
C(9)-C(4)-N(1)	119.5(7)

C(6)-C(5)-C(4)	116.7(8)
C(6)-C(5)-C(10)	121.0(9)
C(4)-C(5)-C(10)	122.3(8)
C(7)-C(6)-C(5)	121.9(9)
C(7)-C(6)-H(6A)	119.0
C(5)-C(6)-H(6A)	119.0
C(6)-C(7)-C(8)	119.2(9)
C(6)-C(7)-C(11)	119.0(11)
C(8)-C(7)-C(11)	121.9(10)
C(7)-C(8)-C(9)	122.0(8)
C(7)-C(8)-H(8A)	119.0
C(9)-C(8)-H(8A)	119.0
C(8)-C(9)-C(4)	116.9(9)
C(8)-C(9)-C(12)	120.8(8)
C(4)-C(9)-C(12)	122.0(8)
C(5)-C(10)-H(10A)	109.5
C(5)-C(10)-H(10B)	109.5
H(10A)-C(10)-H(10B)	109.5
C(5)-C(10)-H(10C)	109.5
H(10A)-C(10)-H(10C)	109.5
H(10B)-C(10)-H(10C)	109.5
C(7)-C(11)-H(11A)	109.5
C(7)-C(11)-H(11B)	109.5
H(11A)-C(11)-H(11B)	109.5
C(7)-C(11)-H(11C)	109.5
H(11A)-C(11)-H(11C)	109.5
H(11B)-C(11)-H(11C)	109.5
C(9)-C(12)-H(12A)	109.5
C(9)-C(12)-H(12B)	109.5
H(12A)-C(12)-H(12B)	109.5
C(9)-C(12)-H(12C)	109.5
H(12A)-C(12)-H(12C)	109.5
H(12B)-C(12)-H(12C)	109.5
C(18)-C(13)-C(14)	122.9(8)
C(18)-C(13)-N(2)	118.2(7)
C(14)-C(13)-N(2)	118.7(7)
C(13)-C(14)-C(15)	116.2(9)
C(13)-C(14)-C(19)	122.4(9)

C(15)-C(14)-C(19)	121.4(9)
C(16)-C(15)-C(14)	122.1(9)
C(16)-C(15)-H(15A)	118.9
C(14)-C(15)-H(15A)	118.9
C(15)-C(16)-C(17)	119.2(9)
C(15)-C(16)-C(20)	121.3(11)
C(17)-C(16)-C(20)	119.4(11)
C(18)-C(17)-C(16)	121.7(10)
C(18)-C(17)-H(17A)	119.2
C(16)-C(17)-H(17A)	119.2
C(17)-C(18)-C(13)	117.7(8)
C(17)-C(18)-C(21)	121.1(9)
C(13)-C(18)-C(21)	121.2(8)
C(14)-C(19)-H(19A)	109.5
C(14)-C(19)-H(19B)	109.5
H(19A)-C(19)-H(19B)	109.5
C(14)-C(19)-H(19C)	109.5
H(19A)-C(19)-H(19C)	109.5
H(19B)-C(19)-H(19C)	109.5
C(16)-C(20)-H(20A)	109.5
C(16)-C(20)-H(20B)	109.5
H(20A)-C(20)-H(20B)	109.5
C(16)-C(20)-H(20C)	109.5
H(20A)-C(20)-H(20C)	109.5
H(20B)-C(20)-H(20C)	109.5
C(18)-C(21)-H(21A)	109.5
C(18)-C(21)-H(21B)	109.5
H(21A)-C(21)-H(21B)	109.5
C(18)-C(21)-H(21C)	109.5
H(21A)-C(21)-H(21C)	109.5
H(21B)-C(21)-H(21C)	109.5

Symmetry transformations used to generate equivalent atoms:

Table S14. Anisotropic displacement parameters ($Å^2 \times 10^3$) for **2c**. The anisotropic displacement factor exponent takes the form: $-2\pi^2[h^2 a^{*2}U^{11} + ... + 2 h k a^* b^* U^{12}]$

	U^{11}	U^{22}	U^{33}	U^{23}	U^{13}	U^{12}
Se(1)	61(1)	32(1)	48(1)	2(1)	-14(1)	-4(1)
Br(1)	124(1)	57(1)	49(1)	-12(1)	3(1)	-6(1)
Br(2)	62(1)	72(1)	90(1)	-9(1)	6(1)	1(1)
N(1)	50(4)	25(3)	37(3)	-4(3)	-3(3)	4(3)
N(2)	54(5)	25(3)	37(4)	0(3)	2(3)	2(3)
C(1)	40(5)	28(4)	40(4)	-4(3)	0(4)	6(4)
C(2)	51(6)	38(5)	40(4)	-2(3)	-6(4)	2(4)
C(3)	55(6)	45(5)	41(4)	3(4)	-9(4)	9(5)
C(4)	45(5)	28(4)	45(5)	-5(3)	-8(4)	-4(4)
C(5)	55(6)	44(5)	44(5)	-1(4)	3(5)	-2(4)
C(6)	60(7)	60(6)	56(5)	3(4)	7(5)	-13(5)
C(7)	67(7)	36(5)	61(6)	11(4)	-20(6)	-21(5)
C(8)	62(6)	16(4)	78(7)	2(4)	-25(6)	0(4)
C(9)	53(6)	28(4)	61(5)	-7(4)	-18(5)	5(4)
C(10)	95(9)	52(6)	66(6)	-3(5)	24(7)	16(5)
C(11)	112(11)	67(7)	100(9)	30(7)	-33(9)	-45(8)
C(12)	60(7)	42(5)	91(7)	-12(5)	-5(6)	19(5)
C(13)	53(6)	25(4)	35(4)	4(3)	-1(4)	4(4)
C(14)	61(6)	37(4)	48(4)	8(4)	-15(5)	10(4)
C(15)	73(7)	37(5)	58(6)	7(4)	-21(5)	0(5)
C(16)	61(6)	54(6)	62(6)	21(5)	-14(5)	-8(5)
C(17)	68(7)	60(6)	53(5)	24(5)	-5(5)	6(5)
C(18)	65(6)	34(4)	50(5)	9(4)	-4(5)	6(4)
C(19)	98(9)	30(5)	62(6)	-11(4)	-5(6)	8(5)
C(20)	104(10)	76(8)	84(8)	37(7)	-9(8)	-28(7)
C(21)	90(9)	56(6)	61(6)	-15(5)	20(6)	11(6)

Table S15. Hydrogen coordinates (x 10^4) and isotropic displacement parameters (Å^2 x 10^3) for **2c**.

	x	y	z	U(eq)
H(2A)	9288	6834	4310	51
H(3A)	8299	5506	4964	56
H(6A)	9843	8131	1379	70
H(8A)	6984	9615	2615	62
H(10A)	9281	6188	2643	107
H(10B)	9487	6384	1650	107
H(10C)	10632	6706	2322	107
H(11A)	8238	10232	1426	140
H(11B)	9804	9873	1353	140
H(11C)	8632	9550	698	140
H(12A)	5949	7985	4153	96
H(12B)	6476	8965	4230	96
H(12C)	5170	8735	3634	96
H(15A)	4526	3018	3941	68
H(17A)	3381	4552	5922	72
H(19A)	6259	3453	2935	95
H(19B)	6274	4467	2729	95
H(19C)	7451	4057	3331	95
H(20A)	2253	3214	5779	132
H(20B)	2015	2930	4810	132
H(20C)	3184	2459	5373	132
H(21A)	5774	6321	5428	103
H(21B)	4198	6251	5766	103
H(21C)	5437	5782	6273	103

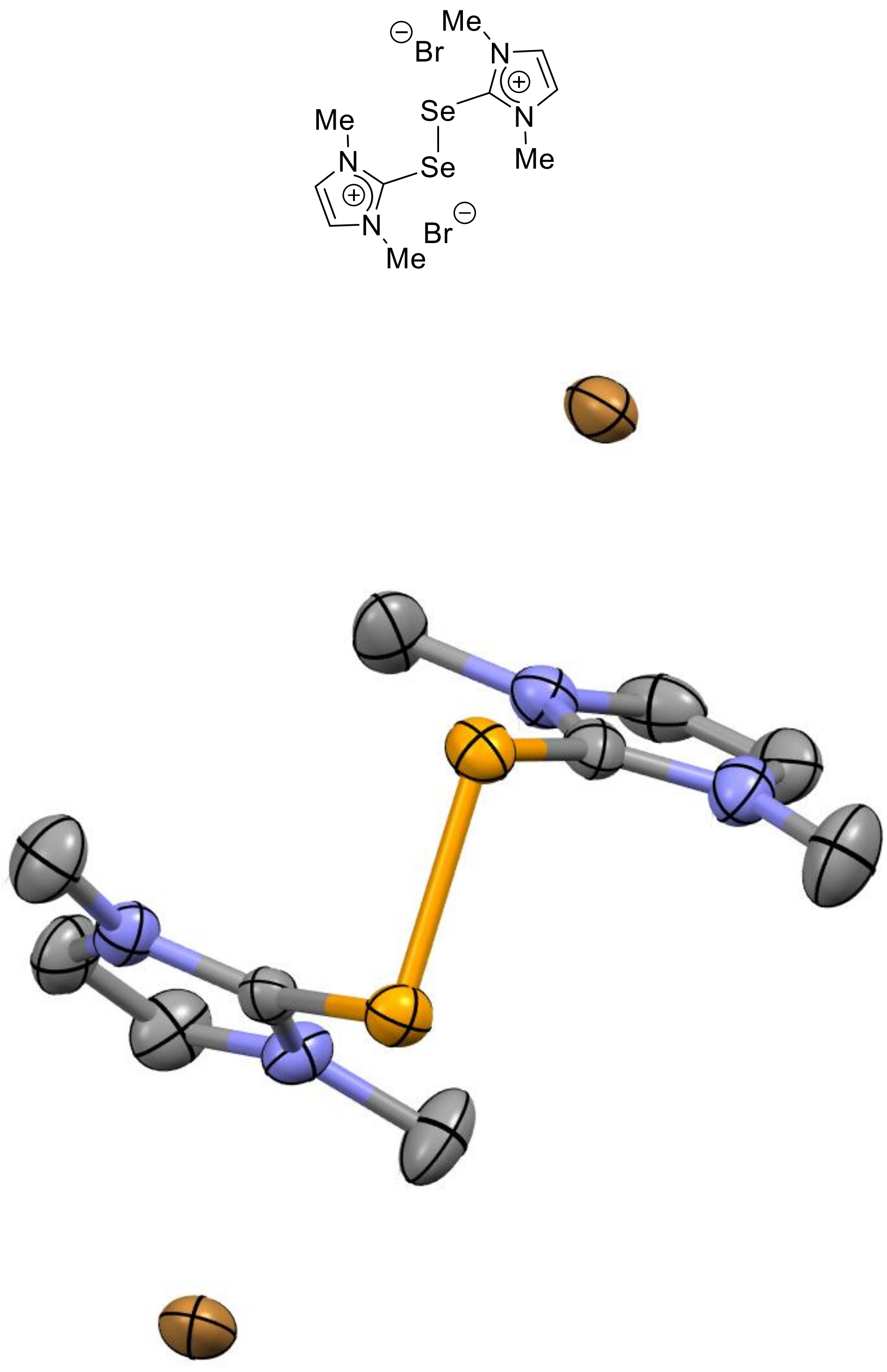

Figure S13. X-ray crystallographic structure of **5** (CCDC 2358232).

Table S16. Crystal data and structure refinement for **5**.

Identification code	Bisimidazolium	
Empirical formula	C10 H16 Br2 N4 Se2	
Formula weight	510.01	
Temperature	297(2) K	
Wavelength	0.71073 Å	
Crystal system	Monoclinic	
Space group	$P2_1$	
Unit cell dimensions	a = 7.8518(9) Å	α= 90°.
	b = 11.2673(13) Å	β= 106.332(3)°.
	c = 9.8026(9) Å	γ = 90°.
Volume	832.23(16) Å^3	
Z	2	
Density (calculated)	2.035 Mg/m^3	
Absorption coefficient	9.234 mm^{-1}	
F(000)	484	
Crystal size	0.400 x 0.300 x 0.300 mm^3	
Theta range for data collection	2.950 to 27.960°.	
Index ranges	-10<=h<=10, -14<=k<=14, -12<=l<=11	
Reflections collected	10011	
Independent reflections	3938 [R(int) = 0.0299]	
Completeness to theta = 25.242°	99.1 %	
Absorption correction	multi-scan	
Max. and min. transmission	0.7456 and 0.4339	
Refinement method	Full-matrix least-squares on F^2	
Data / restraints / parameters	3938 / 1 / 167	
Goodness-of-fit on F^2	1.000	
Final R indices [I>2sigma(I)]	R1 = 0.0228, wR2 = 0.0478	
R indices (all data)	R1 = 0.0249, wR2 = 0.0483	
Absolute structure parameter	0.018(9)	
Extinction coefficient	n/a	
Largest diff. peak and hole	0.346 and -0.502 e.Å^{-3}	

Table S17. Atomic coordinates (x 10^4) and equivalent isotropic displacement parameters (Å^2 x 10^3) for **5**. U(eq) is defined as one third of the trace of the orthogonalized U^{ij} tensor.

	x	y	z	U(eq)
N(1)	5354(5)	5051(3)	228(4)	33(1)
N(2)	8068(4)	5503(3)	1320(4)	35(1)
N(3)	6586(5)	6785(3)	4332(4)	35(1)
N(4)	8037(5)	5610(4)	5991(4)	40(1)
Br(1)	8422(1)	2597(1)	85(1)	51(1)
Br(2)	2987(1)	5701(1)	5839(1)	45(1)
Se(1)	6873(1)	3506(1)	2628(1)	33(1)
Se(2)	5109(1)	4435(1)	3977(1)	35(1)
C(1)	6737(5)	4777(3)	1340(4)	29(1)
C(10)	5238(7)	7254(5)	3093(5)	52(1)
C(2)	5847(7)	5961(4)	-510(5)	46(1)
C(3)	7535(7)	6242(4)	159(5)	46(1)
C(4)	9857(6)	5479(6)	2313(6)	58(1)
C(5)	3633(6)	4488(5)	-160(6)	51(1)
C(6)	6685(5)	5664(4)	4802(4)	31(1)
C(7)	7904(6)	7430(4)	5236(6)	47(1)
C(8)	8804(7)	6700(5)	6260(6)	48(1)
C(9)	8630(8)	4547(6)	6852(6)	67(2)

Table S18. Bond lengths [Å] and angles [°] for **5**.

N(1)-C(1)	1.341(5)
N(1)-C(2)	1.373(6)
N(1)-C(5)	1.443(6)
N(2)-C(1)	1.332(5)
N(2)-C(3)	1.377(6)
N(2)-C(4)	1.467(6)
N(3)-C(6)	1.339(5)
N(3)-C(7)	1.367(6)
N(3)-C(10)	1.467(6)
N(4)-C(6)	1.339(5)
N(4)-C(8)	1.361(6)
N(4)-C(9)	1.464(7)
Se(1)-C(1)	1.892(4)
Se(1)-Se(2)	2.4073(6)
Se(2)-C(6)	1.882(4)
C(10)-H(10A)	0.9600
C(10)-H(10B)	0.9600
C(10)-H(10C)	0.9600
C(2)-C(3)	1.341(7)
C(2)-H(2)	0.9300
C(3)-H(3)	0.9300
C(4)-H(4A)	0.9600
C(4)-H(4B)	0.9600
C(4)-H(4C)	0.9600
C(5)-H(5A)	0.9600
C(5)-H(5B)	0.9600
C(5)-H(5C)	0.9600
C(7)-C(8)	1.336(7)
C(7)-H(7)	0.9300
C(8)-H(8)	0.9300
C(9)-H(9A)	0.9600
C(9)-H(9B)	0.9600
C(9)-H(9C)	0.9600
C(1)-N(1)-C(2)	108.5(4)
C(1)-N(1)-C(5)	126.7(4)

C(2)-N(1)-C(5)	124.8(4)
C(1)-N(2)-C(3)	109.0(4)
C(1)-N(2)-C(4)	126.0(4)
C(3)-N(2)-C(4)	124.9(4)
C(6)-N(3)-C(7)	108.6(4)
C(6)-N(3)-C(10)	125.7(4)
C(7)-N(3)-C(10)	125.7(4)
C(6)-N(4)-C(8)	109.0(4)
C(6)-N(4)-C(9)	125.8(4)
C(8)-N(4)-C(9)	125.1(4)
C(1)-Se(1)-Se(2)	96.00(11)
C(6)-Se(2)-Se(1)	98.38(12)
N(2)-C(1)-N(1)	107.8(3)
N(2)-C(1)-Se(1)	125.3(3)
N(1)-C(1)-Se(1)	126.7(3)
N(3)-C(10)-H(10A)	109.5
N(3)-C(10)-H(10B)	109.5
H(10A)-C(10)-H(10B)	109.5
N(3)-C(10)-H(10C)	109.5
H(10A)-C(10)-H(10C)	109.5
H(10B)-C(10)-H(10C)	109.5
C(3)-C(2)-N(1)	107.8(4)
C(3)-C(2)-H(2)	126.1
N(1)-C(2)-H(2)	126.1
C(2)-C(3)-N(2)	106.9(4)
C(2)-C(3)-H(3)	126.5
N(2)-C(3)-H(3)	126.5
N(2)-C(4)-H(4A)	109.5
N(2)-C(4)-H(4B)	109.5
H(4A)-C(4)-H(4B)	109.5
N(2)-C(4)-H(4C)	109.5
H(4A)-C(4)-H(4C)	109.5
H(4B)-C(4)-H(4C)	109.5
N(1)-C(5)-H(5A)	109.5
N(1)-C(5)-H(5B)	109.5
H(5A)-C(5)-H(5B)	109.5
N(1)-C(5)-H(5C)	109.5
H(5A)-C(5)-H(5C)	109.5

H(5B)-C(5)-H(5C)	109.5
N(3)-C(6)-N(4)	107.3(4)
N(3)-C(6)-Se(2)	125.4(3)
N(4)-C(6)-Se(2)	127.2(3)
C(8)-C(7)-N(3)	107.7(4)
C(8)-C(7)-H(7)	126.2
N(3)-C(7)-H(7)	126.2
C(7)-C(8)-N(4)	107.4(4)
C(7)-C(8)-H(8)	126.3
N(4)-C(8)-H(8)	126.3
N(4)-C(9)-H(9A)	109.5
N(4)-C(9)-H(9B)	109.5
H(9A)-C(9)-H(9B)	109.5
N(4)-C(9)-H(9C)	109.5
H(9A)-C(9)-H(9C)	109.5
H(9B)-C(9)-H(9C)	109.5

Symmetry transformations used to generate equivalent atoms:

Table S19. Anisotropic displacement parameters ($Å^2 \times 10^3$) for **5**. The anisotropic displacement factor exponent takes the form: $-2\pi^2[h^2 a^{*2}U^{11} + ... + 2 h k a^* b^* U^{12}]$

	U^{11}	U^{22}	U^{33}	U^{23}	U^{13}	U^{12}
N(1)	36(2)	27(2)	35(2)	0(1)	8(2)	3(2)
N(2)	32(2)	33(2)	40(2)	6(2)	11(2)	2(2)
N(3)	37(2)	32(2)	37(2)	-3(2)	13(2)	1(2)
N(4)	34(2)	46(2)	38(2)	4(2)	8(1)	1(2)
Br(1)	54(1)	55(1)	44(1)	-6(1)	16(1)	18(1)
Br(2)	43(1)	52(1)	42(1)	-13(1)	15(1)	2(1)
Se(1)	38(1)	27(1)	36(1)	4(1)	13(1)	3(1)
Se(2)	35(1)	36(1)	36(1)	-2(1)	14(1)	-6(1)
C(1)	33(2)	26(2)	30(2)	1(2)	10(2)	6(2)
C(10)	68(3)	39(3)	44(3)	5(2)	9(2)	14(2)
C(2)	51(3)	40(3)	44(2)	15(2)	7(2)	7(2)
C(3)	44(3)	38(2)	57(3)	15(2)	15(2)	-3(2)
C(4)	33(3)	69(4)	61(3)	21(3)	-2(2)	-7(2)
C(5)	39(2)	48(3)	58(3)	0(2)	2(2)	-8(2)
C(6)	30(2)	36(2)	29(2)	1(2)	10(2)	-1(2)
C(7)	44(3)	36(2)	66(3)	-16(2)	22(2)	-8(2)
C(8)	35(3)	51(3)	54(3)	-15(2)	6(2)	-7(2)
C(9)	58(3)	70(4)	61(3)	23(3)	-4(3)	0(3)

Table S20. Hydrogen coordinates (x 10^4) and isotropic displacement parameters ($Å^2$ x 10^3) for **5**.

	x	y	z	U(eq)
H(10A)	5360	6880	2246	77
H(10B)	5393	8096	3031	77
H(10C)	4077	7094	3192	77
H(2)	5137	6319	-1328	55
H(3)	8216	6824	-111	55
H(4A)	10388	4718	2266	86
H(4B)	9784	5614	3262	86
H(4C)	10568	6088	2061	86
H(5A)	3185	4456	-1177	76
H(5B)	2832	4936	219	76
H(5C)	3739	3697	218	76
H(7)	8133	8233	5154	57
H(8)	9776	6900	7016	58
H(9A)	7887	4419	7467	101
H(9B)	9837	4651	7414	101
H(9C)	8554	3872	6239	101

Table S21. Torsion angles [°] for **5**.

C(3)-N(2)-C(1)-N(1)	-1.1(5)
C(4)-N(2)-C(1)-N(1)	-177.4(4)
C(3)-N(2)-C(1)-Se(1)	174.2(3)
C(4)-N(2)-C(1)-Se(1)	-2.1(6)
C(2)-N(1)-C(1)-N(2)	0.7(5)
C(5)-N(1)-C(1)-N(2)	180.0(4)
C(2)-N(1)-C(1)-Se(1)	-174.4(3)
C(5)-N(1)-C(1)-Se(1)	4.8(6)
Se(2)-Se(1)-C(1)-N(2)	108.3(3)
Se(2)-Se(1)-C(1)-N(1)	-77.3(3)
C(1)-N(1)-C(2)-C(3)	-0.1(5)
C(5)-N(1)-C(2)-C(3)	-179.4(4)
N(1)-C(2)-C(3)-N(2)	-0.5(5)
C(1)-N(2)-C(3)-C(2)	1.0(5)
C(4)-N(2)-C(3)-C(2)	177.3(5)
C(7)-N(3)-C(6)-N(4)	0.5(5)
C(10)-N(3)-C(6)-N(4)	-176.9(4)
C(7)-N(3)-C(6)-Se(2)	177.3(3)
C(10)-N(3)-C(6)-Se(2)	-0.1(6)
C(8)-N(4)-C(6)-N(3)	-0.7(5)
C(9)-N(4)-C(6)-N(3)	-179.4(5)
C(8)-N(4)-C(6)-Se(2)	-177.4(3)
C(9)-N(4)-C(6)-Se(2)	3.9(7)
Se(1)-Se(2)-C(6)-N(3)	98.8(3)
Se(1)-Se(2)-C(6)-N(4)	-85.1(4)
C(6)-N(3)-C(7)-C(8)	-0.1(5)
C(10)-N(3)-C(7)-C(8)	177.3(4)
N(3)-C(7)-C(8)-N(4)	-0.3(5)
C(6)-N(4)-C(8)-C(7)	0.6(5)
C(9)-N(4)-C(8)-C(7)	179.3(5)

Symmetry transformations used to generate equivalent atoms:

6. NMR spectra

^{1}H NMR

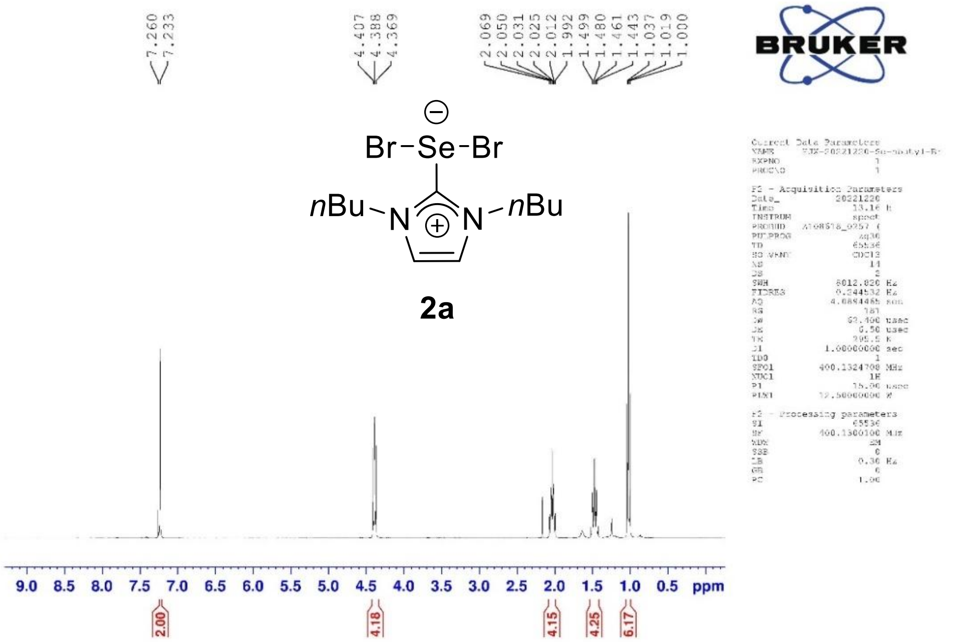

^{13}C NMR

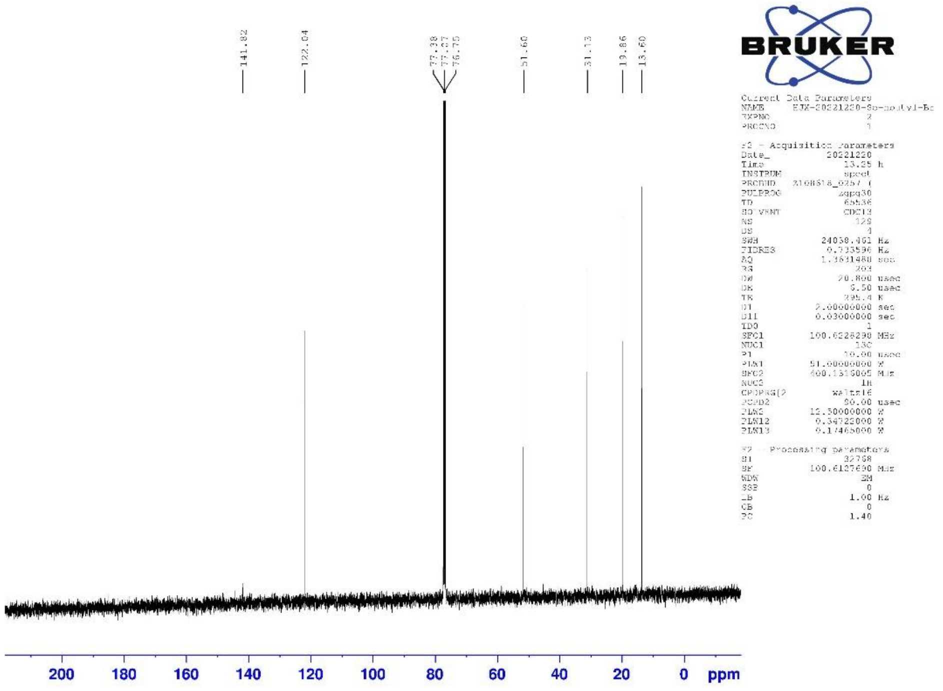

^{77}Se NMR

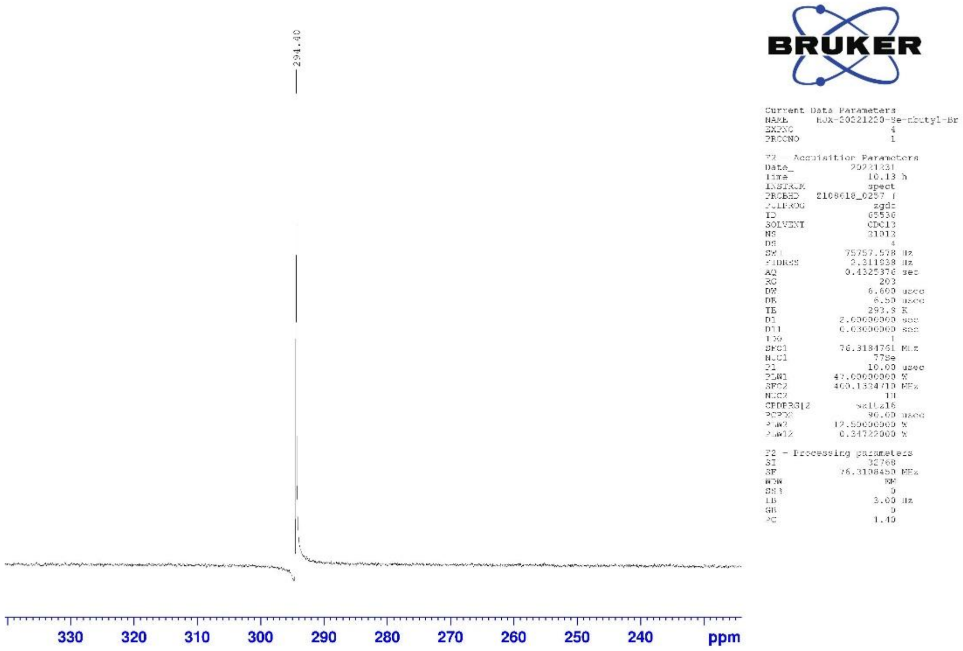

^{1}H NMR

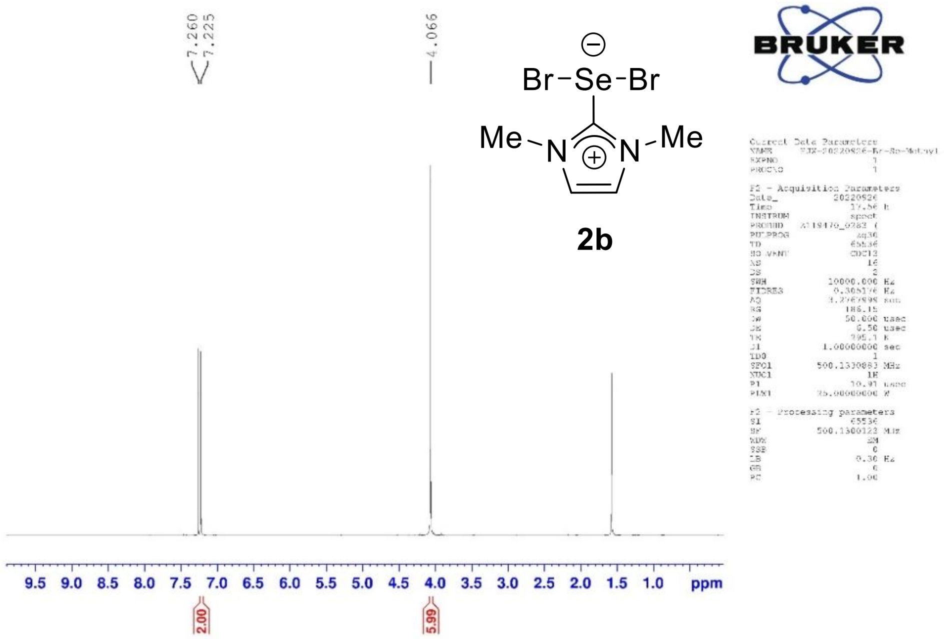

^{13}C NMR

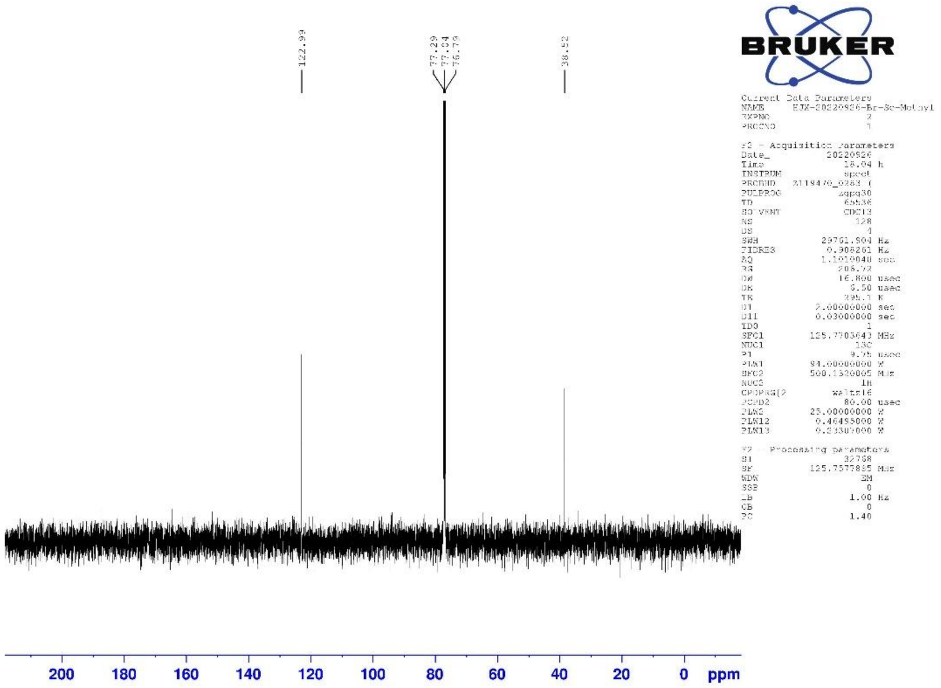

^{77}Se NMR

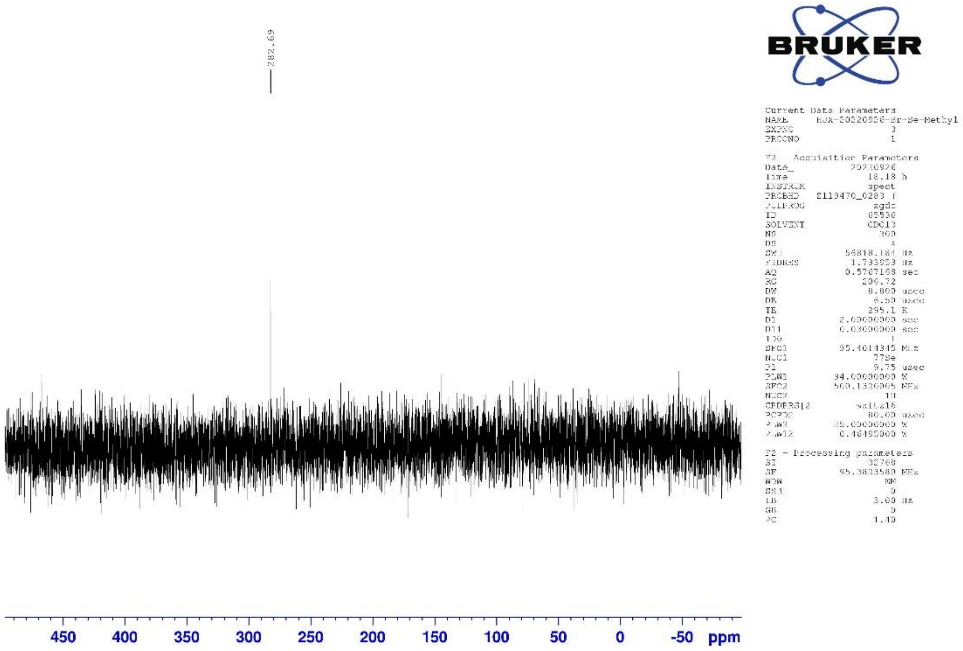
282.69
BRUKER
Current Data Parameters
NAME HJX-20220926-Br-Se-Methyl
EXPNO 3
PROCNO 1
F2 - Acquisition Parameters
Date_ 20220926
Time 18.18 h
INSTRUM spect
PROBHD Z119470_0283 (
PULPROG zgdc
TD 65536
SOLVENT CDCl3
NS 300
DS 4
SWH 56818.184 Hz
FIDRES 1.733953 Hz
AQ 0.5767168 sec
RG 206.72
DW 8.800 usec
DE 6.50 usec
TE 295.1 K
D1 2.00000000 sec
D11 0.03000000 sec
TD0 1
SFO1 95.4014345 MHz
NUC1 77Se
P1 9.75 usec
PLW1 94.00000000 W
SFO2 500.1320005 MHz
NUC2 1H
CPDPRG[2 waltz16
PCPD2 80.00 usec
PLW2 25.00000000 W
PLW12 0.46495000 W
F2 - Processing parameters
SI 32768
SF 95.3833580 MHz
WDW EM
SSB 0
LB 3.00 Hz
GB 0
PC 1.40
450
400
350
300
250
200
150
100
50
0
-50
ppm

^{1}H NMR

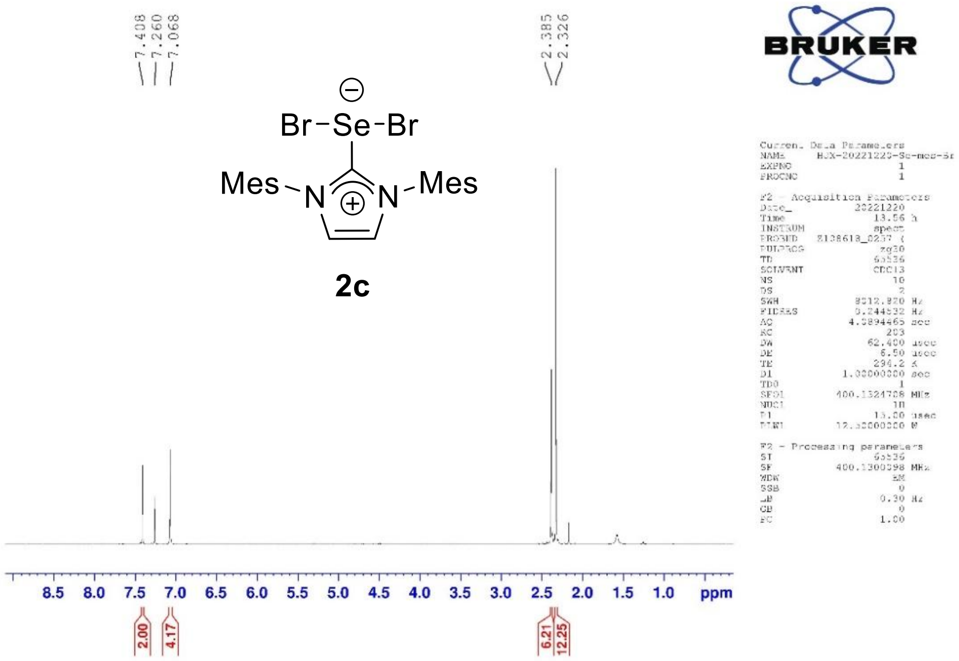

^{13}C NMR

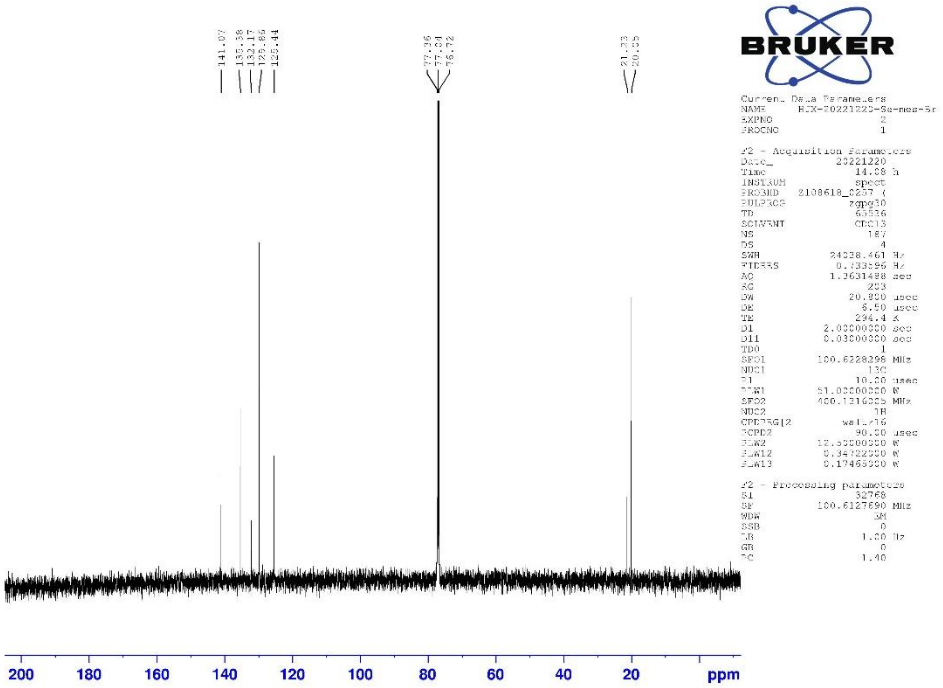

^{77}Se NMR

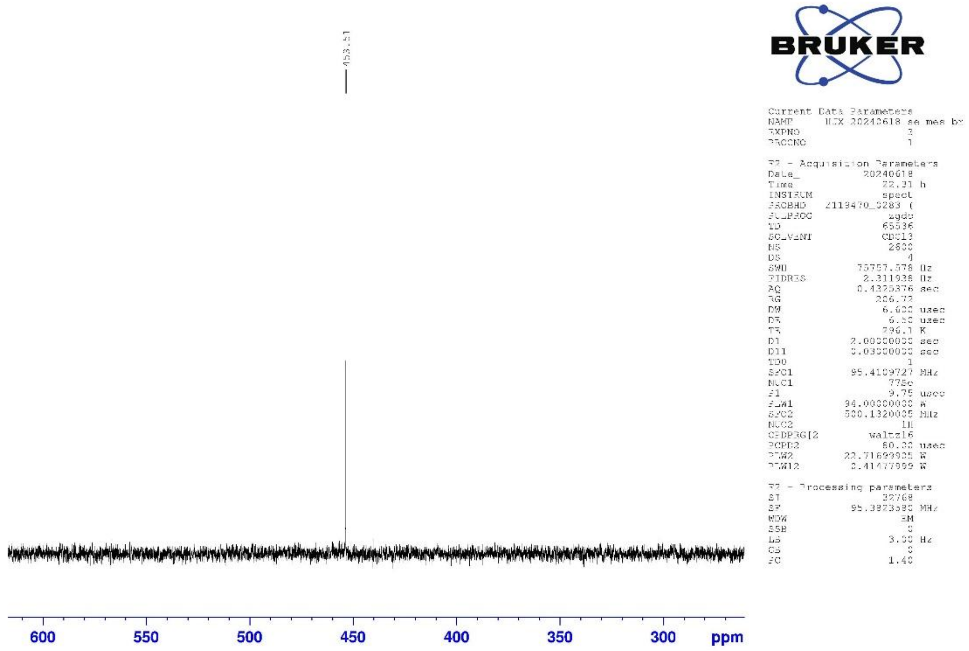

453.51
BRUKER
Current Data Parameters
EXPNO 3
PROCNO 1
F2 - Acquisition Parameters
Date_ 20240618
Time 22.31 h
INSTRUM spect
TD 65536
SOLVENT CDCl3
NS 2600
DS 4
SWH 75757.578 Hz
FIDRES 2.311938 Hz
AQ 0.4325376 sec
RG 206.72
DW 6.600 usec
TE 296.1 K
D1 2.00000000 sec
D11 0.03000000 sec
TD0 1
SFO1 95.4109727 MHz
NUC1 77Se
P1 9.75 usec
PLW1 94.00000000 W
SFO2 500.1320005 MHz
NUC2 1H
CPDPRG[2 waltz16
PCPD2 80.00 usec
PLW2 22.71699905 W
PLW12 0.41477999 W
F2 - Processing parameters
SI 32768
SF 95.3823580 MHz
WDW EM
SSB 0
LB 3.00 Hz
GB 0
PC 1.40
600
550
500
450
400
350
300
ppm

^{1}H NMR

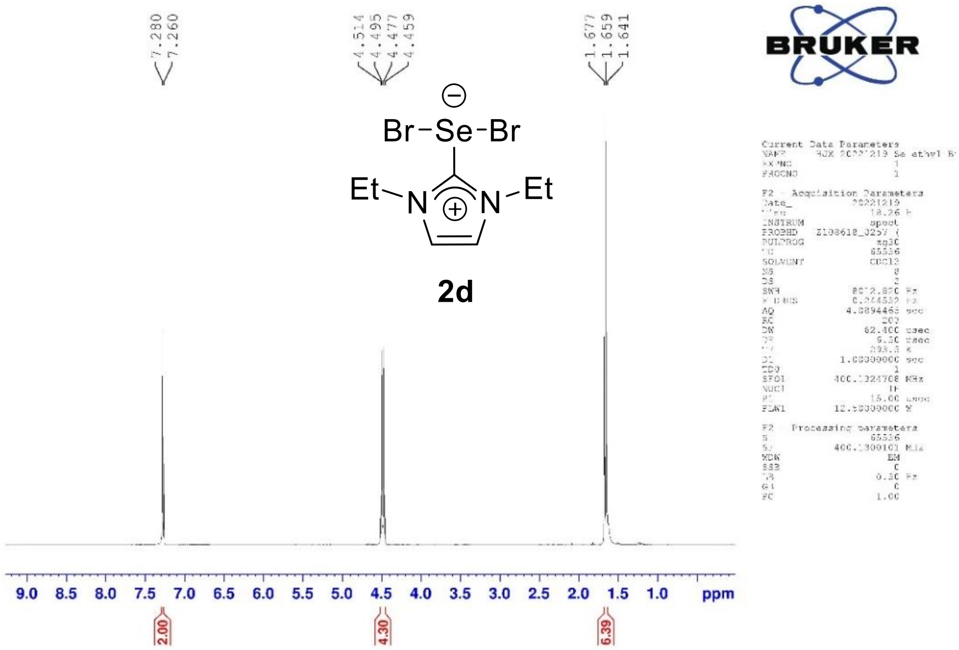

^{13}C NMR

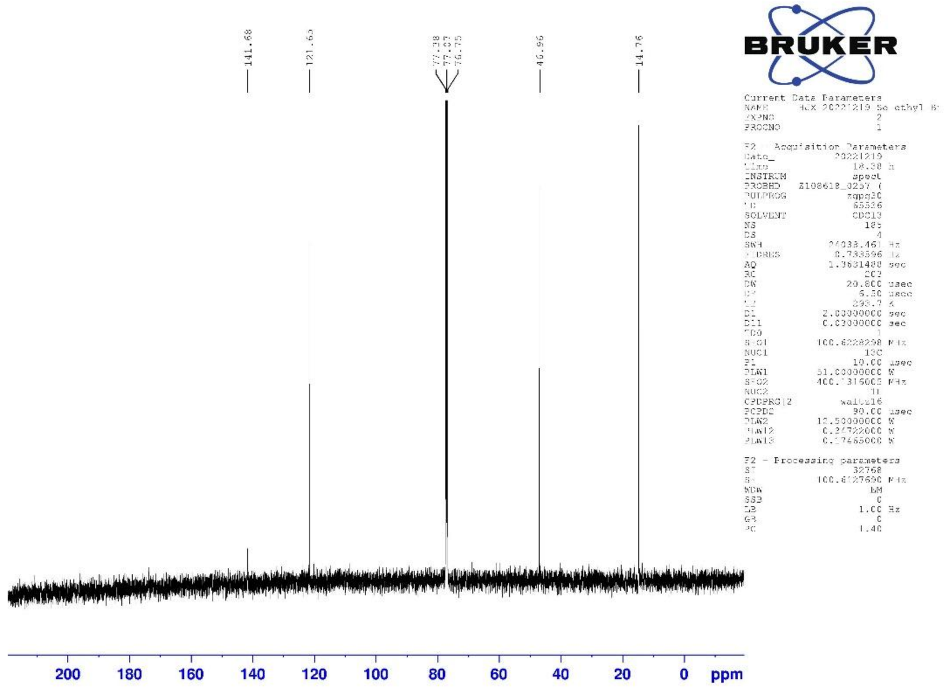

^{77}Se NMR

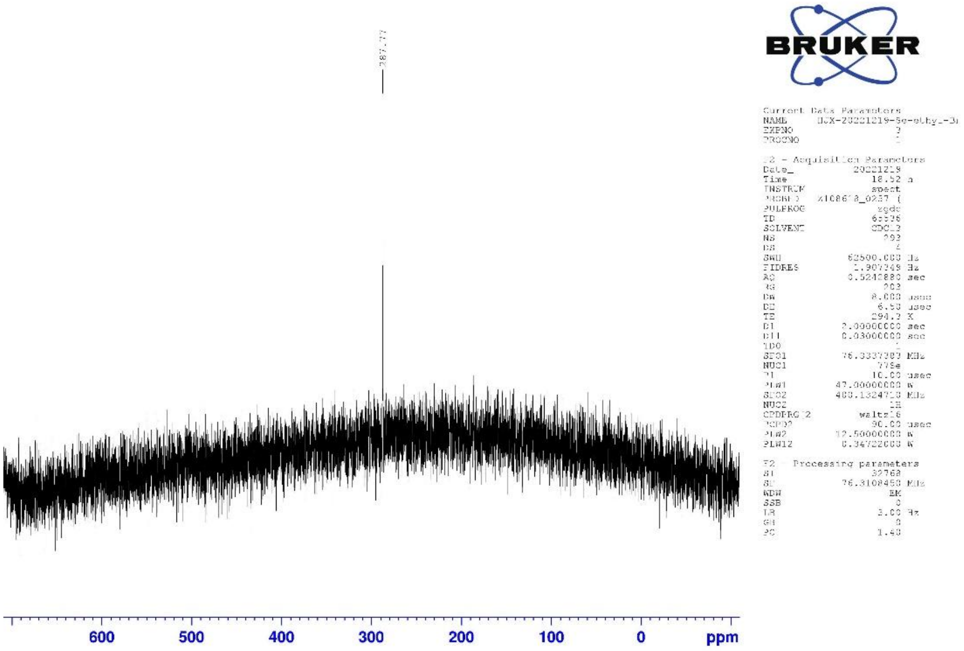

^{1}H NMR

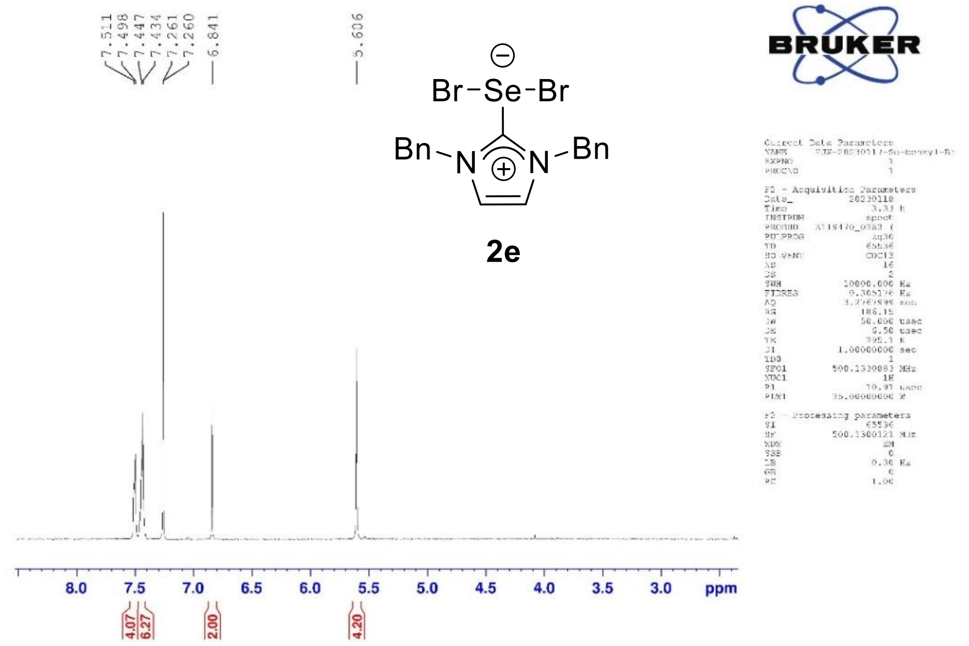

^{13}C NMR

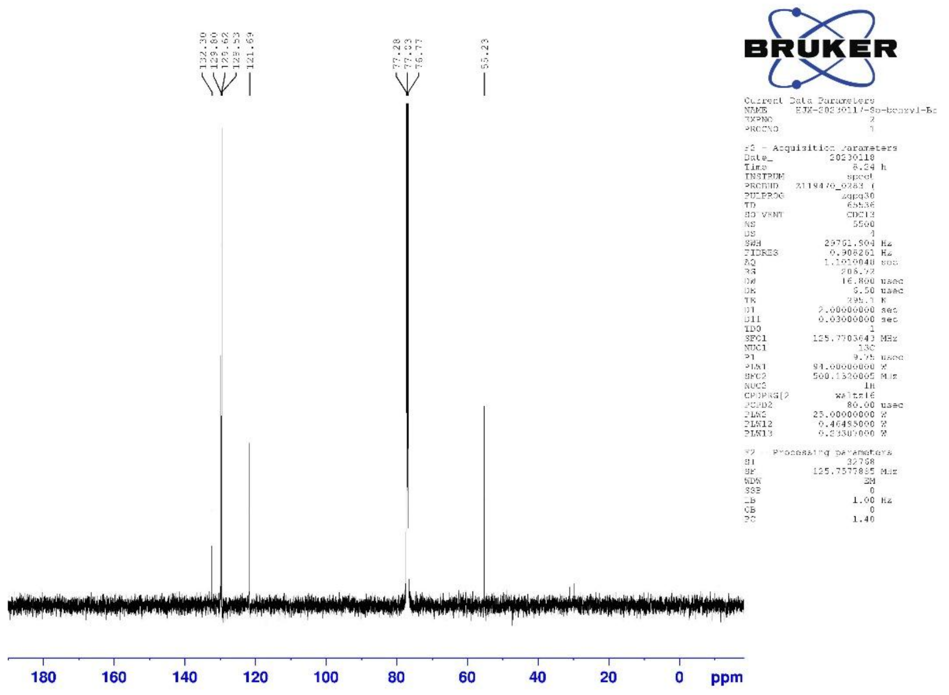

^{77}Se NMR

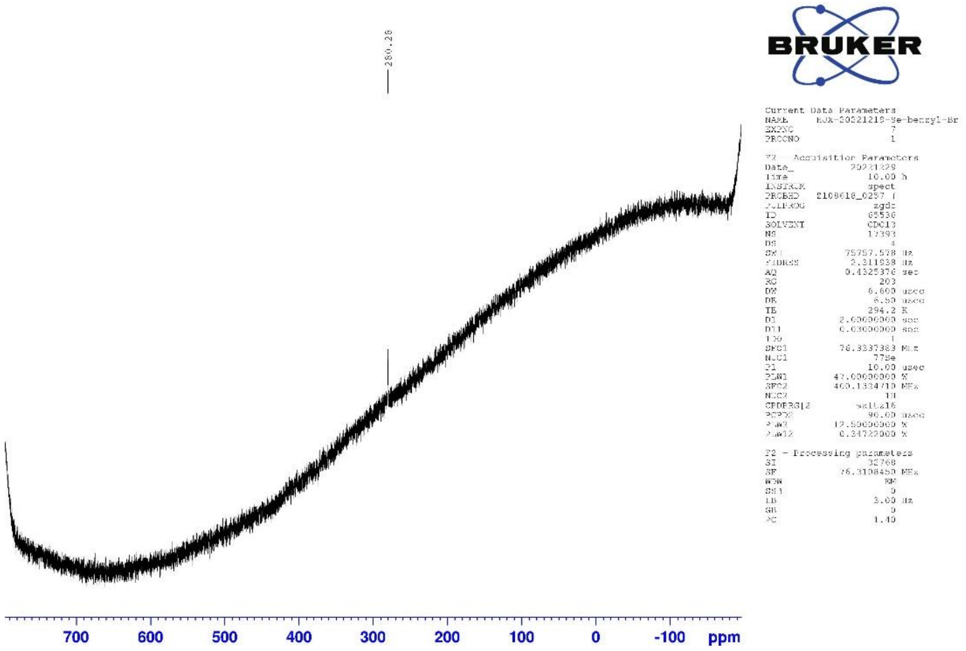
280.28
Current Data Parameters
F2 - Processing parameters
700
600
500
400
300
200
100
0
-100
ppm

^{1}H NMR

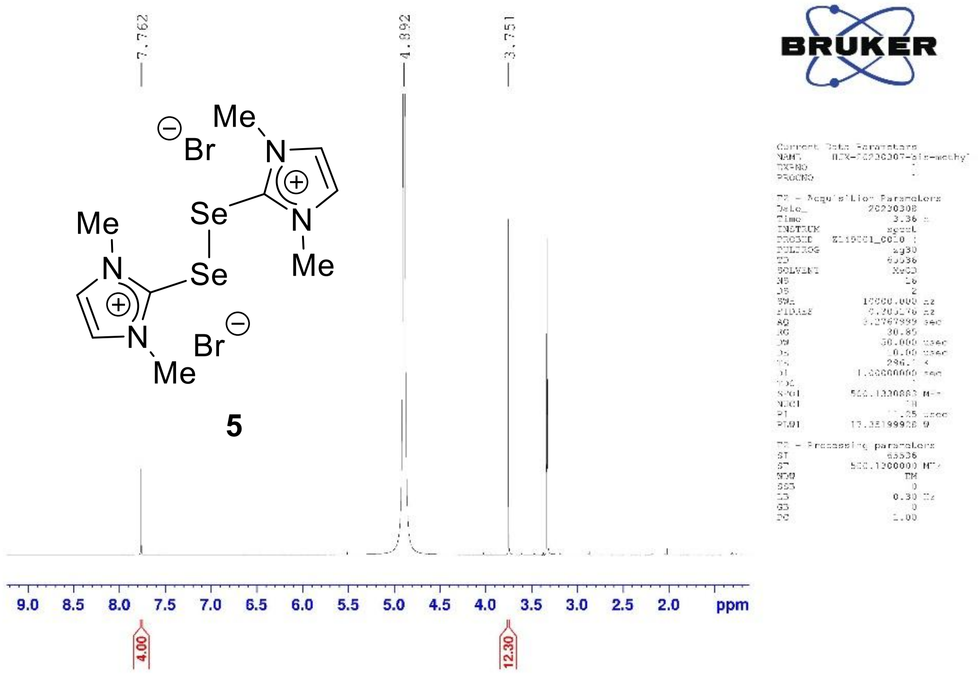

^{13}C NMR

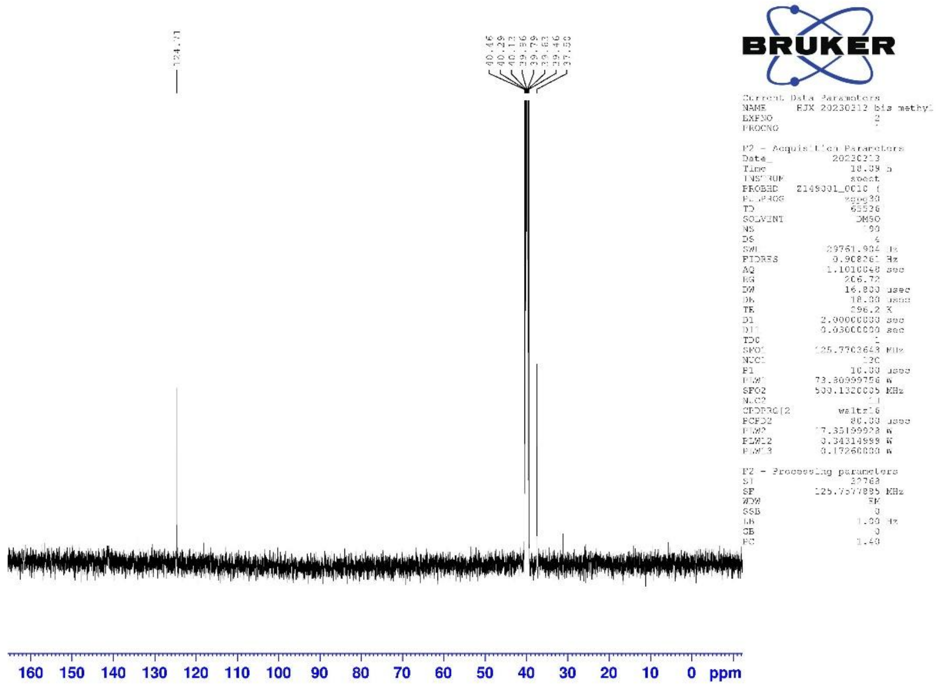

^{77}Se NMR

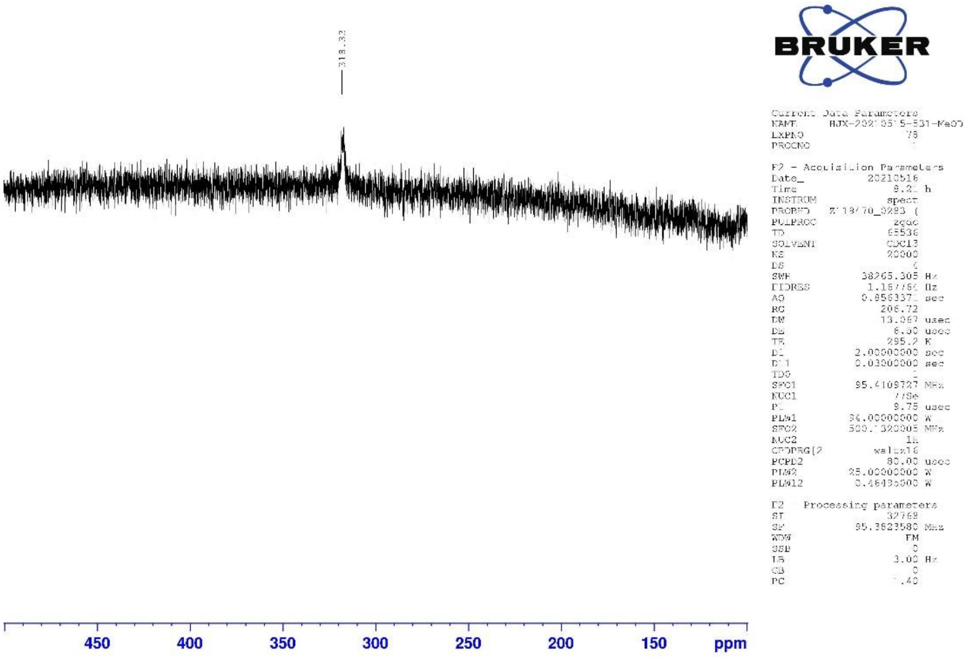

318.32
BRUKER
Current Data Parameters
EXPNO 78
F2 - Acquisition Parameters
Date_ 20210516
INSTRUM spect
PULPROG zgqc
TD 65536
SOLVENT CDCl3
NS 20000
SWH 38265.305 Hz
RG 206.72
DW 13.067 usec
DE 6.50 usec
TE 295.2 K
D1 2.00000000 sec
SFO1 95.4109727 MHz
NUC1 77Se
P1 9.75 usec
PLW1 94.00000000 W
NUC2 1H
CPDPRG[2 waltz16
PCPD2 80.00 usec
PLW2 25.00000000 W
F2 - Processing parameters
SI 32768
SF 95.3823580 MHz
WDW EM
SSB 0
LB 3.00 Hz
GB 0
450
400
350
300
250
200
150
ppm

^{1}H NMR

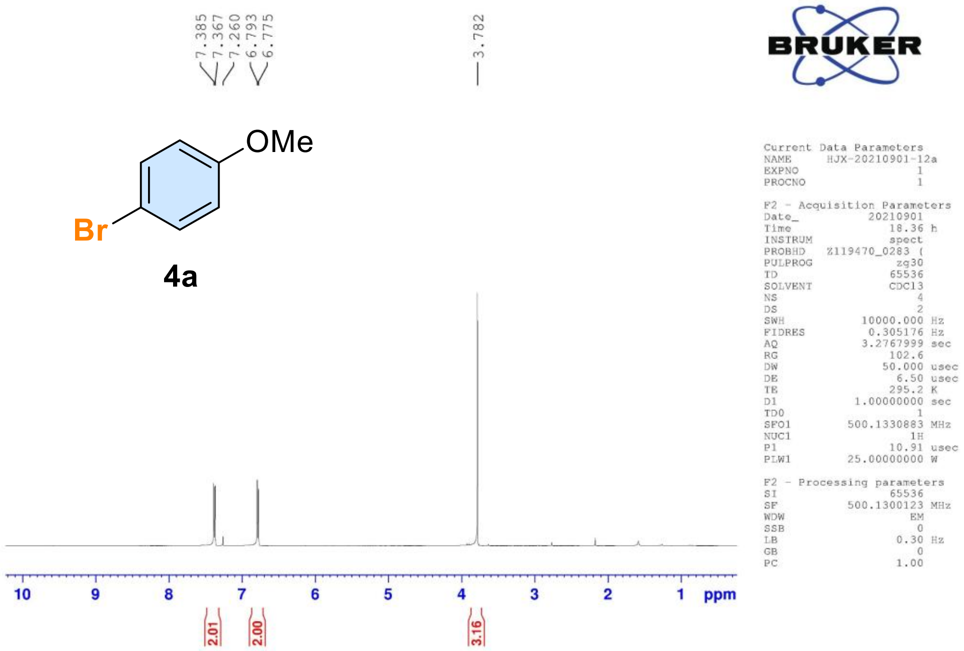

^{13}C NMR

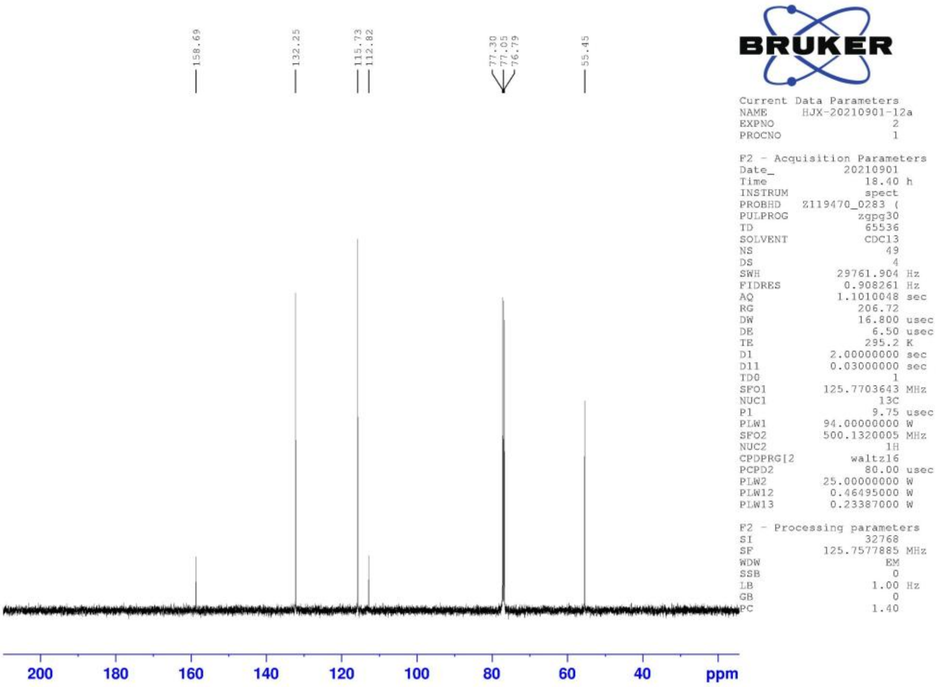

^{1}H NMR

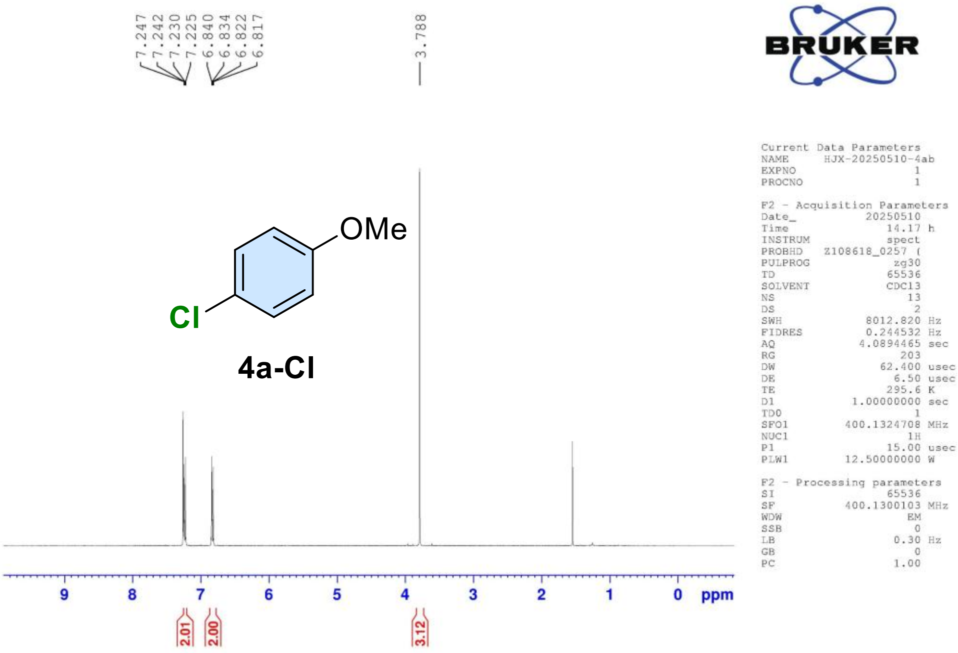

^{13}C NMR

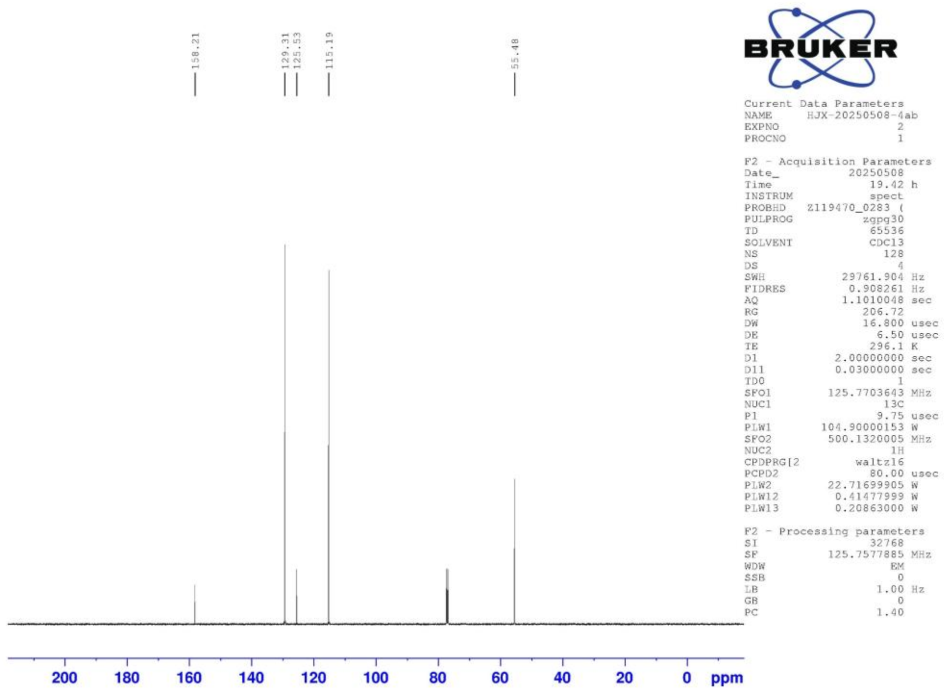

^{1}H NMR

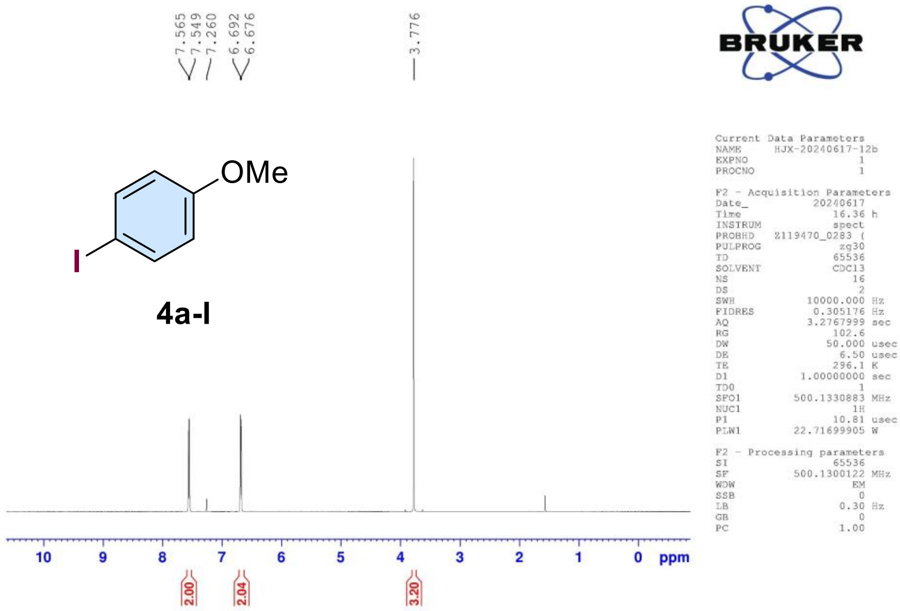

^{13}C NMR

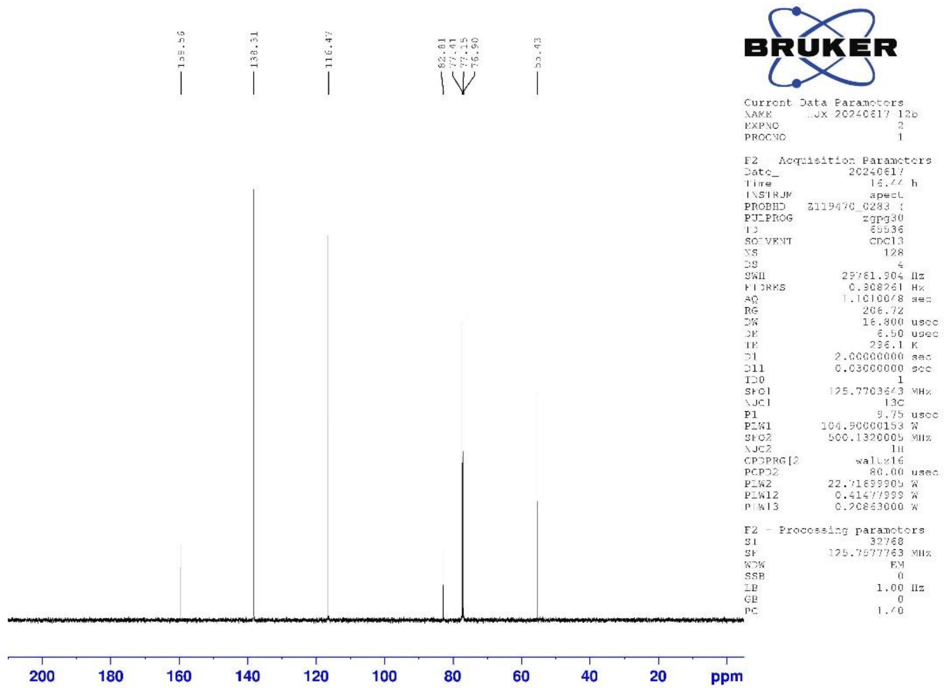

^{1}H NMR

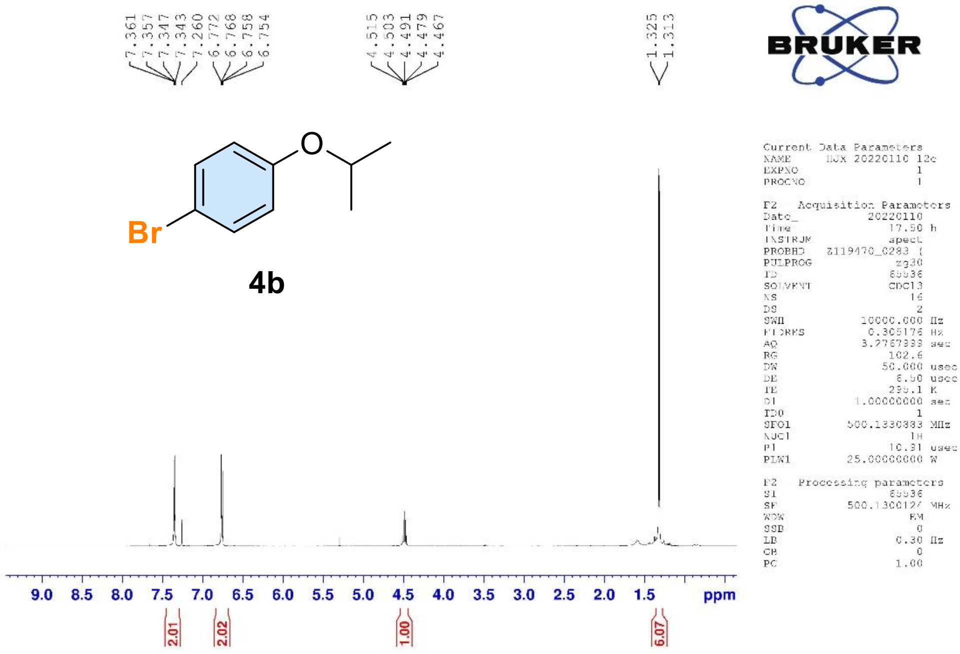

^{13}C NMR

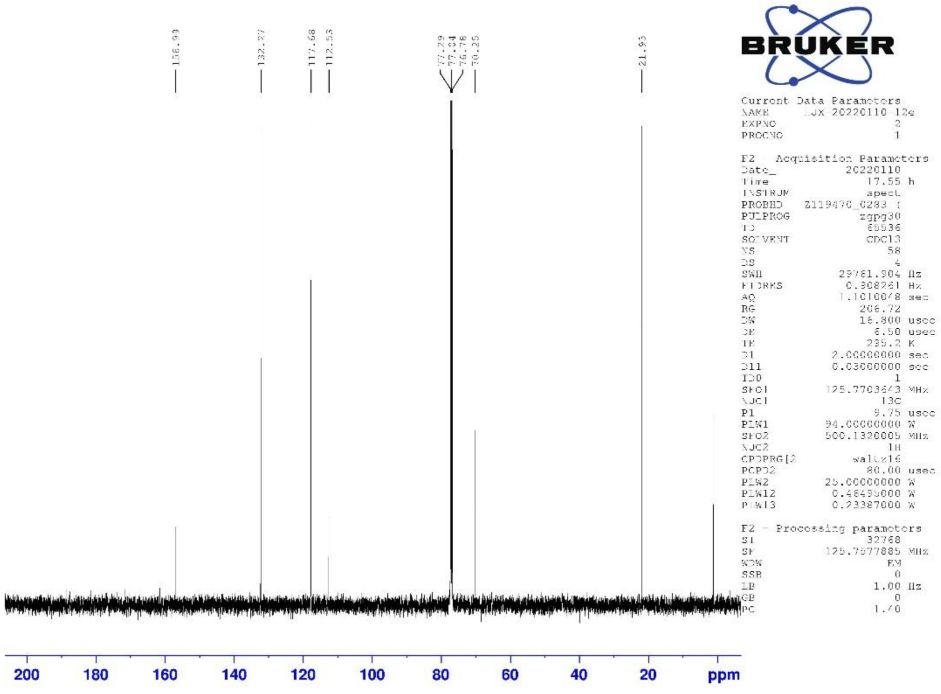

^{1}H NMR

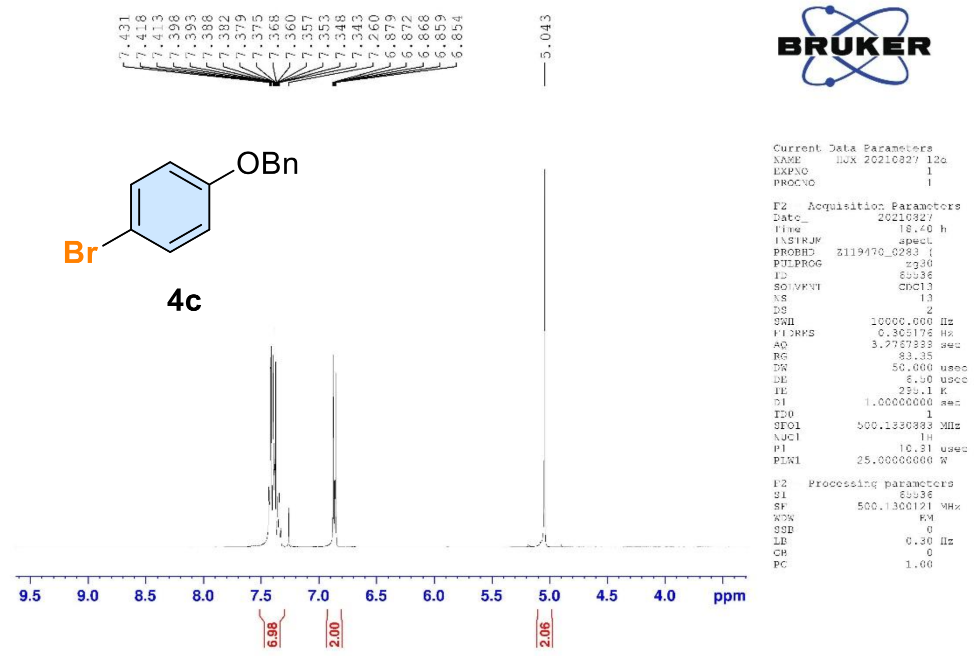

^{13}C NMR

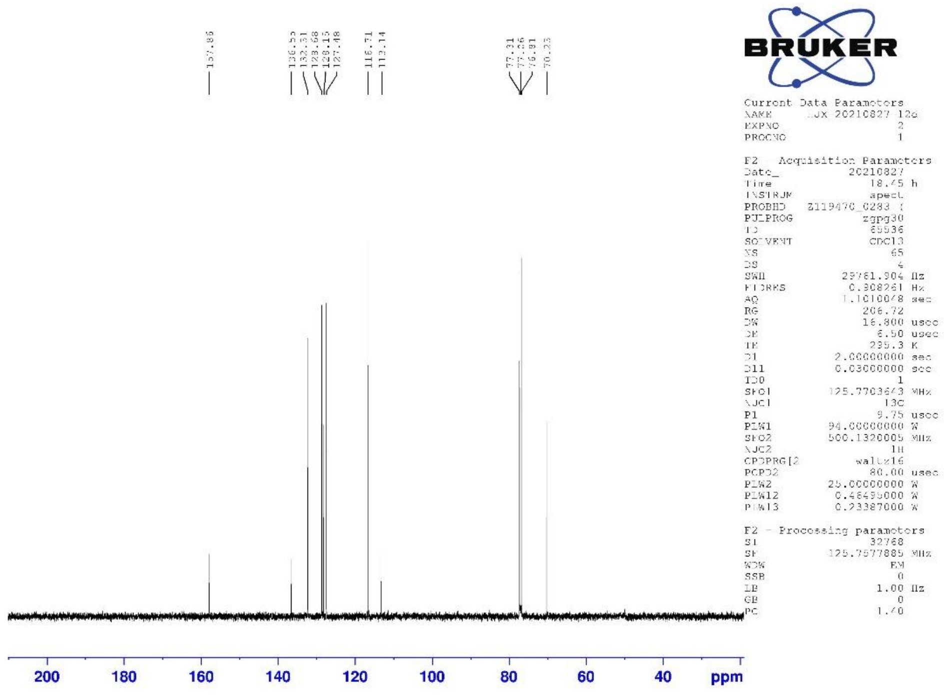

^{1}H NMR

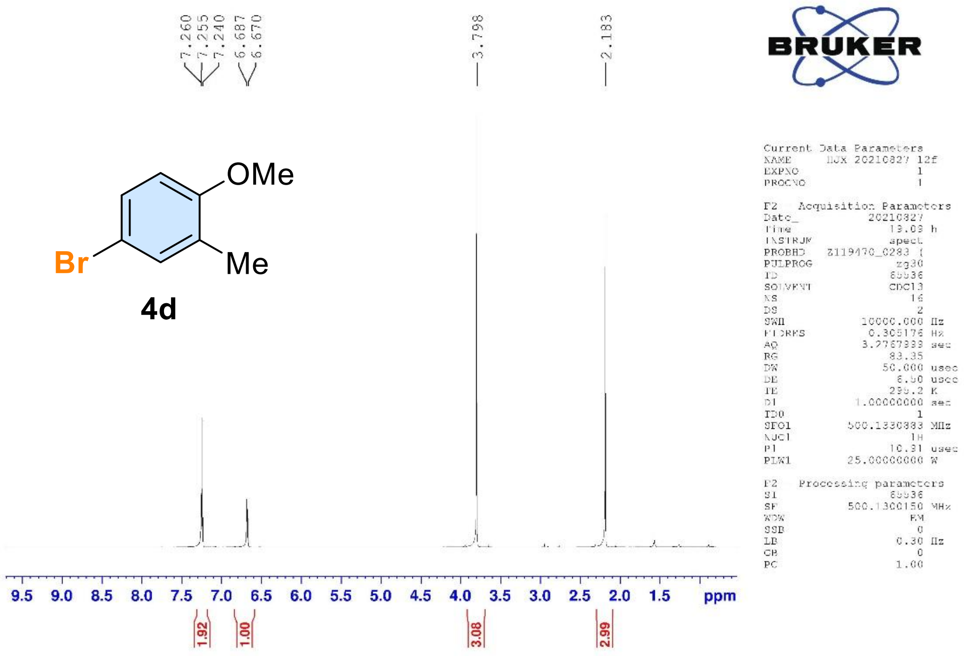

^{13}C NMR

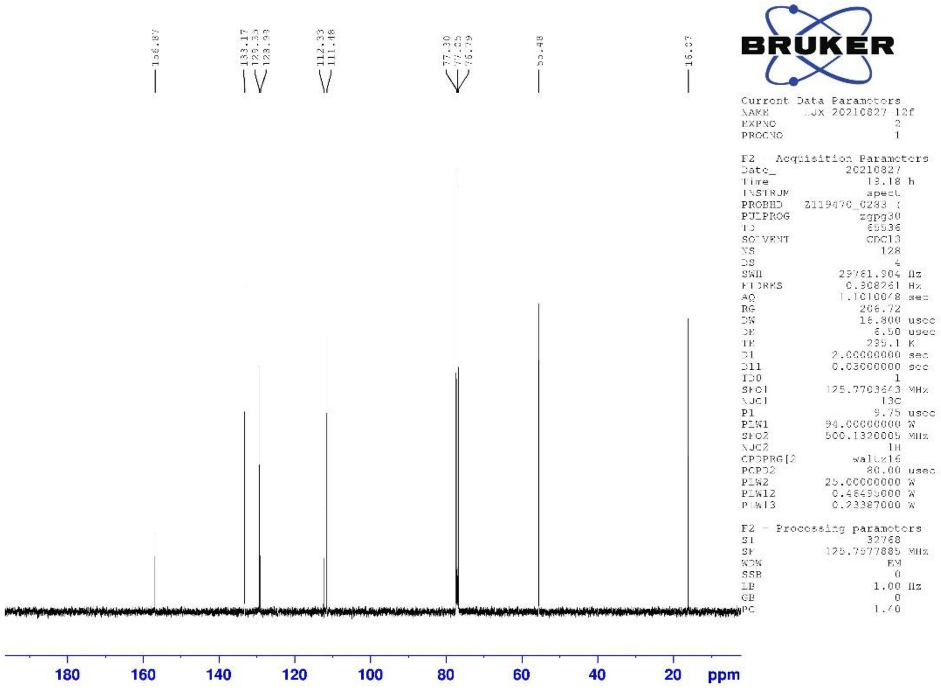

^{1}H NMR

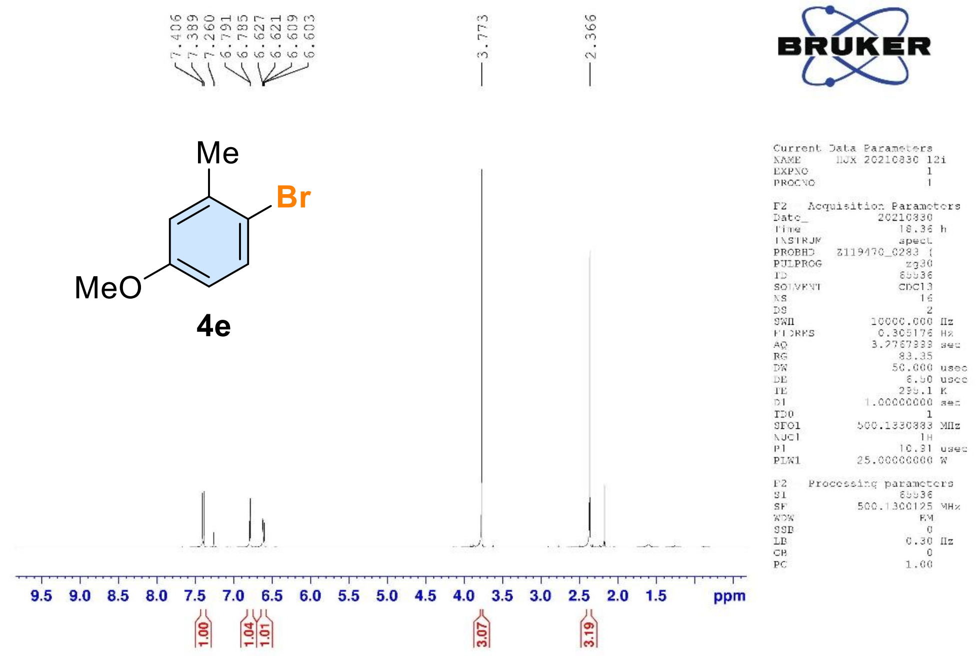

^{13}C NMR

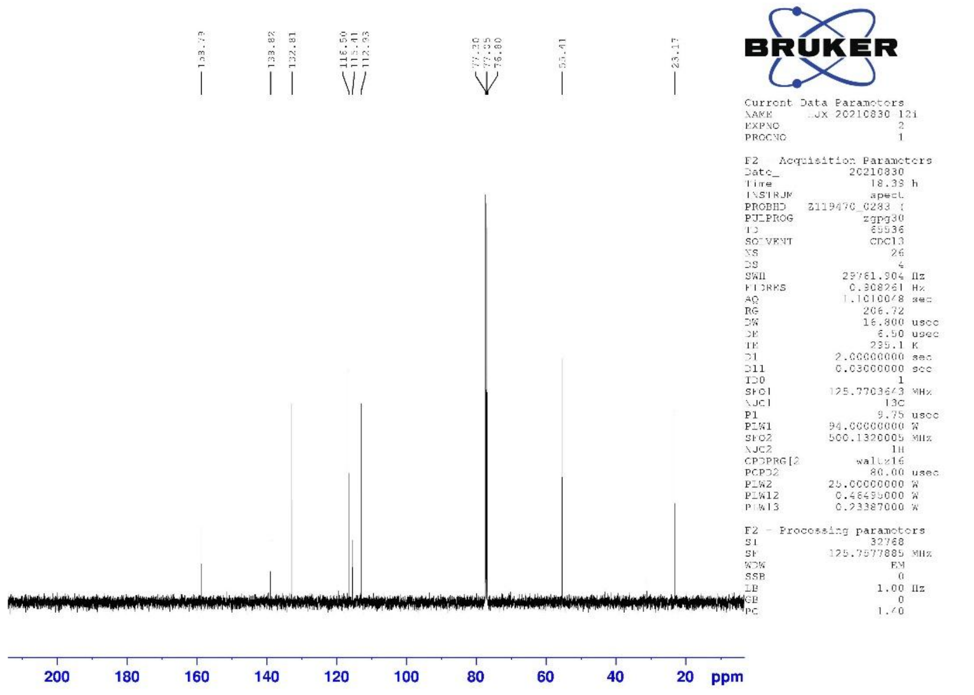

^{1}H NMR

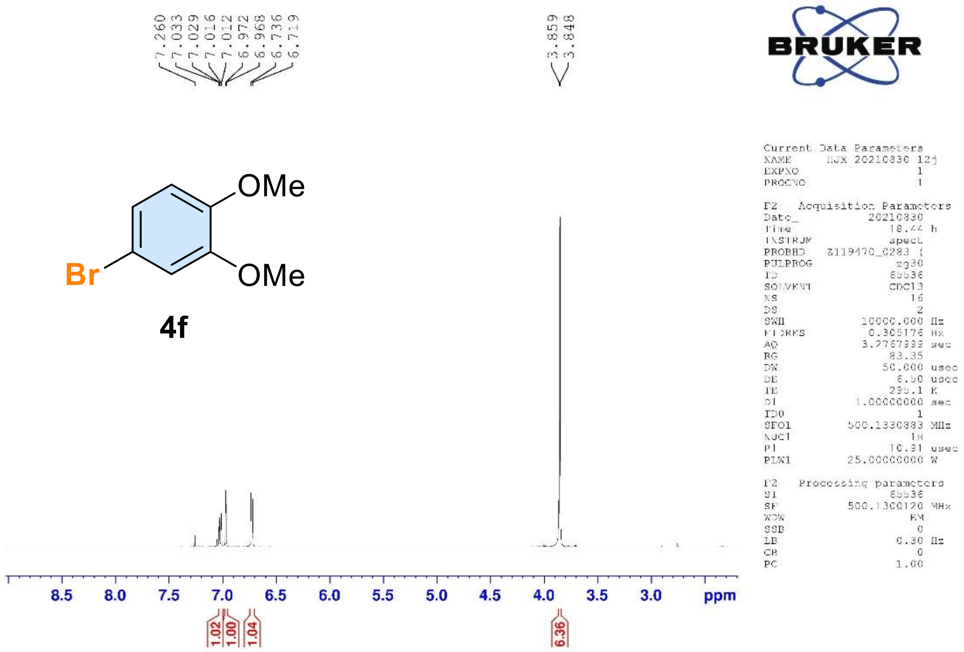

^{13}C NMR

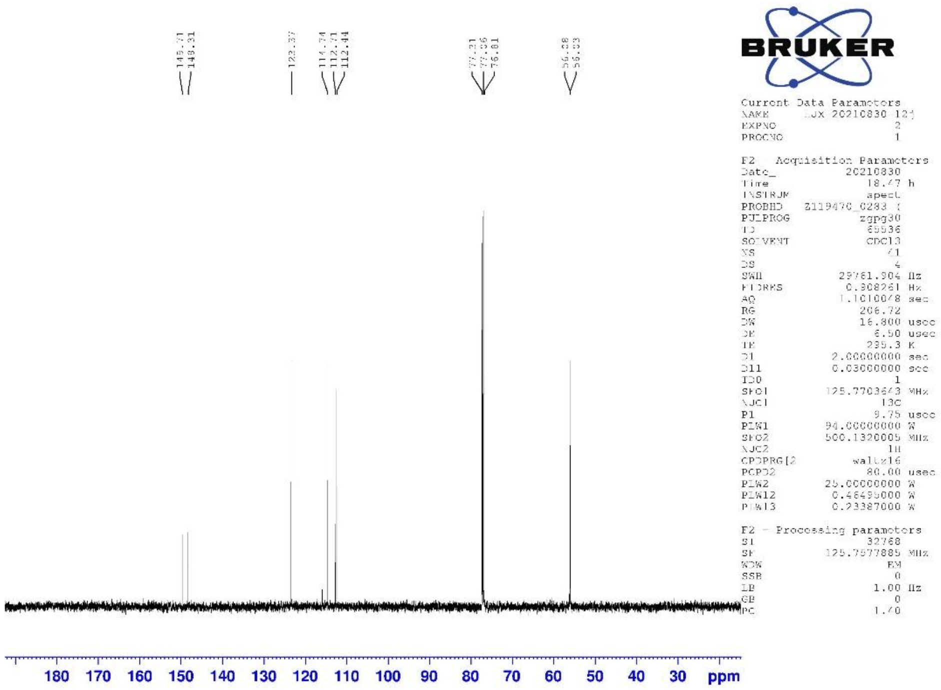

^{1}H NMR

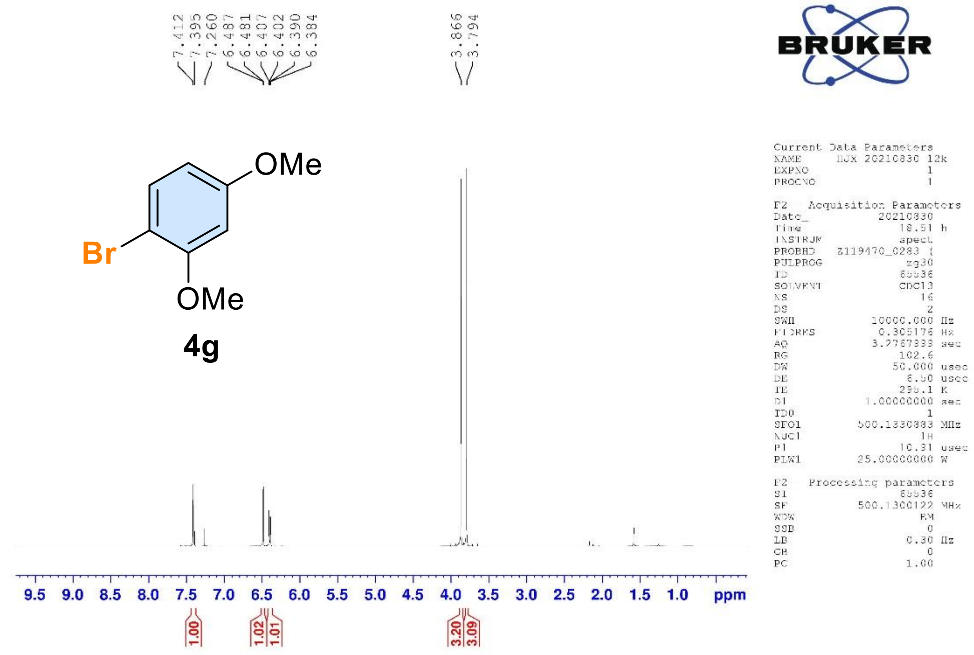

^{13}C NMR

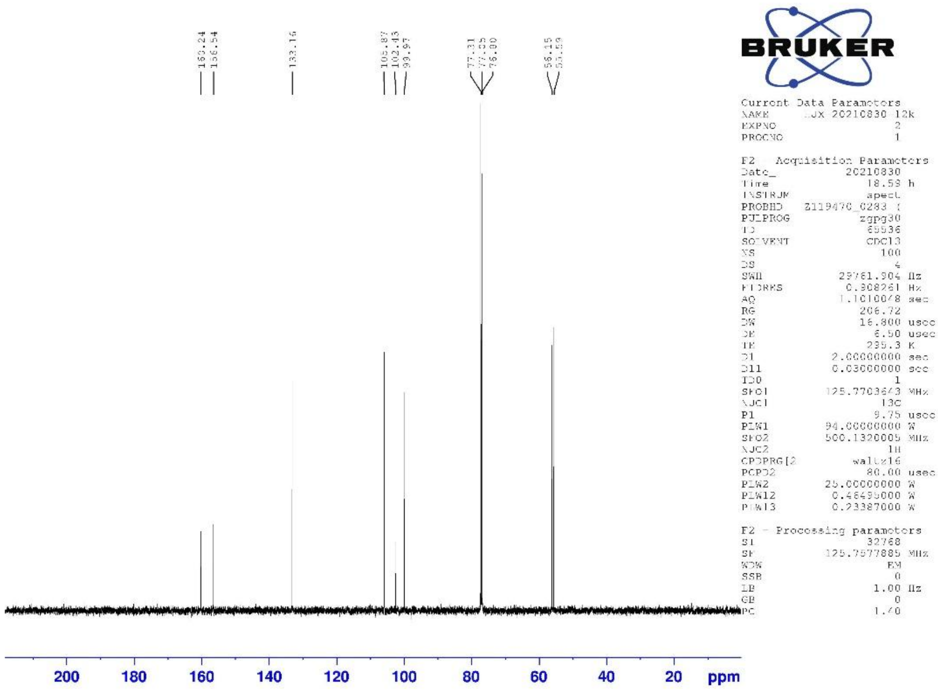

^{1}H NMR

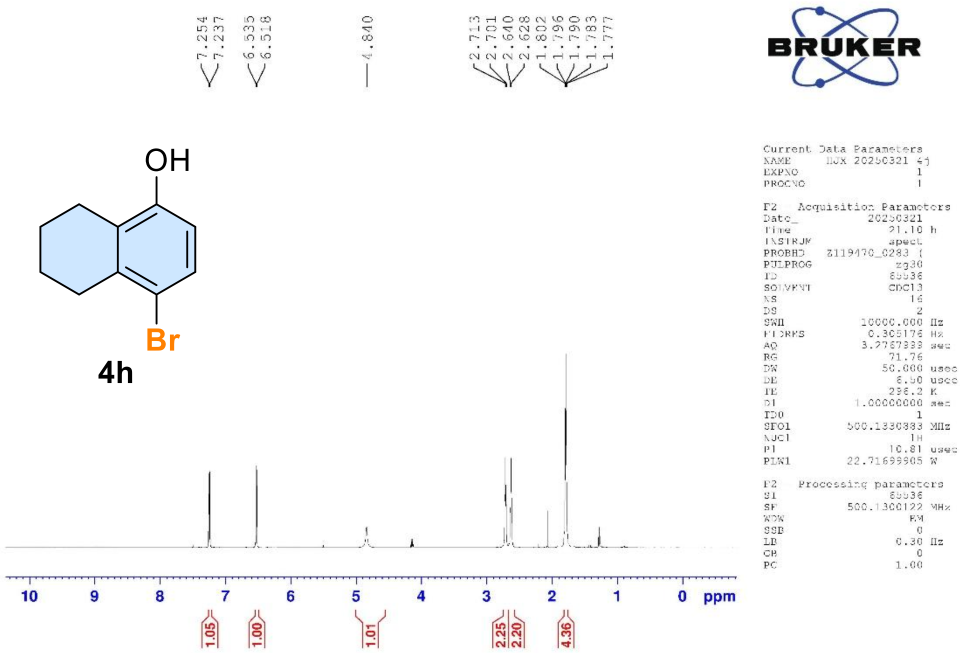

^{13}C NMR

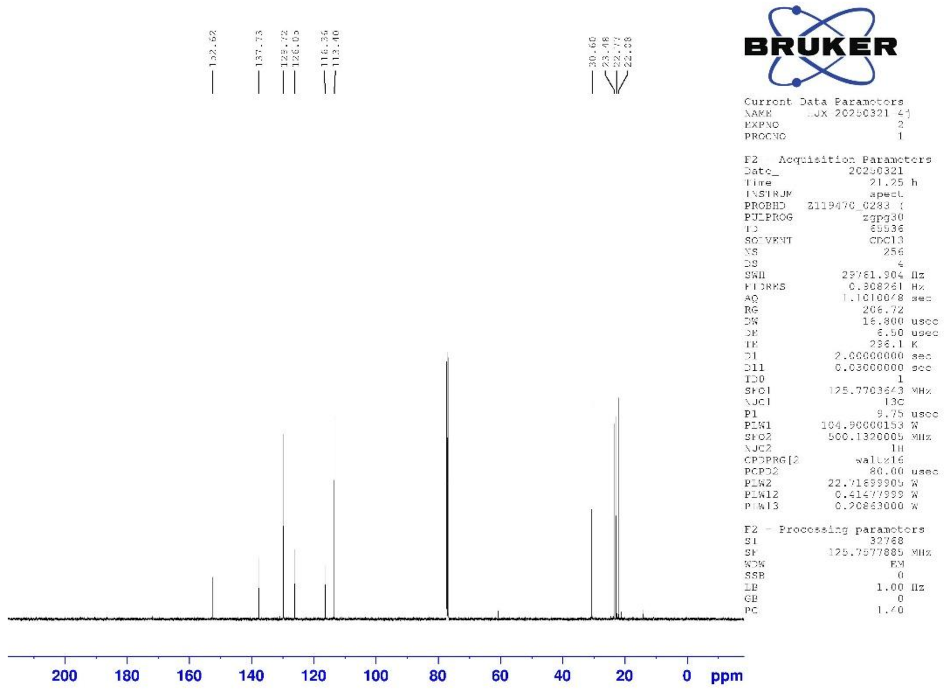

^{1}H NMR

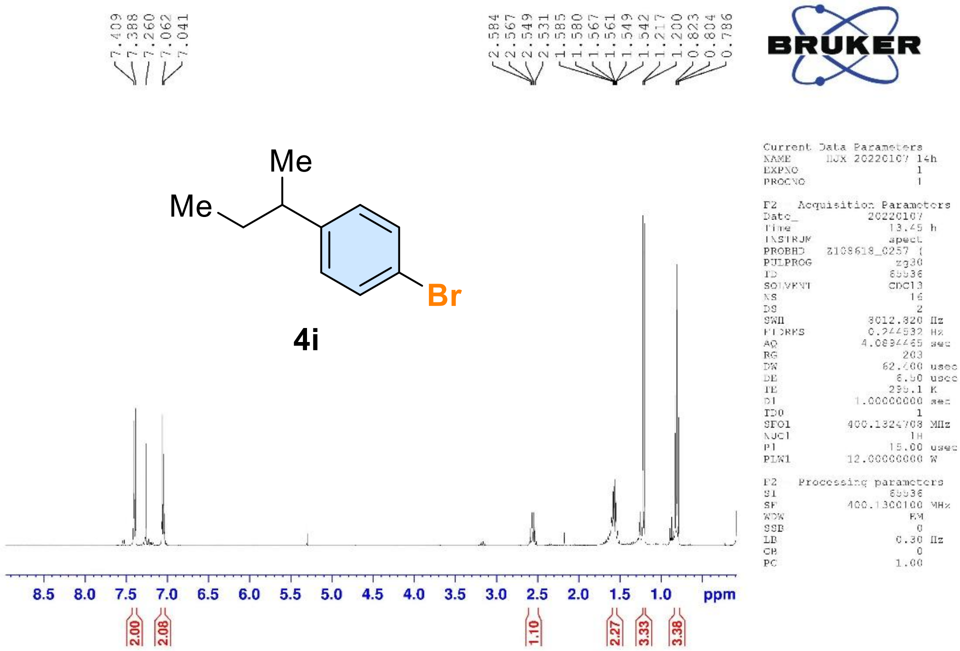

^{13}C NMR

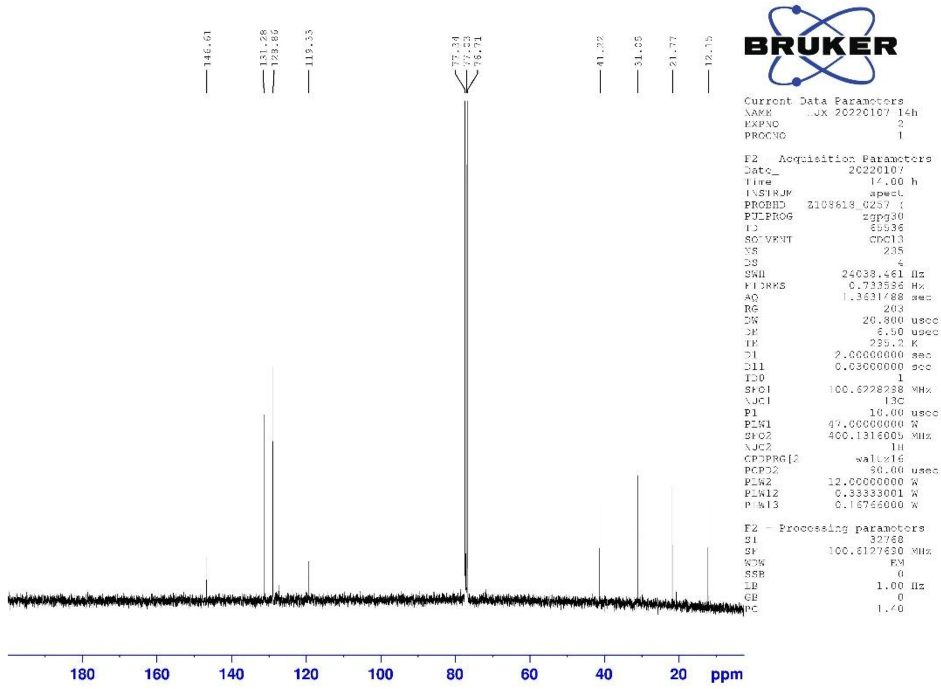

^{1}H NMR

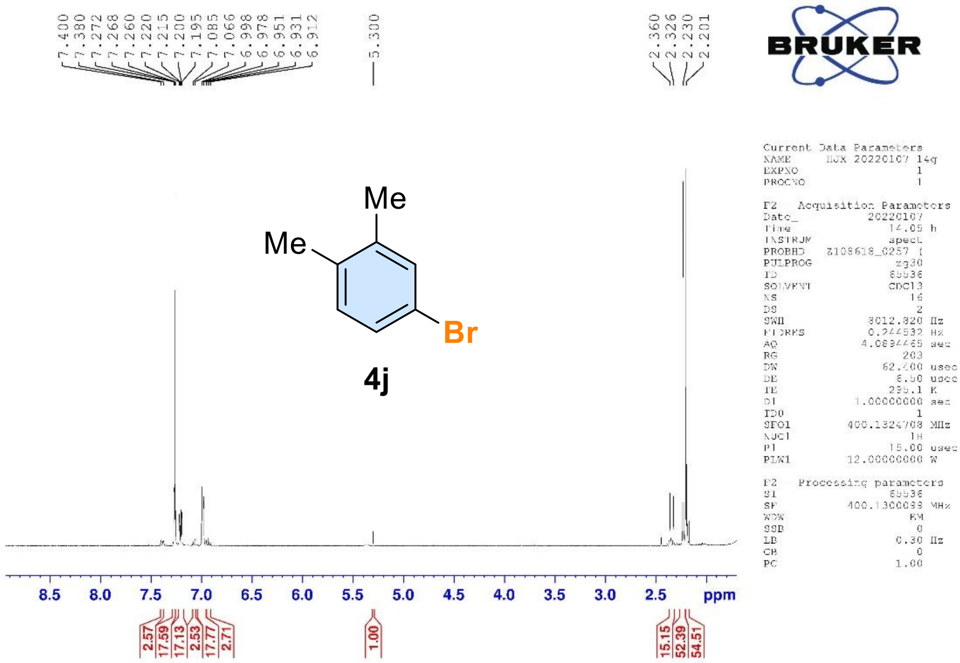

^{13}C NMR

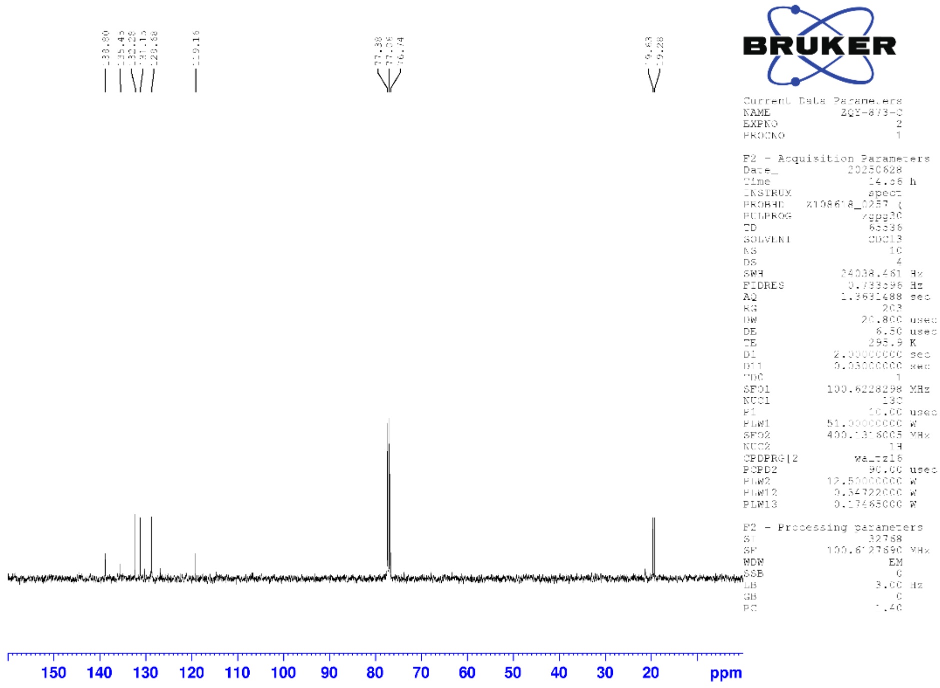

^{1}H NMR

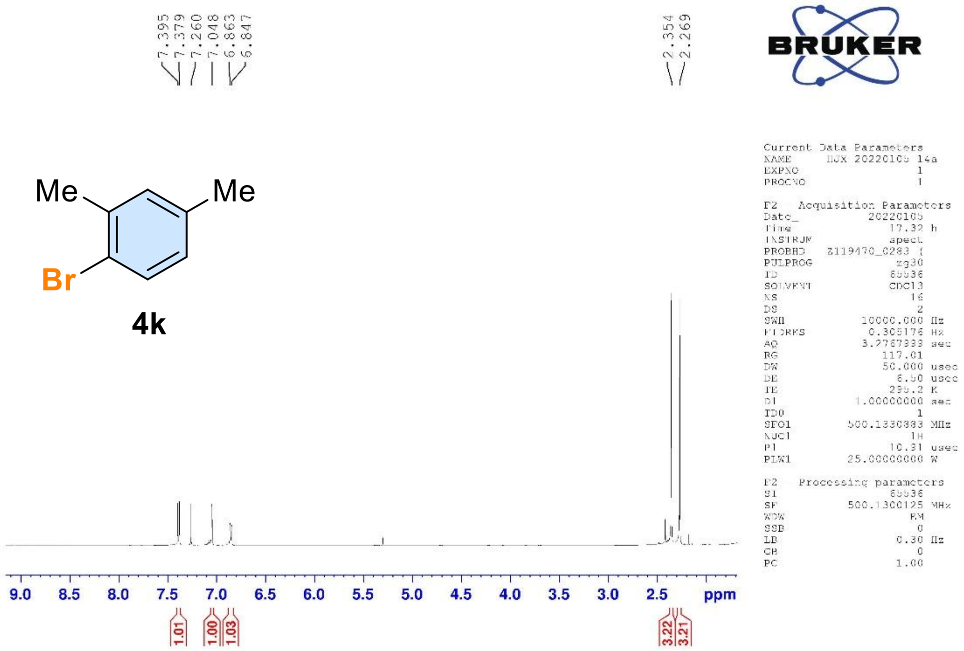

^{13}C NMR

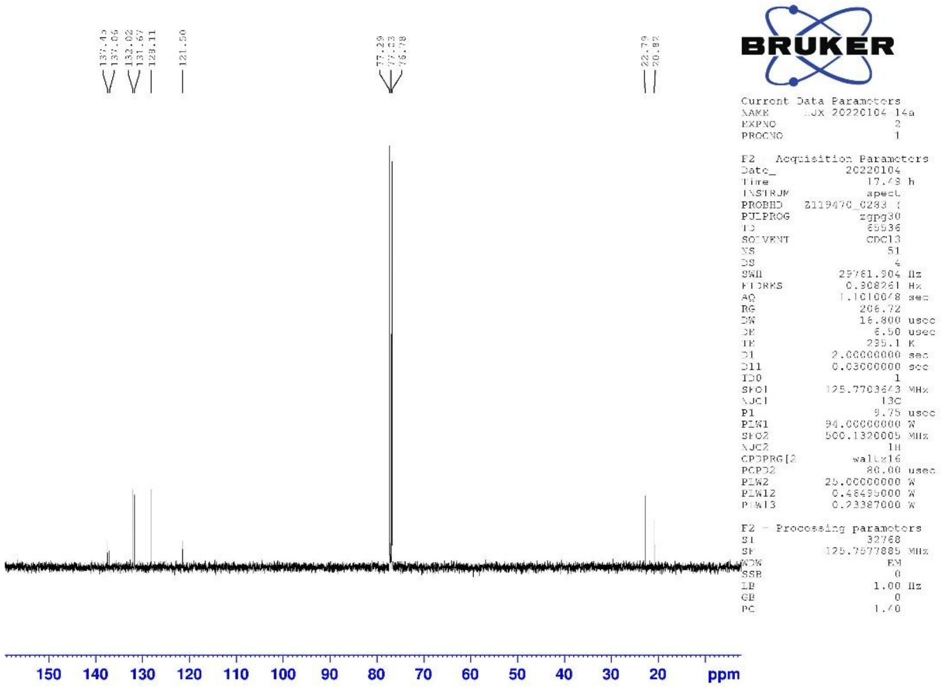

^{1}H NMR

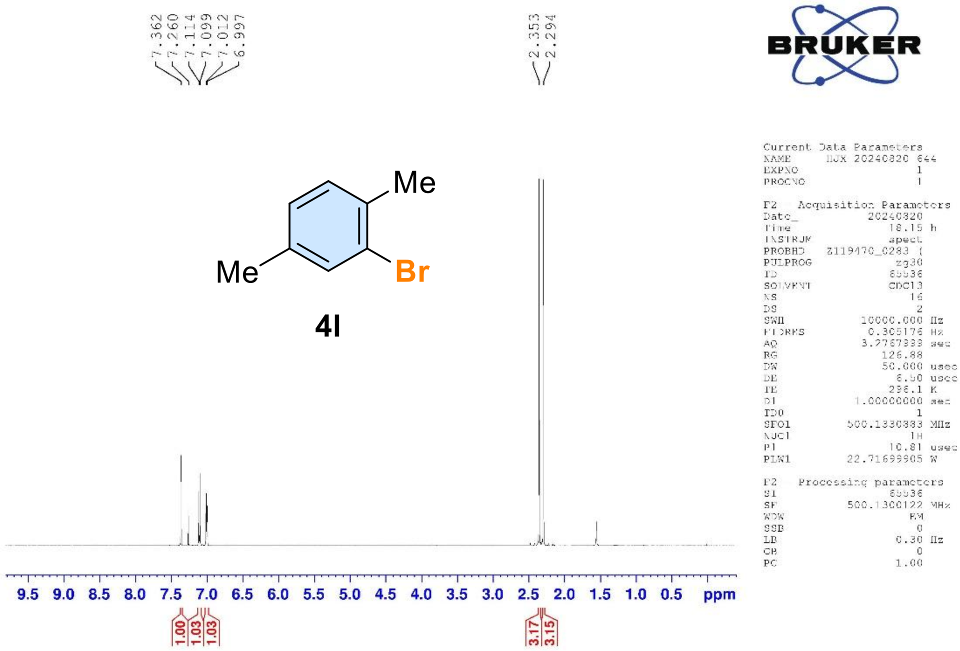

^{13}C NMR

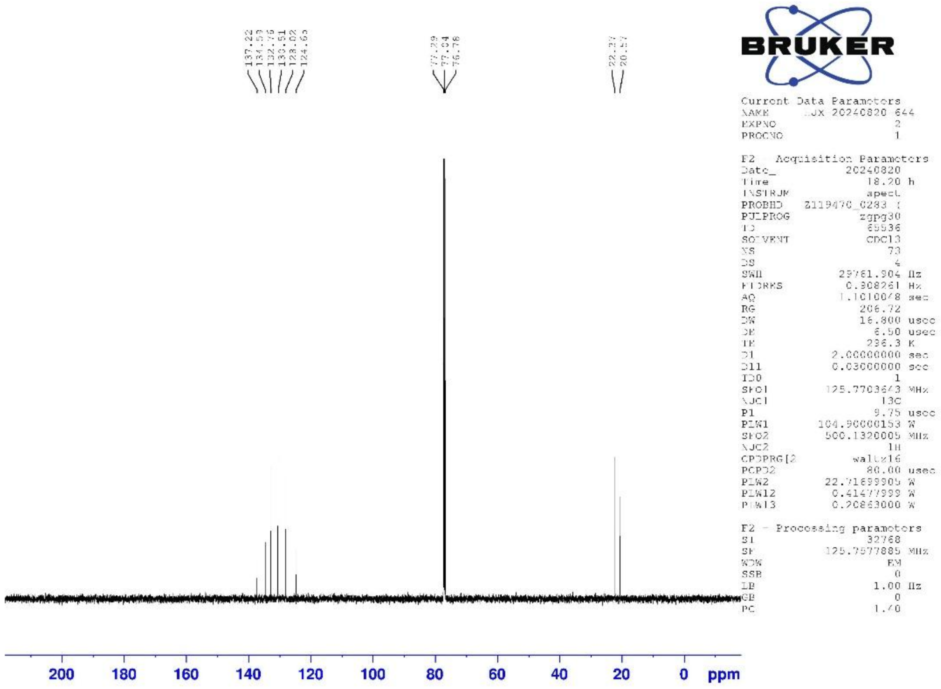

^{1}H NMR

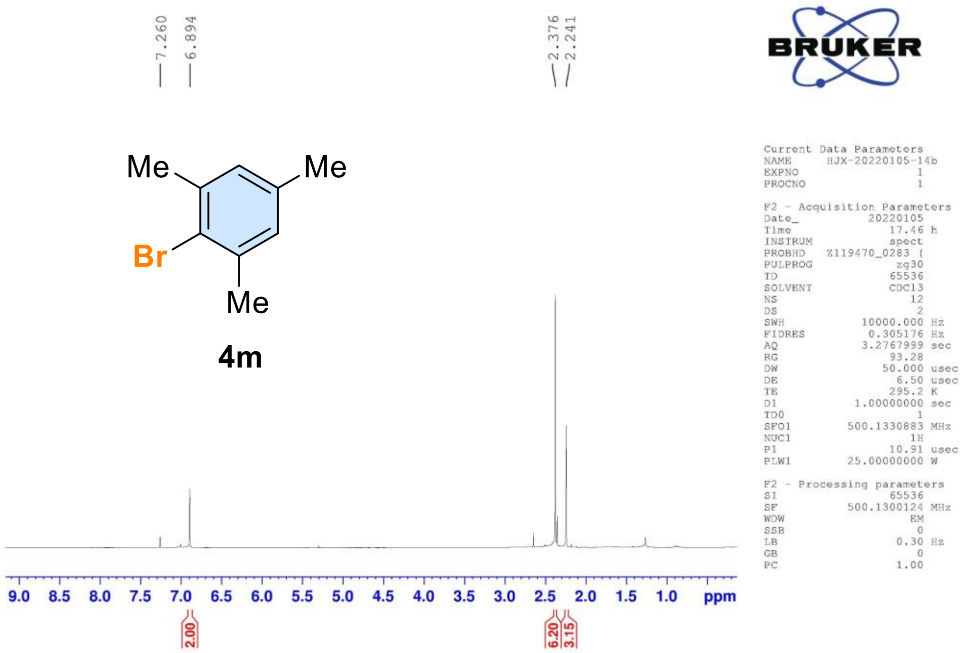

^{13}C NMR

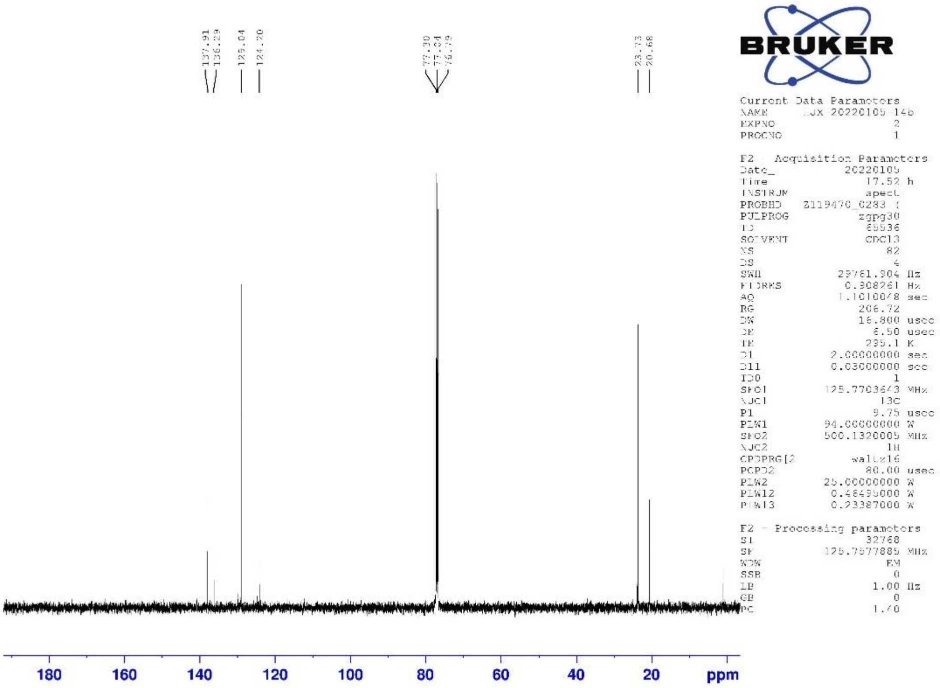

^{1}H NMR

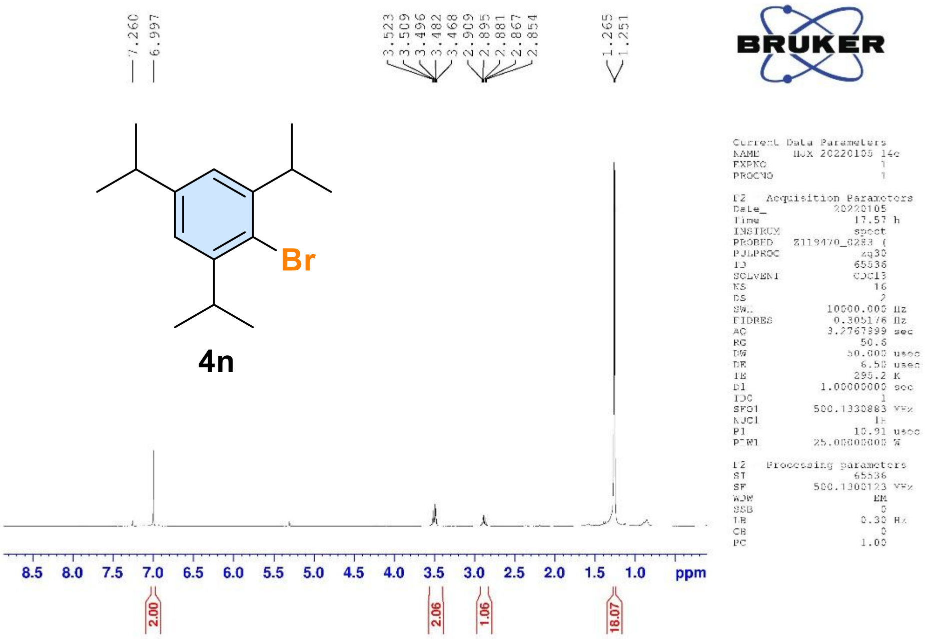

^{13}C NMR

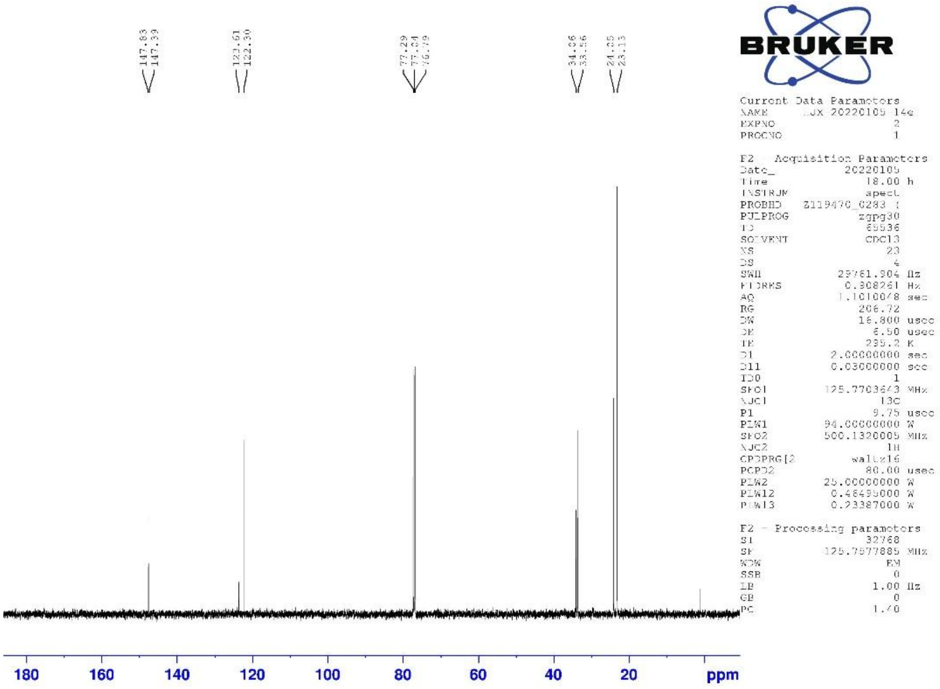

^{1}H NMR

4n-Cl

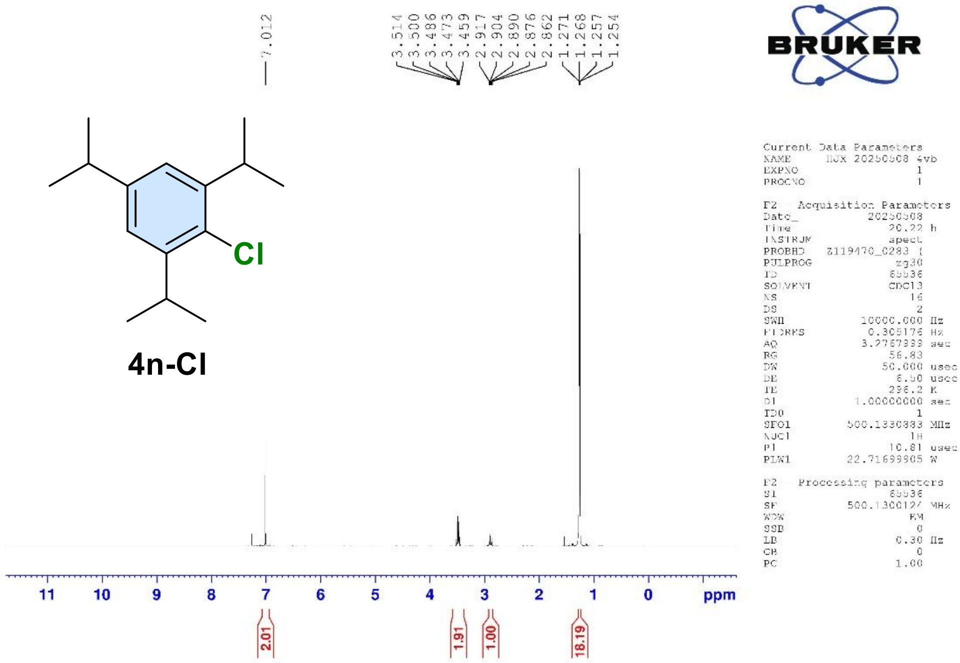

^{13}C NMR

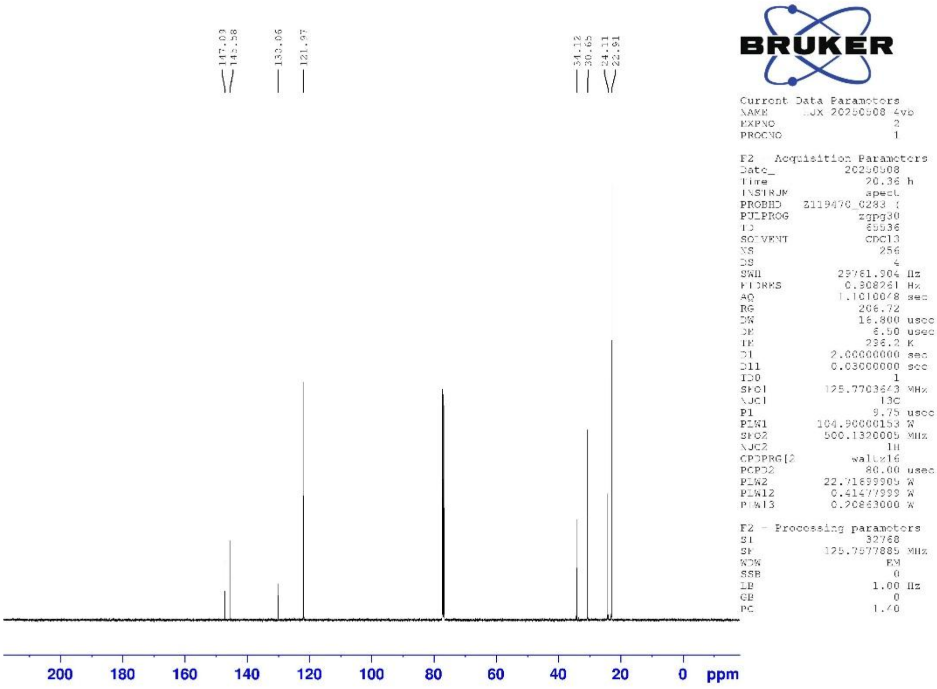

^{1}H NMR

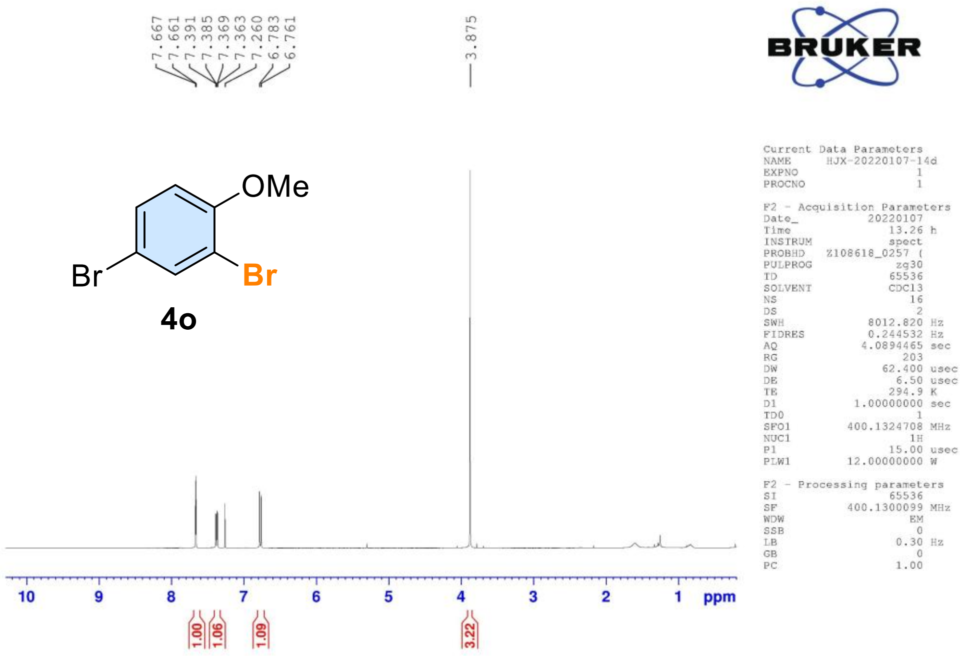

^{13}C NMR

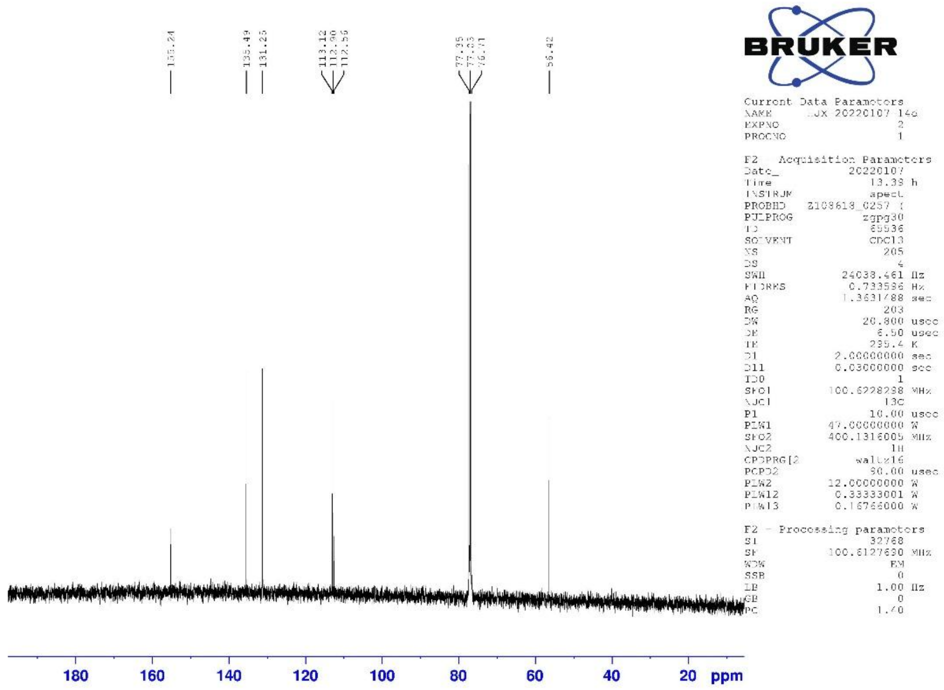

^{1}H NMR

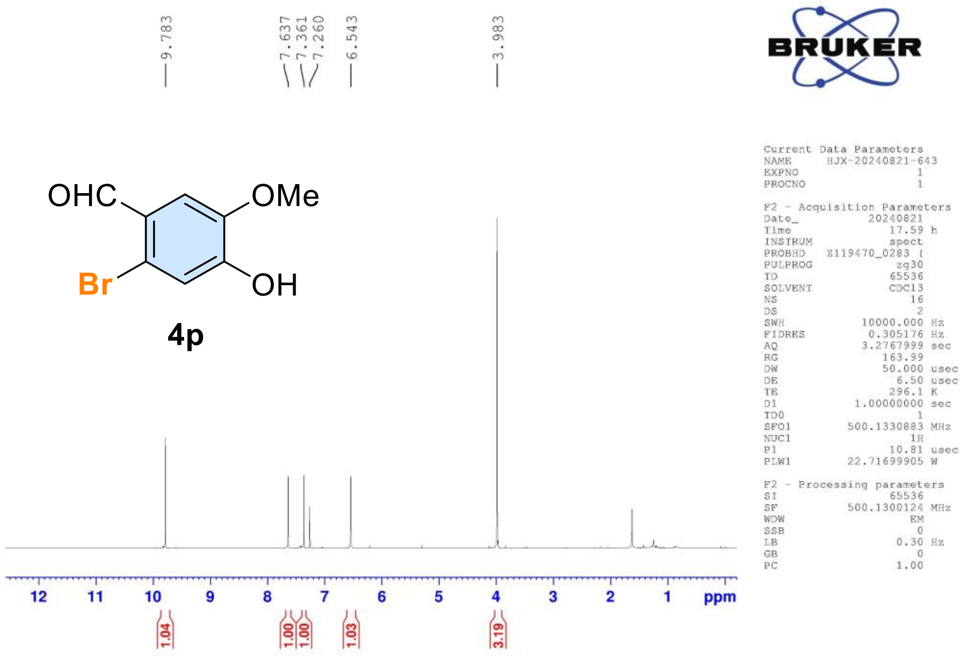

^{13}C NMR

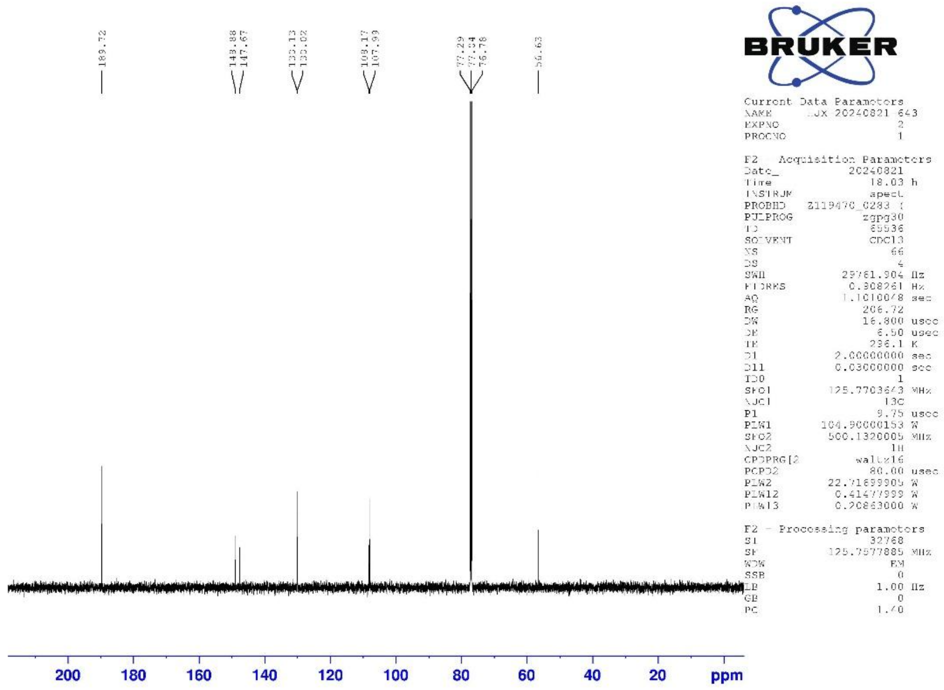

^{1}H NMR

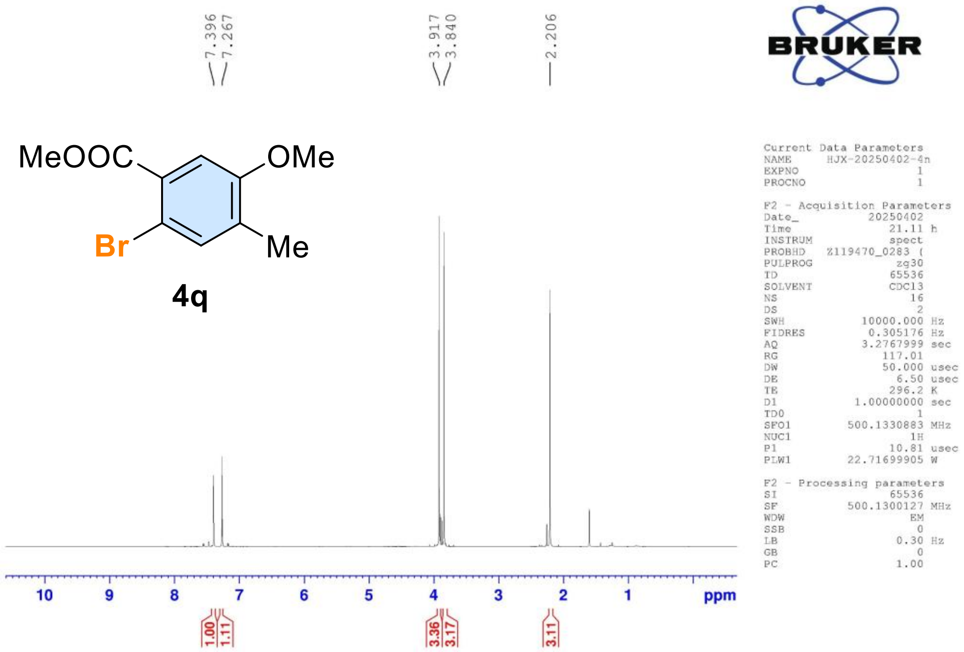

^{13}C NMR

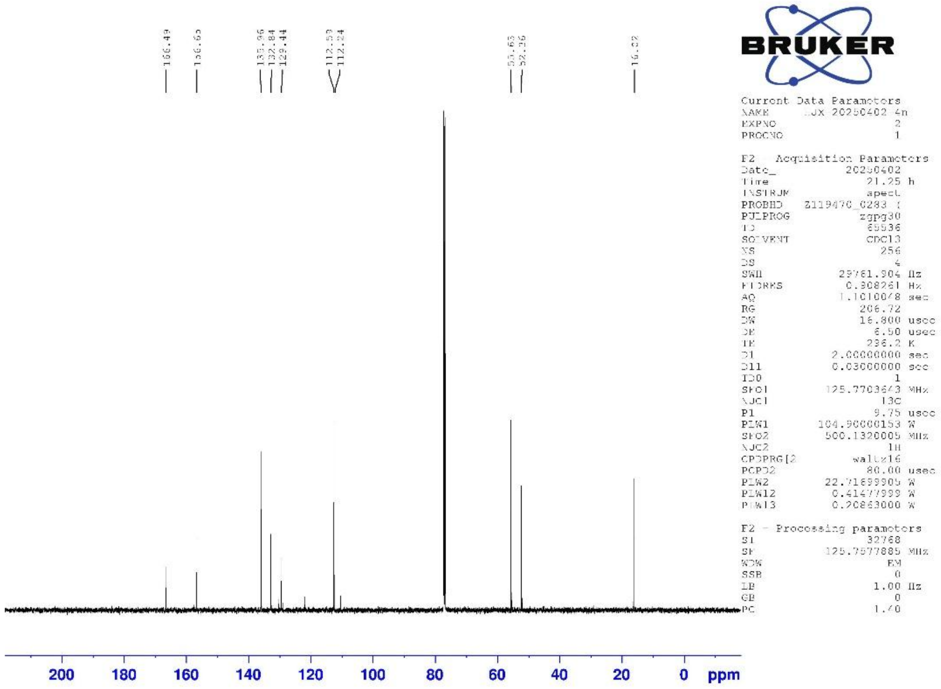

^{1}H NMR

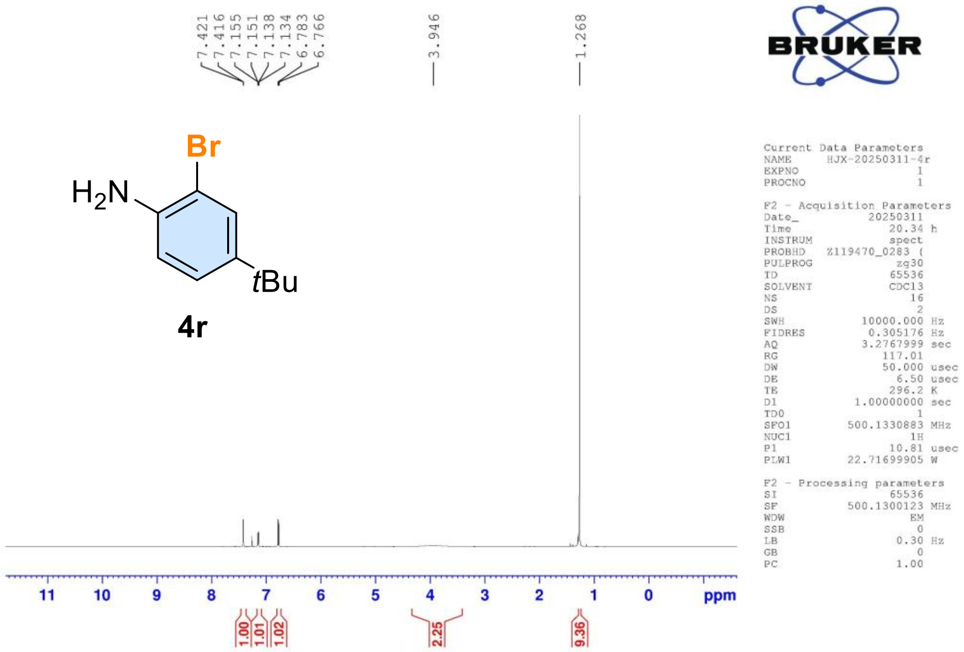

^{13}C NMR

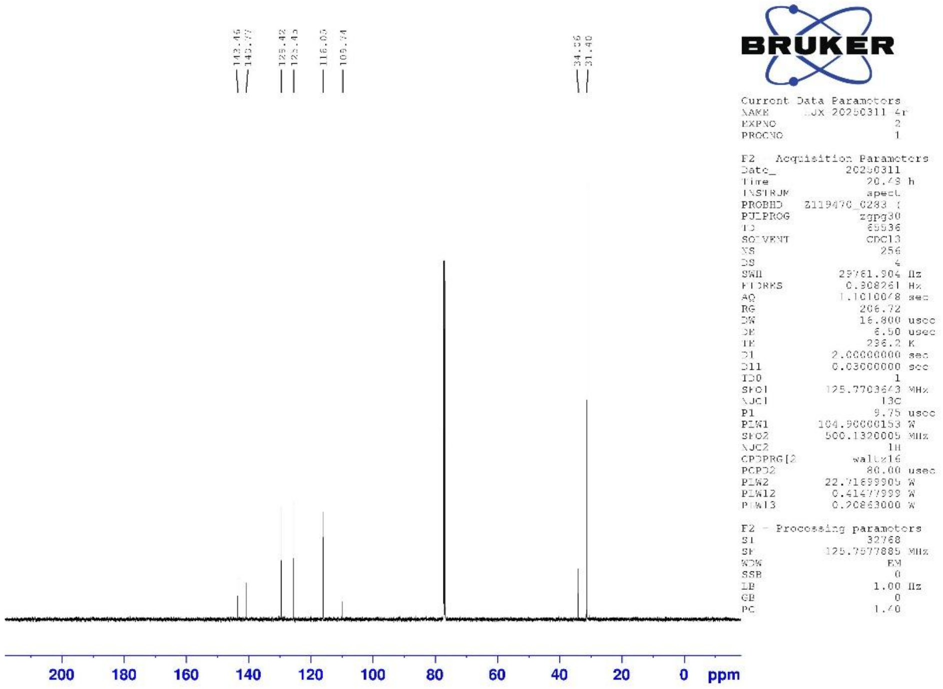

^{1}H NMR

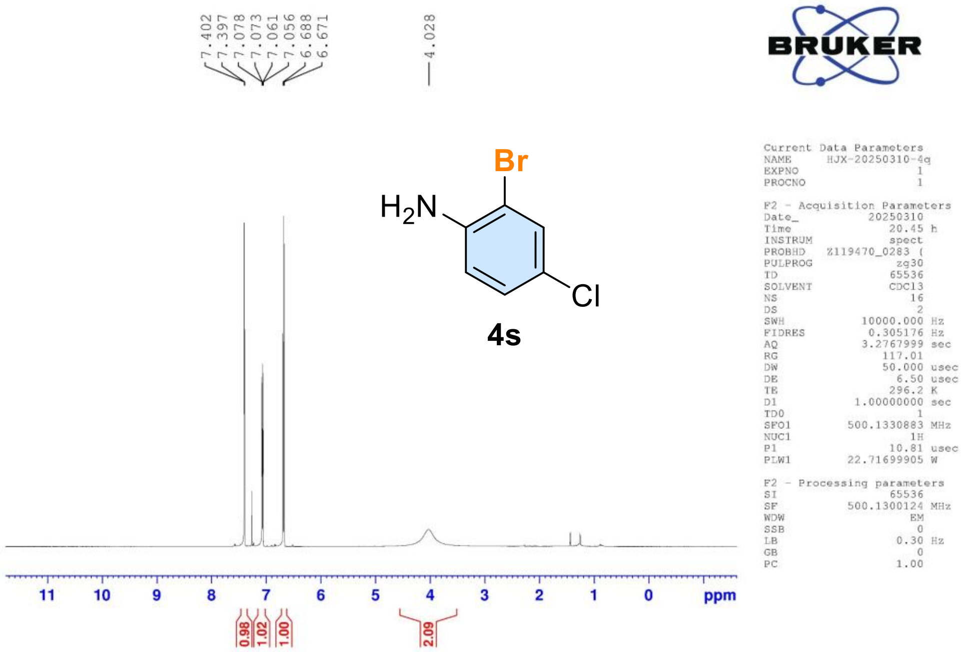

^{13}C NMR

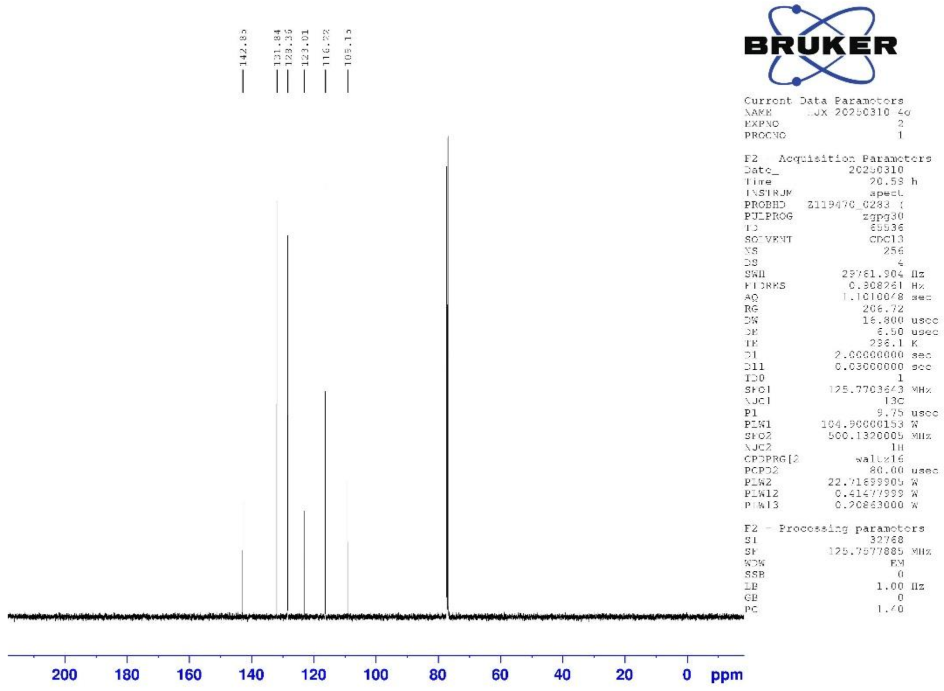

^{1}H NMR

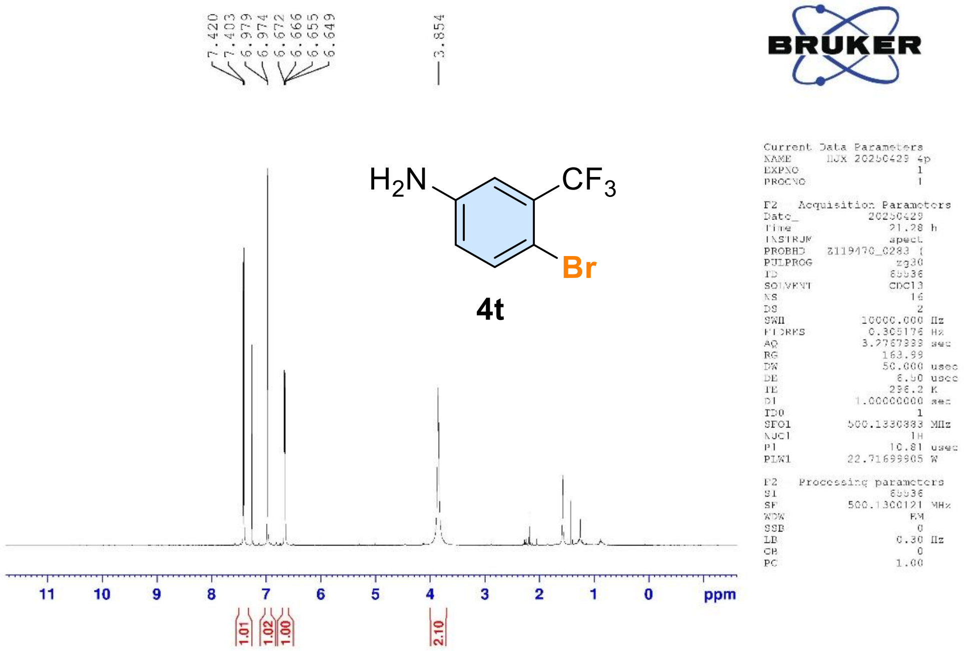

^{13}C NMR

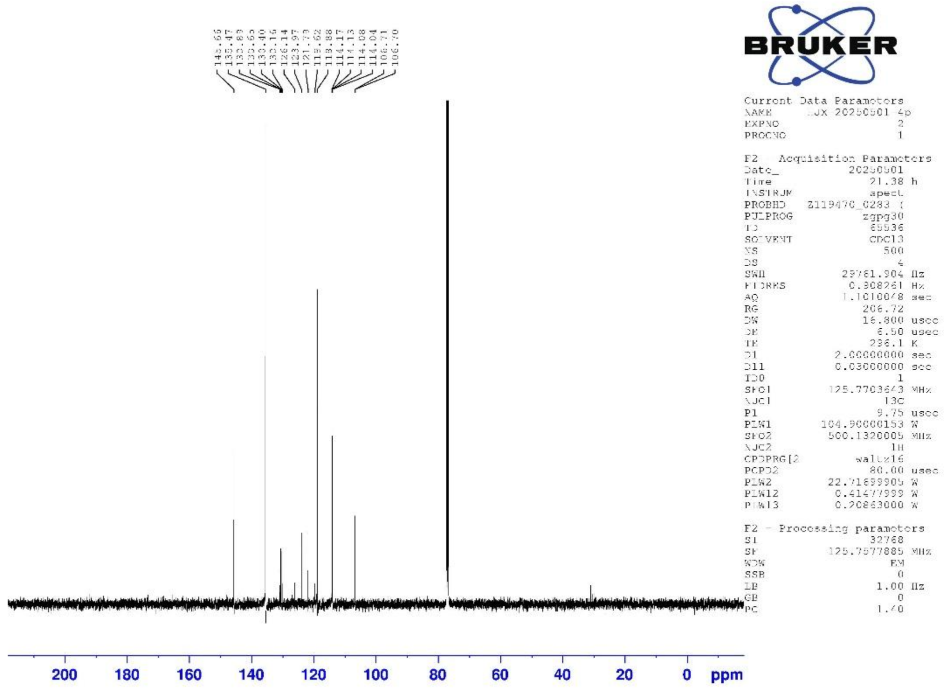

^{19}F NMR

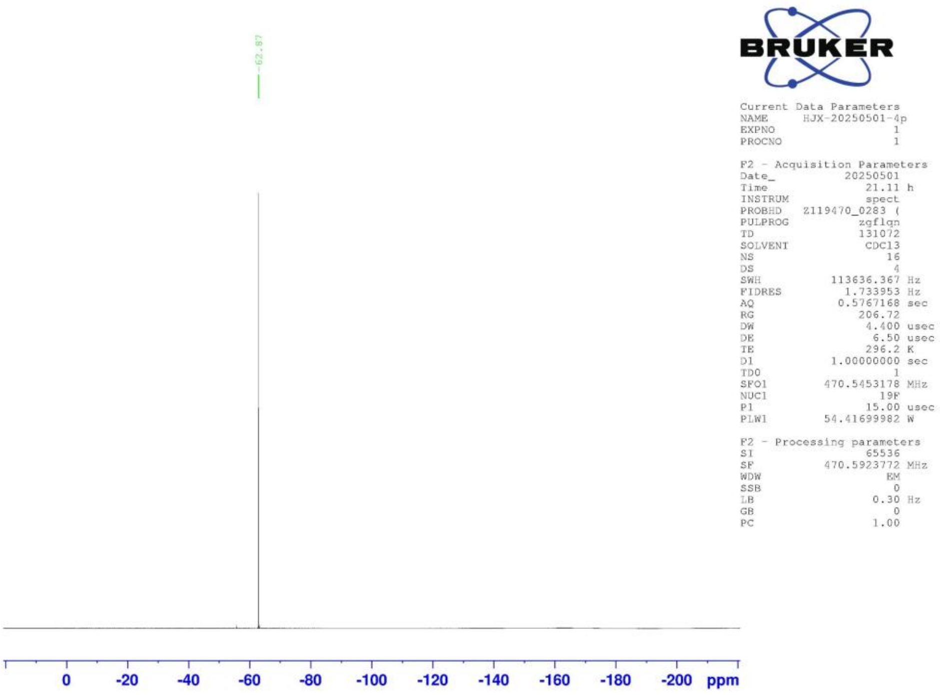

^{1}H NMR

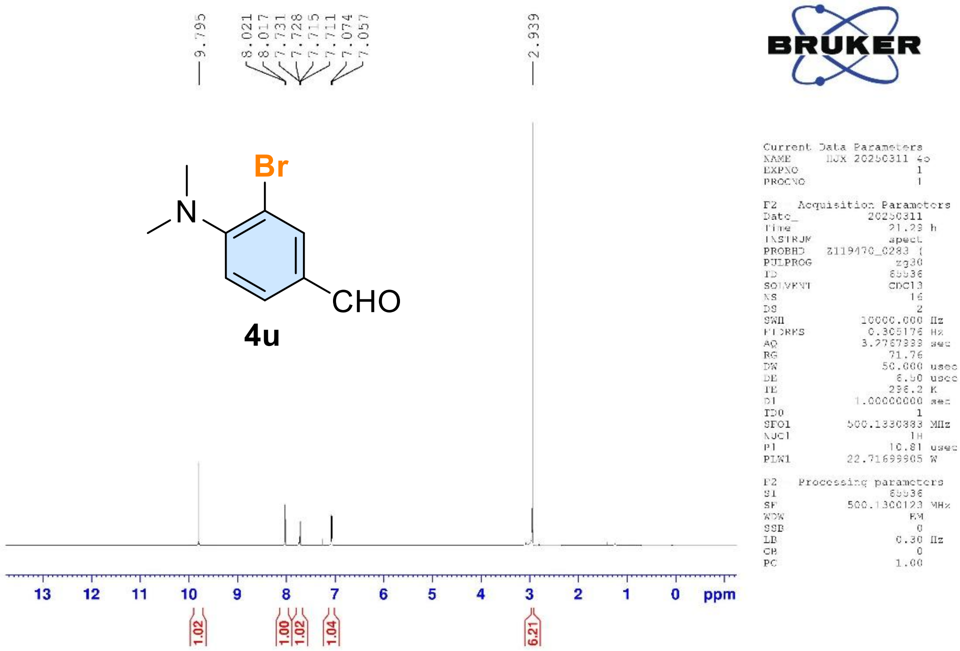

^{13}C NMR

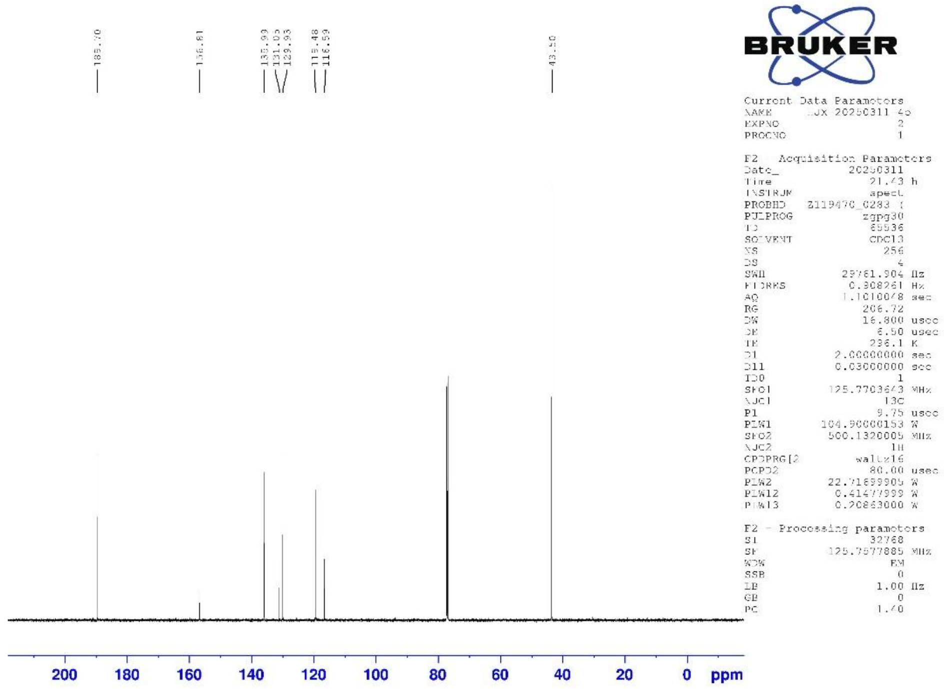

^{1}H NMR

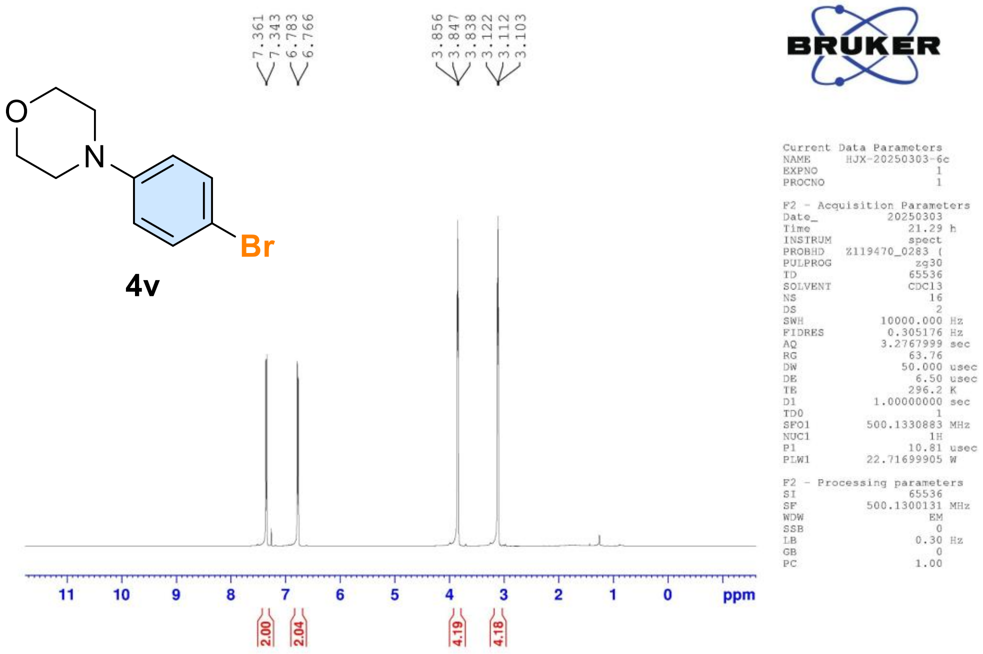

^{13}C NMR

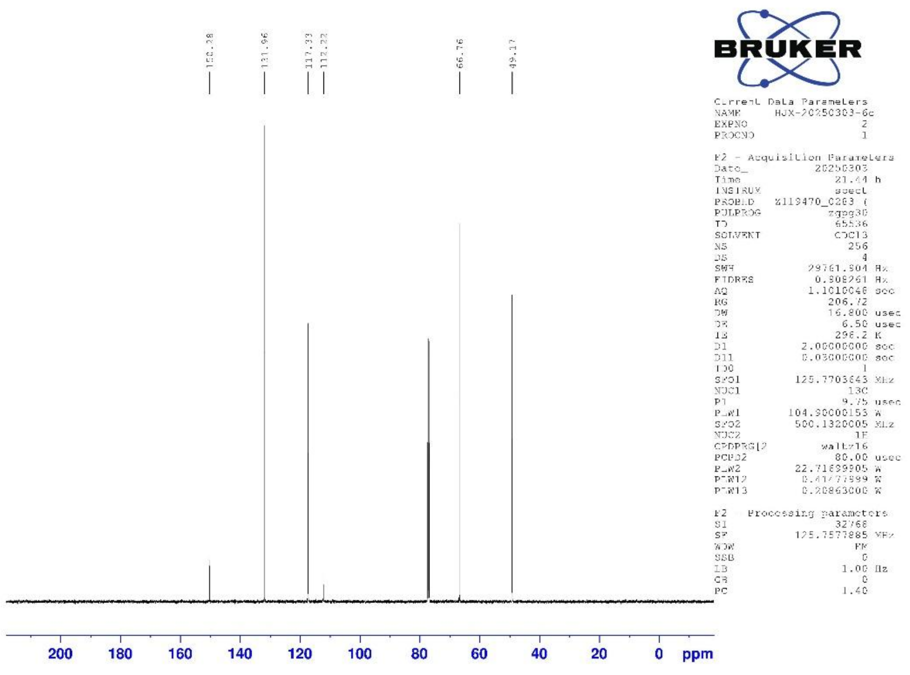

^{1}H NMR

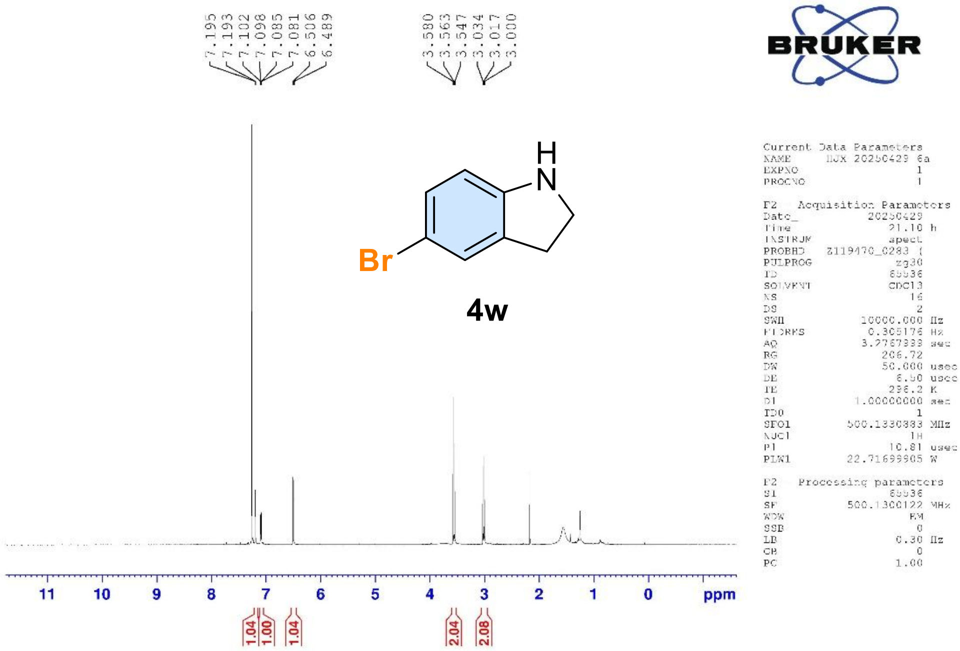

^{13}C NMR

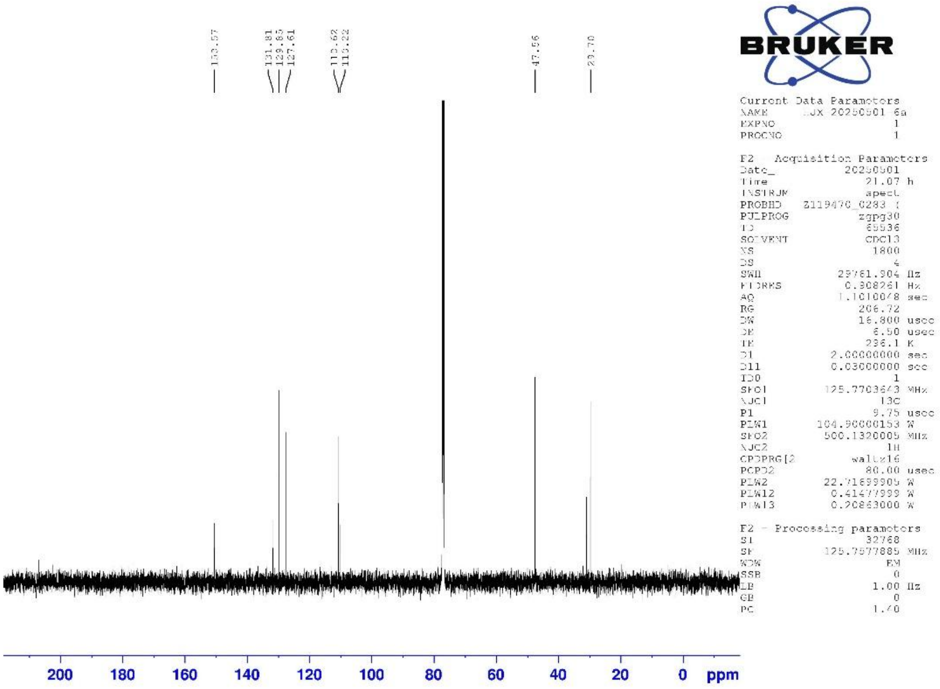

^{1}H NMR

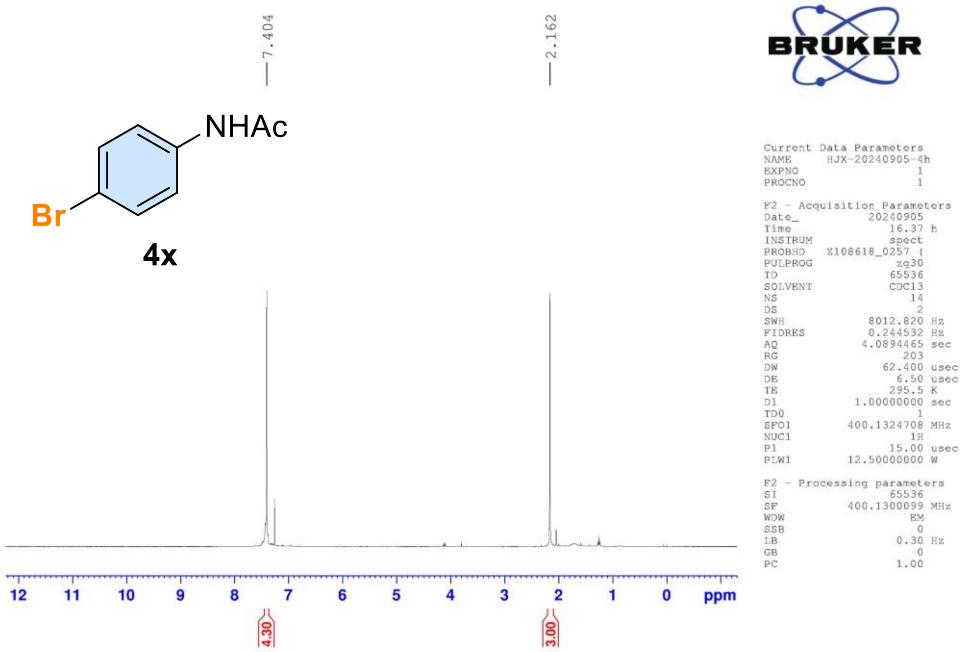

^{13}C NMR

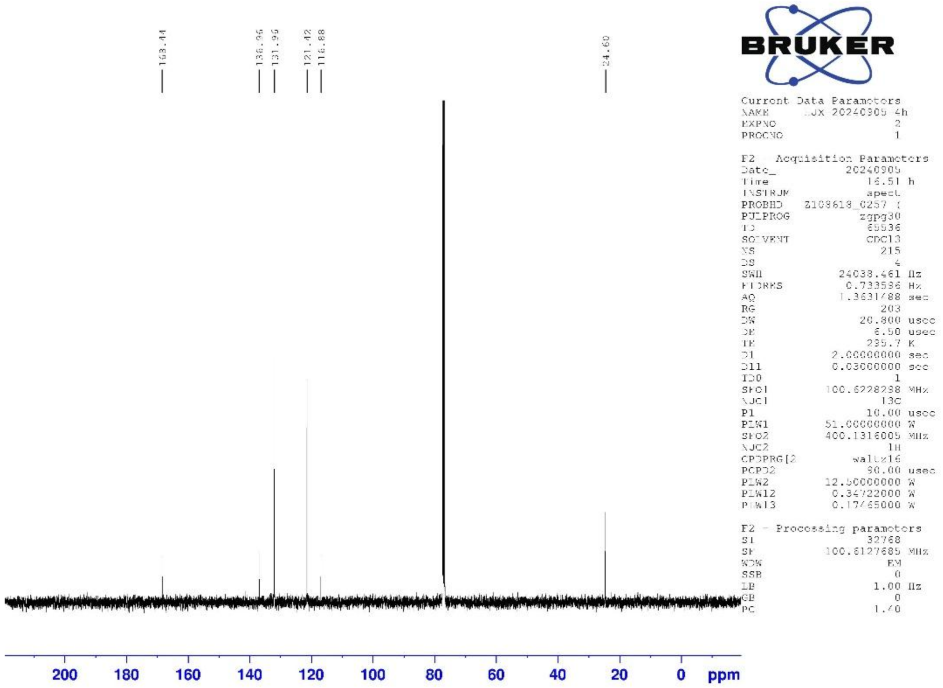

^{1}H NMR

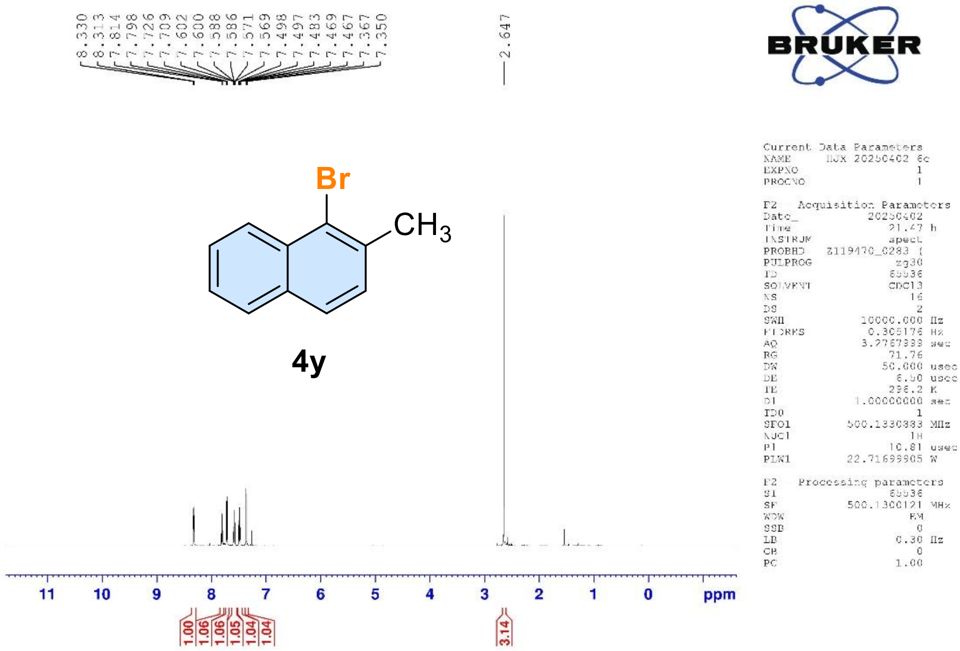

^{13}C NMR

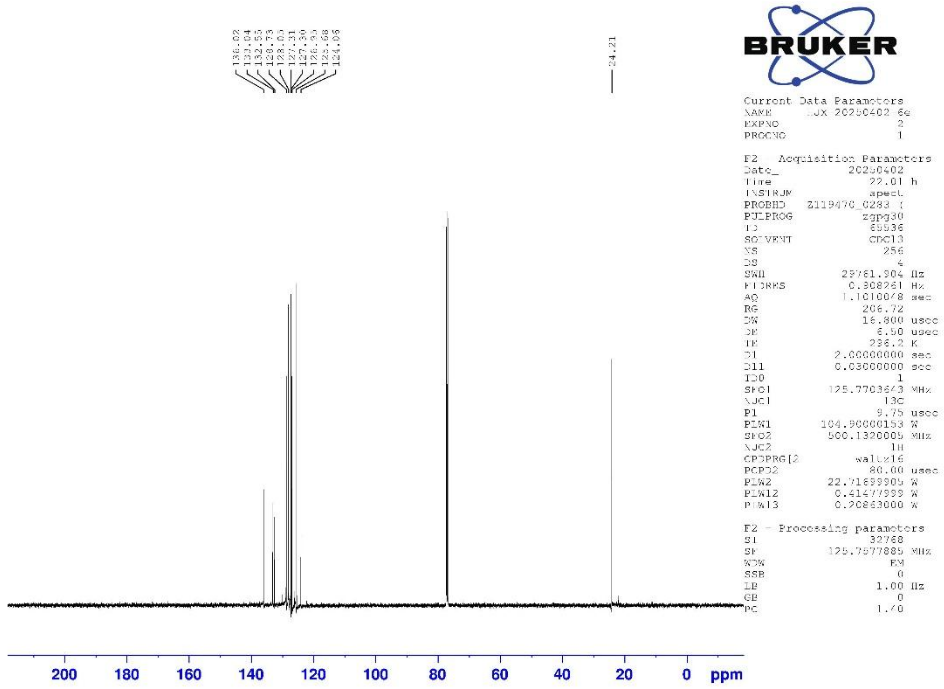

^{1}H NMR

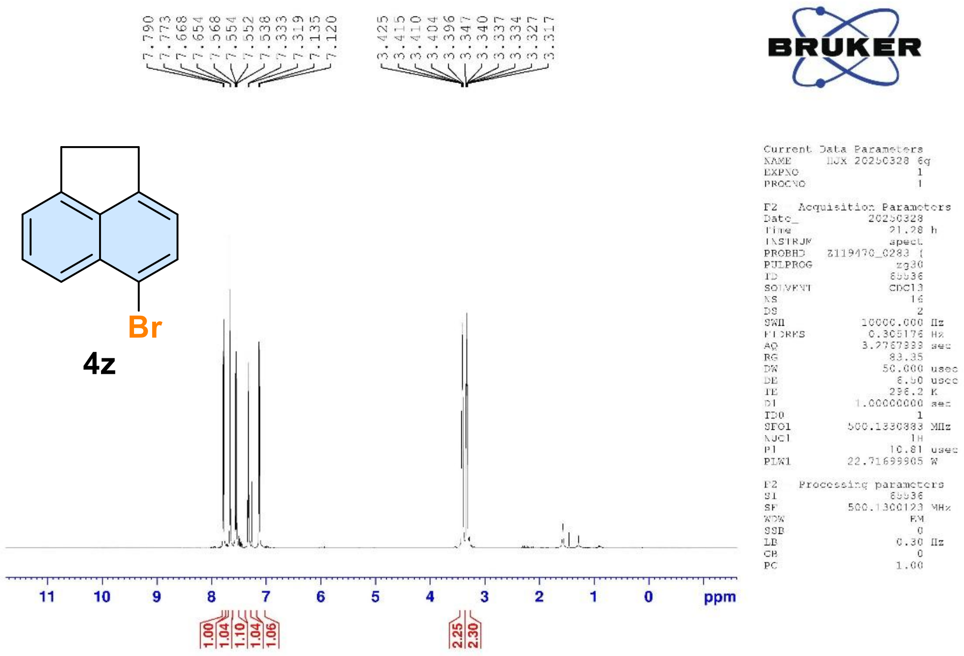

^{13}C NMR

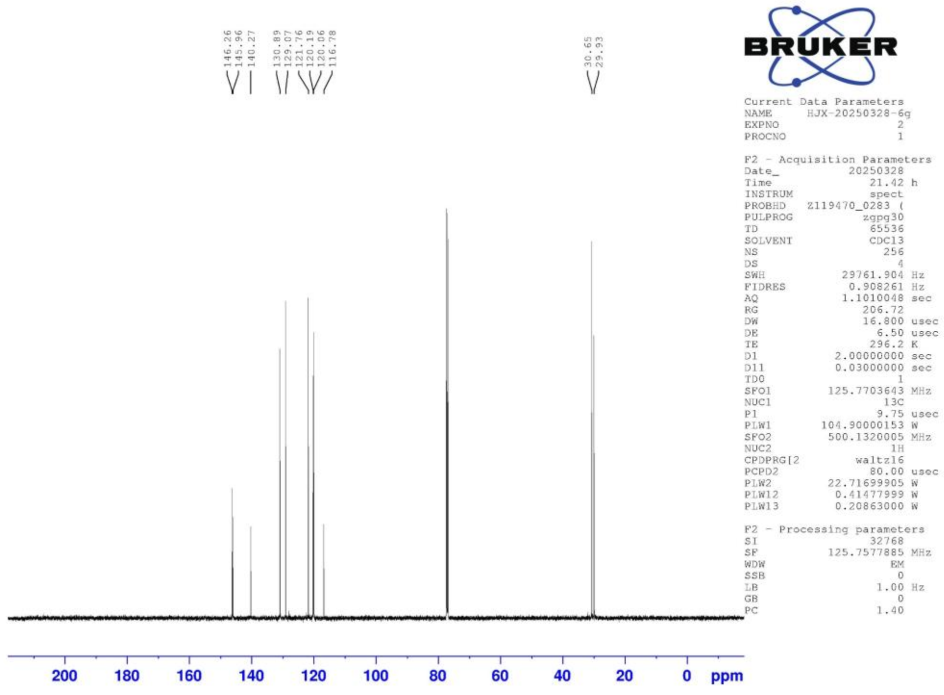

^{1}H NMR

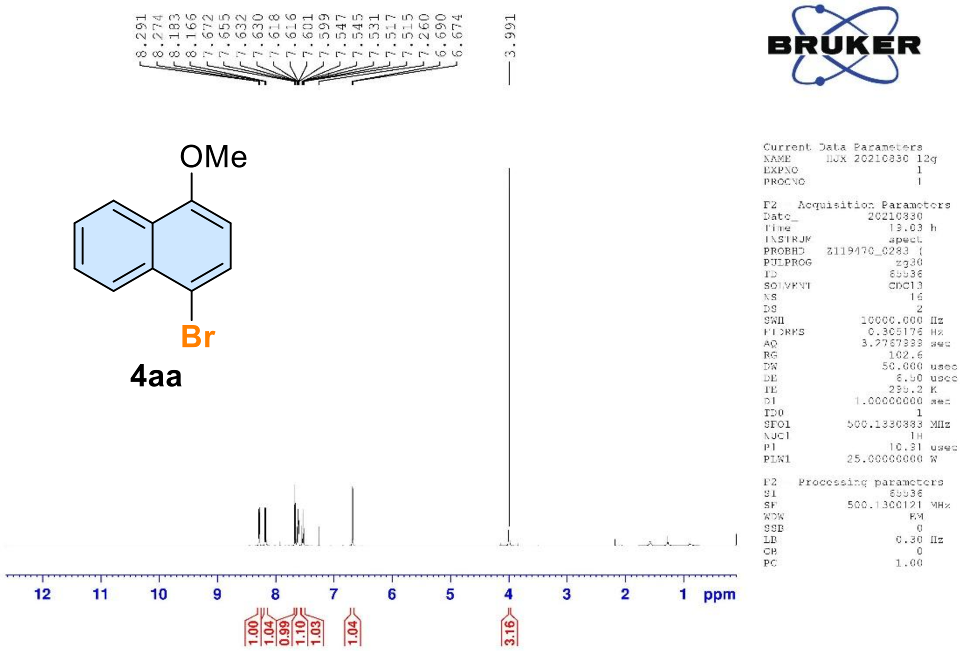

^{13}C NMR

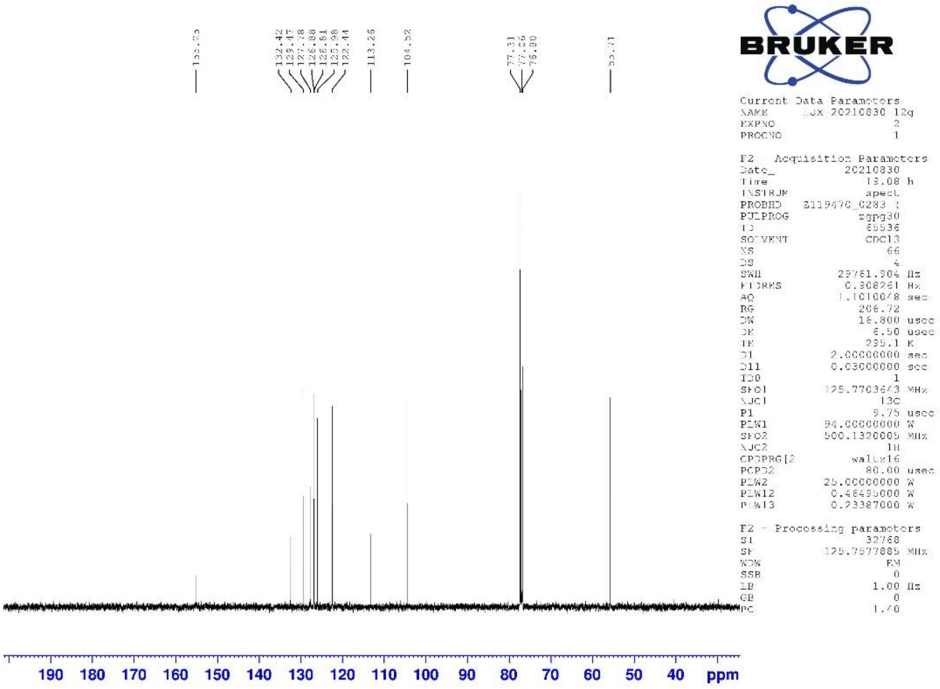

^{1}H NMR

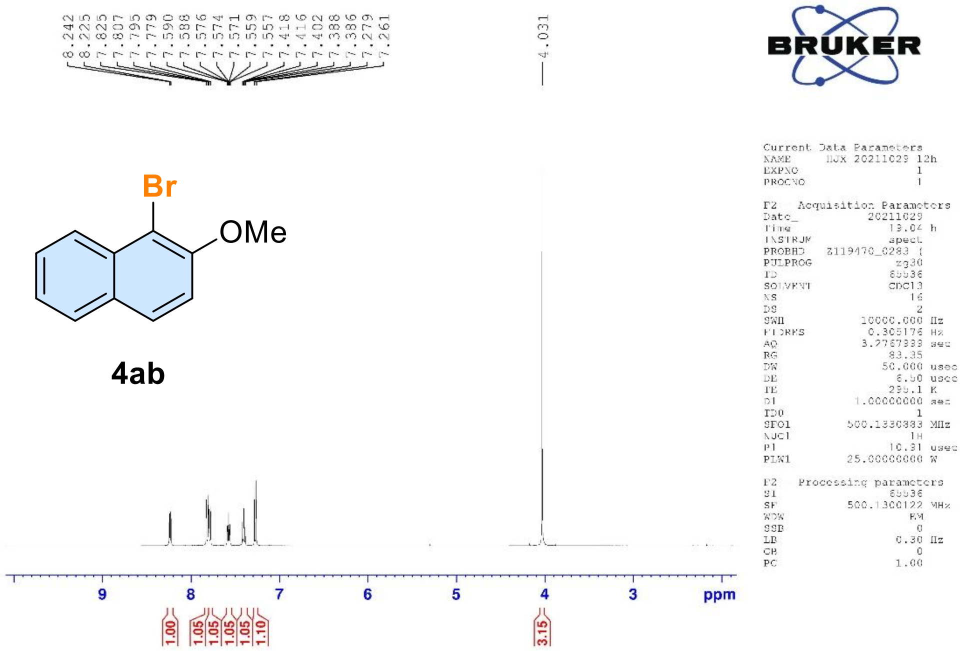

^{13}C NMR

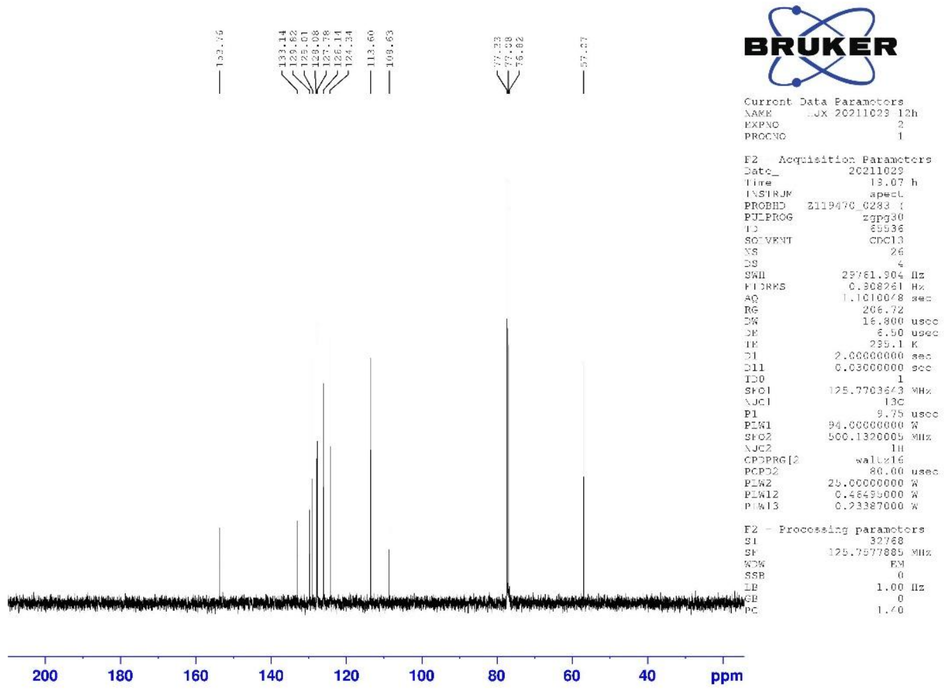

^{1}H NMR

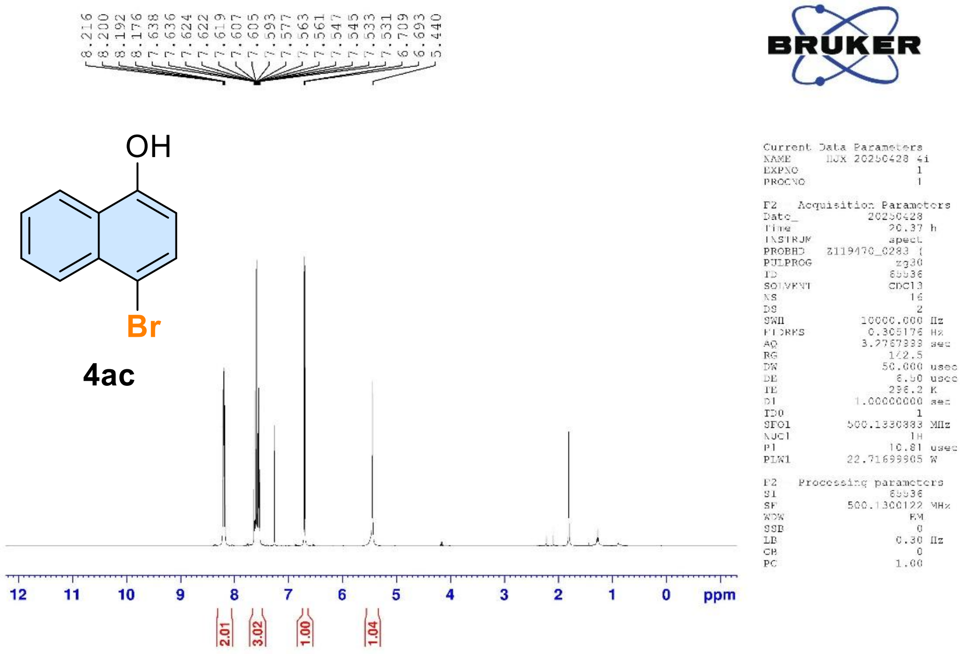

^{13}C NMR

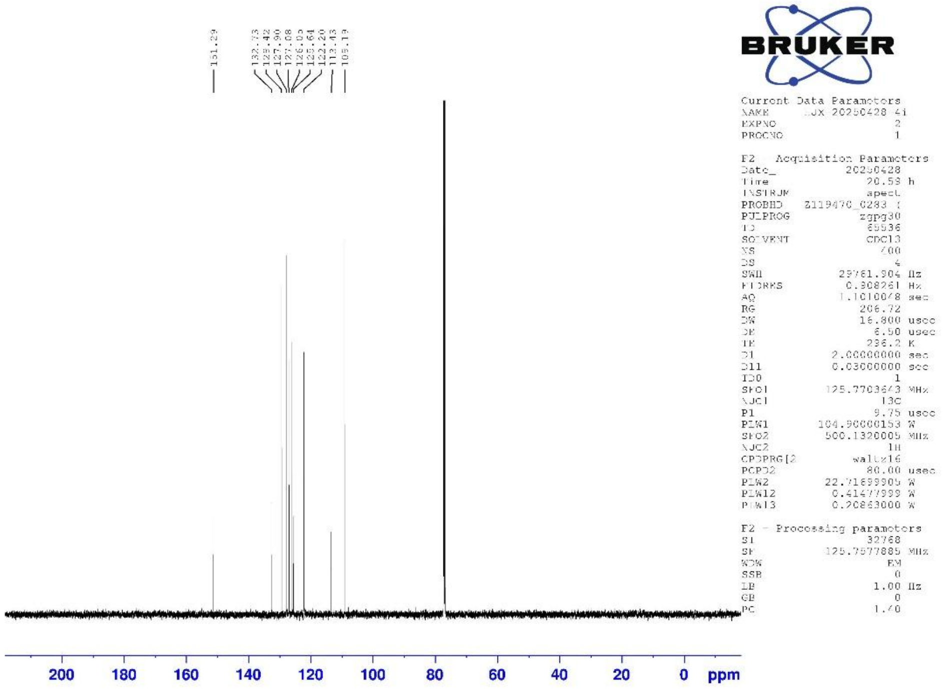

^{1}H NMR

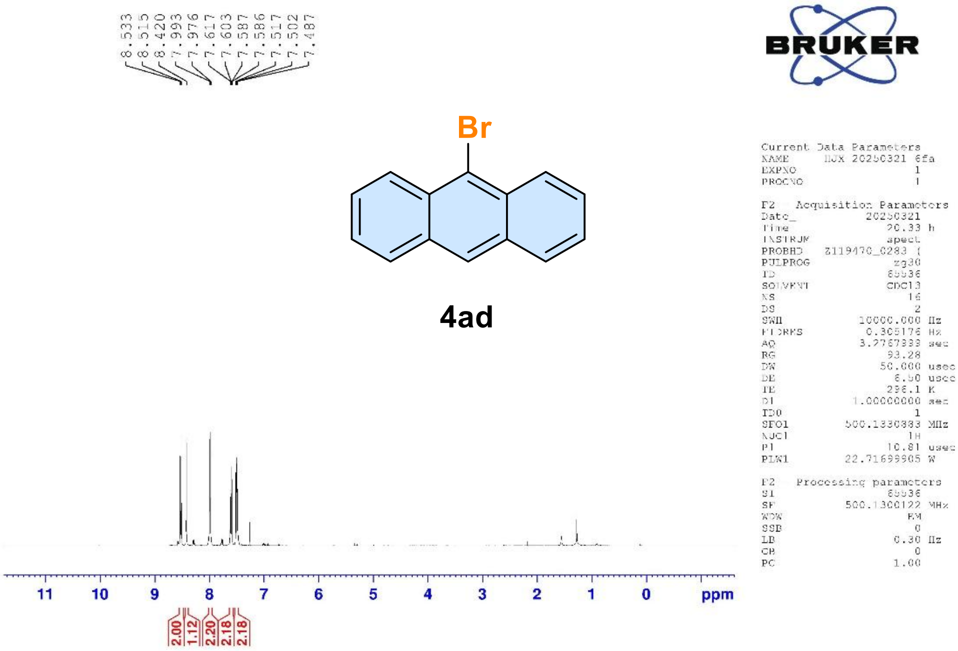

4ad

^{13}C NMR

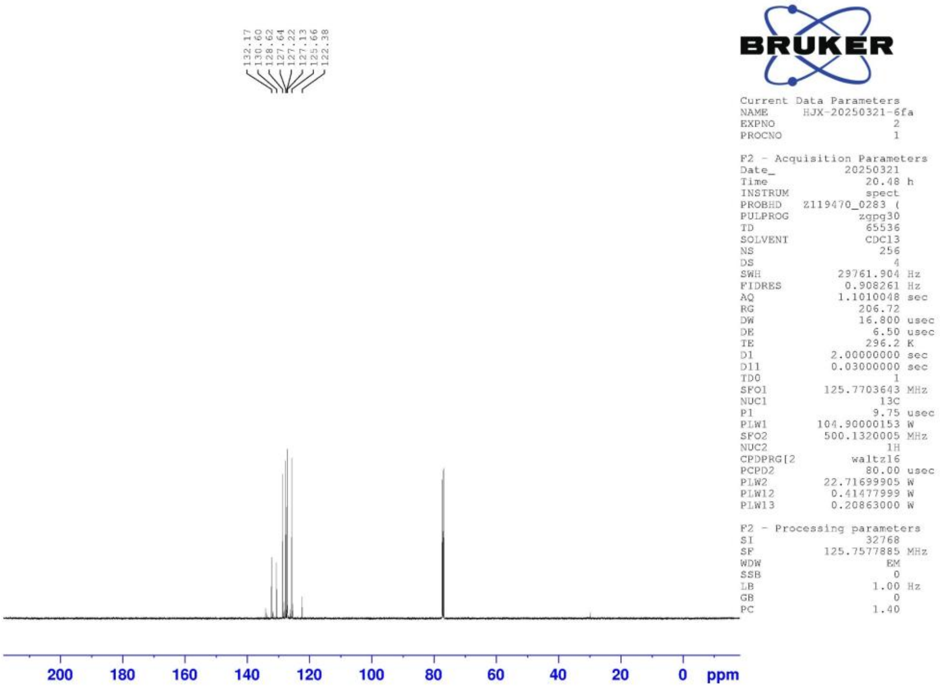

^{1}H NMR

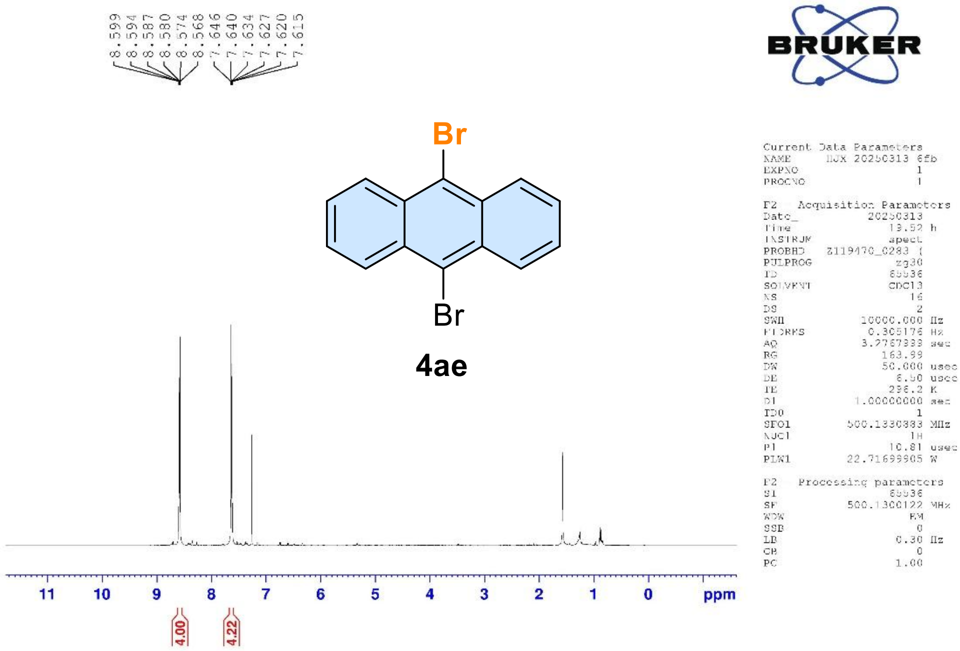

^{13}C NMR

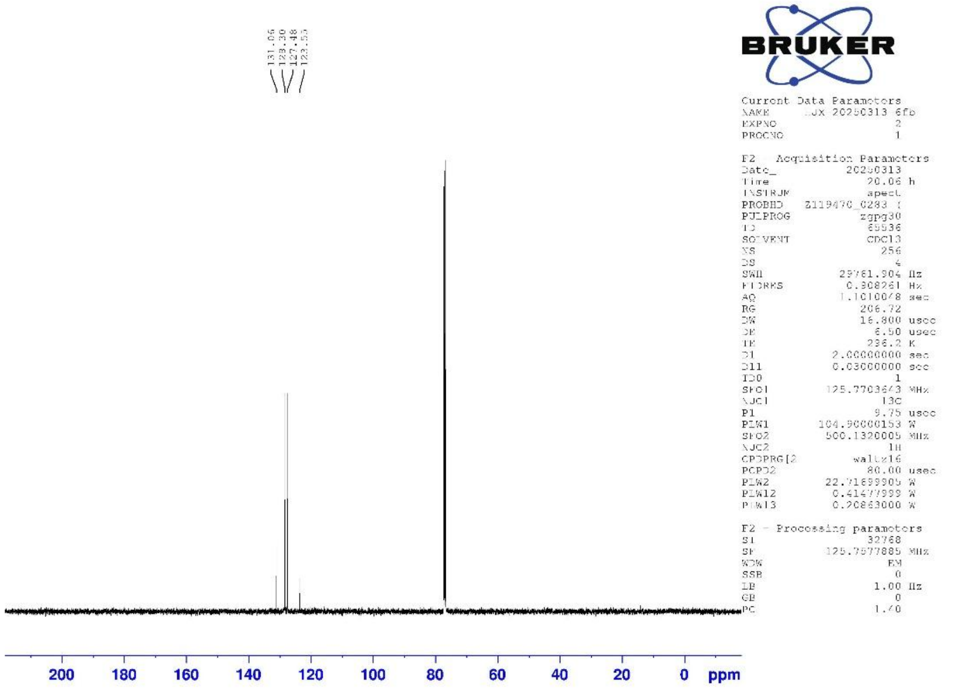

^{1}H NMR

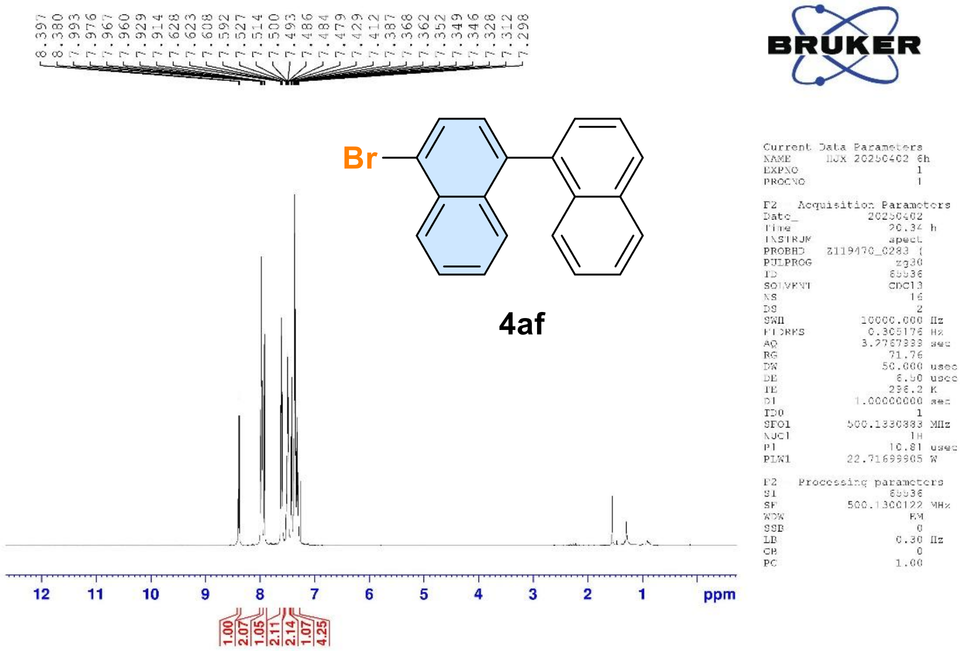

^{13}C NMR

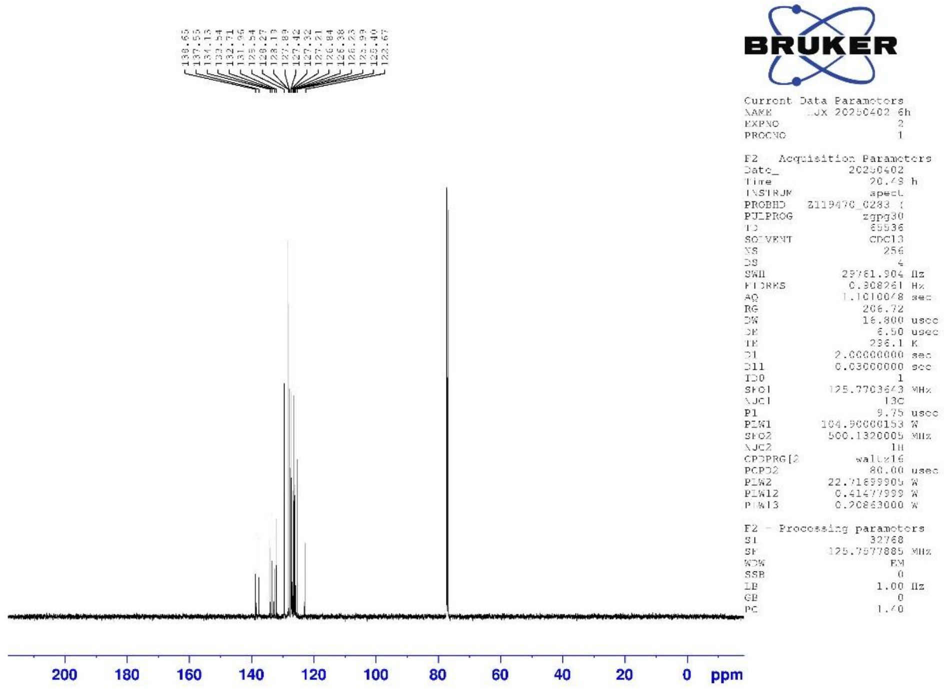

^{1}H NMR

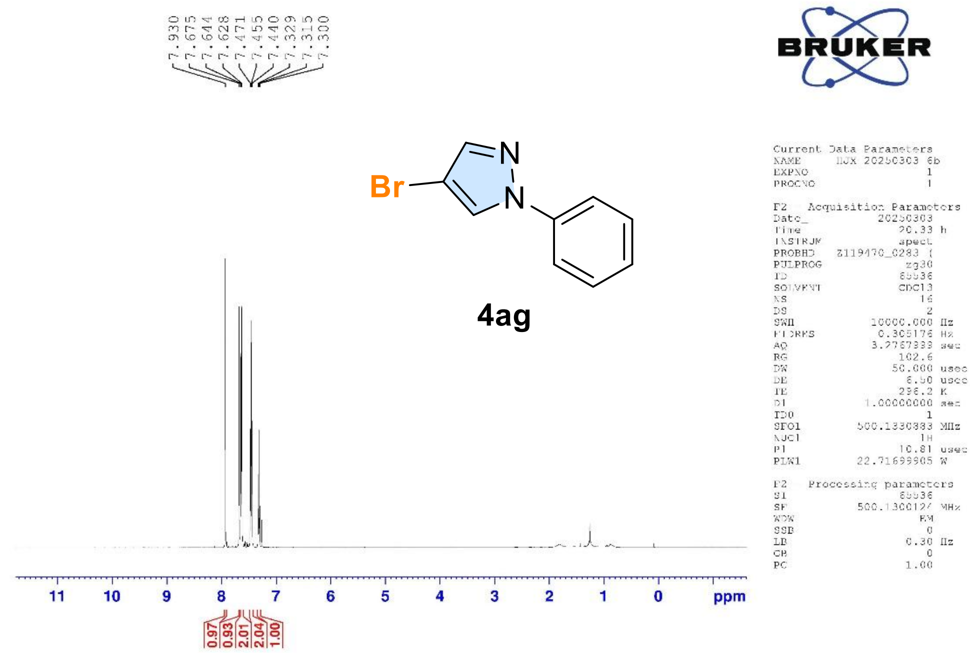

^{13}C NMR

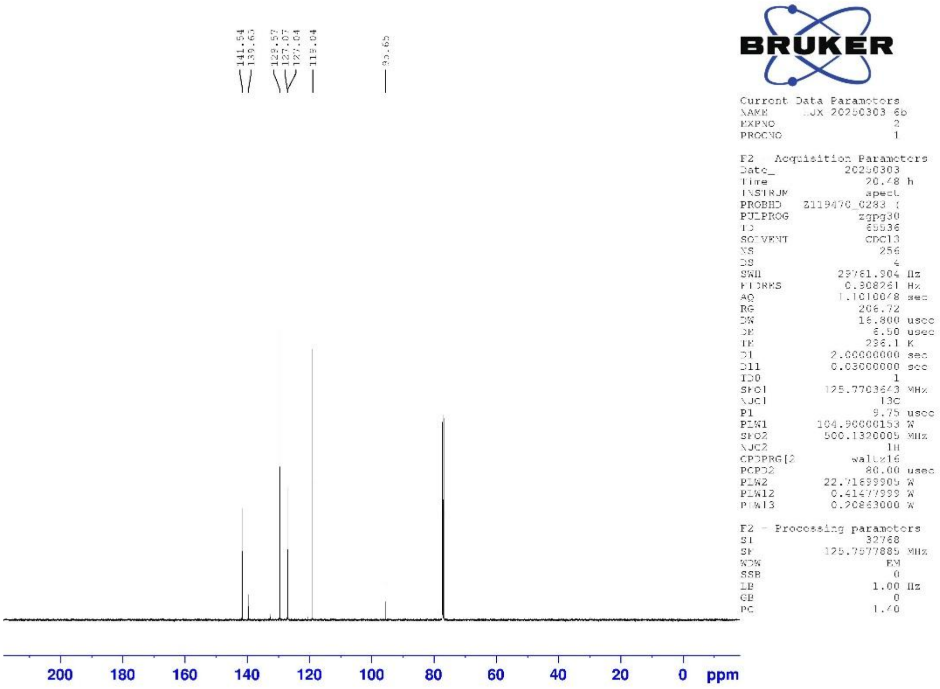

^{1}H NMR

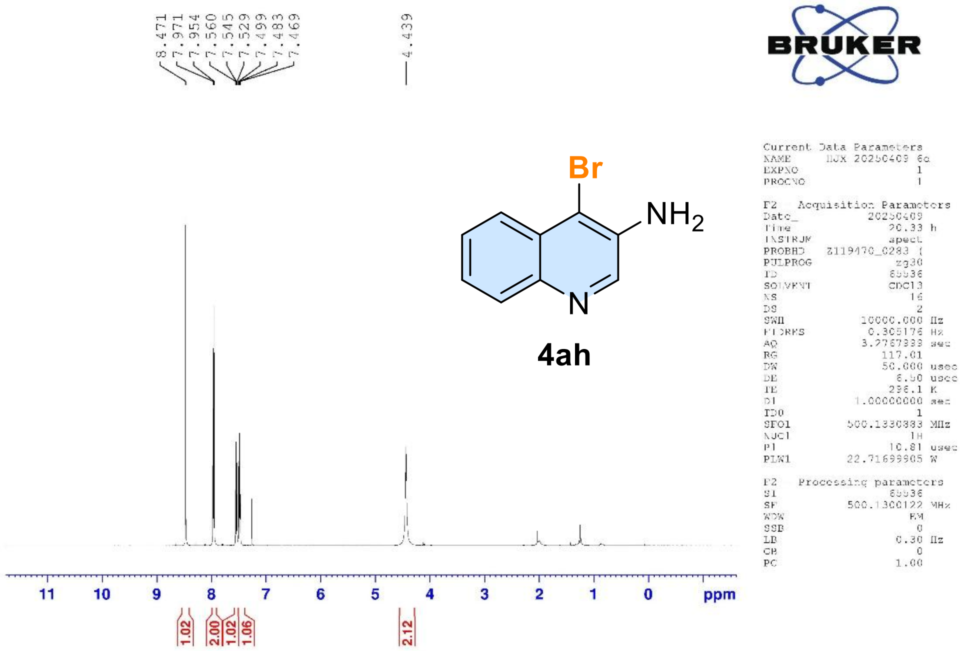

^{13}C NMR

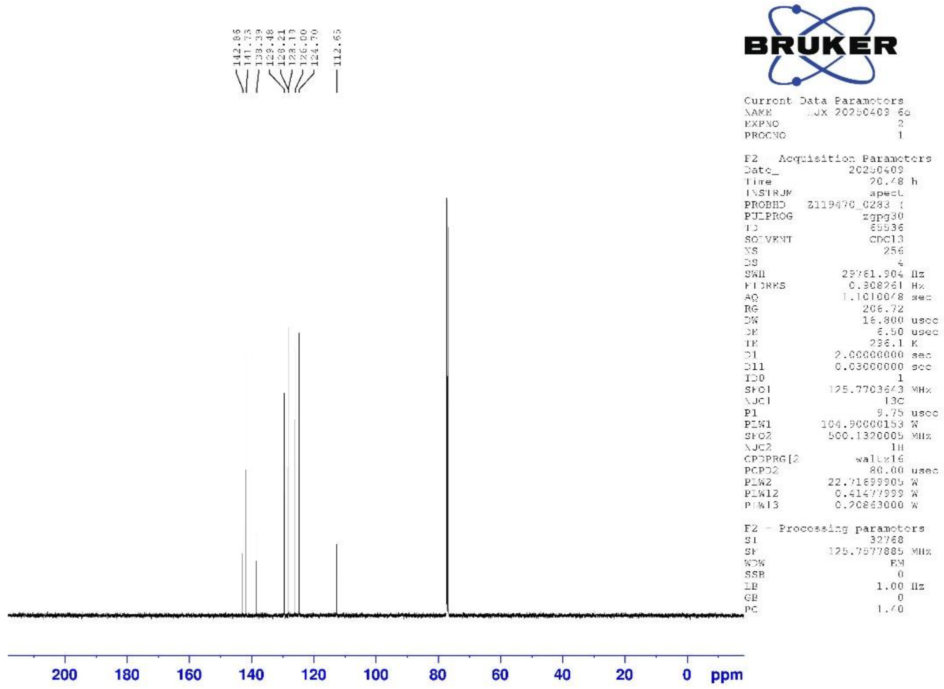

^{1}H NMR

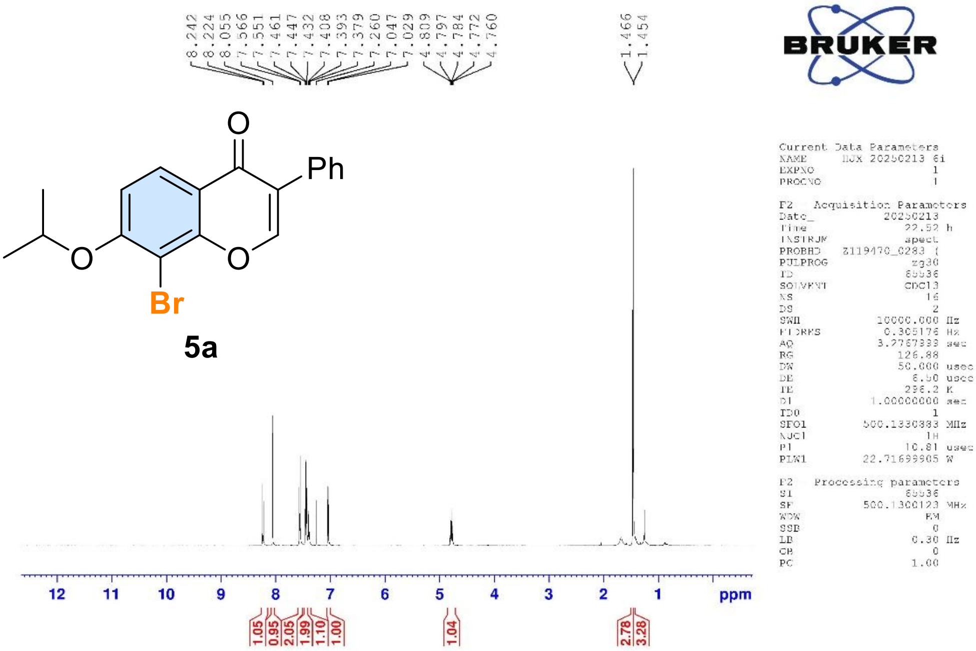

^{13}C NMR

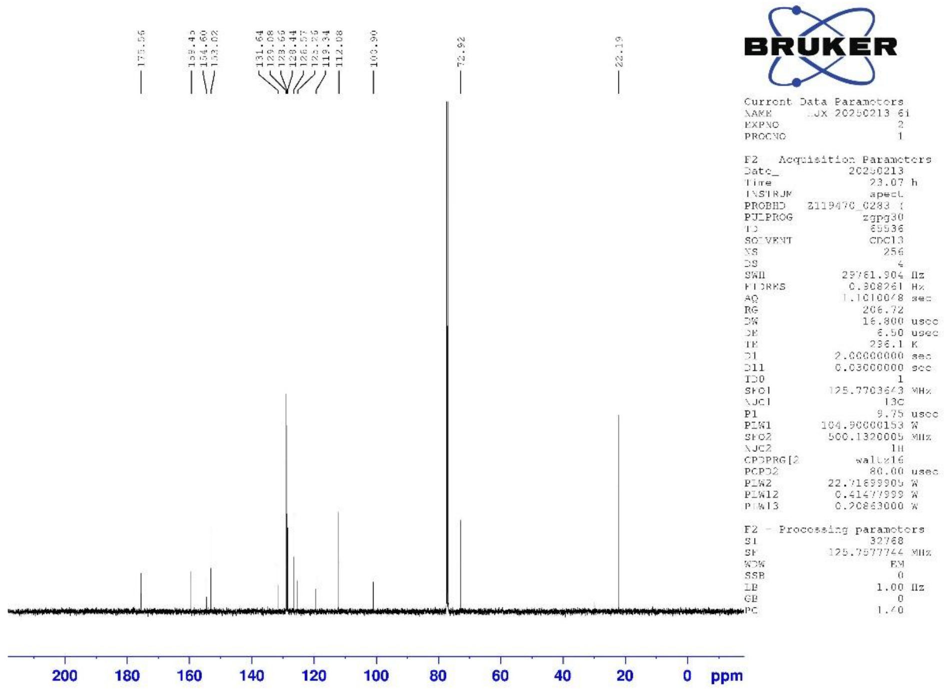

^{1}H NMR

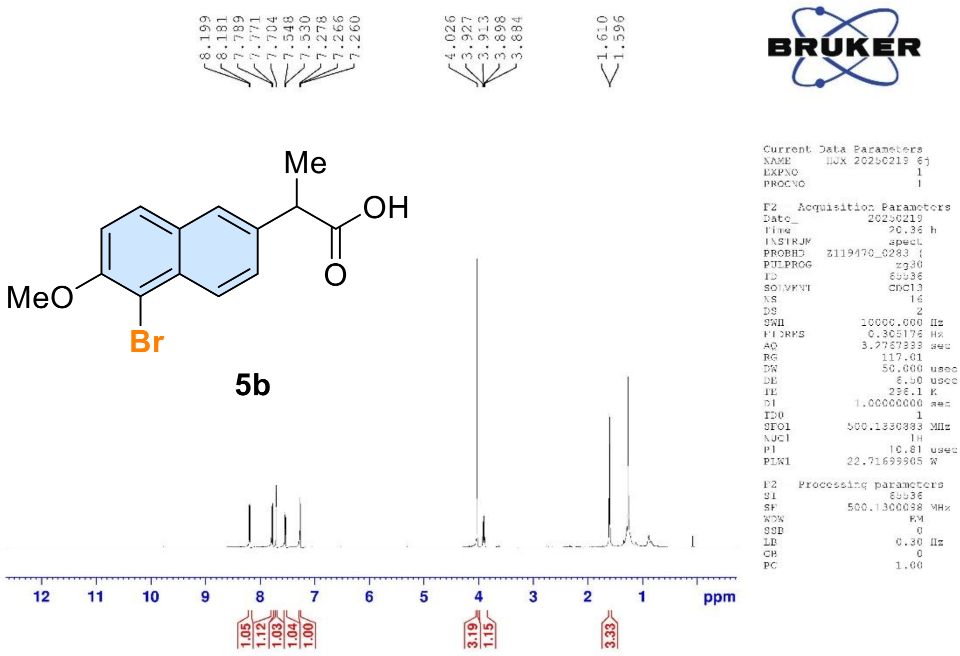

^{13}C NMR

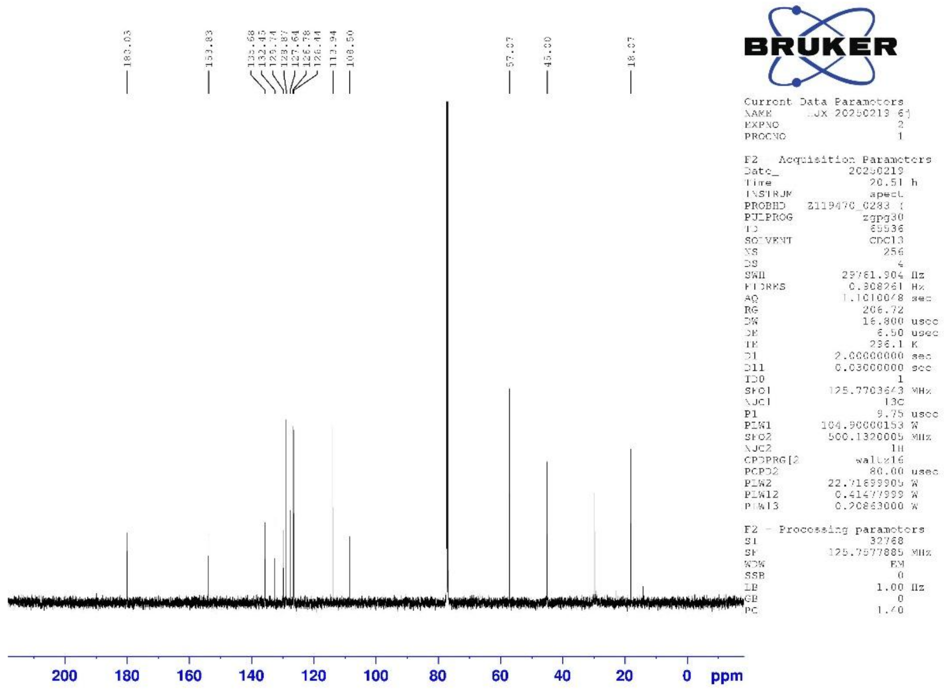

^{1}H NMR

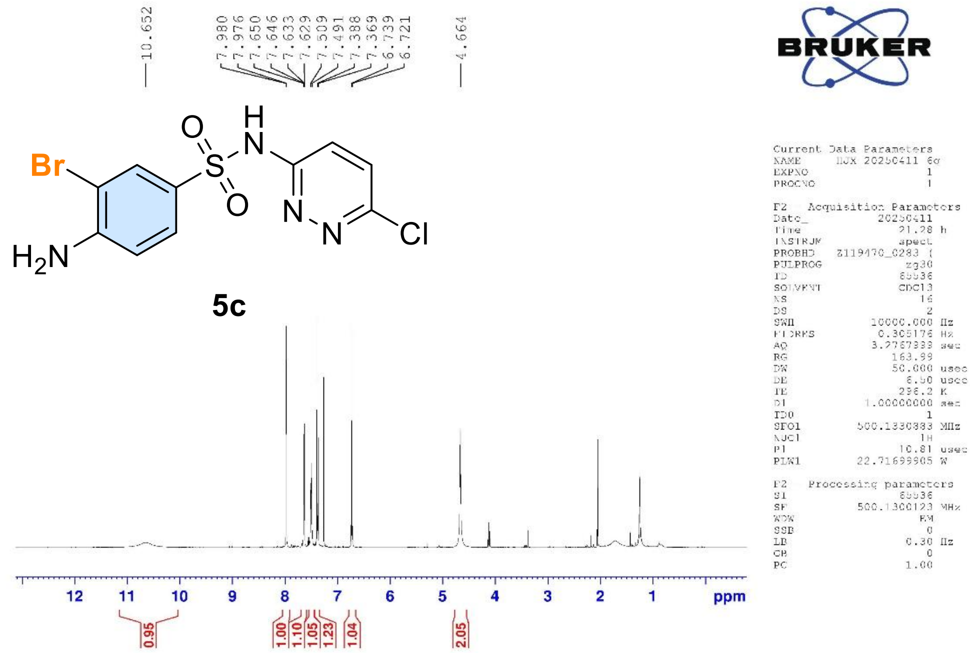

^{13}C NMR

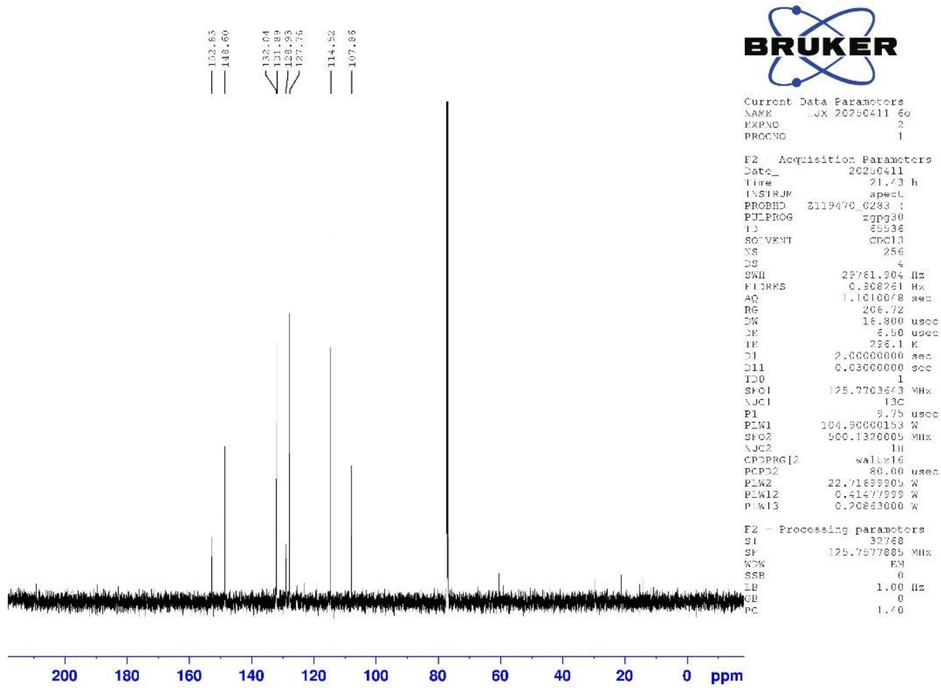

^{1}H NMR

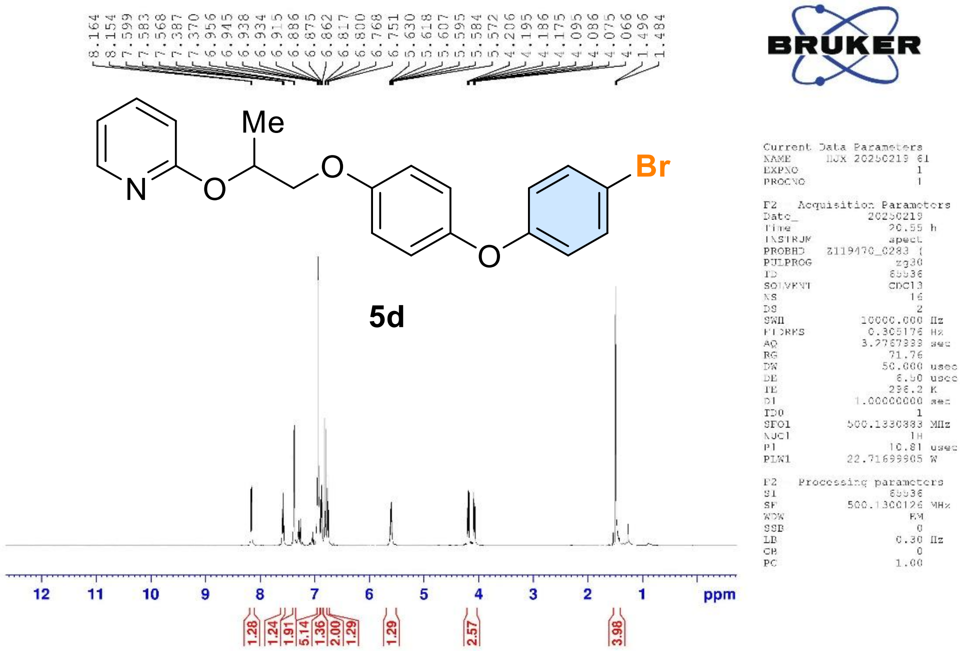

^{13}C NMR

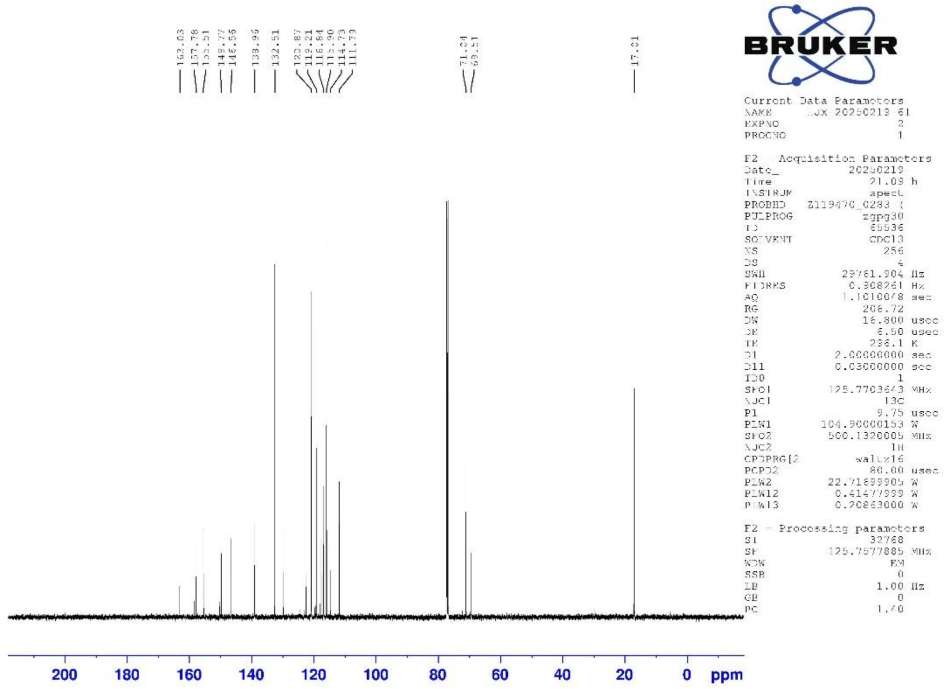

^{1}H NMR

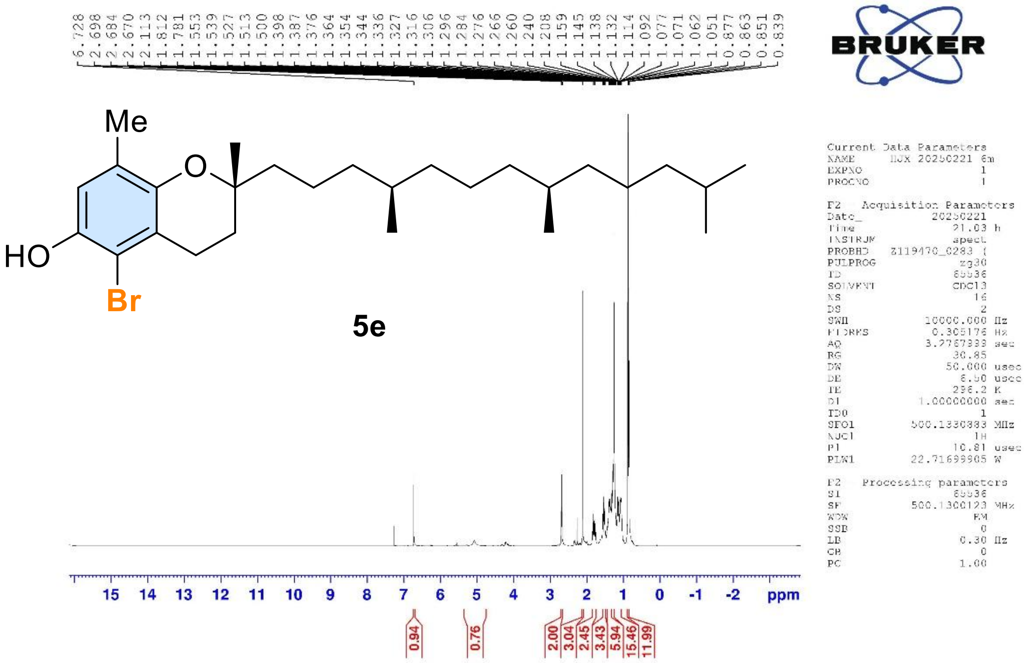

^{13}C NMR

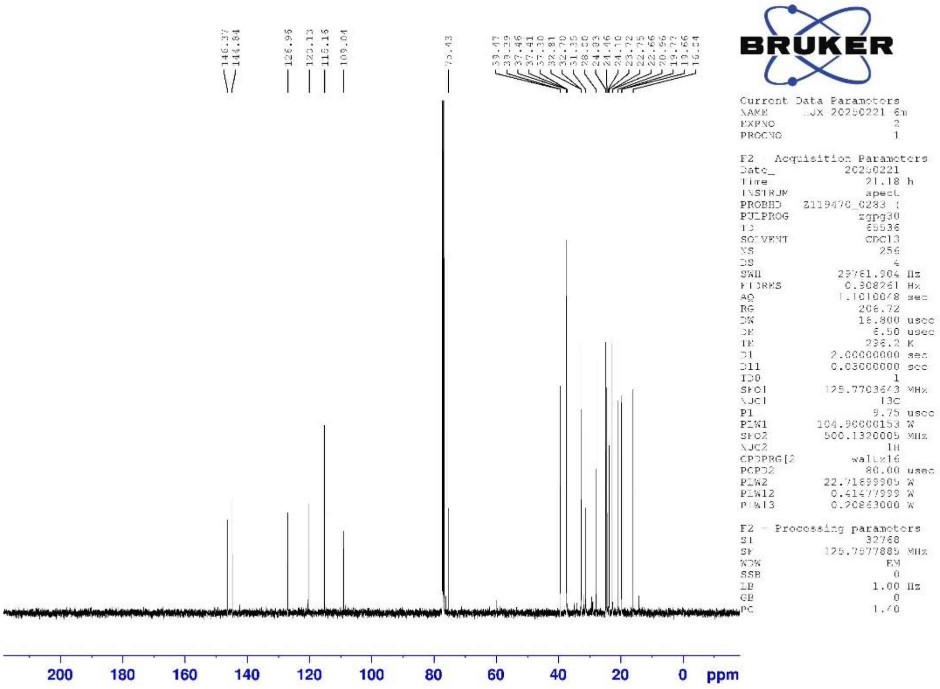

^{1}H NMR

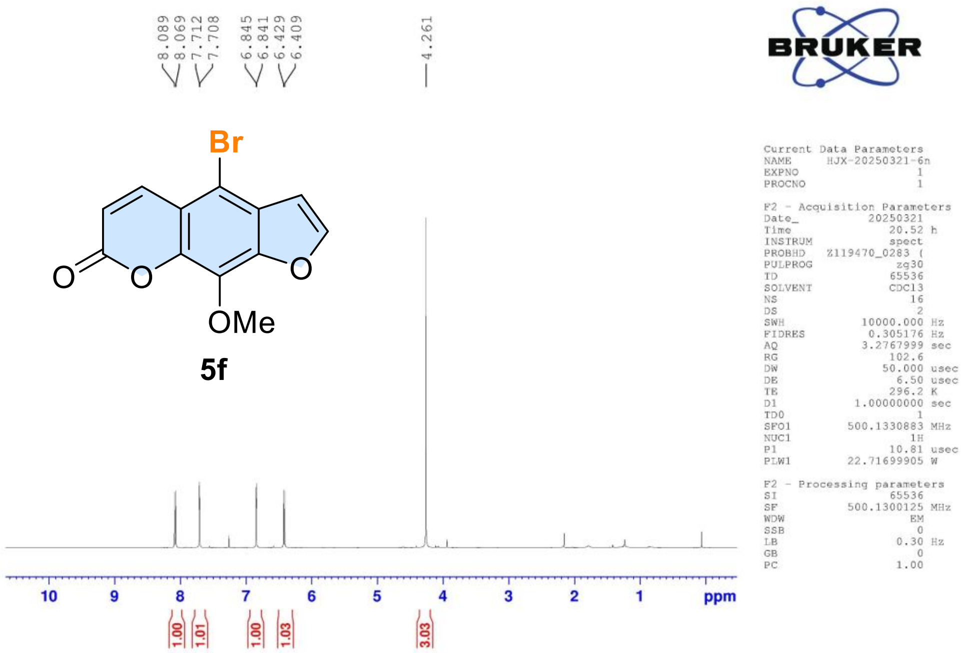

^{13}C NMR

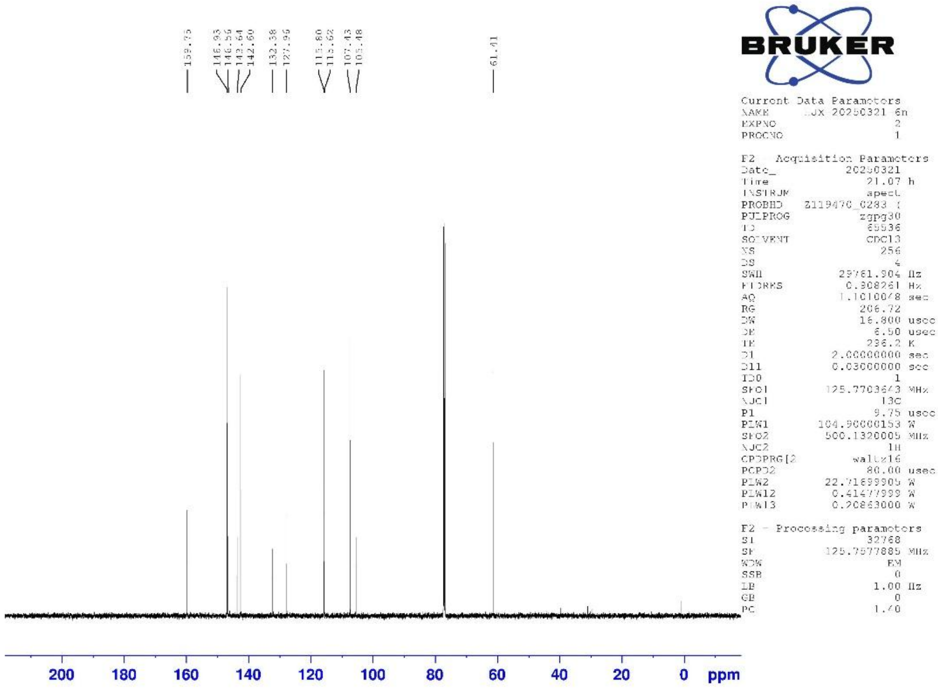

^{1}H NMR

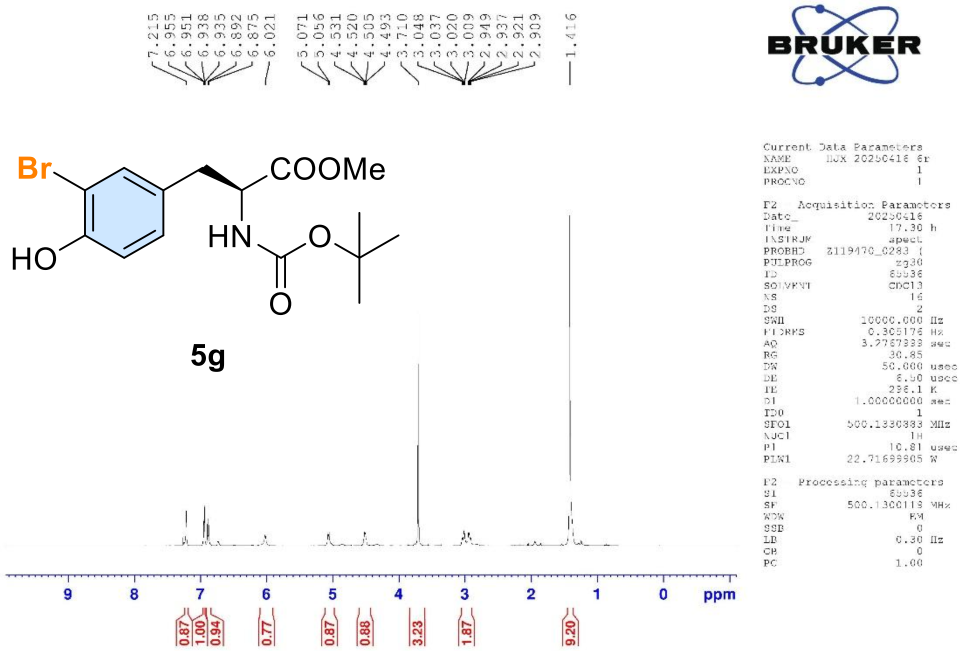

^{13}C NMR

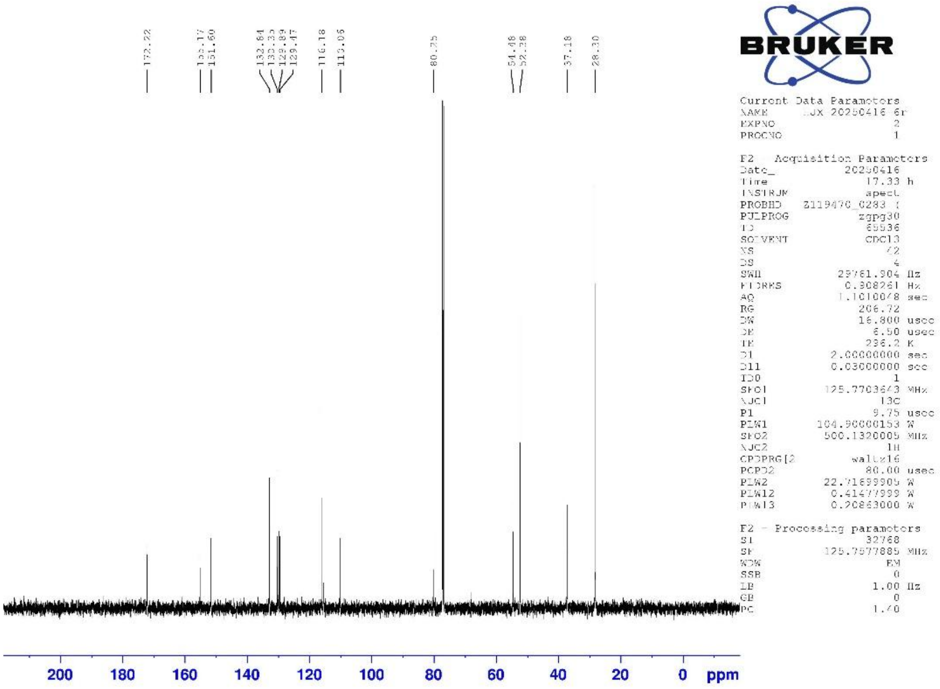

^{1}H NMR

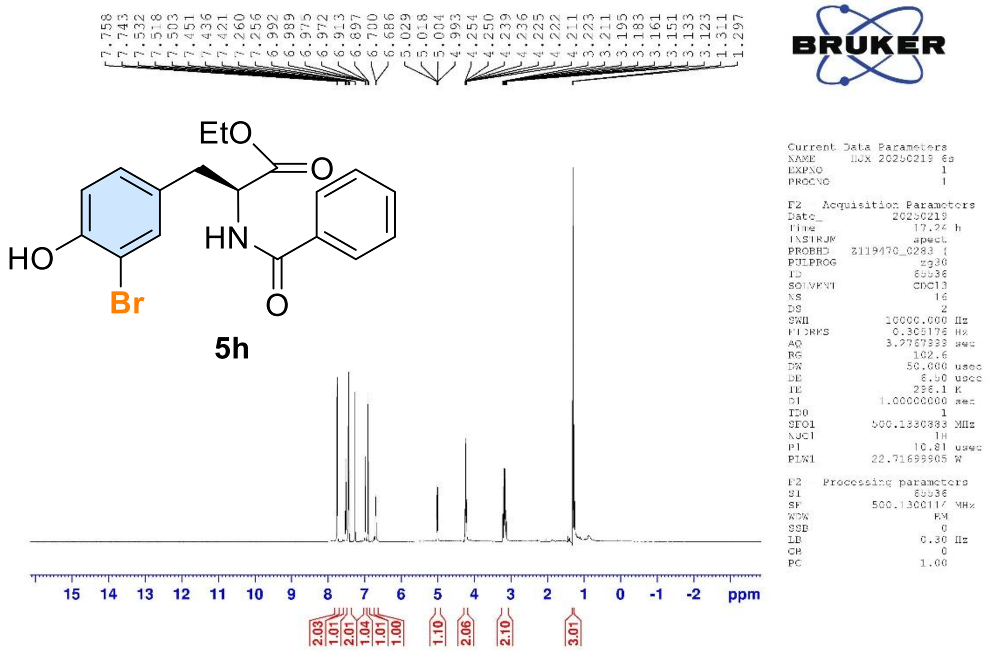

^{13}C NMR

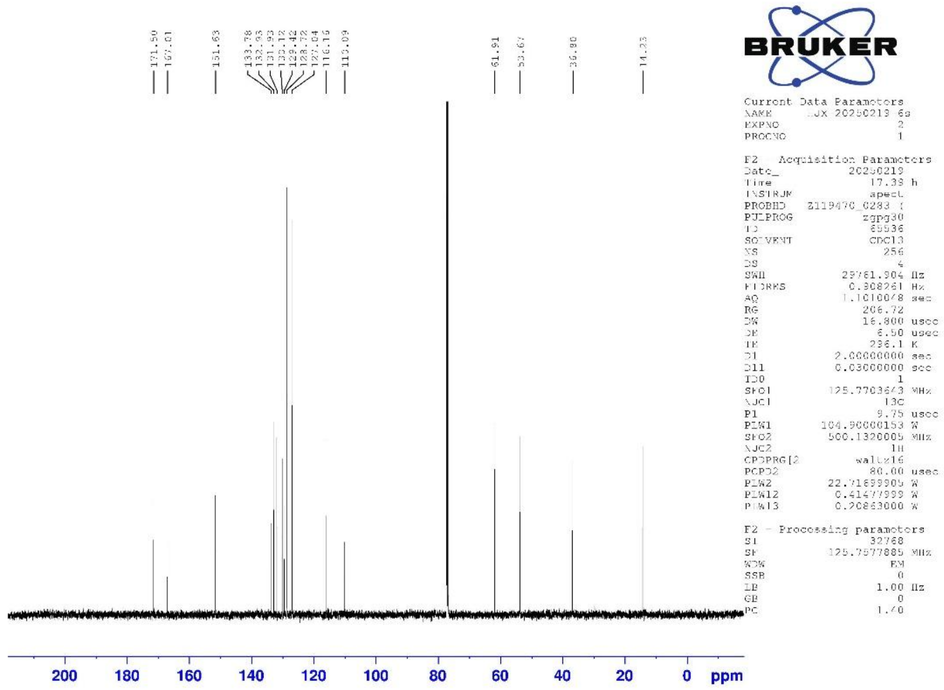

^{1}H NMR

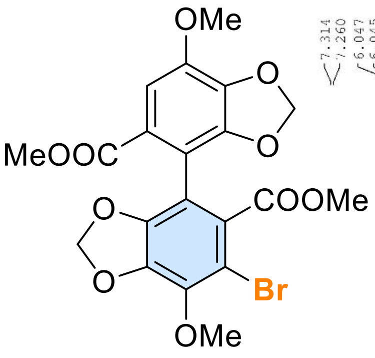

5i

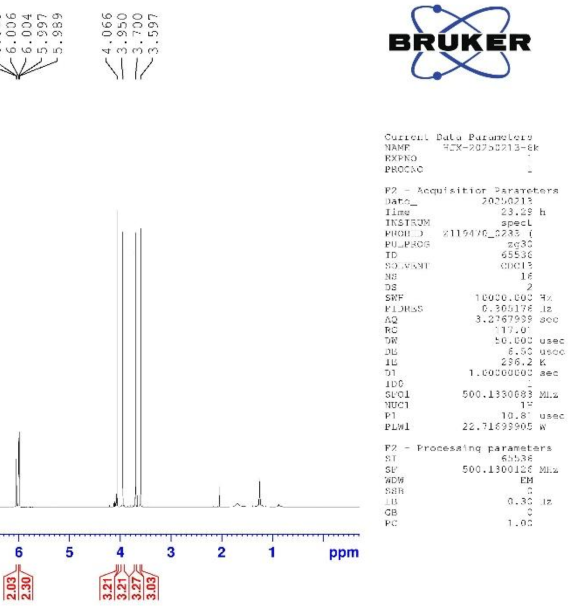

^{13}C NMR

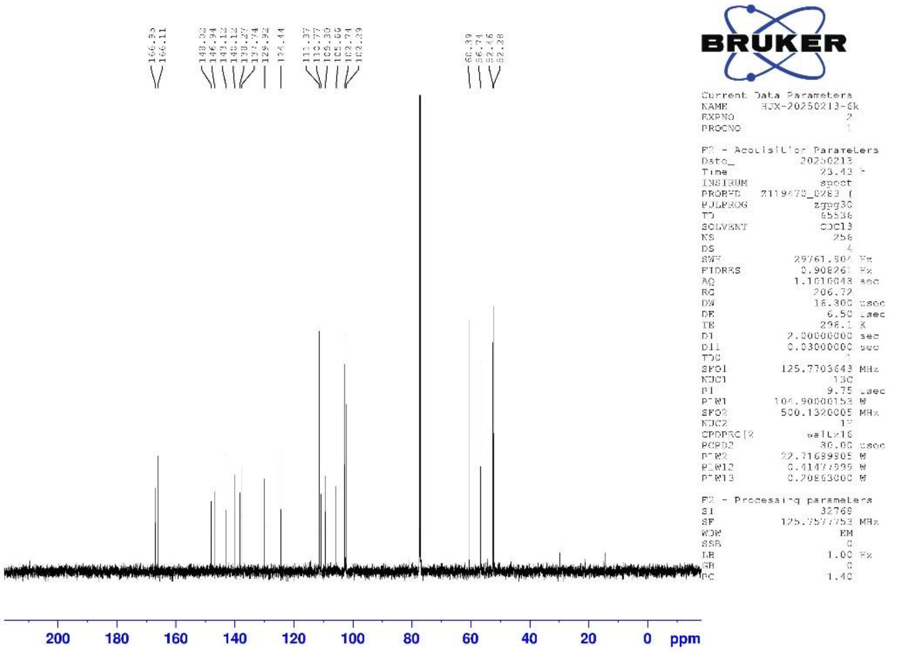

^{1}H NMR

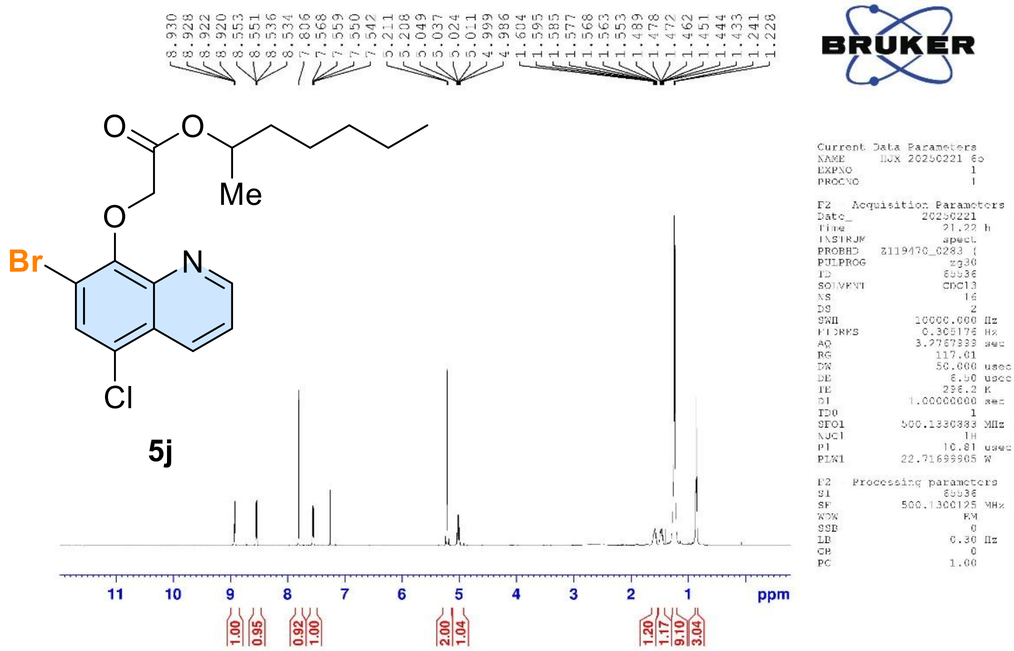

^{13}C NMR

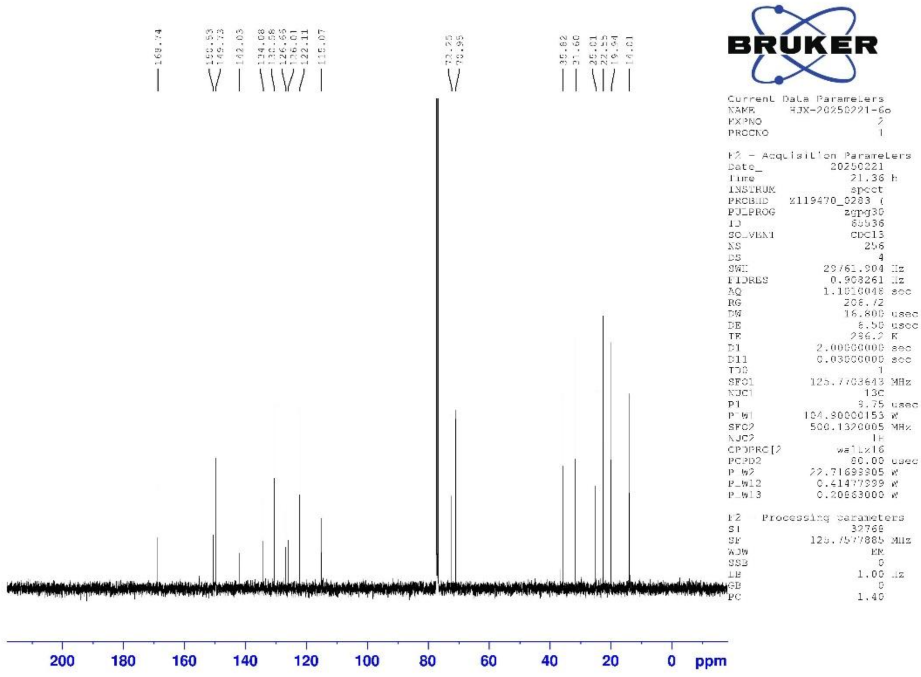

^{1}H NMR

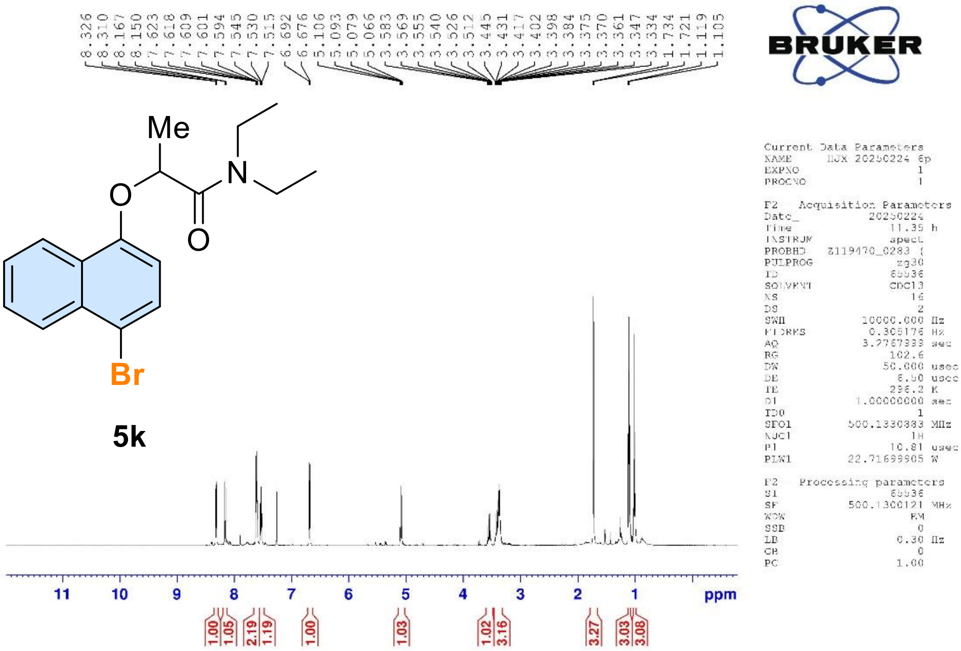

^{13}C NMR

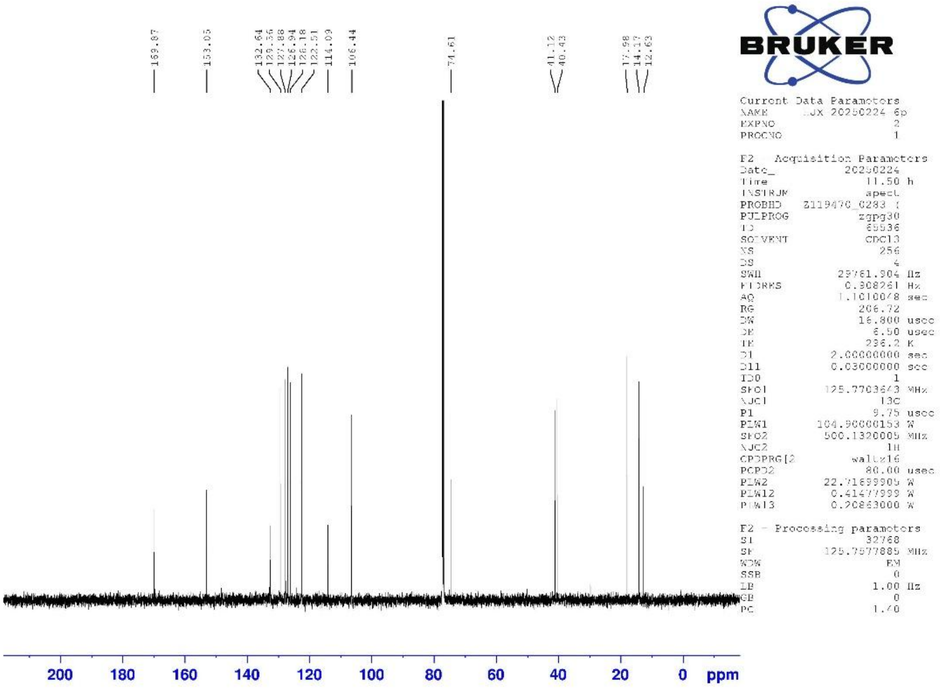

^{1}H NMR

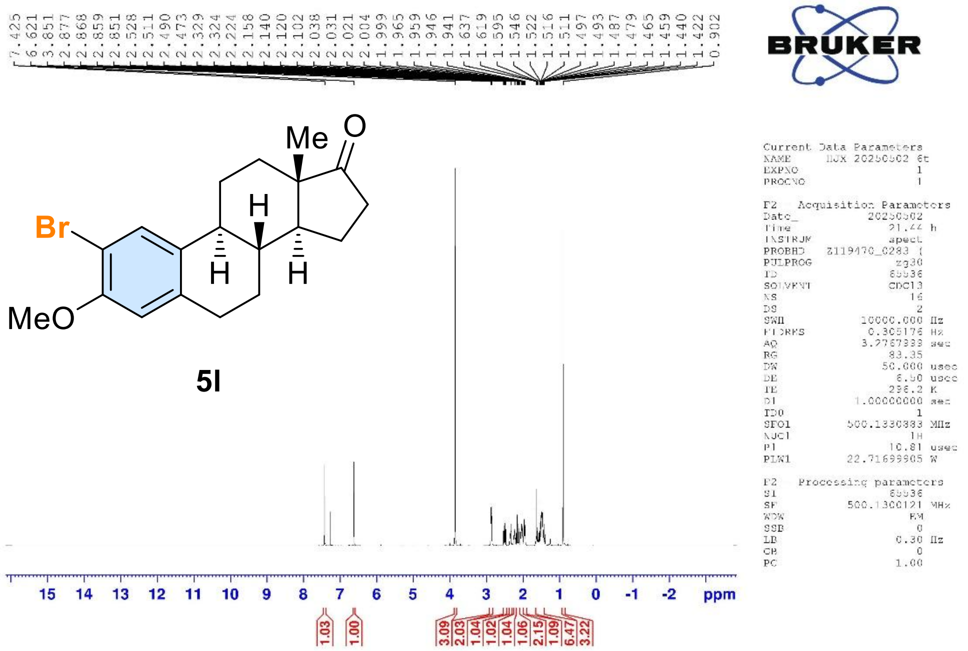

5l

^{13}C NMR

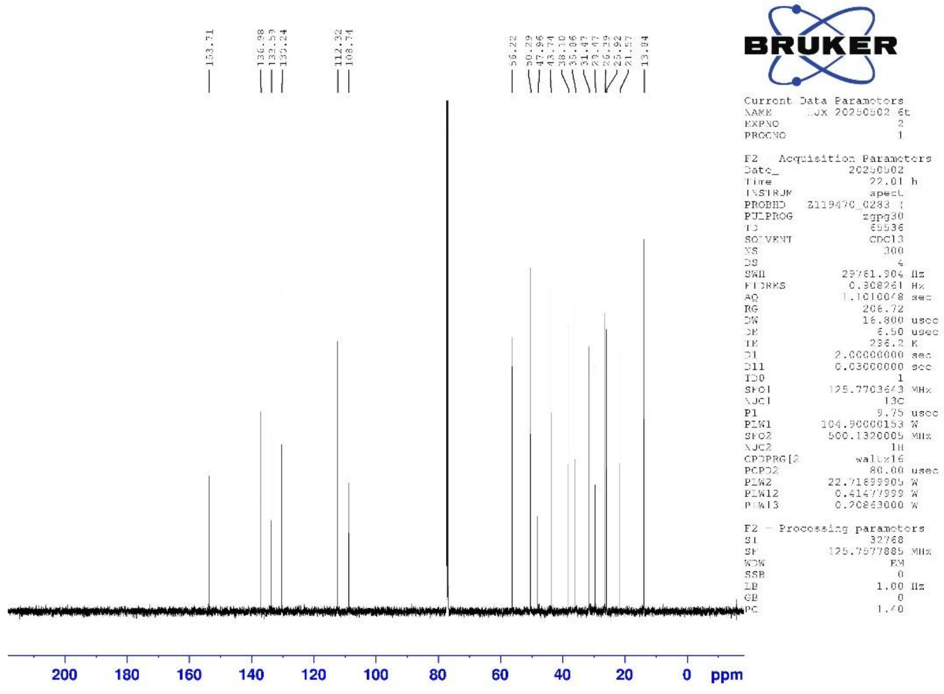

^{1}H NMR

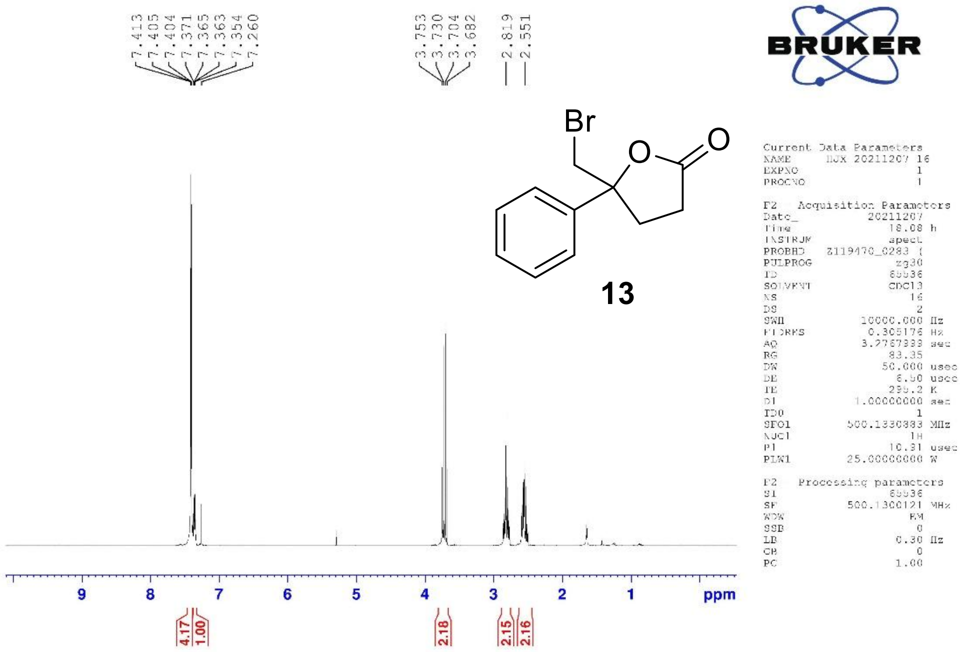

^{13}C NMR

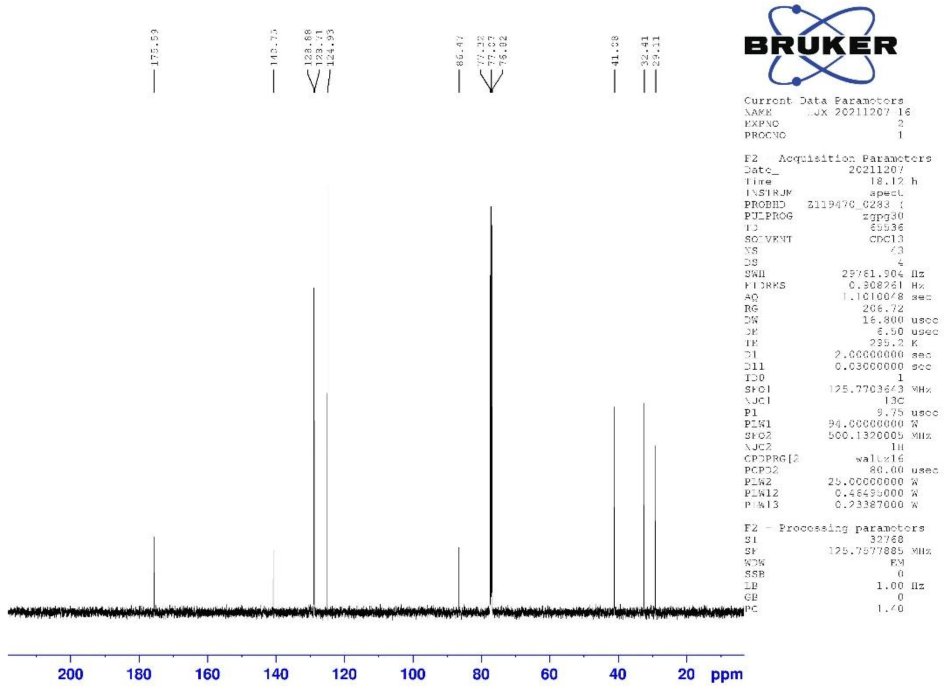

^{1}H NMR

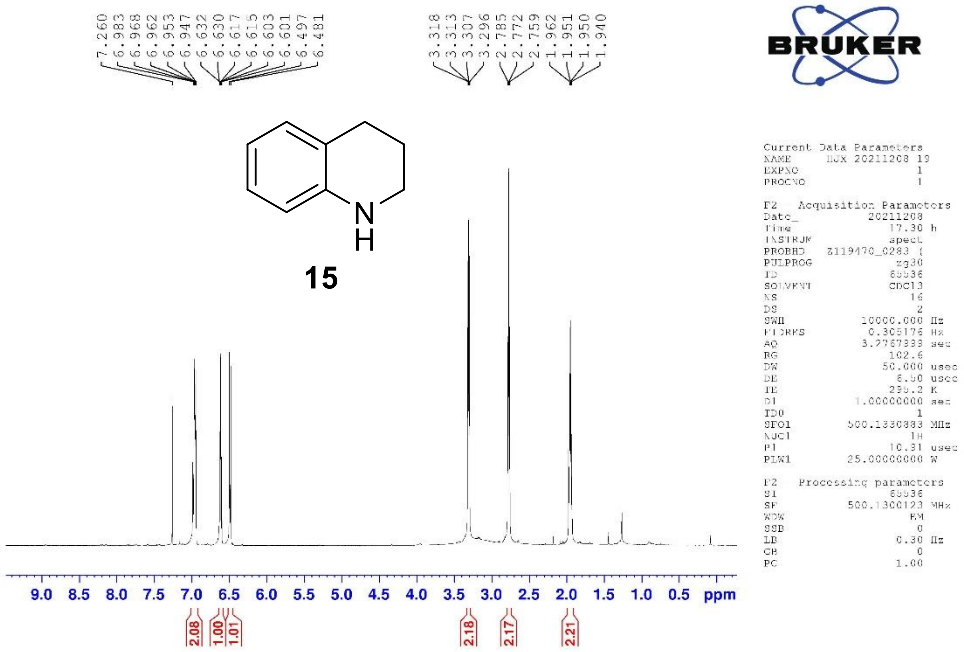

^{13}C NMR

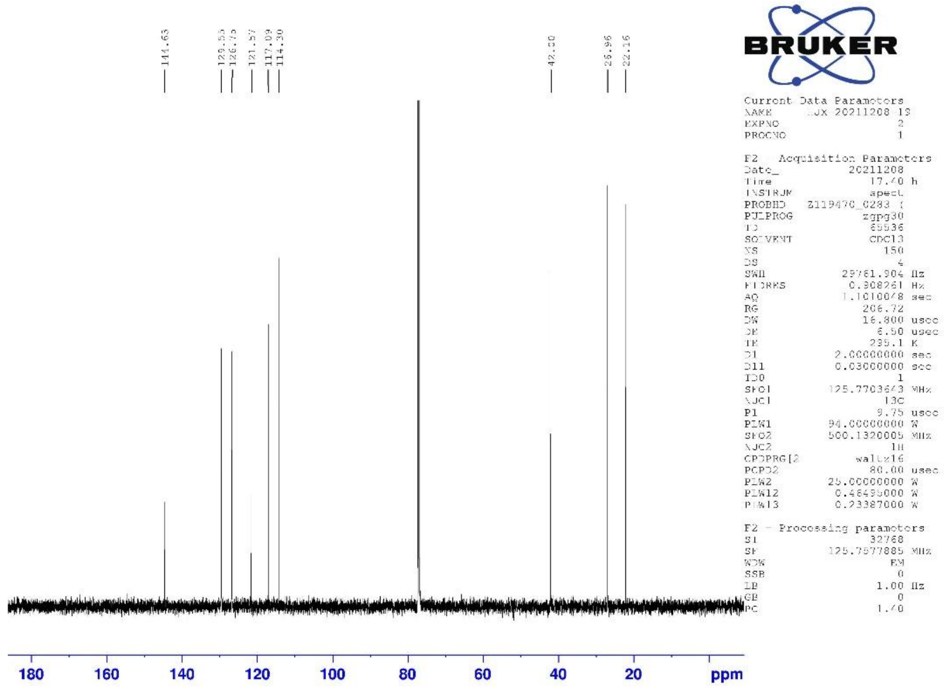

^{1}H NMR

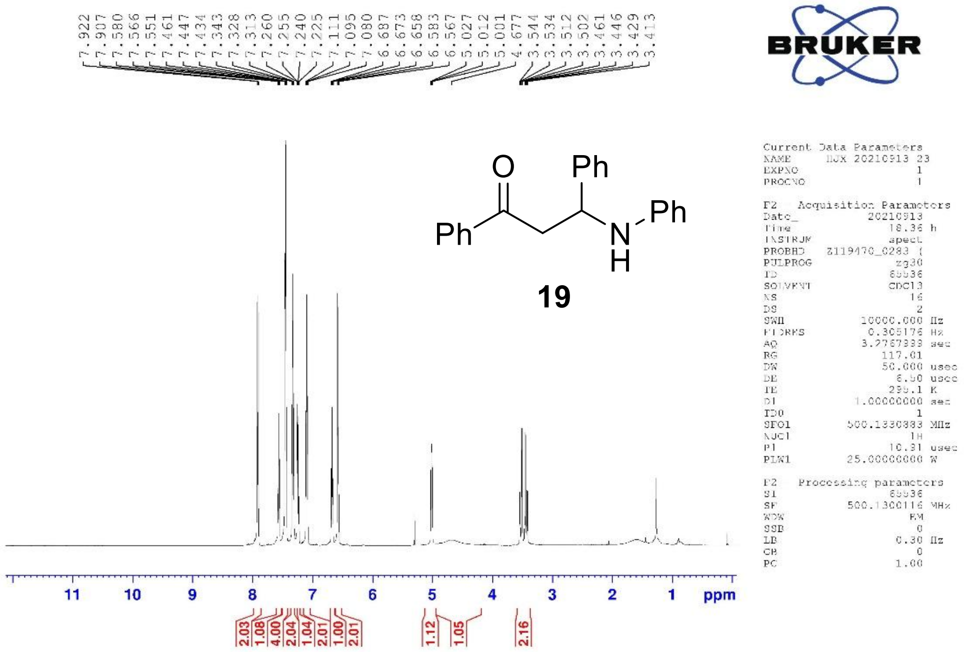

^{13}C NMR

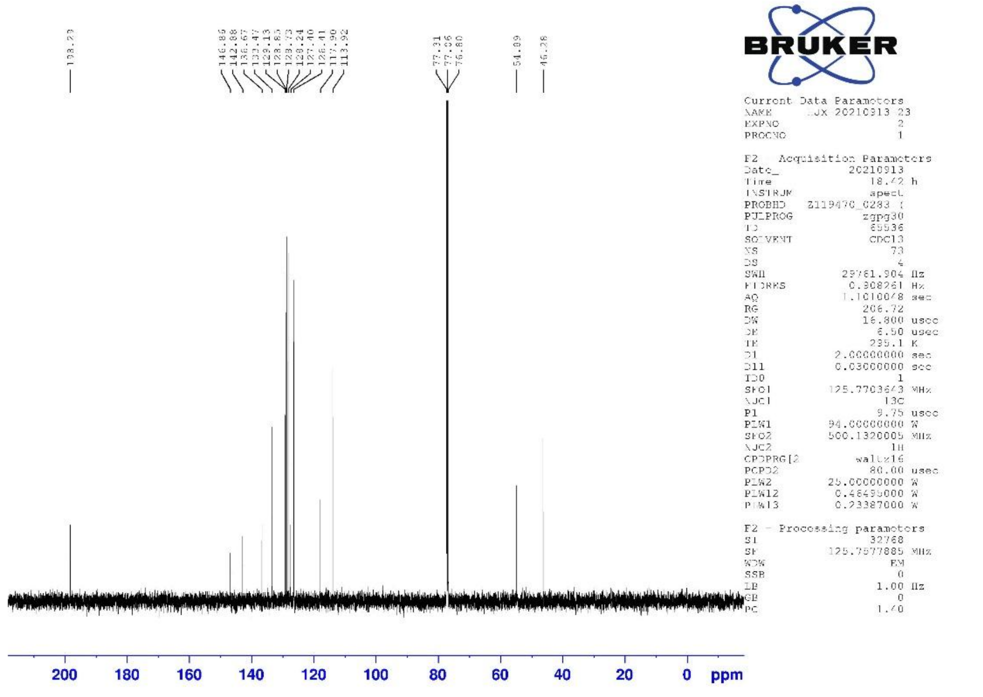

Supplement: Supplementary file 1 — Supporting Information [file ANIE-64-e202511770-s002.pdf]
